# Supplementary material for: Bisguanidinium dinuclear oxodiperoxomolybdosulfate ion pair-catalyzed enantioselective sulfoxidation
Source: Nat Commun. 2016 Nov 21;7:13455. doi: 10.1038/ncomms13455 (PMC5121337; doi:10.1038/ncomms13455)
Supplement: Supplementary Information — Supplementary Figures 1-96, Supplementary Tables 1-5, Supplementary Notes 1-2, Supplementary Methods and Supplementary References. [file ncomms13455-s1.pdf]

## Supplementary Figures

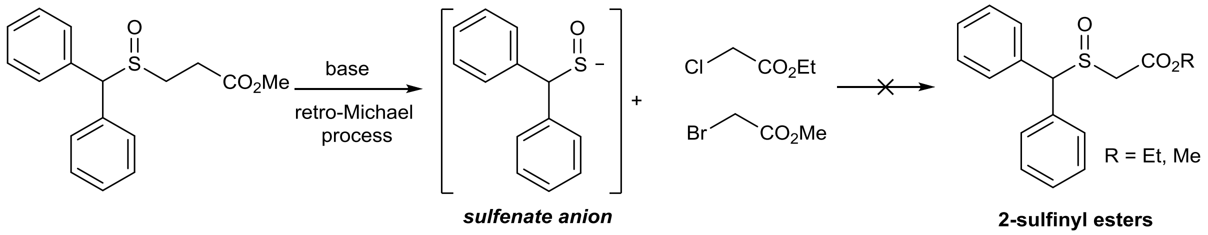


**Supplementary Figure 1.** Unsuccessful attempt to 2-sulfinyl esters by using sulfenate anion strategy.

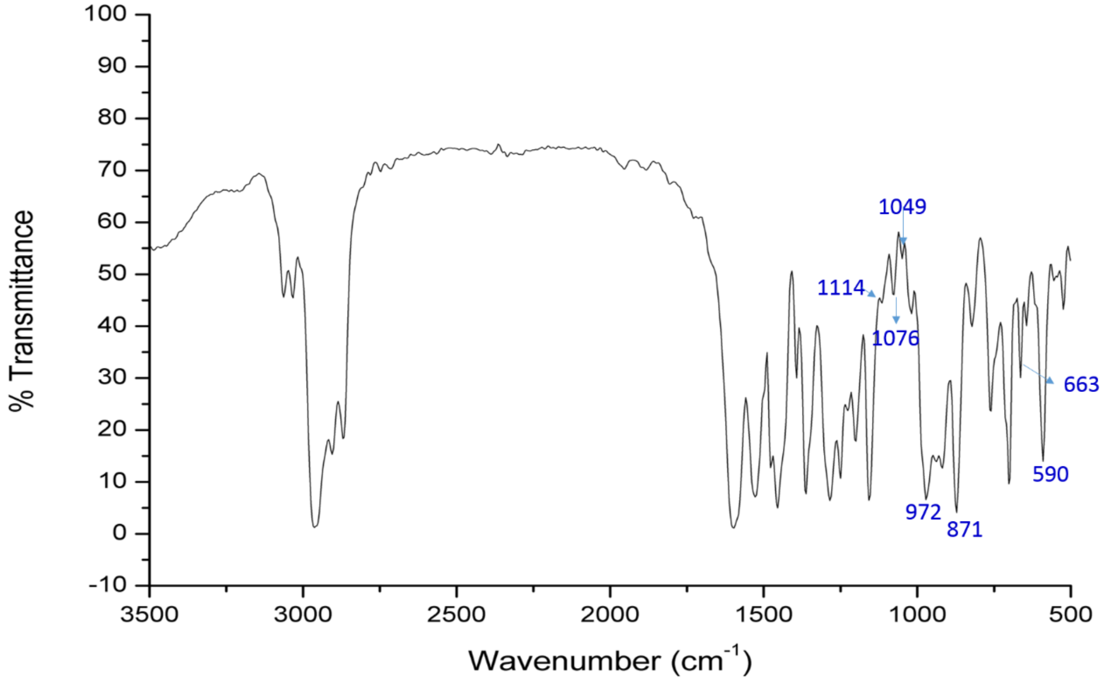


**Supplementary Figure 2.** Infrared spectrum of (*R*,*R*)-**1b**.

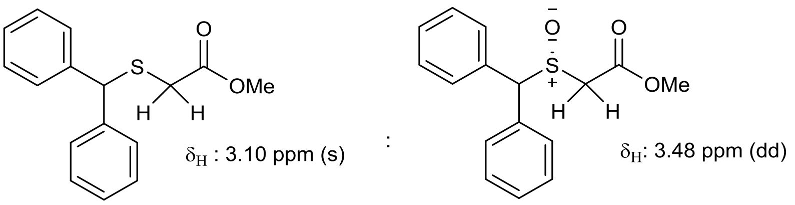


**2a** **ent-3a**

**2a**: ***ent*-3a** = 1: 4

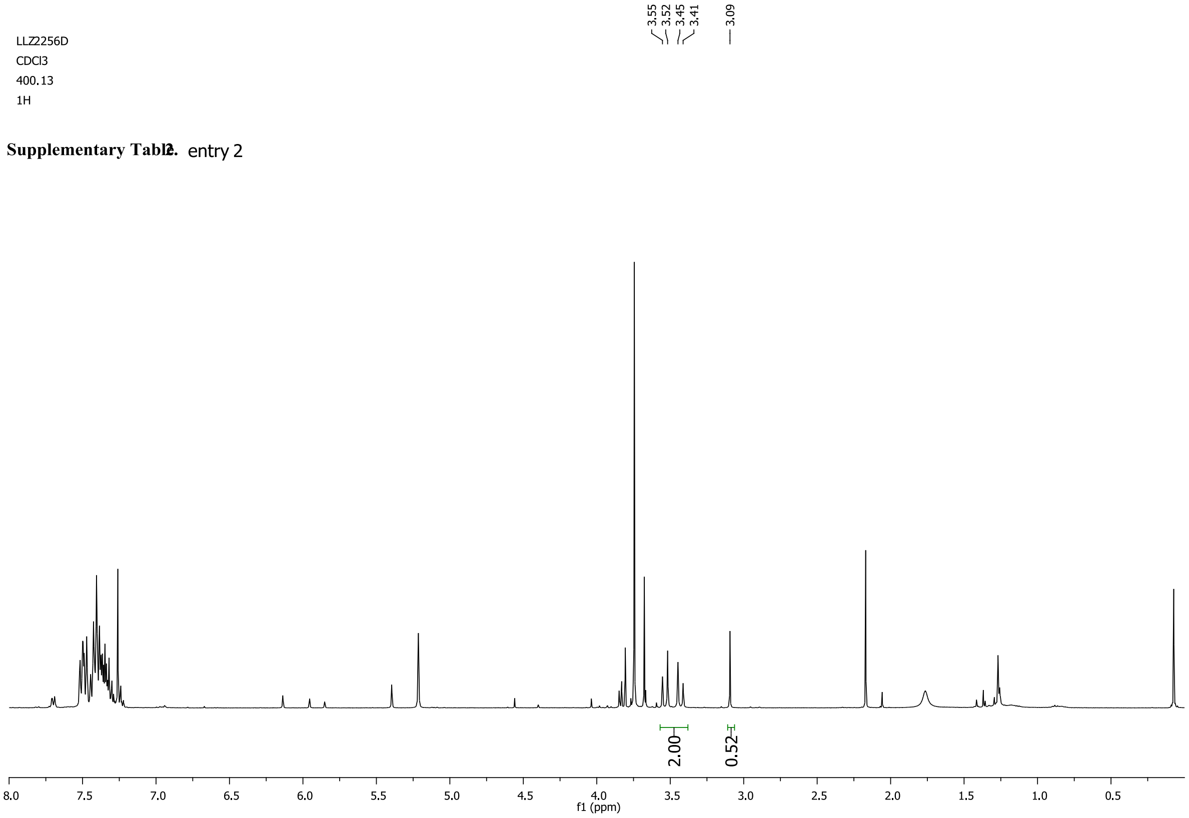


**Supplementary Figure 3.** $^{1}$H NMR spectrum of crude reaction mixture by using 0.5 equivalent of (*R,R*)-**1b**.

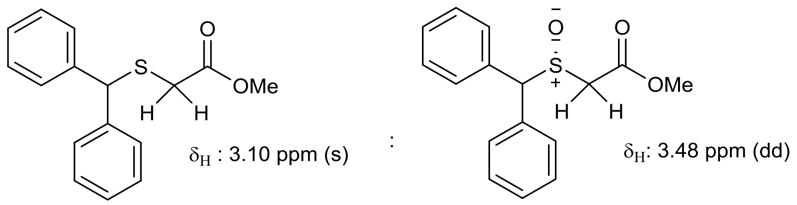


**2a** **_ent_-3a**

**2a**: ***ent*-3a** = 1: 1

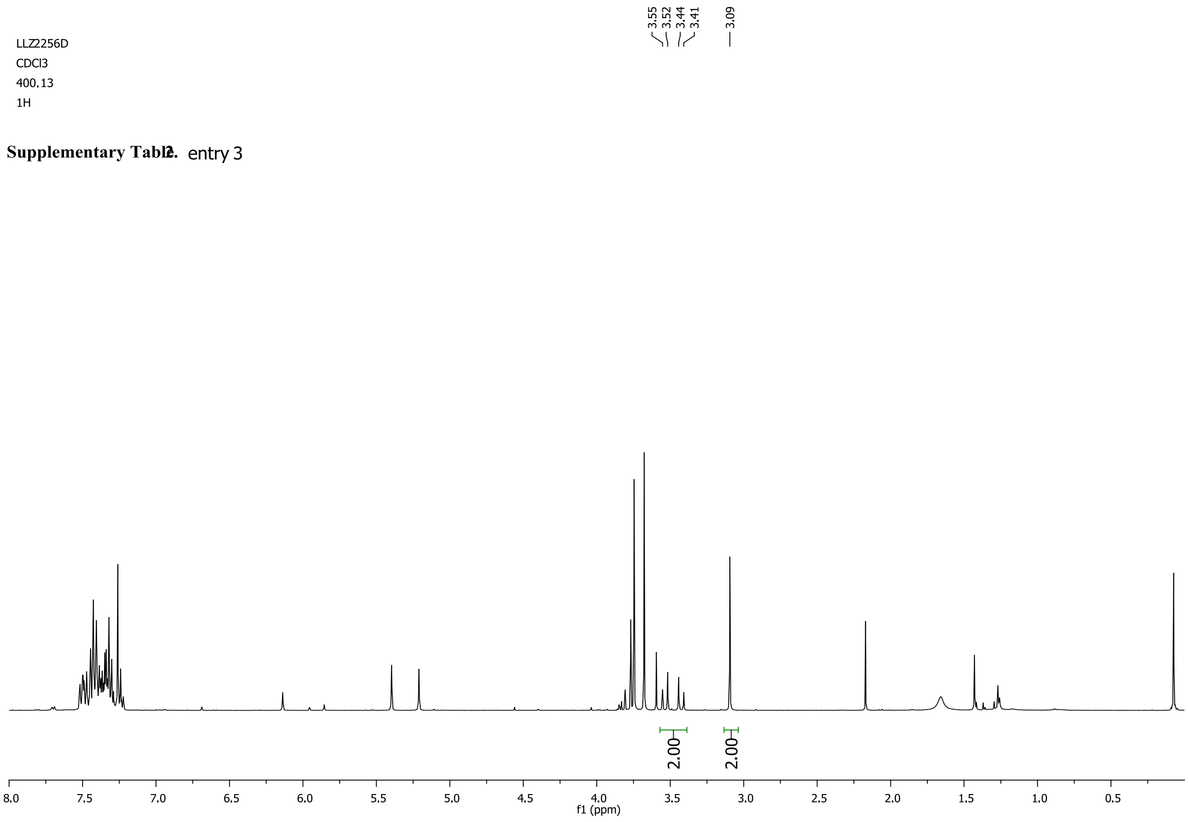


**Supplementary Figure 4.** $^{1}$H NMR spectrum of crude reaction mixture using 0.25 equivalent of (*R,R*)-**1b**.

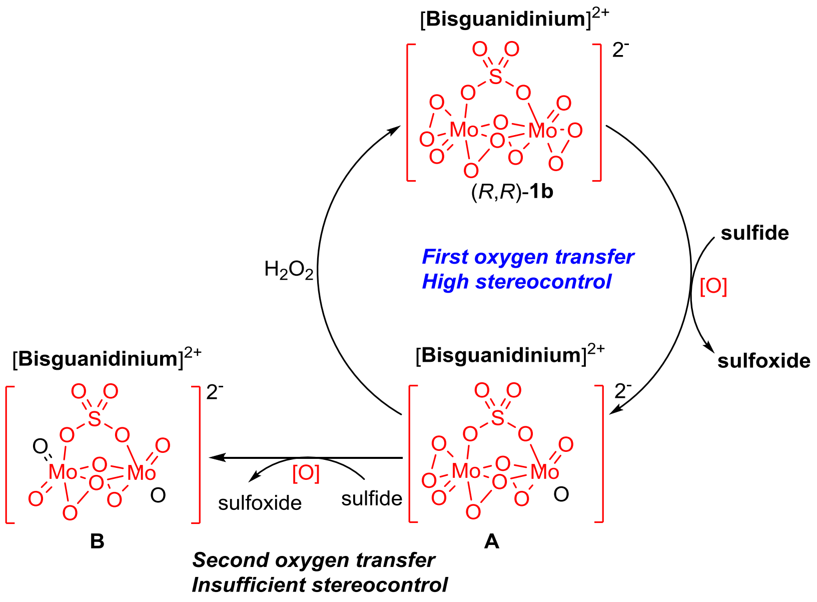


**Supplementary Figure 5.** Plausible mechanistic cycle of catalyst (*R*,*R*)-**1b** with $H_2O_2$ as oxidant.

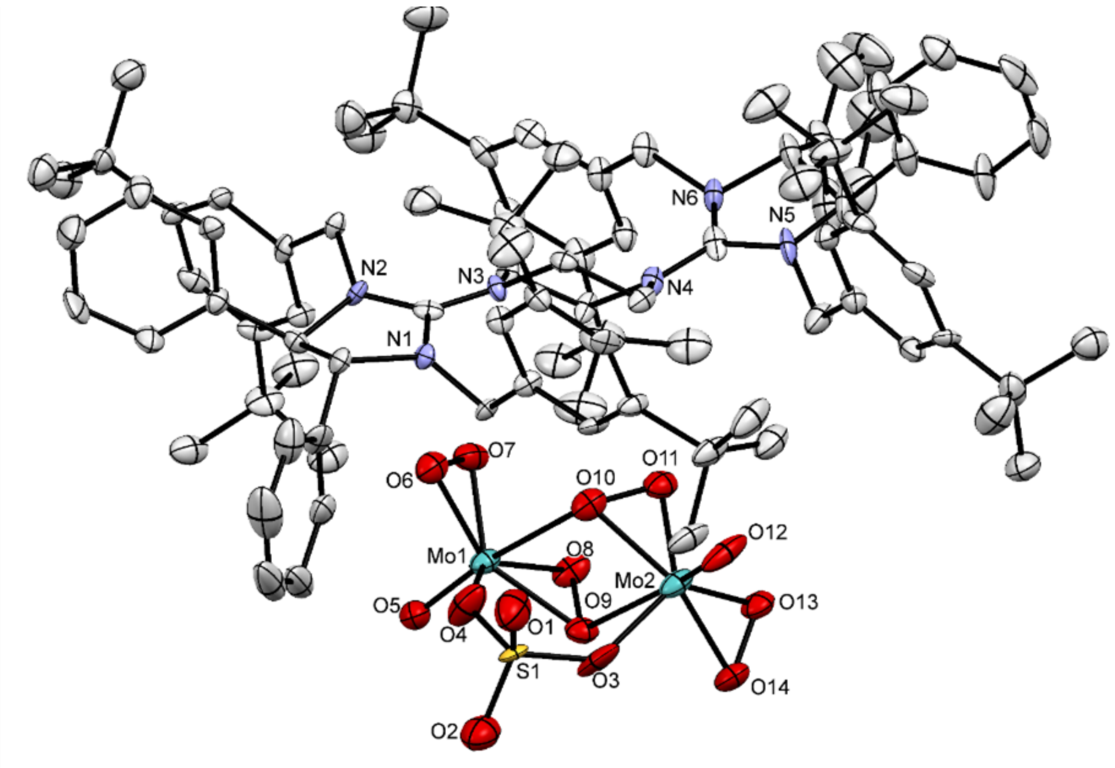


**Supplementary Figure 6.** X-ray structure of catalyst (*R*,*R*)-**1b**.

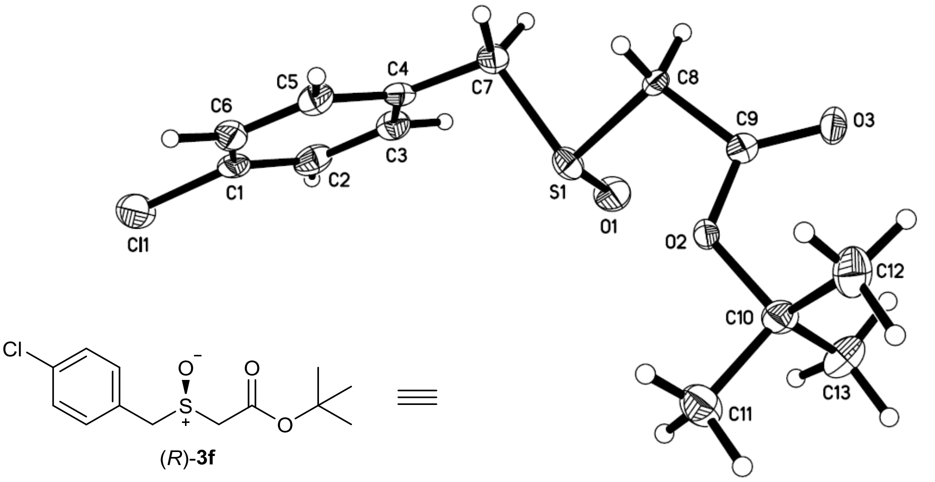


**Supplementary Figure 7.** X-ray structure of compound (*R*)-**3f**.

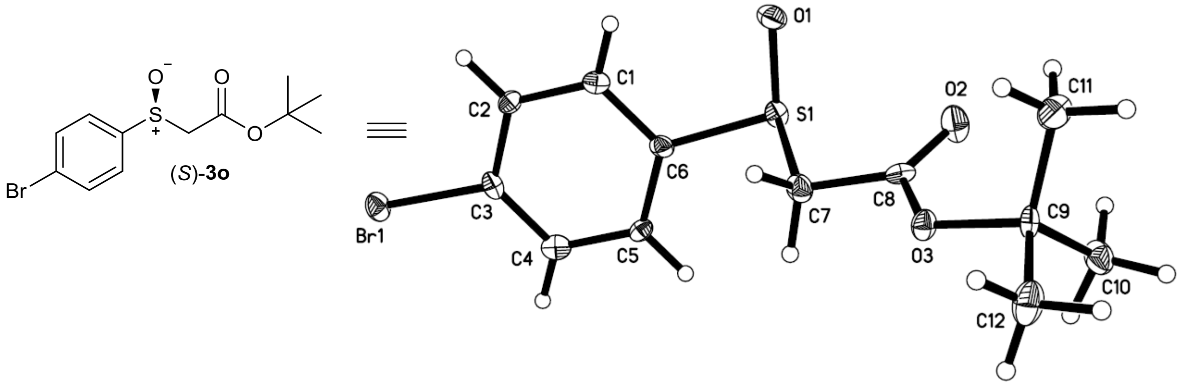


**Supplementary Figure 8.** X-ray structure of compound (*S*)-**3o**.

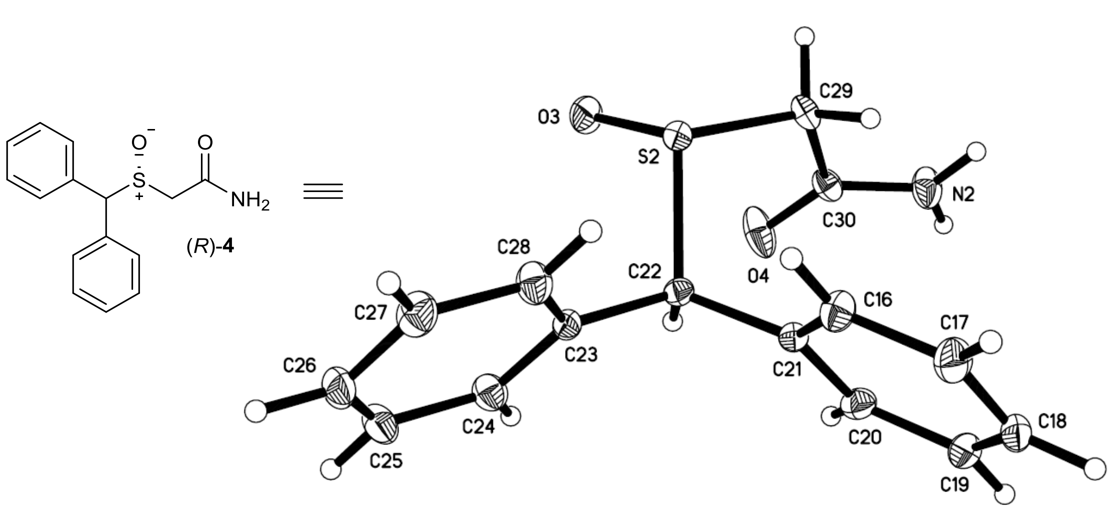


**Supplementary Figure 9.** X-ray structure of compound (*R*)-**4**.

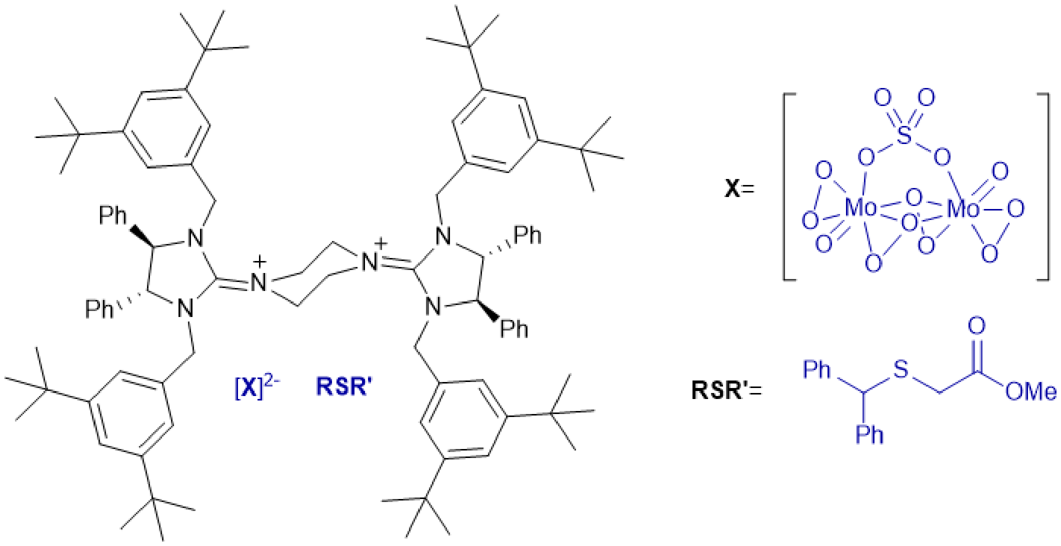


**Supplementary Figure 10.** ONIOM partitioning used in multiscale calculations. Blue-colored atoms are included in the high level (DFT) layer, and a bisguanidinium group is included in the system but outside the DFT layer (and thus treated only by PM6). An anionic $[Mo_2O_2(O_2)_4SO_4]^{2-}$ cluster and a model sulfide substrate (shown in blue) are included in the high level (B3LYP/B1) layer.

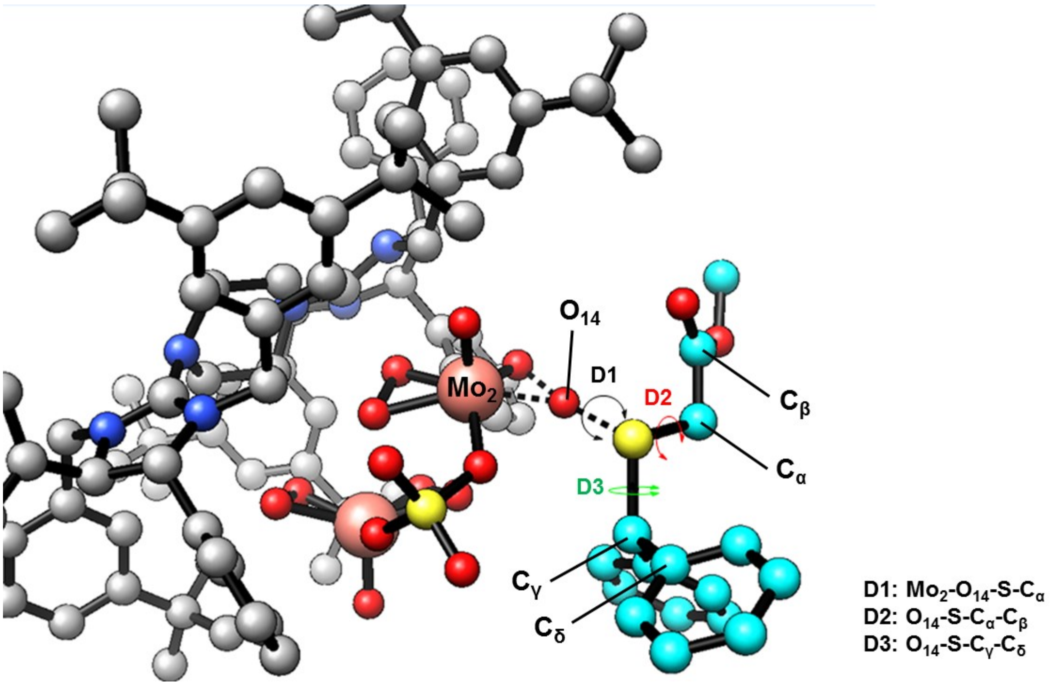


**Supplementary Figure 11.** TS conformational sampling methodology.

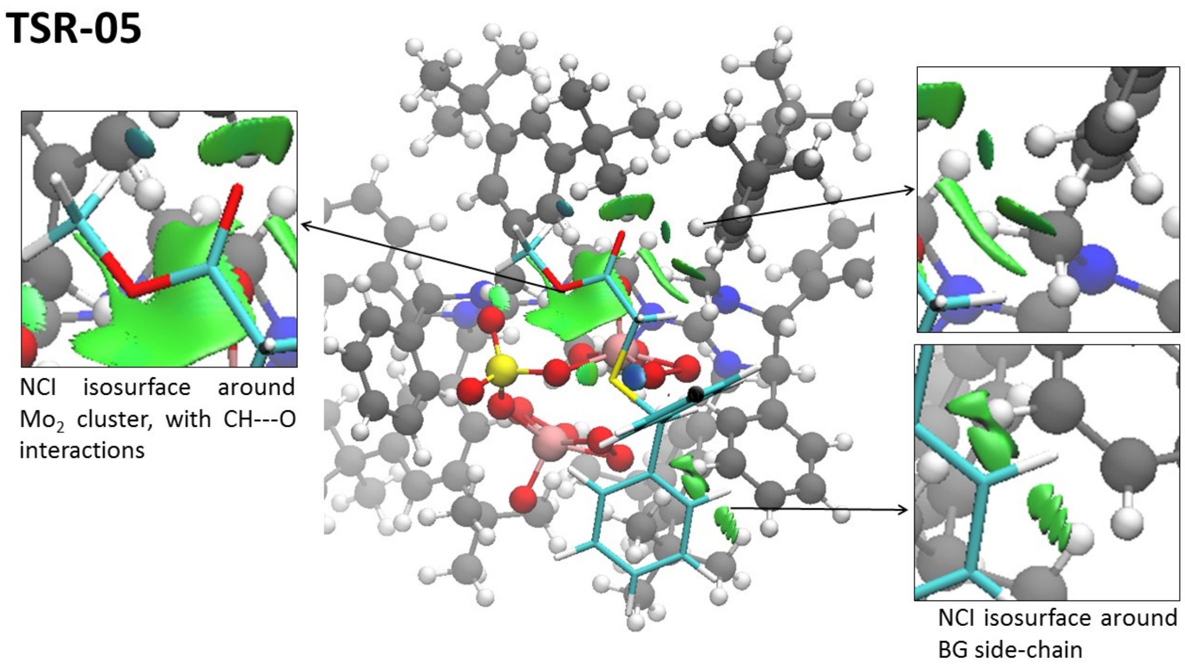


**Supplementary Figure 12.** NCI isosurface of TSR-05 which is the most stable TSs leading to *R* product.

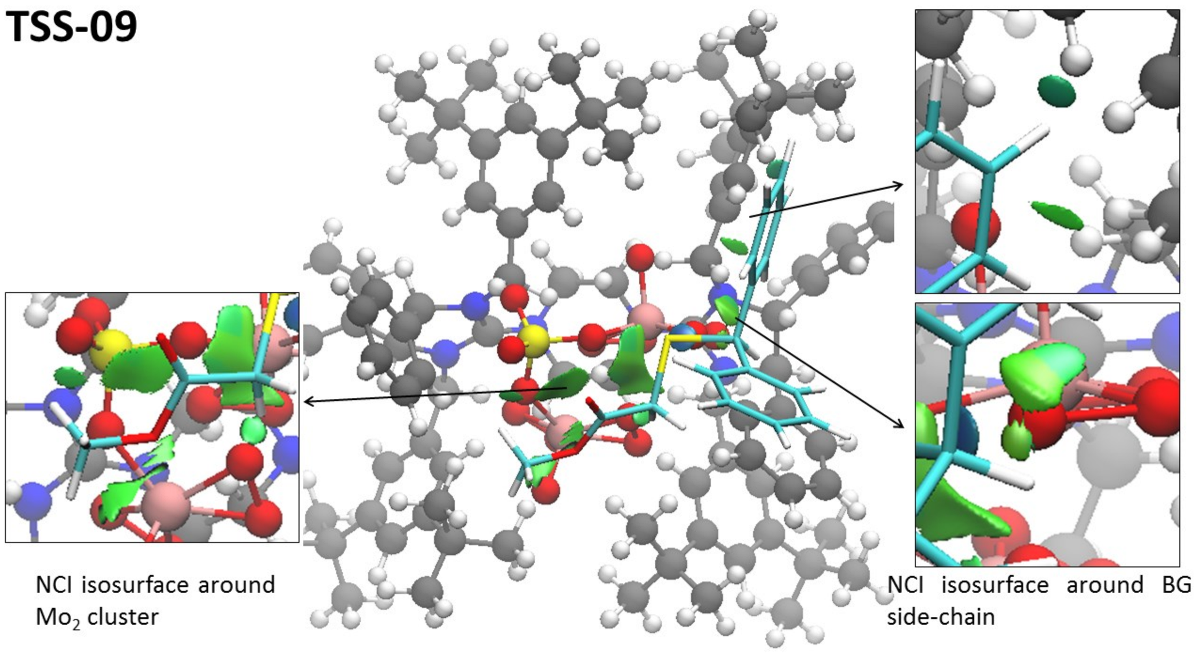


**Supplementary Figure 13.** NCI isosurface of TSS-09 which is the most stable TSs leading to *S* product.

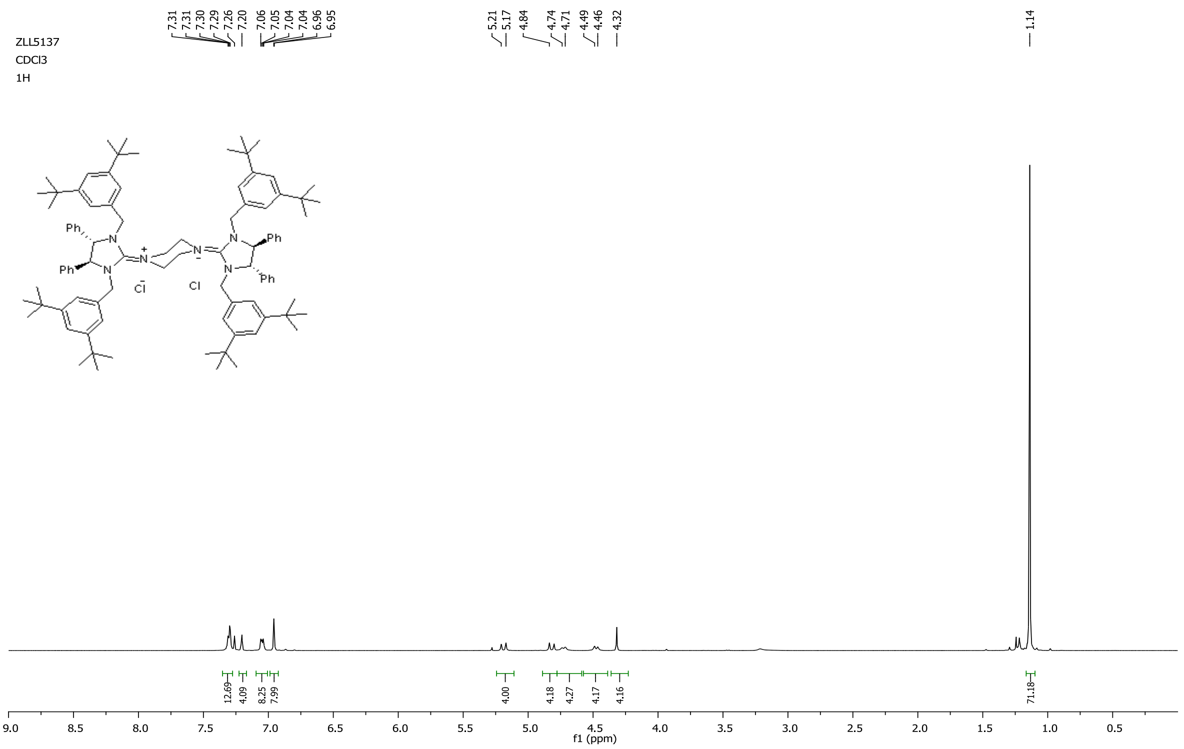


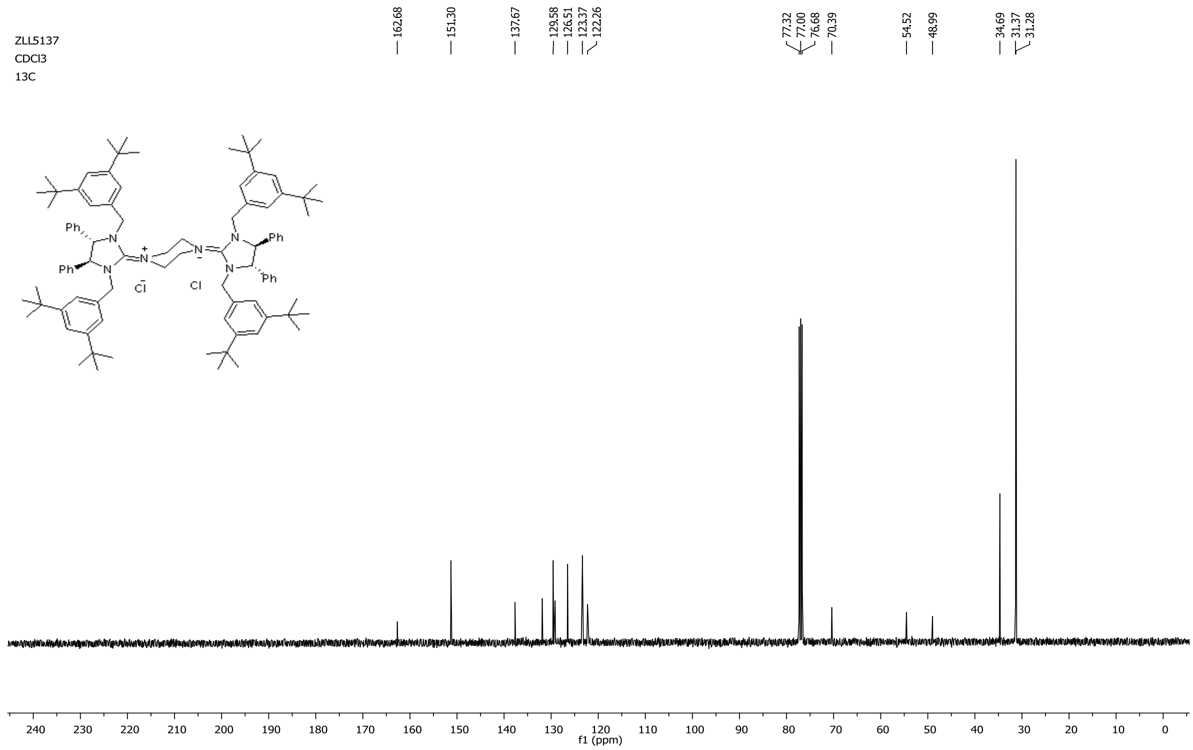


**Supplementary Figure 14.** $^{1}H$ and $^{13}C$ NMR spectra of catalyst (*S*,*S*)-**1a**.

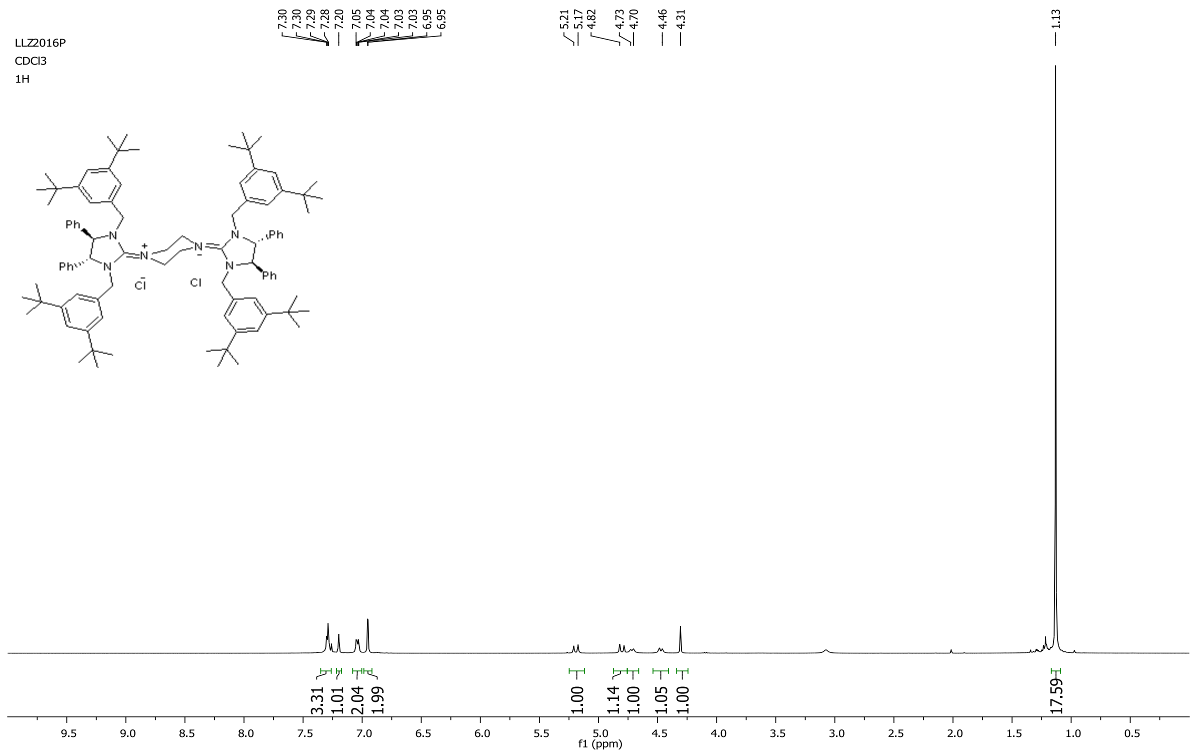


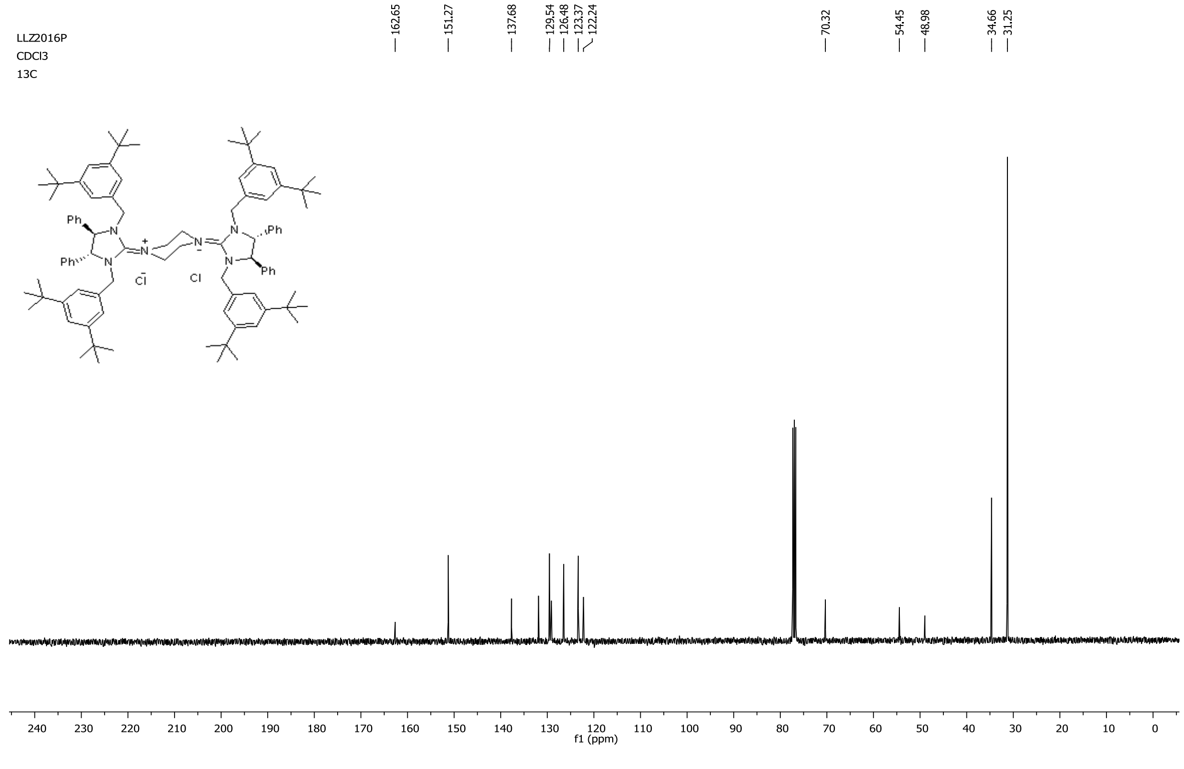


**Supplementary Figure 15.** $^{1}$H and $^{13}$C NMR spectra of catalyst (*R*,*R*)-**1a**.

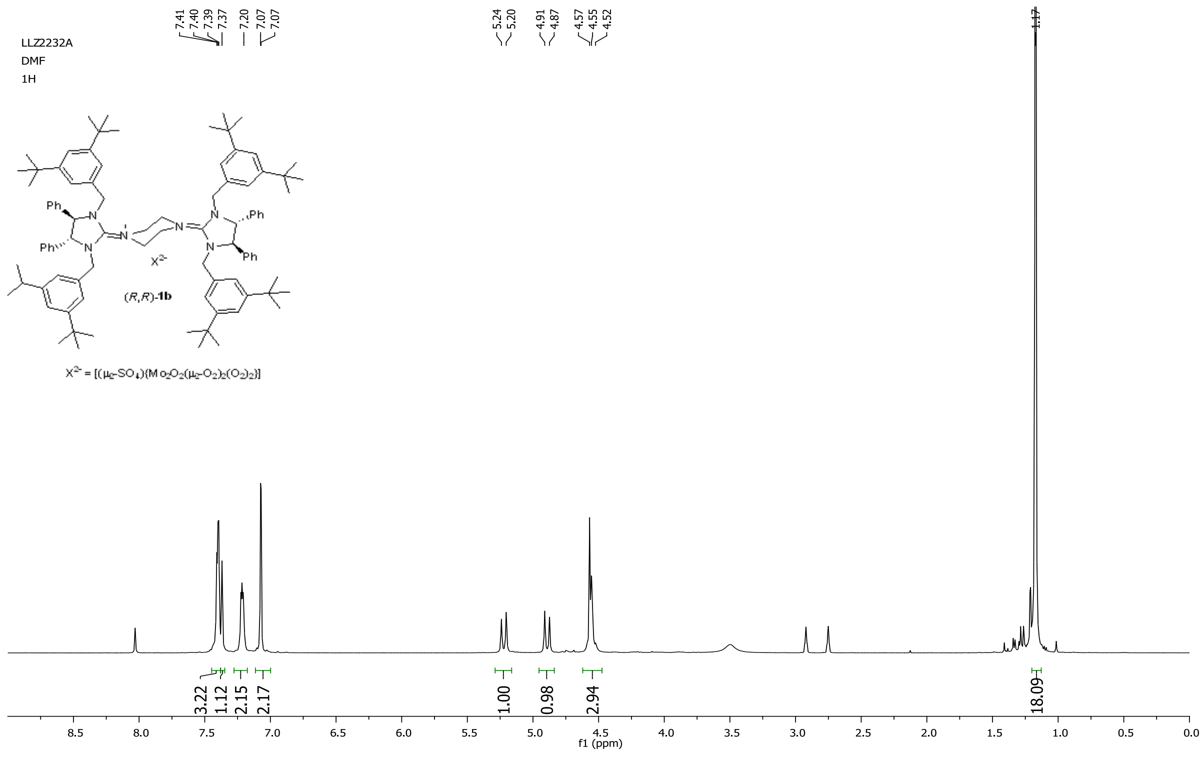


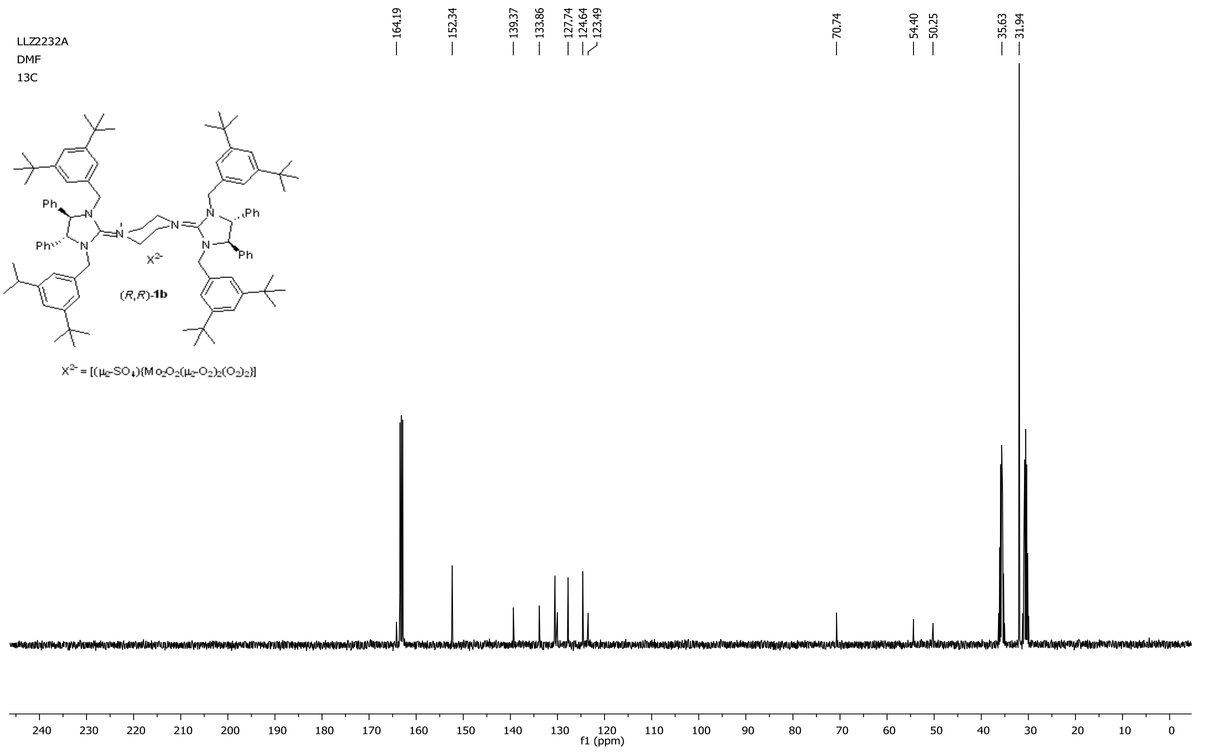


**Supplementary Figure 16.** $^{1}H$ and $^{13}C$ NMR spectra of catalyst (*R*,*R*)-**1b**.

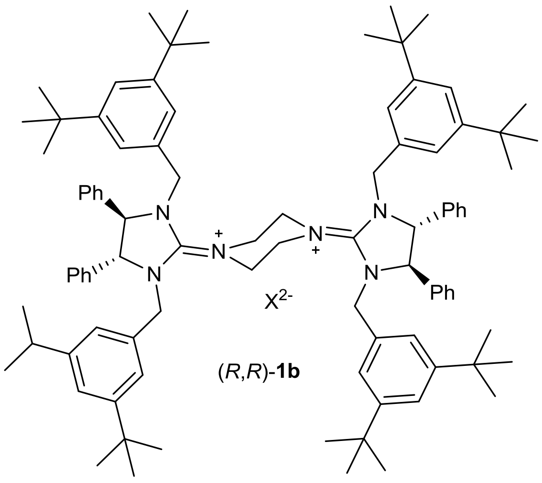


X$^{2-}$ = [($\mu_2$-SO$_4$){Mo$_2$O$_2$($\mu_2$-O$_2$)$_2$(O$_2$)$_2$}]

(*R*,*R*)-**1b**

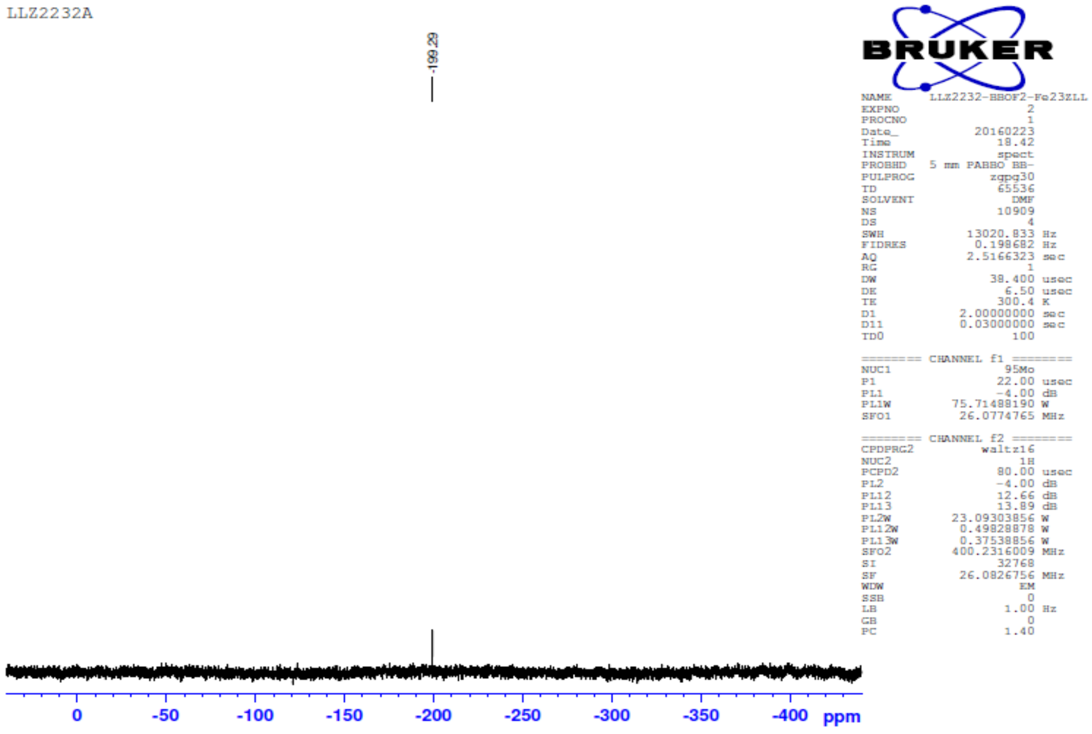


**Supplementary Figure 17.** $^{95}$Mo NMR spectra of catalyst (*R*,*R*)**-1b**.

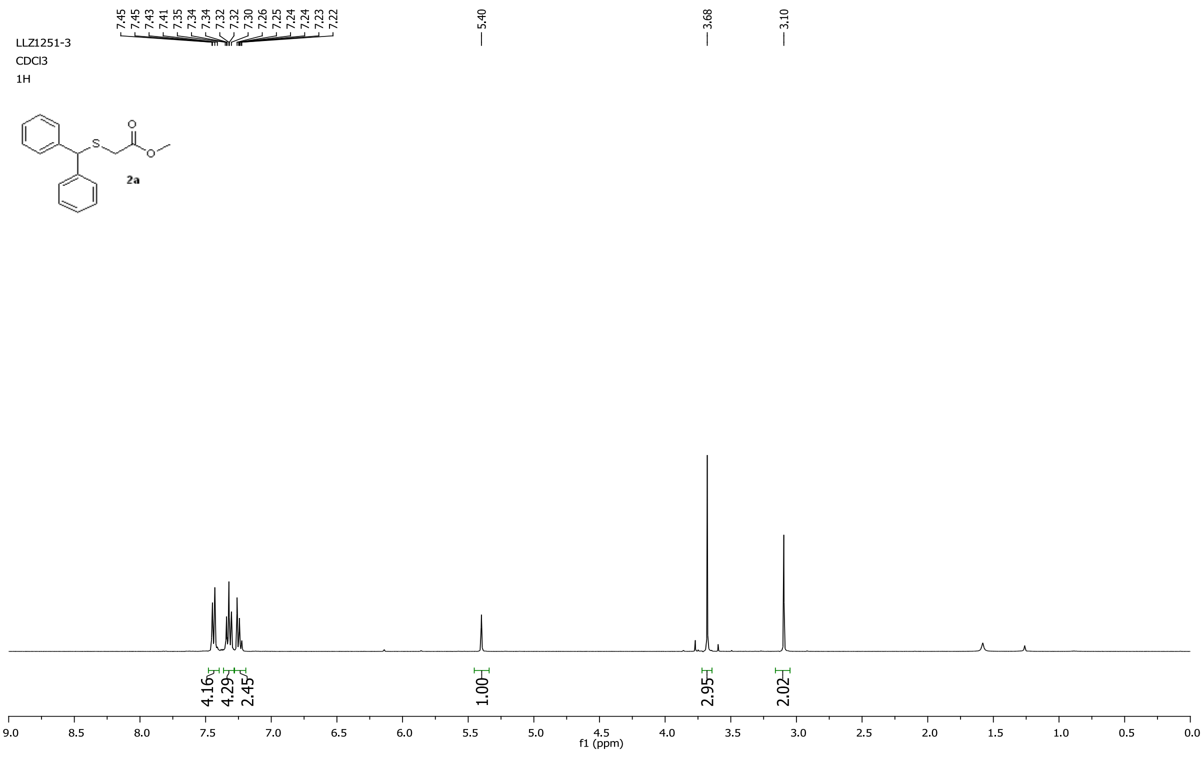


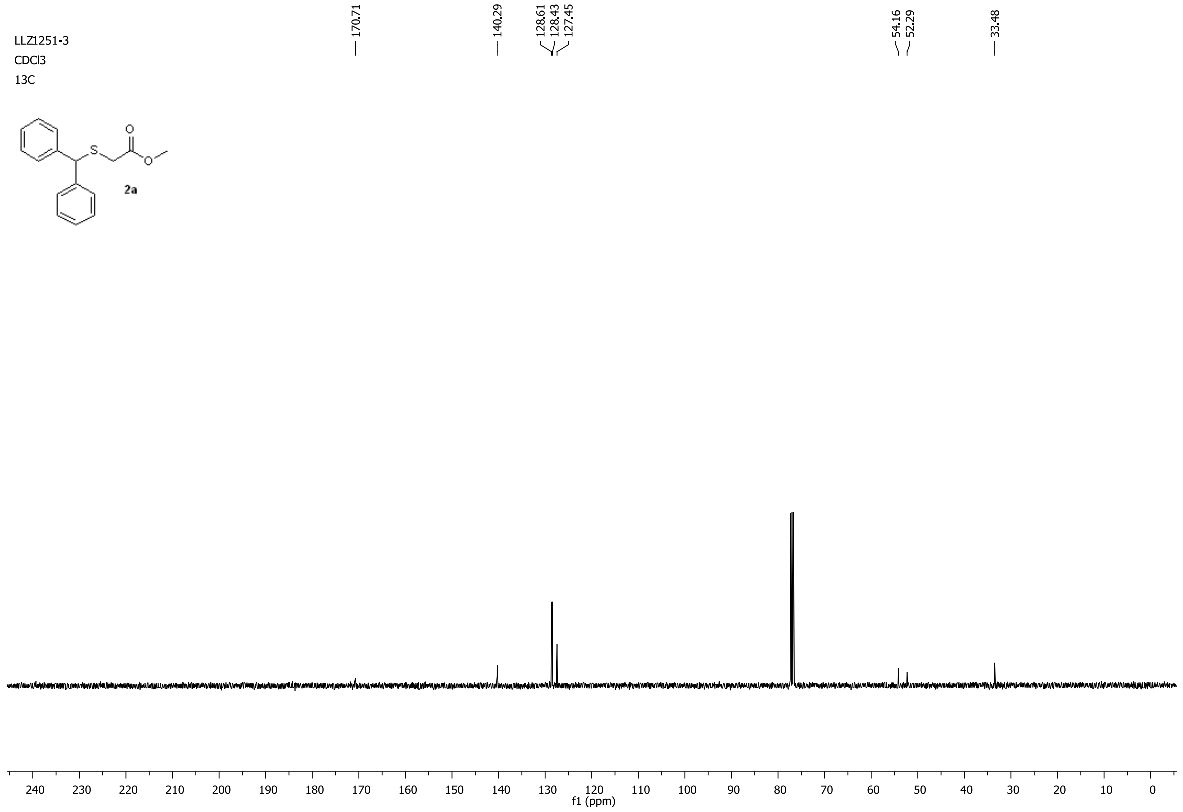


**Supplementary Figure 18.** $^{1}$H and $^{13}$C NMR spectra of sulfide **2a**.

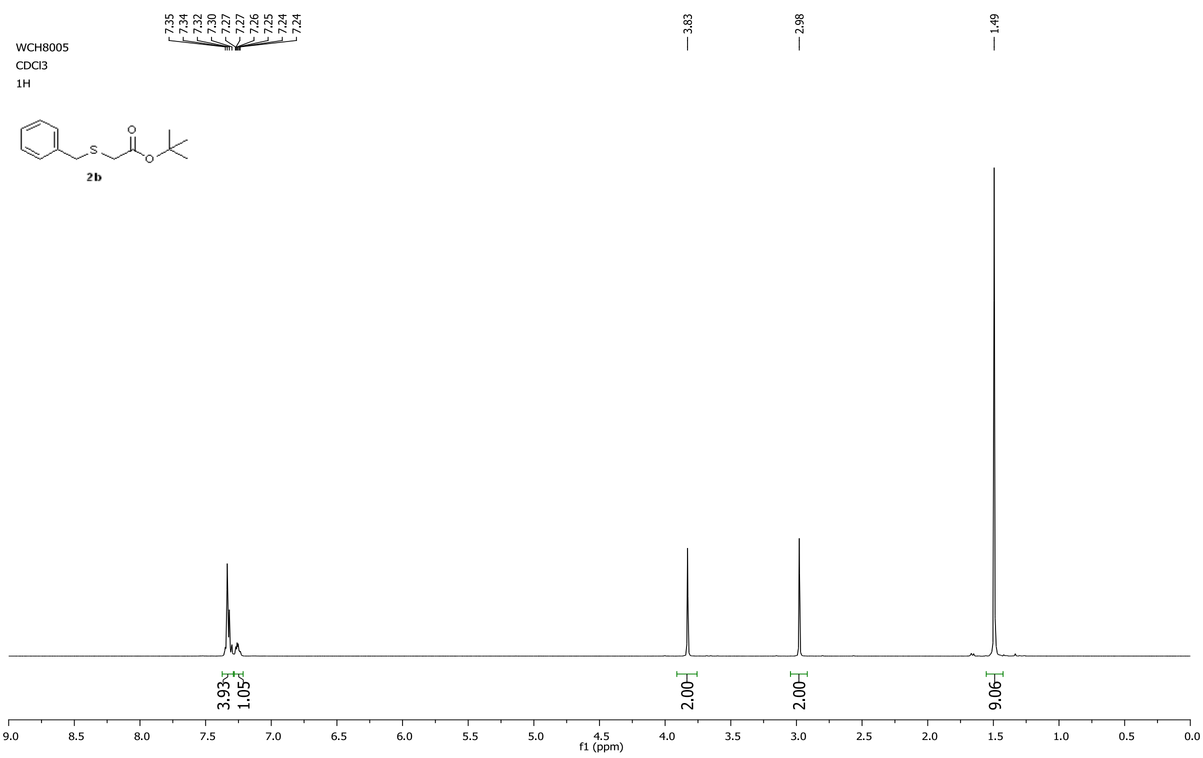


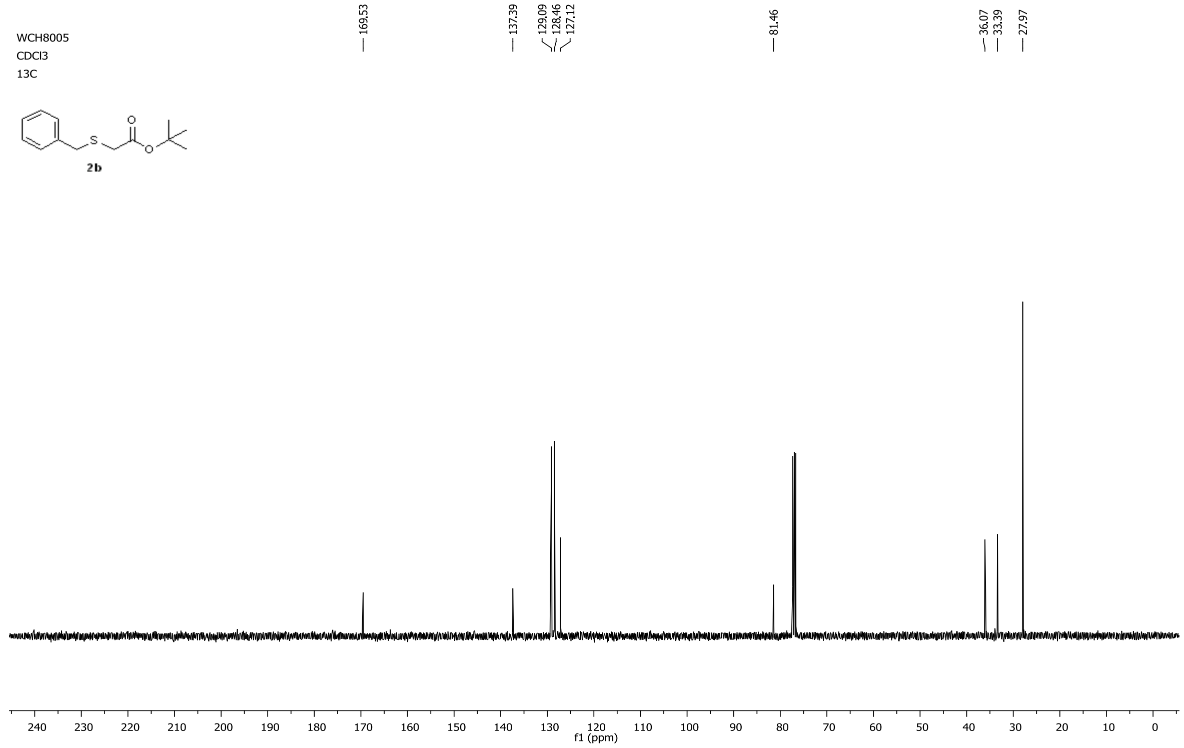


**Supplementary Figure 19.** $^{1}$H and $^{13}$C NMR spectra of sulfide **2b**.

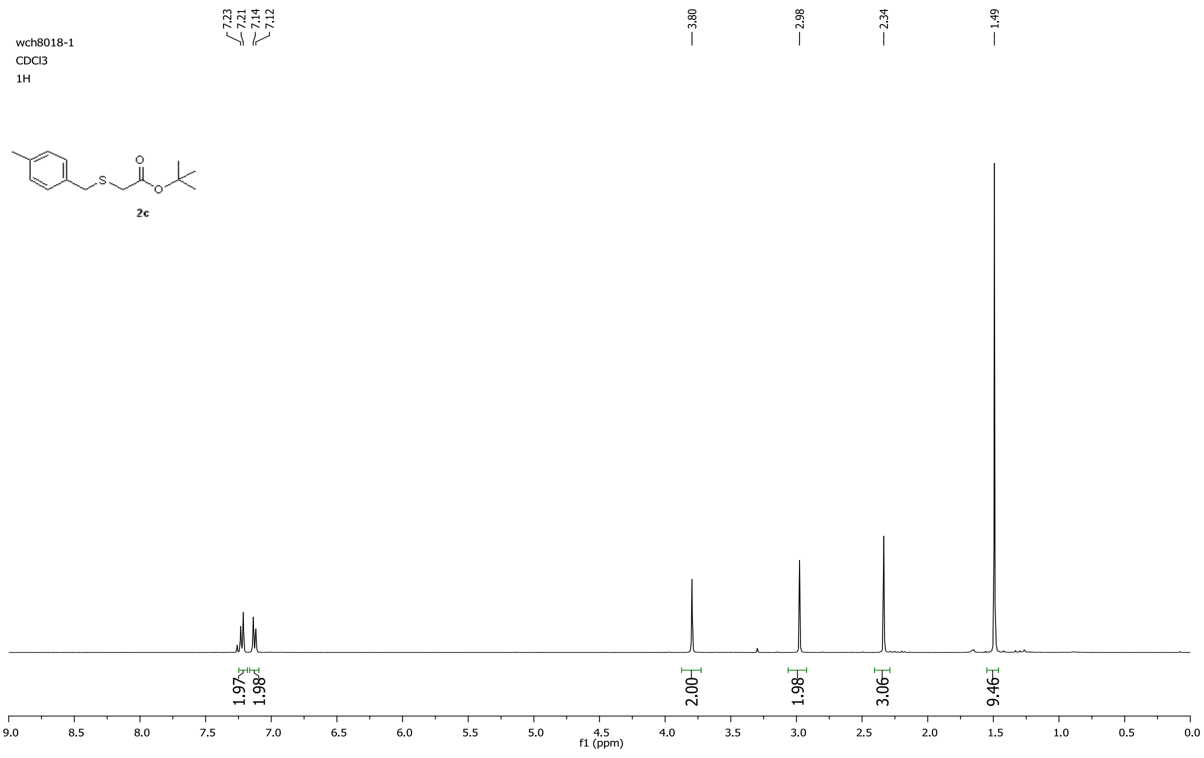


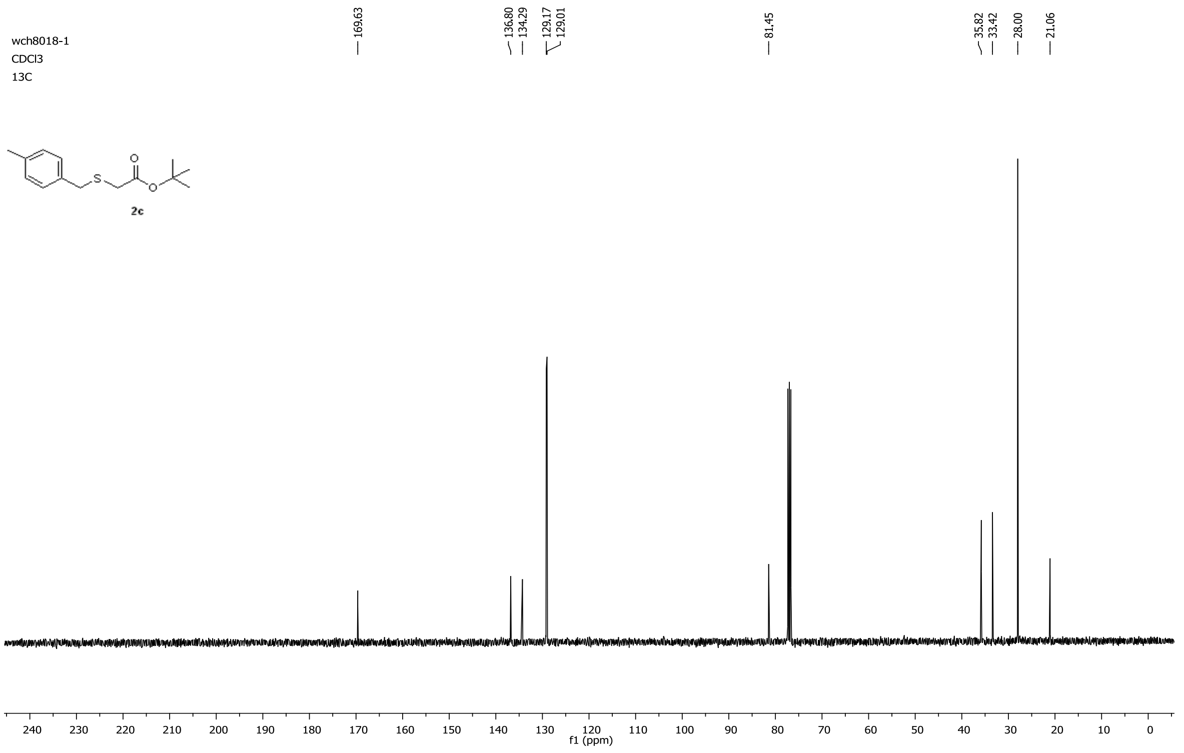


**Supplementary Figure 20.** $^1$H and $^{13}$C NMR spectra of sulfide **2c**.

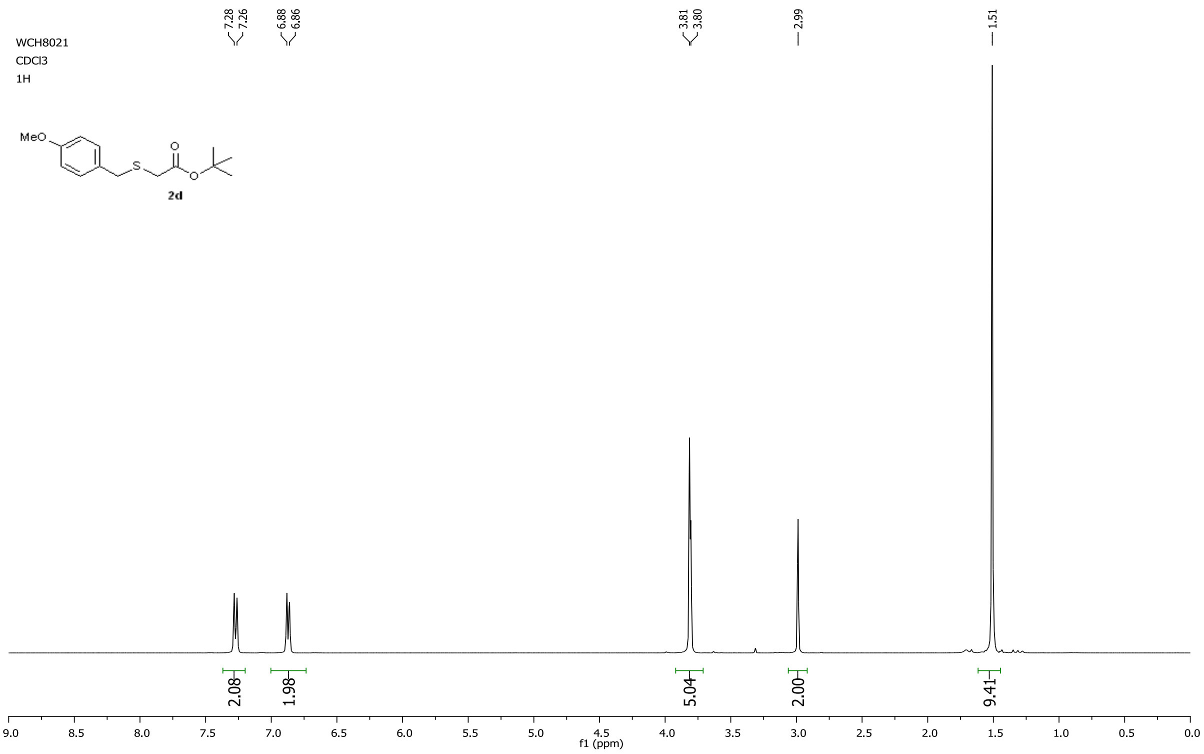


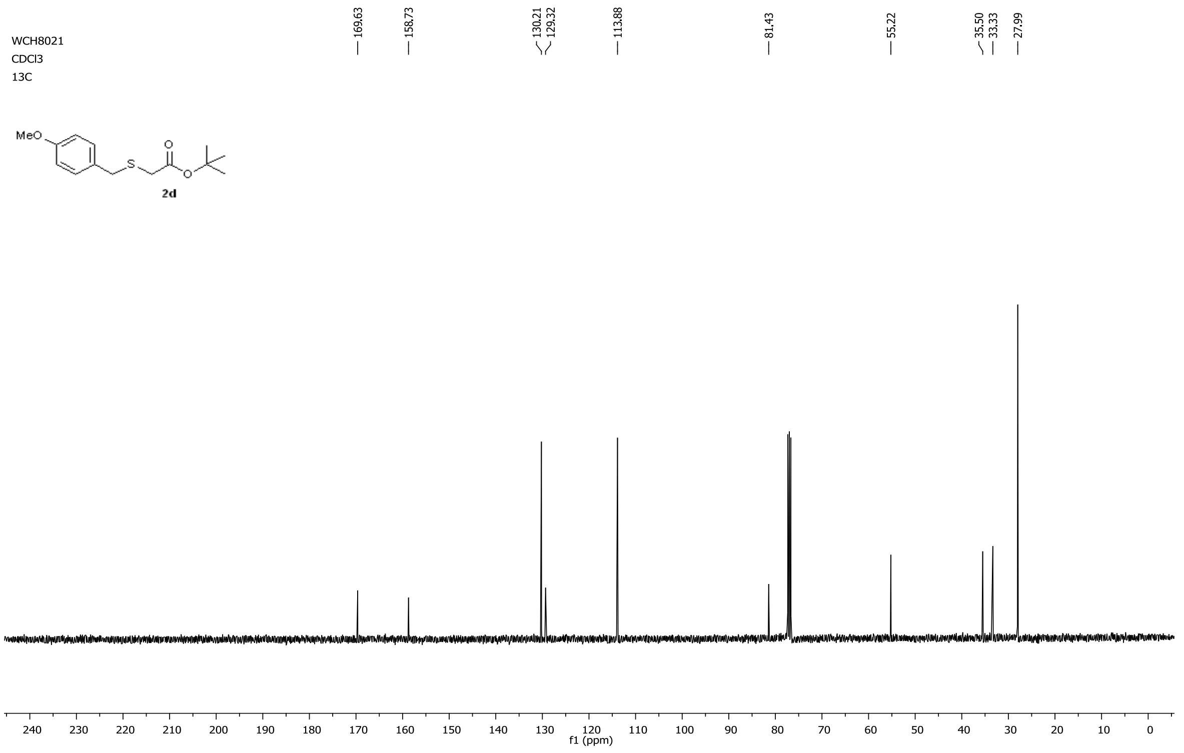


**Supplementary Figure 21.** $^{1}$H and $^{13}$C NMR spectra of sulfide **2d**.

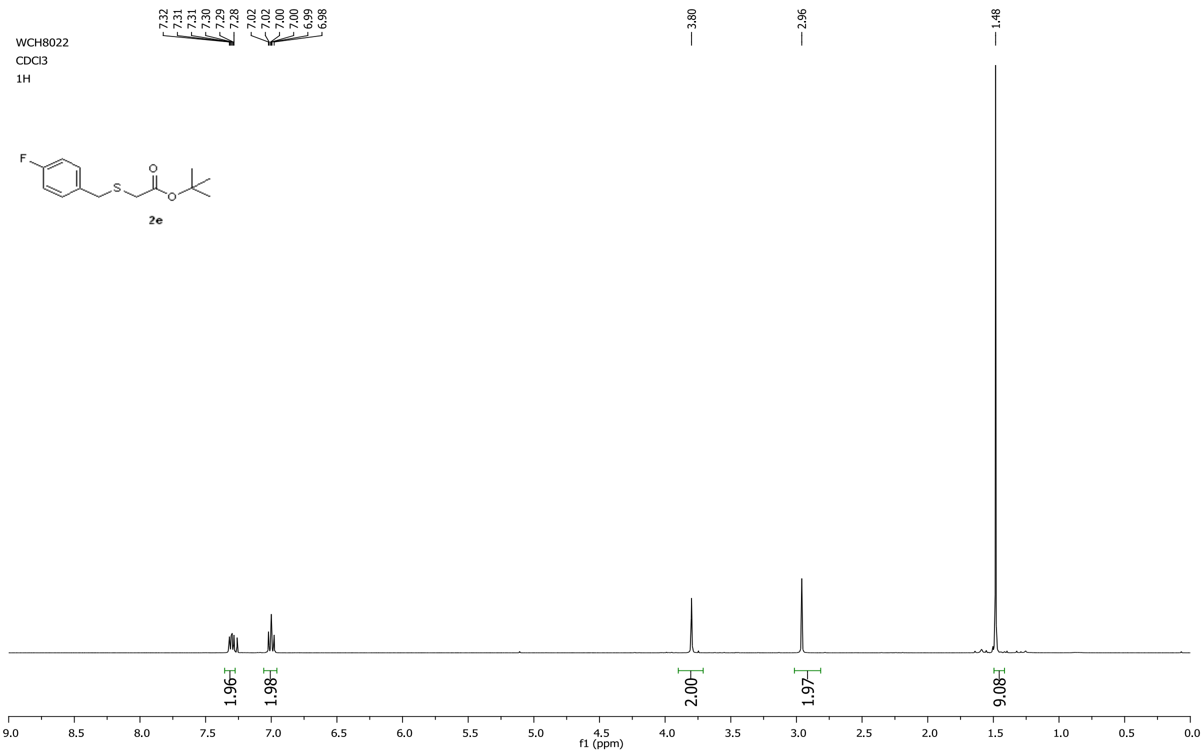


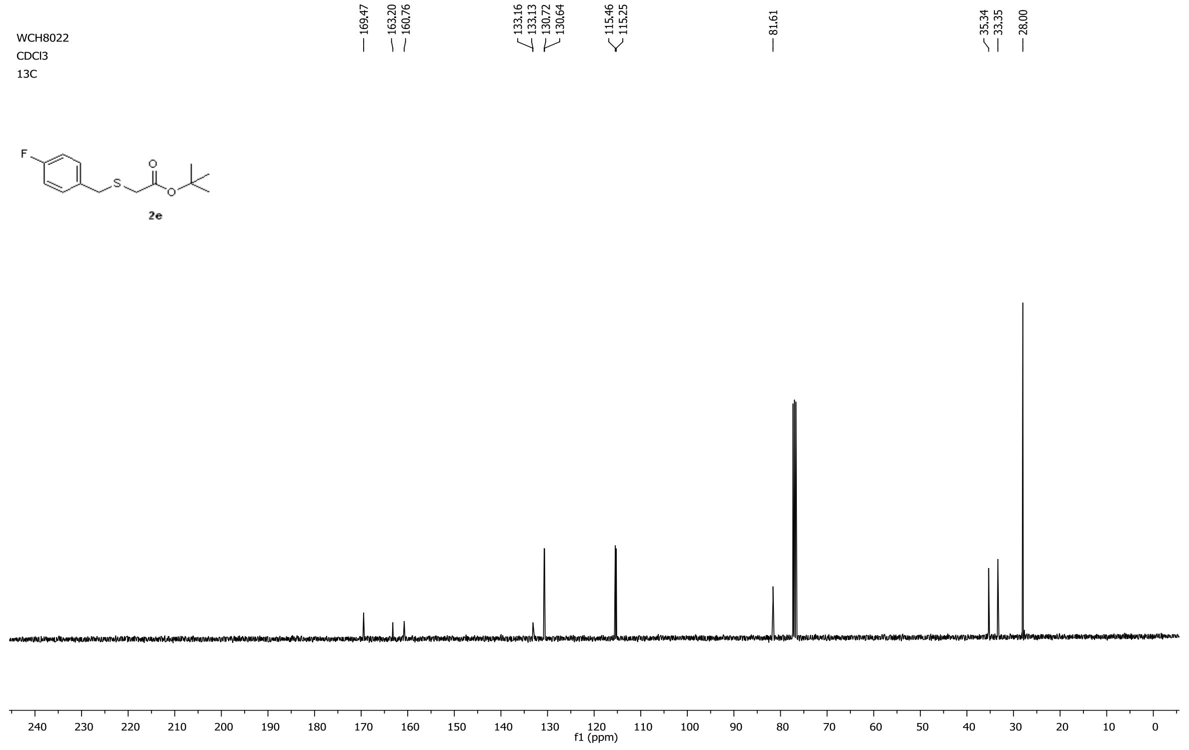


**Supplementary Figure 22.** $^{1}H$ and $^{13}C$ NMR spectra of sulfide **2e**.

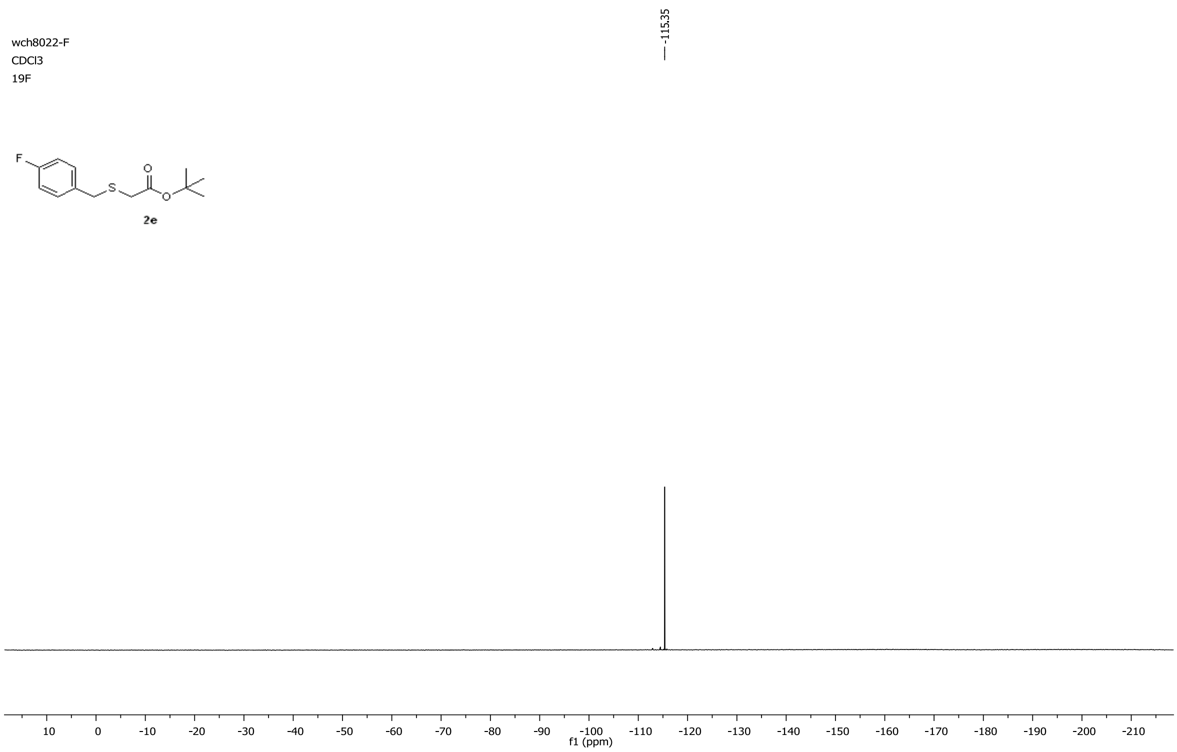


**Supplementary Figure 23.** $^{19}$F NMR spectrum of sulfide **2e**.

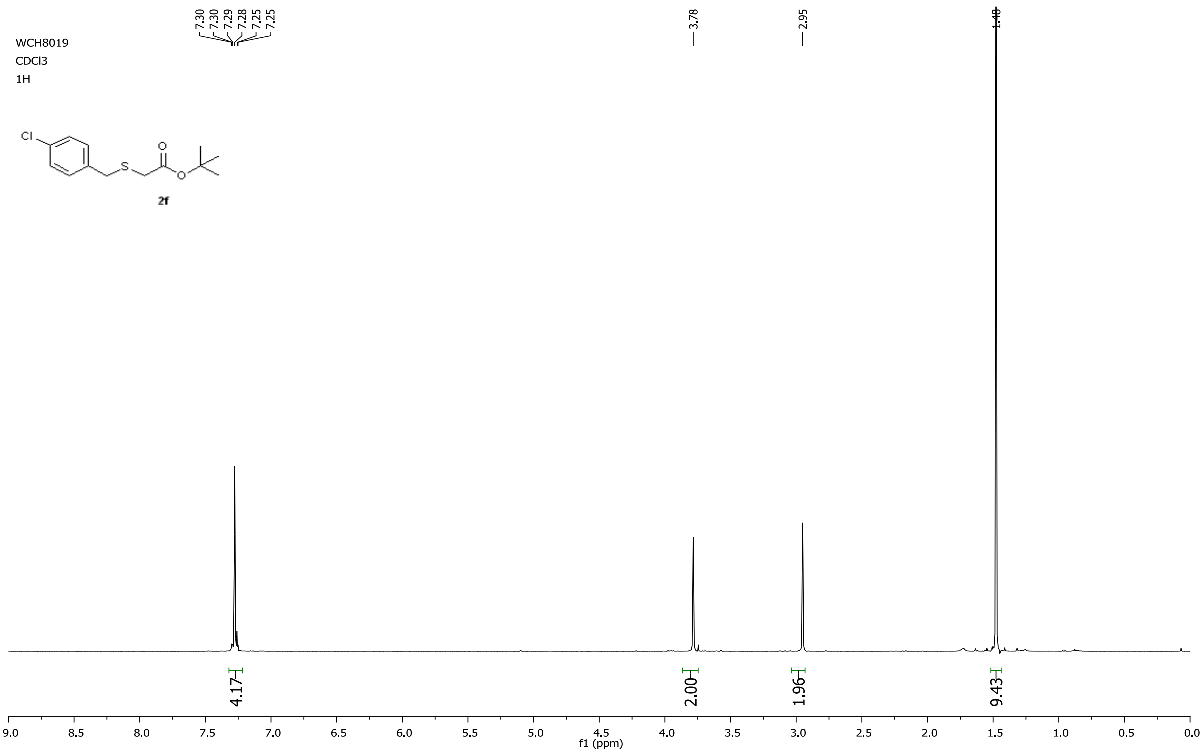


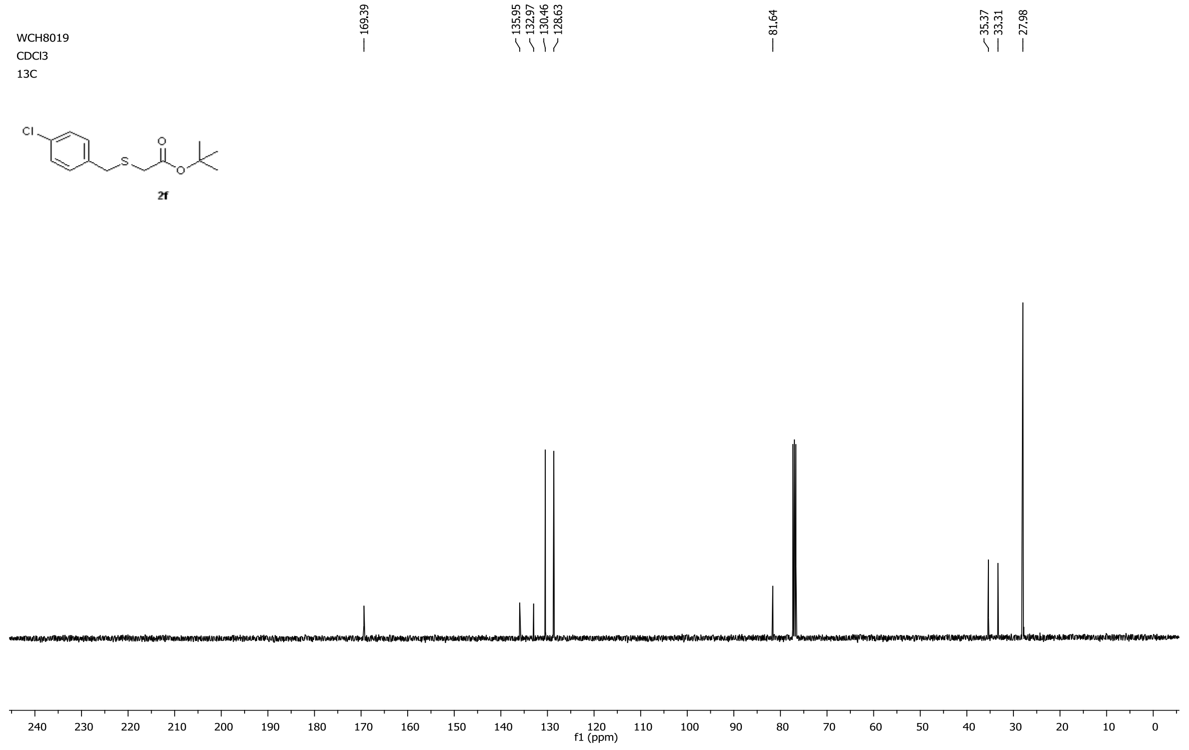


**Supplementary Figure 24.** $^1$H and $^{13}$C NMR spectra of sulfide **2f**.

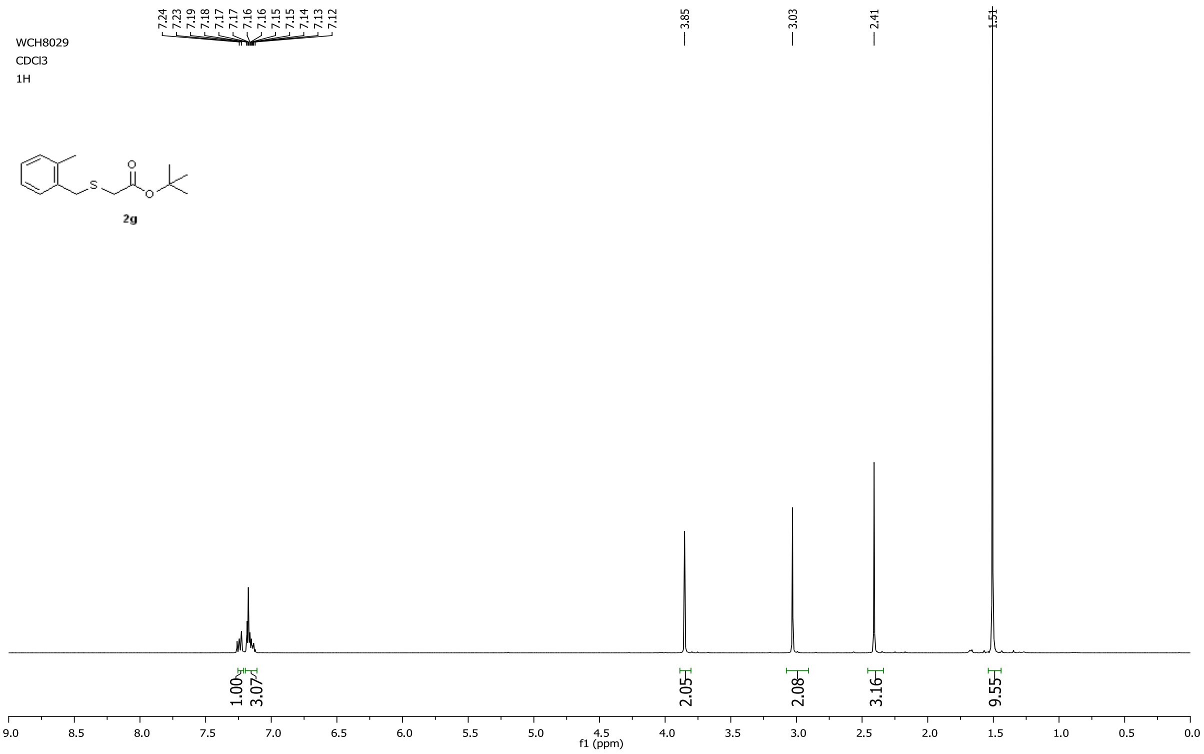


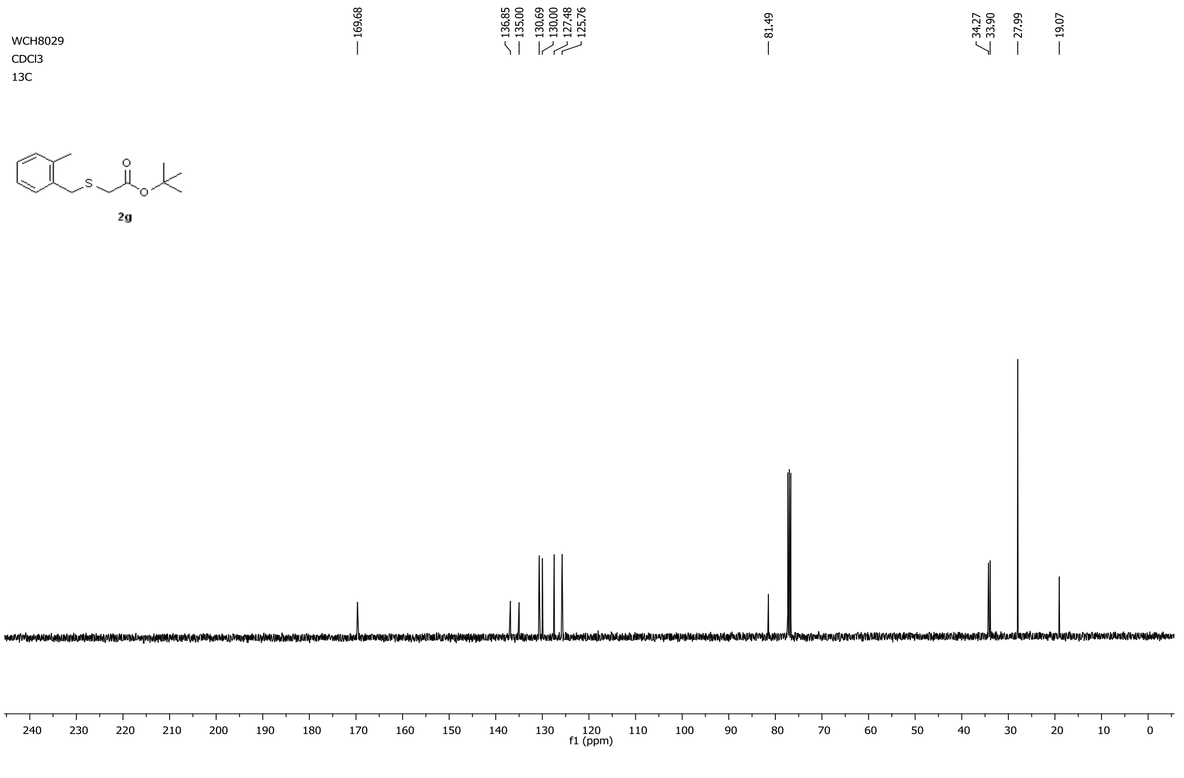


**Supplementary Figure 25.** $^{1}$H and $^{13}$C NMR spectra of sulfide **2g**.

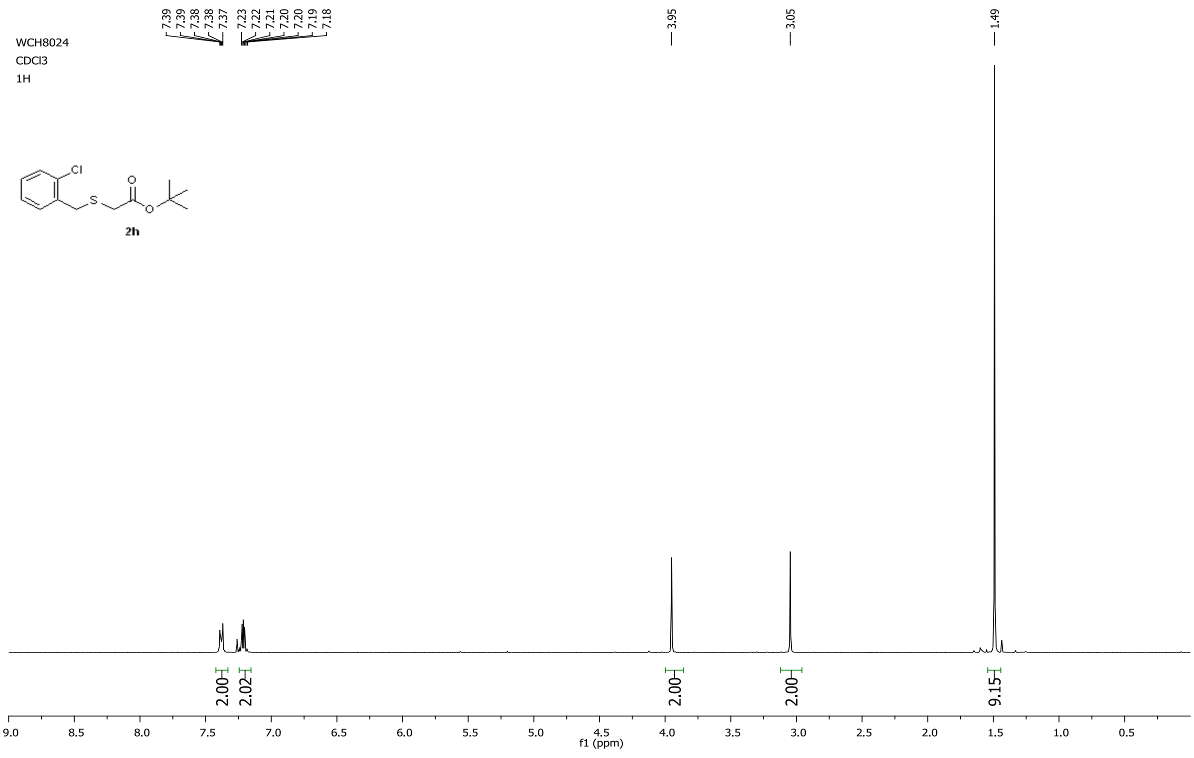


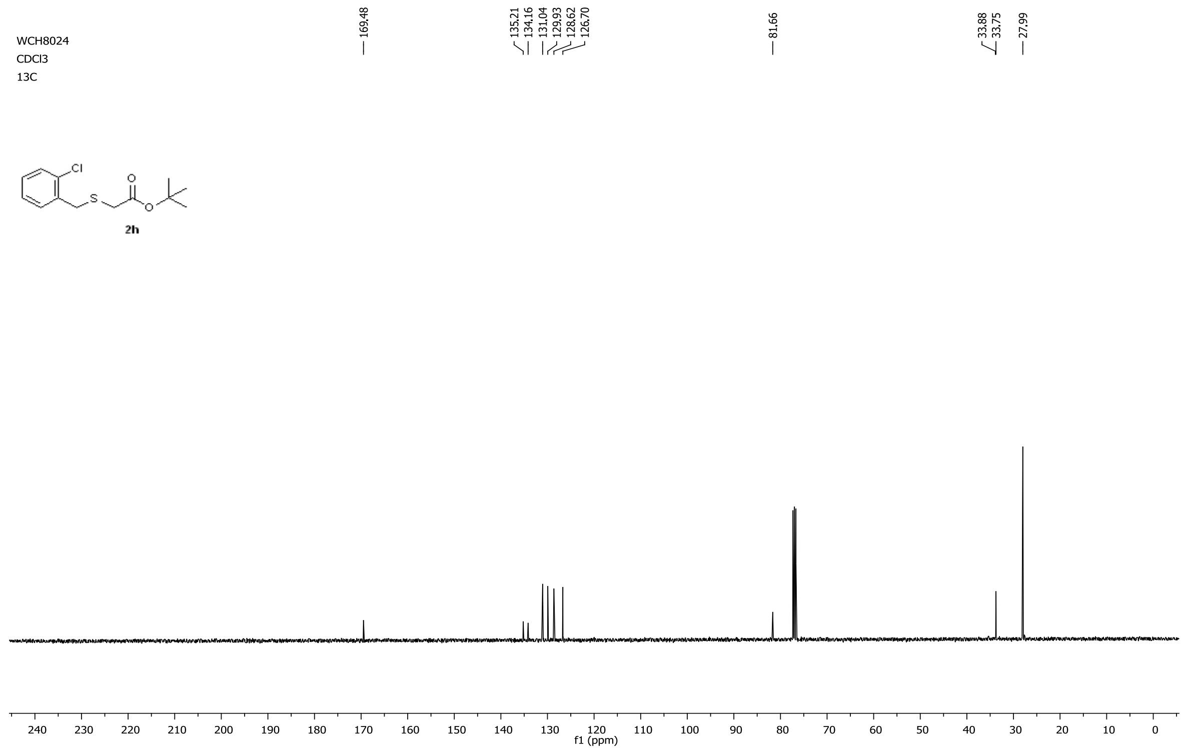


**Supplementary Figure 26.** $^{1}$H and $^{13}$C NMR spectra of sulfide **2h**.

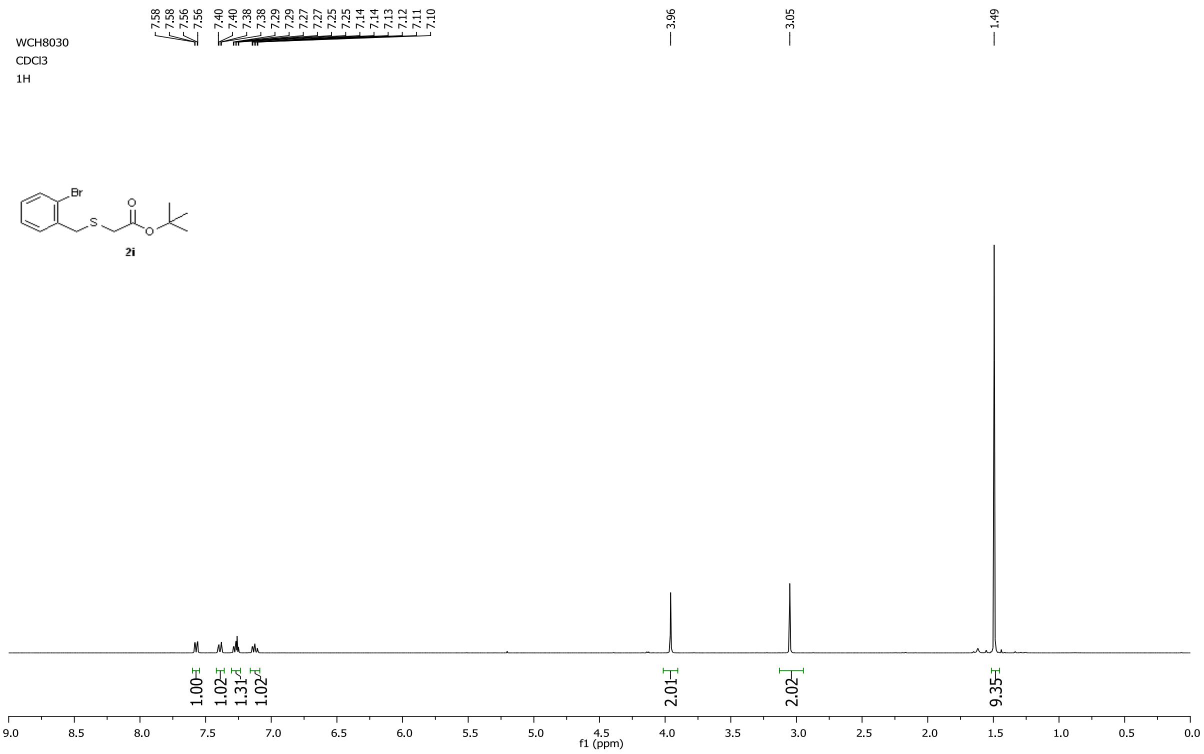


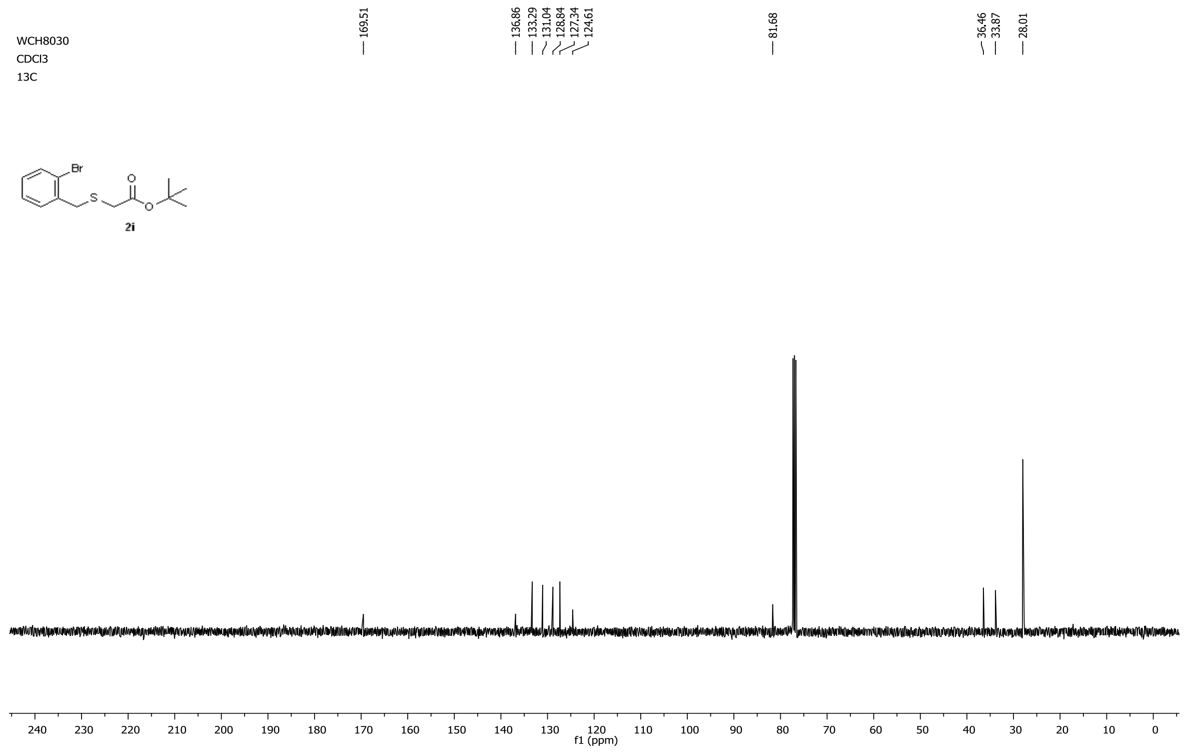


**Supplementary Figure 27.** $^{1}$H and $^{13}$C NMR spectra of sulfide **2i**.

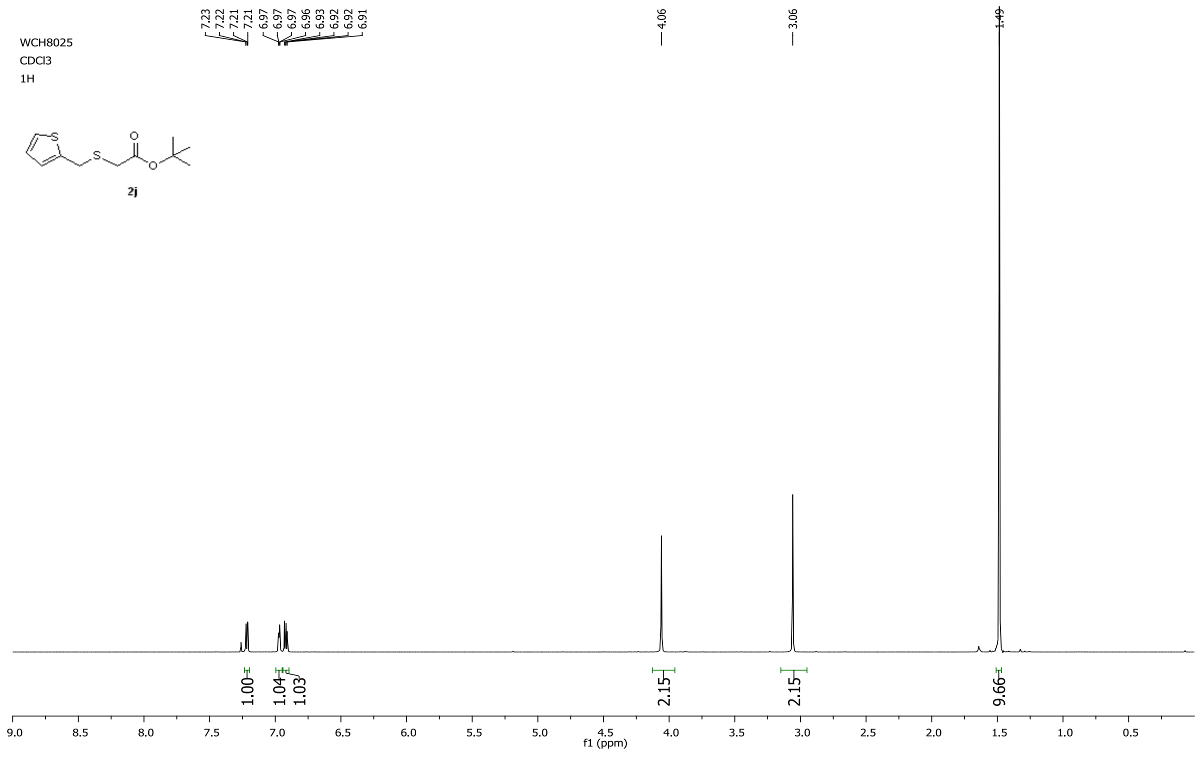


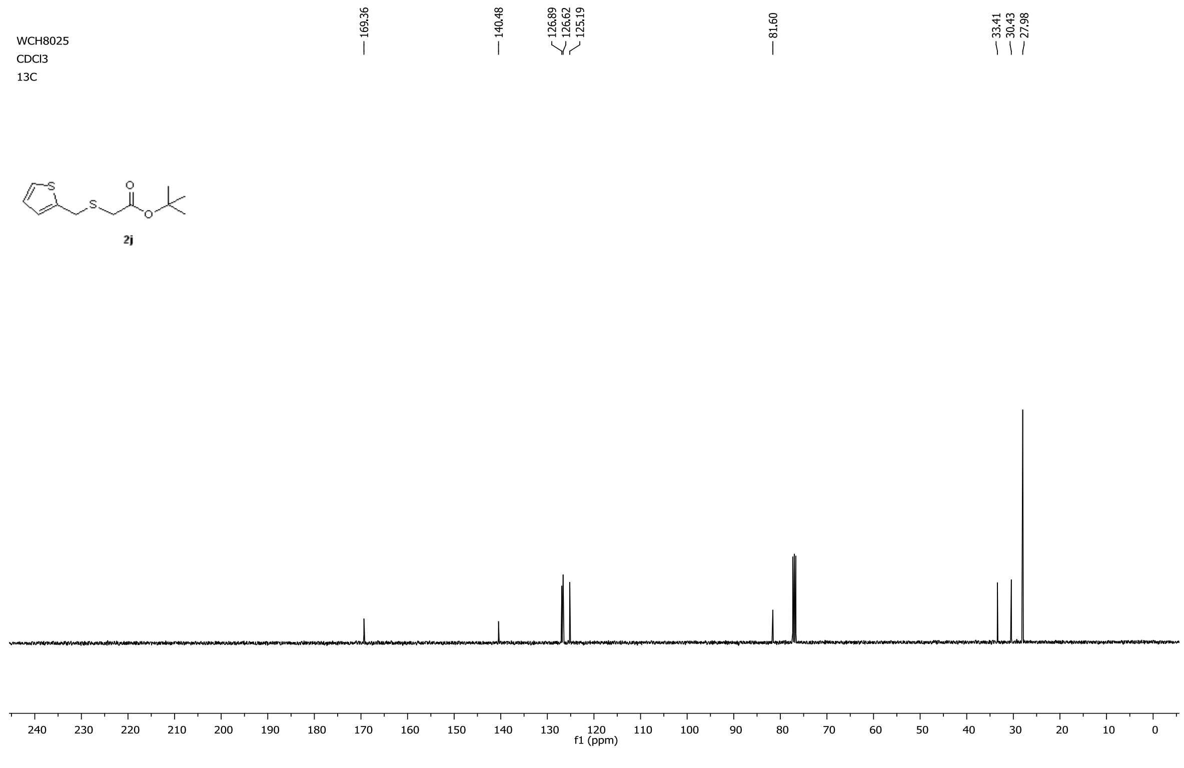


**Supplementary Figure 28.** $^{1}$H and $^{13}$C NMR spectra of sulfide **2j**.

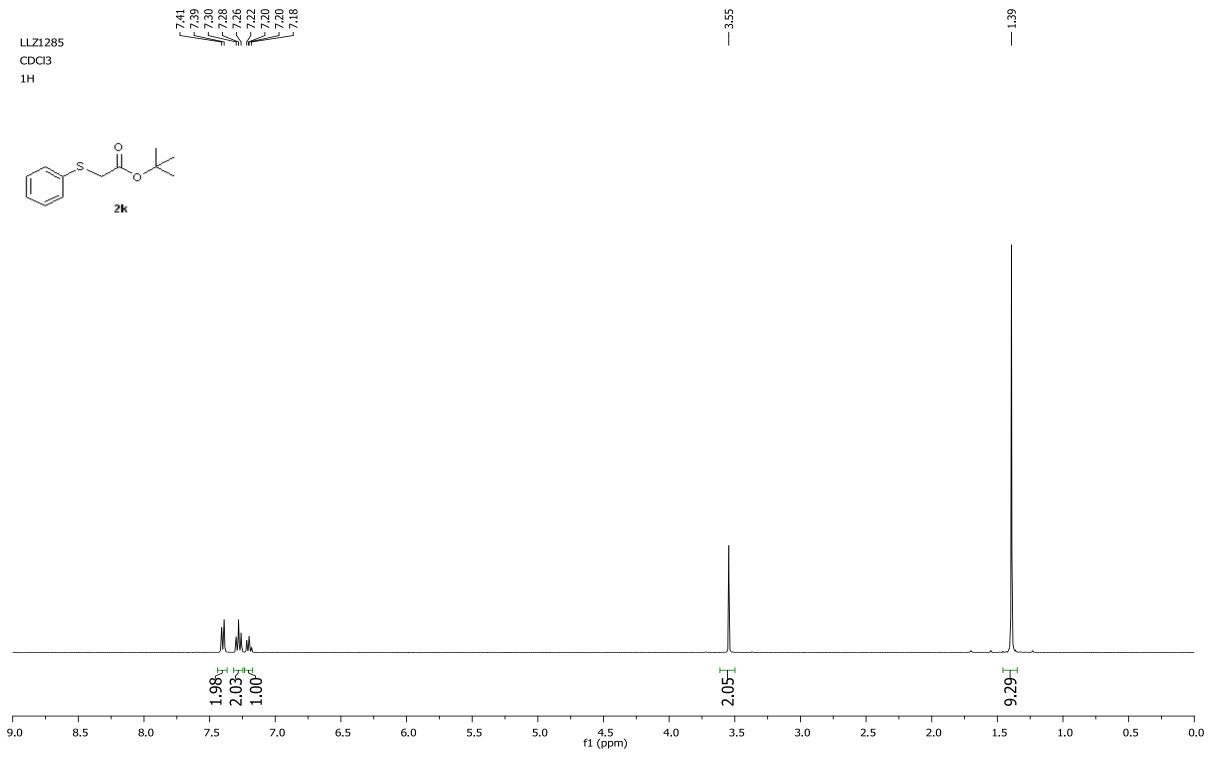


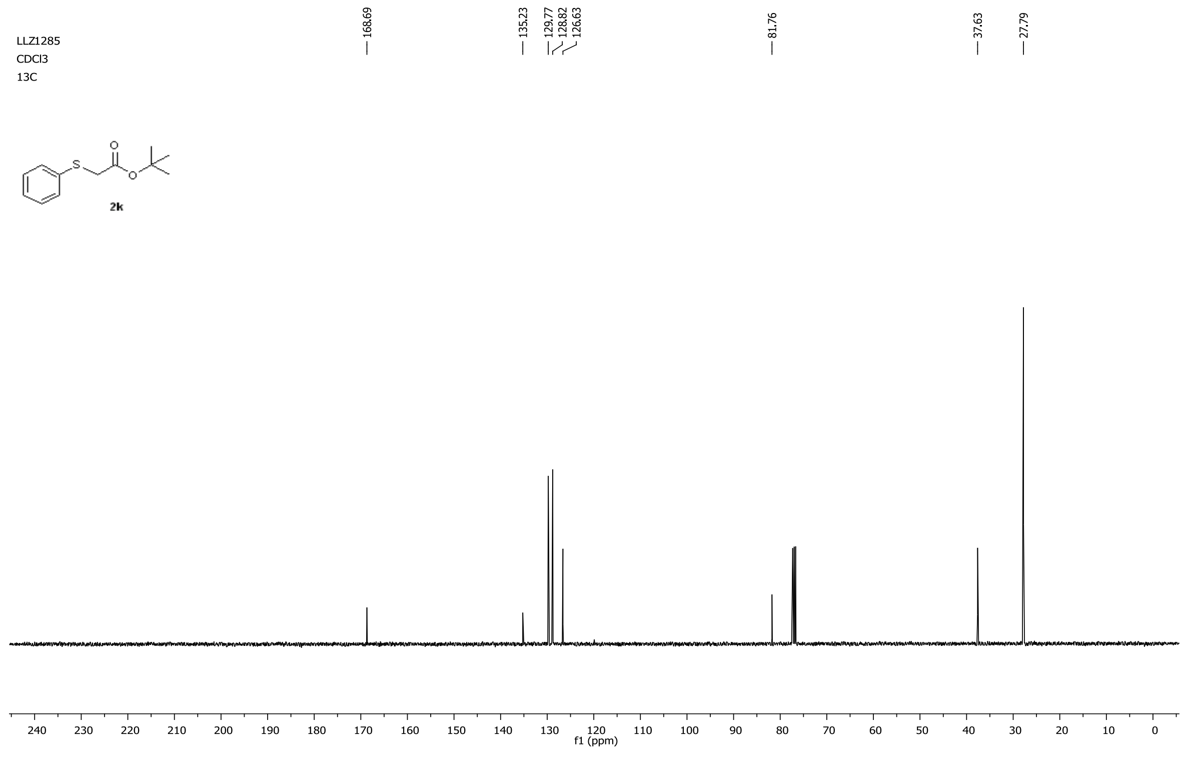


**Supplementary Figure 29.** $^{1}$H and $^{13}$C NMR spectra of sulfide **2k**.

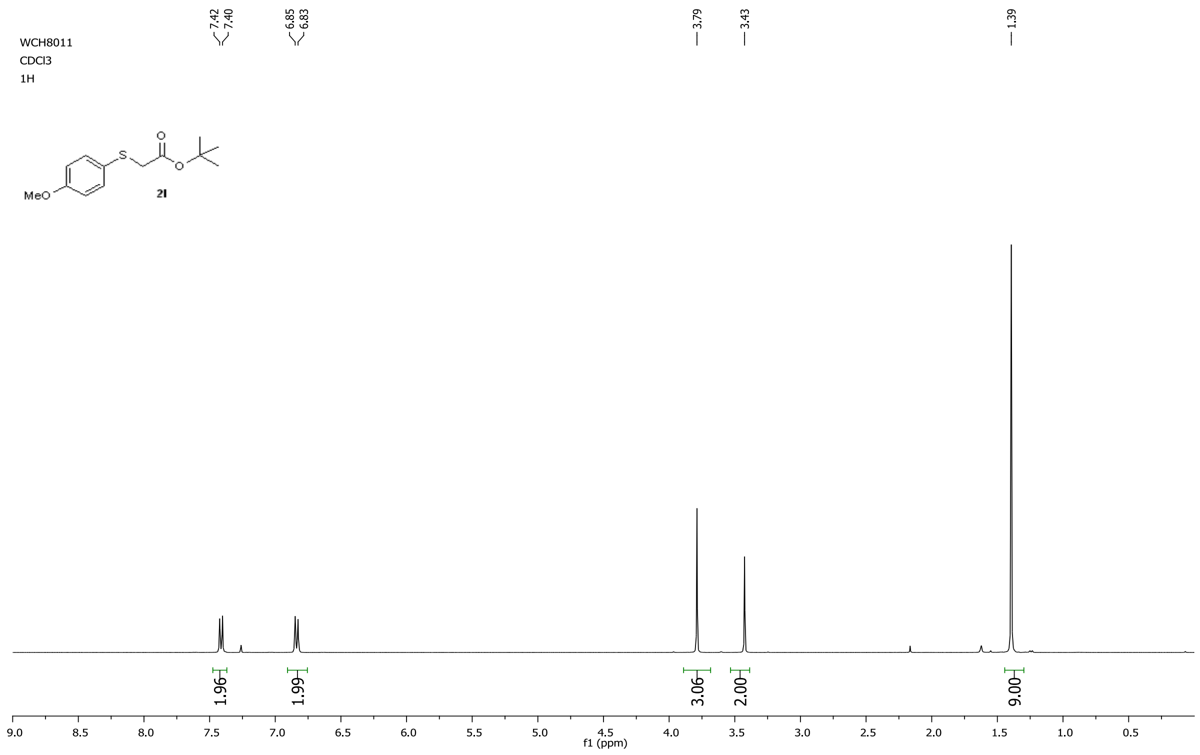


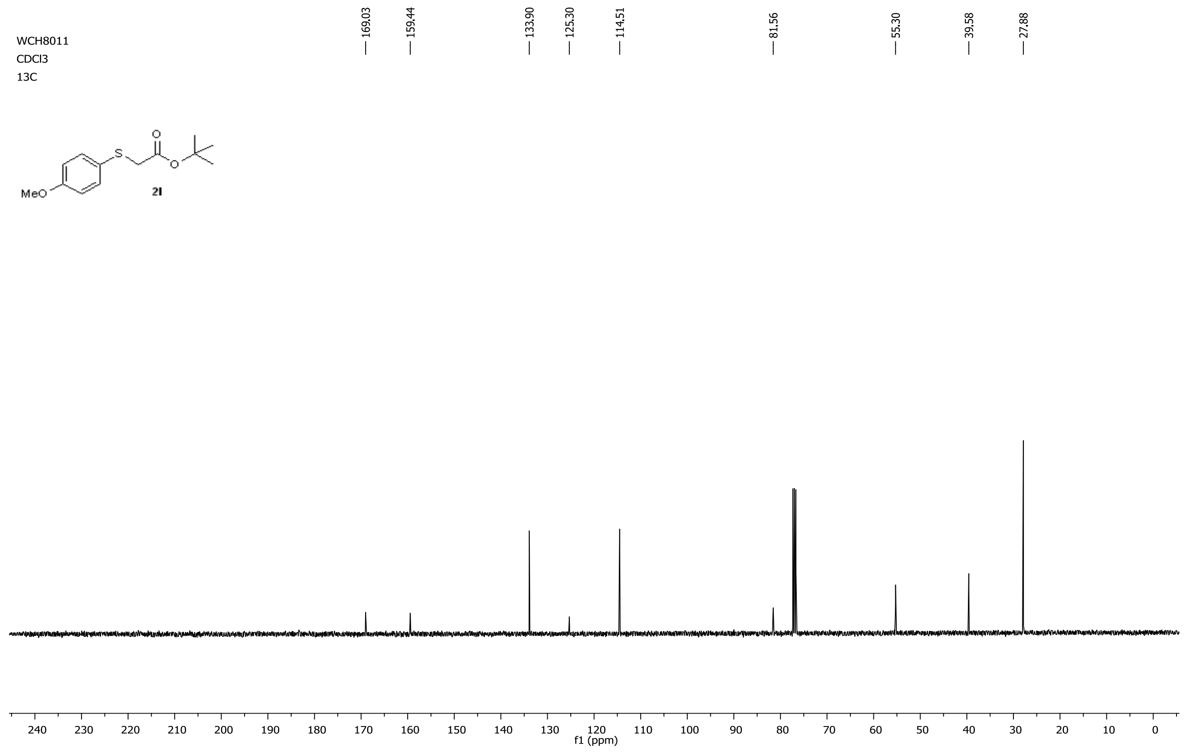


**Supplementary Figure 30.** $^{1}$H and $^{13}$C NMR spectra of sulfide **2l**.

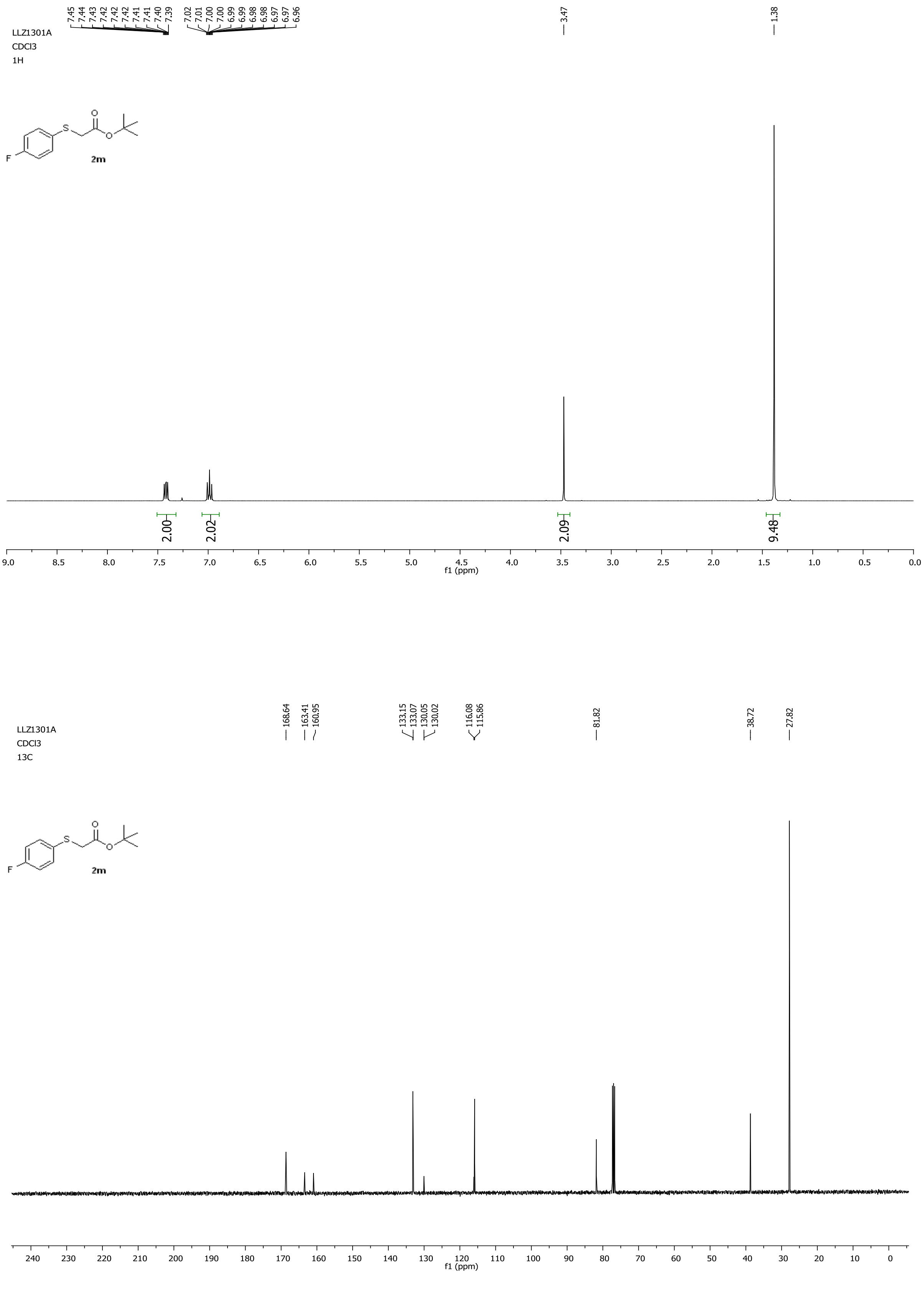


**Supplementary Figure 31.** $^{1}H$ and $^{13}C$ NMR spectra of sulfide **2m**.

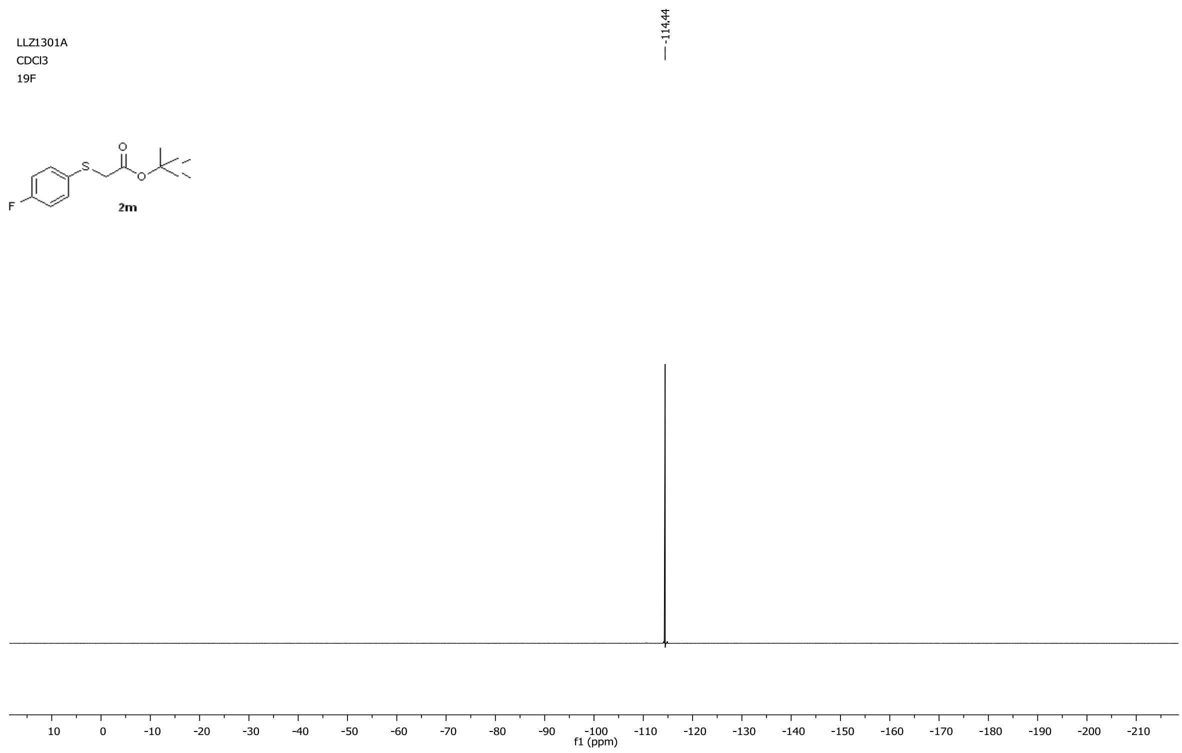


**Supplementary Figure 32.** $^{19}$F NMR spectrum of sulfide **2m**.

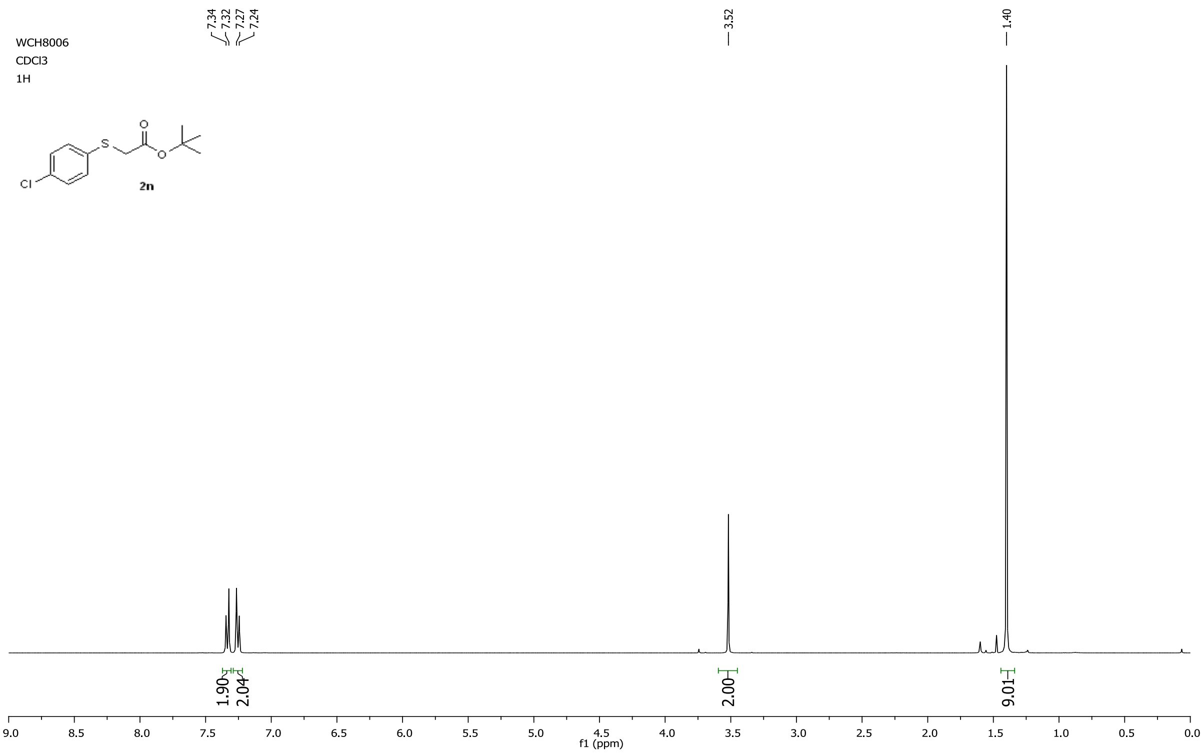


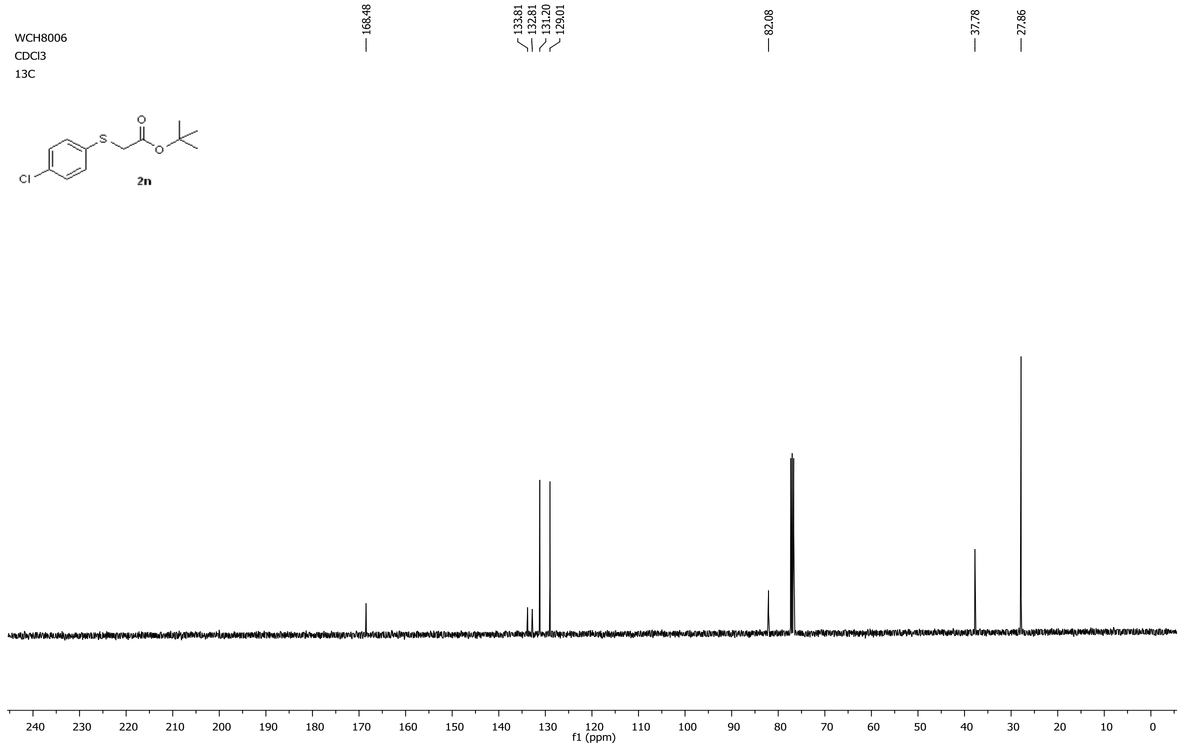


**Supplementary Figure 33.** $^{1}$H and $^{13}$C NMR spectra of sulfide **2n**.

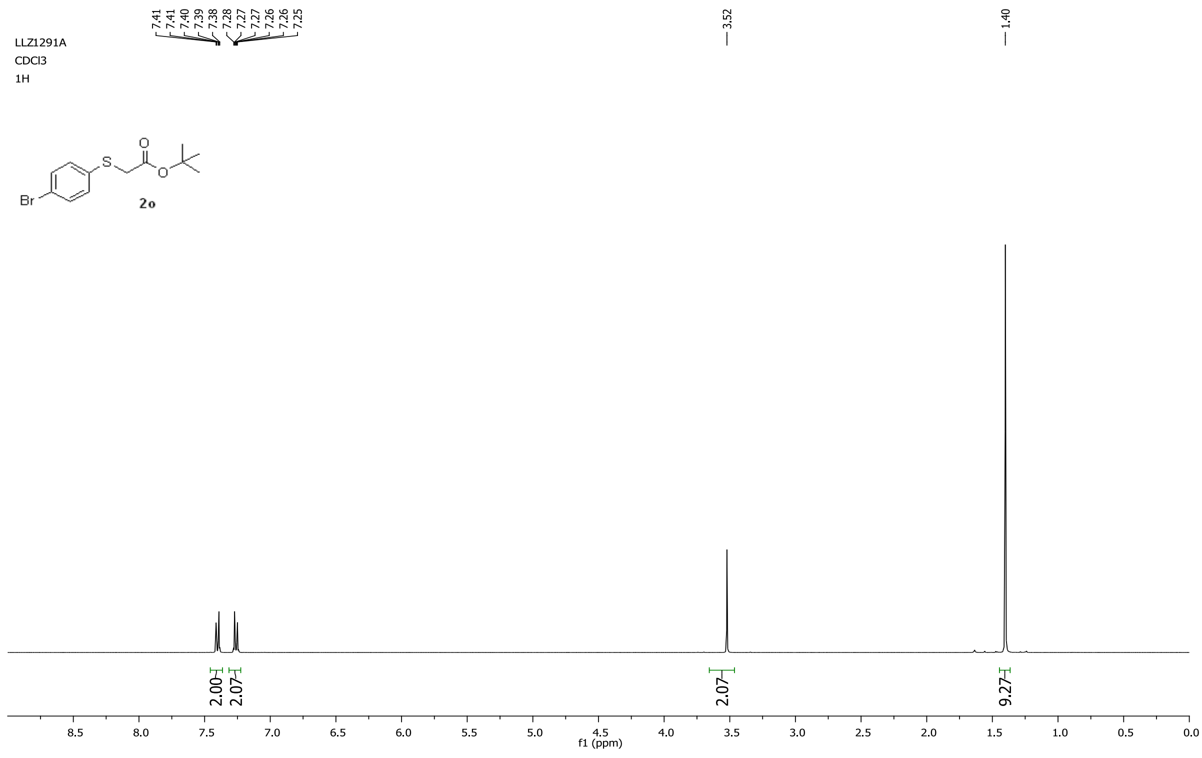


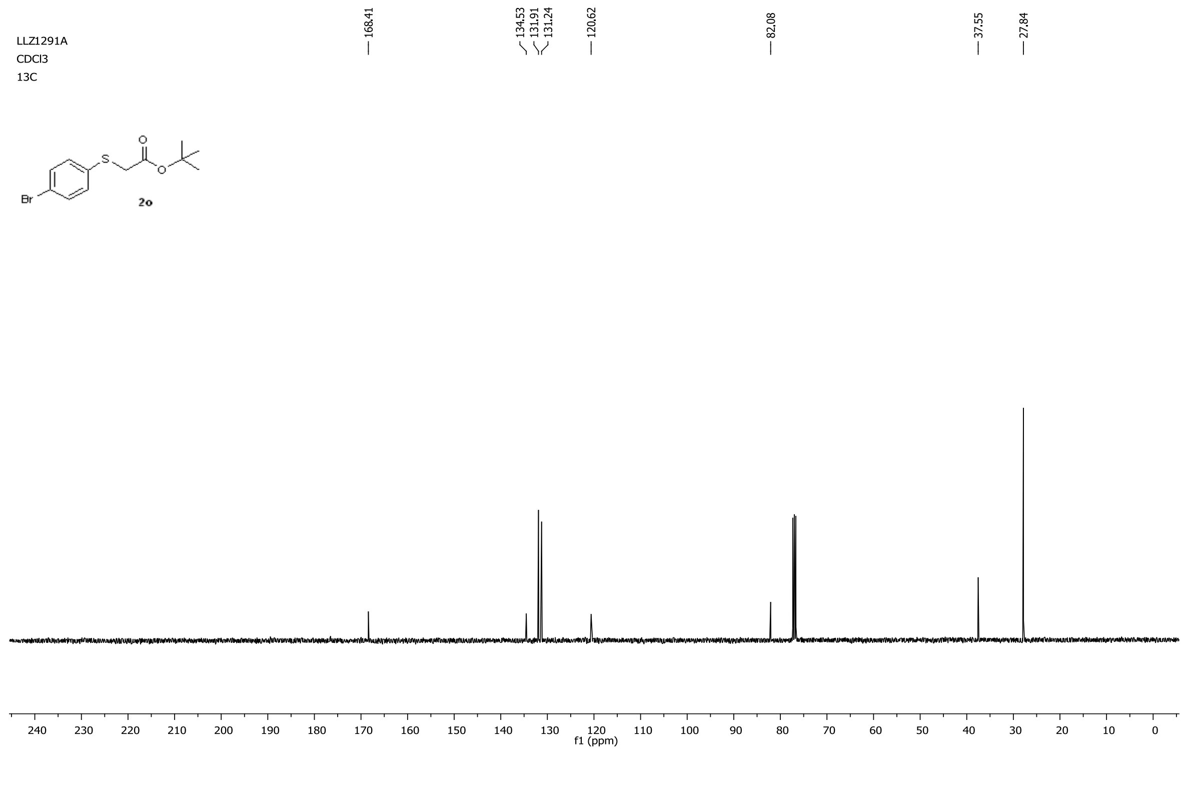


**Supplementary Figure 34.** $^{1}H$ and $^{13}C$ NMR spectra of sulfide **2o**.

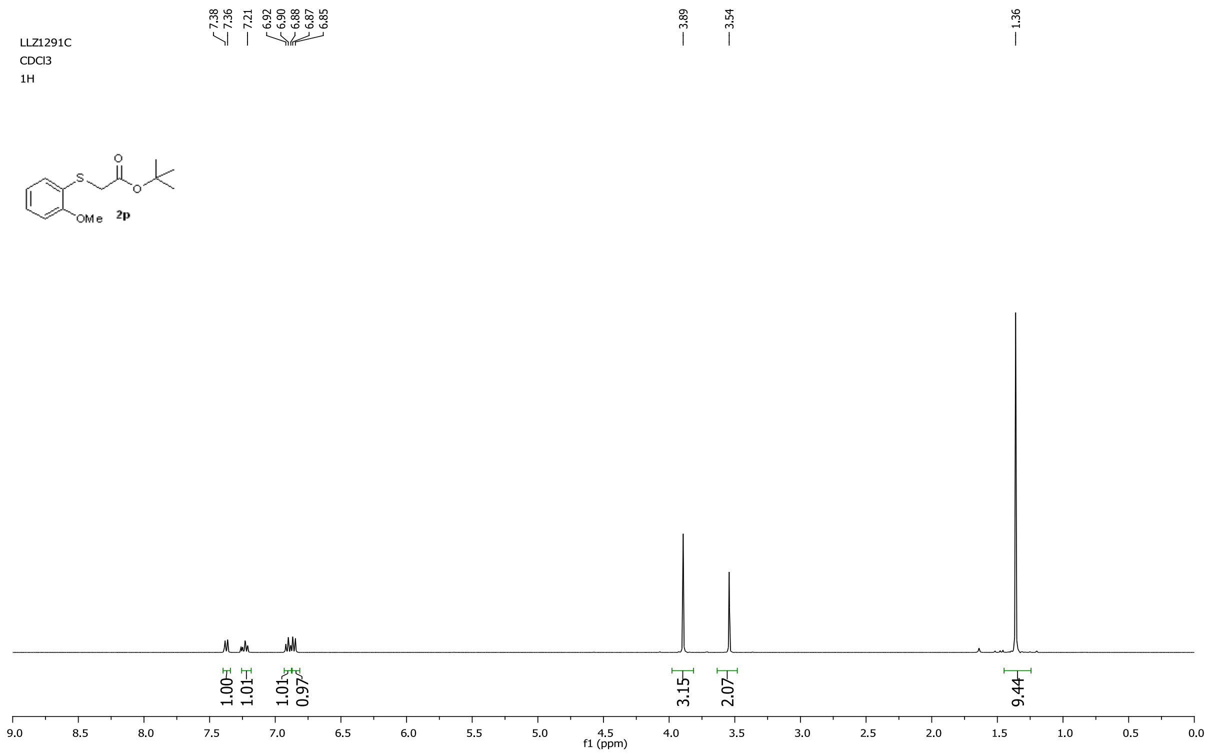


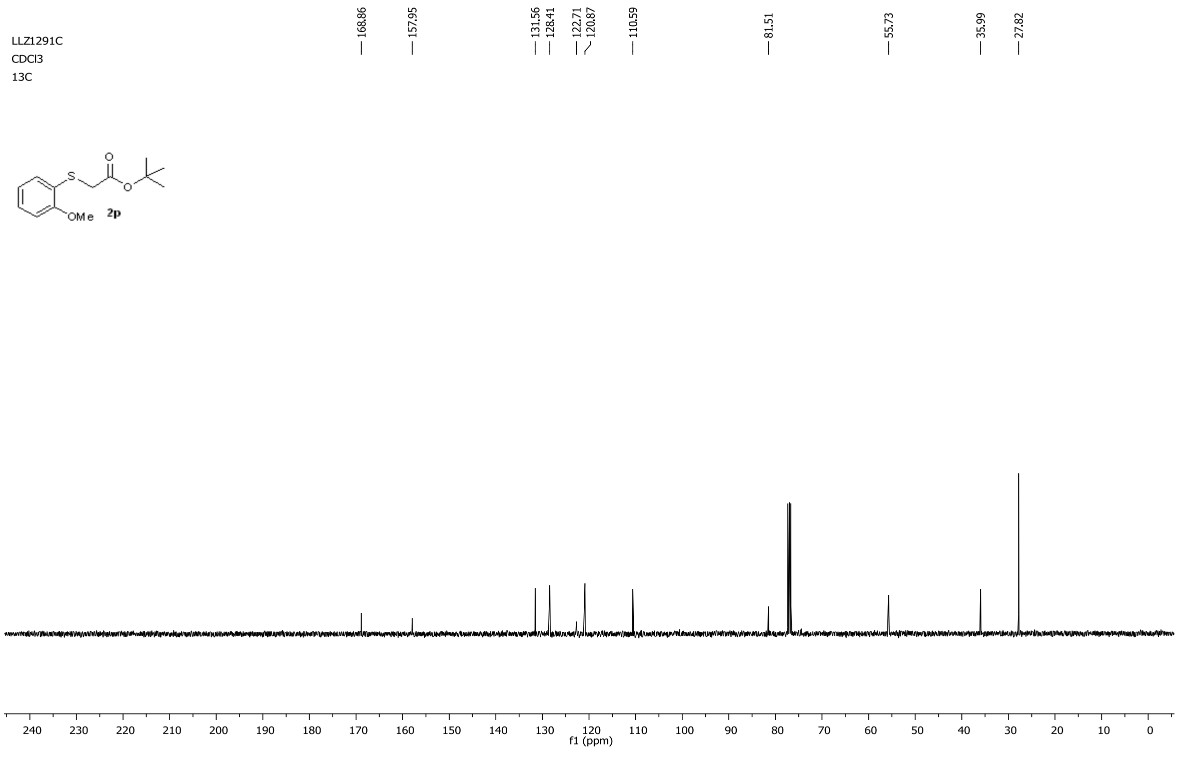


**Supplementary Figure 35.** $^{1}$H and $^{13}$C NMR spectra of sulfide **2p**.

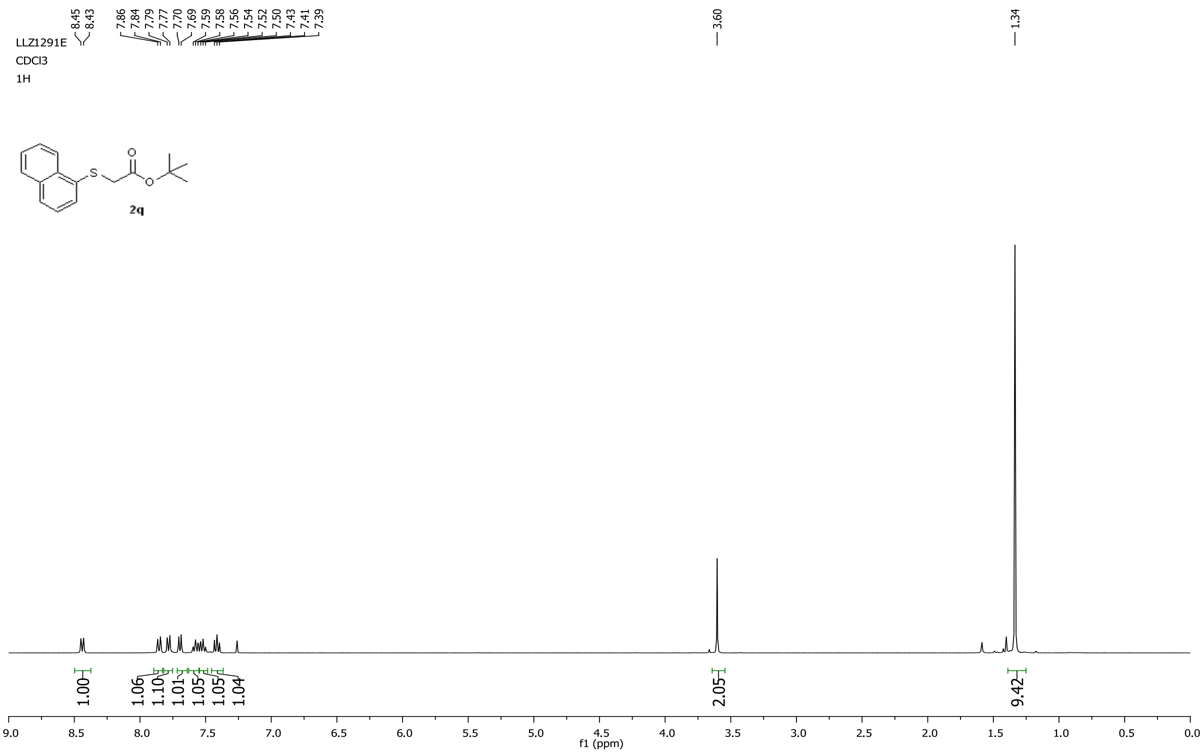


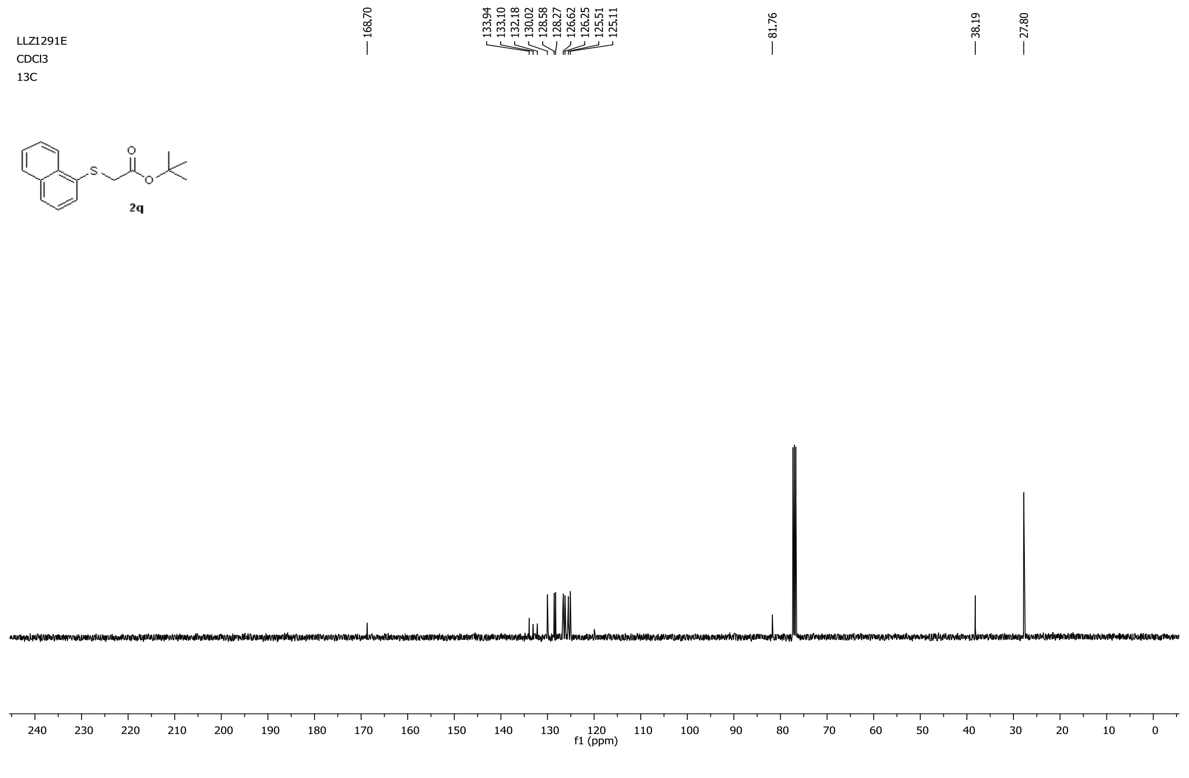


**Supplementary Figure 36.** $^{1}$H and $^{13}$C NMR spectra of sulfide **2q**.

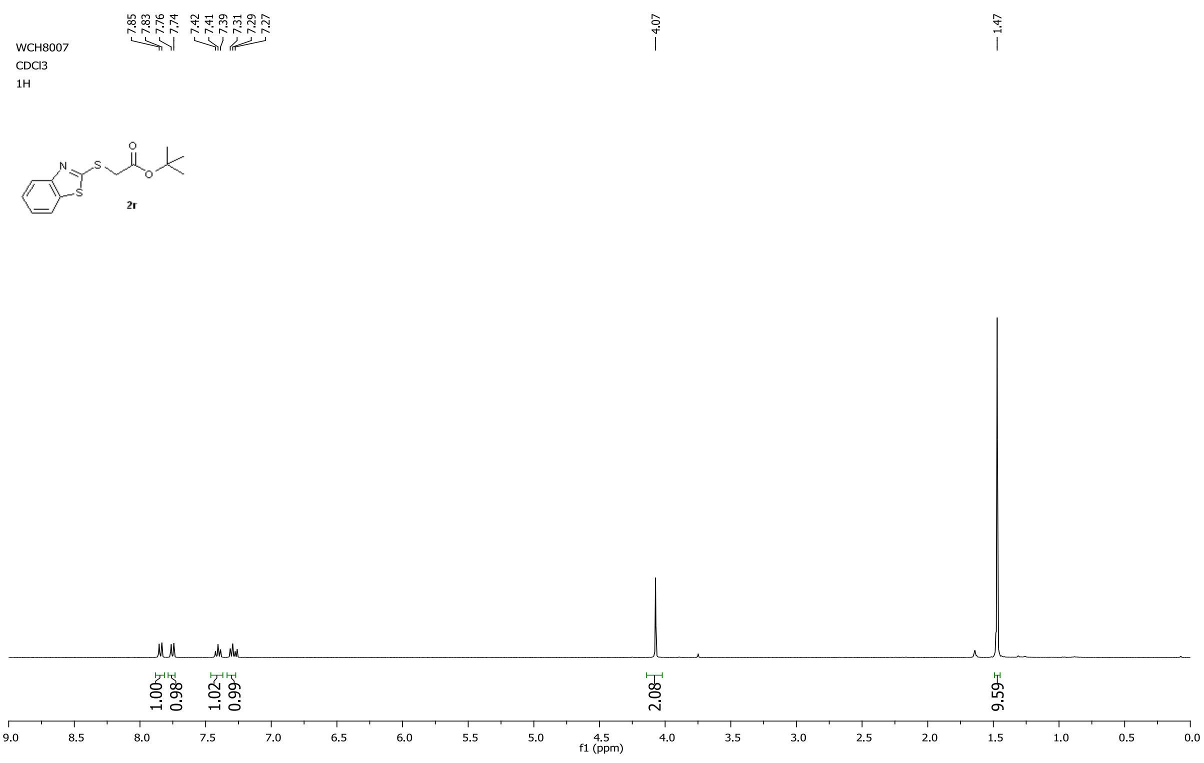

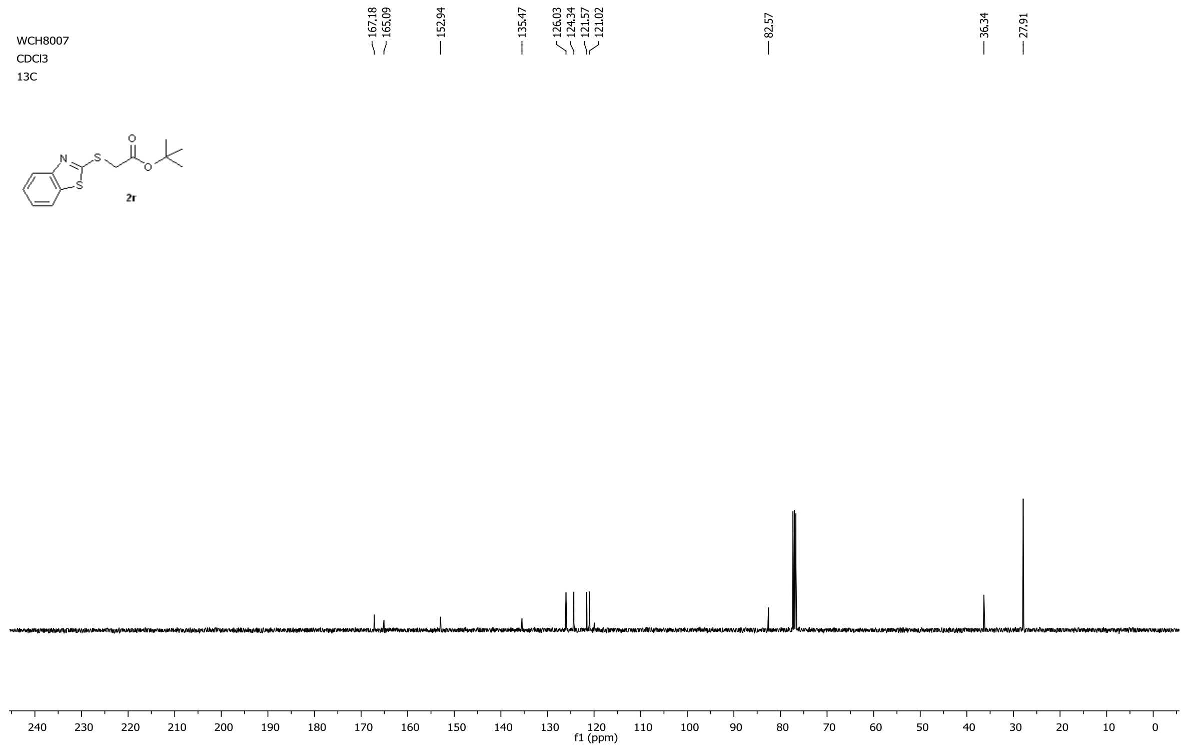

**Supplementary Figure 37.** $^{1}H$ and $^{13}C$ NMR spectra of sulfide **2r**.

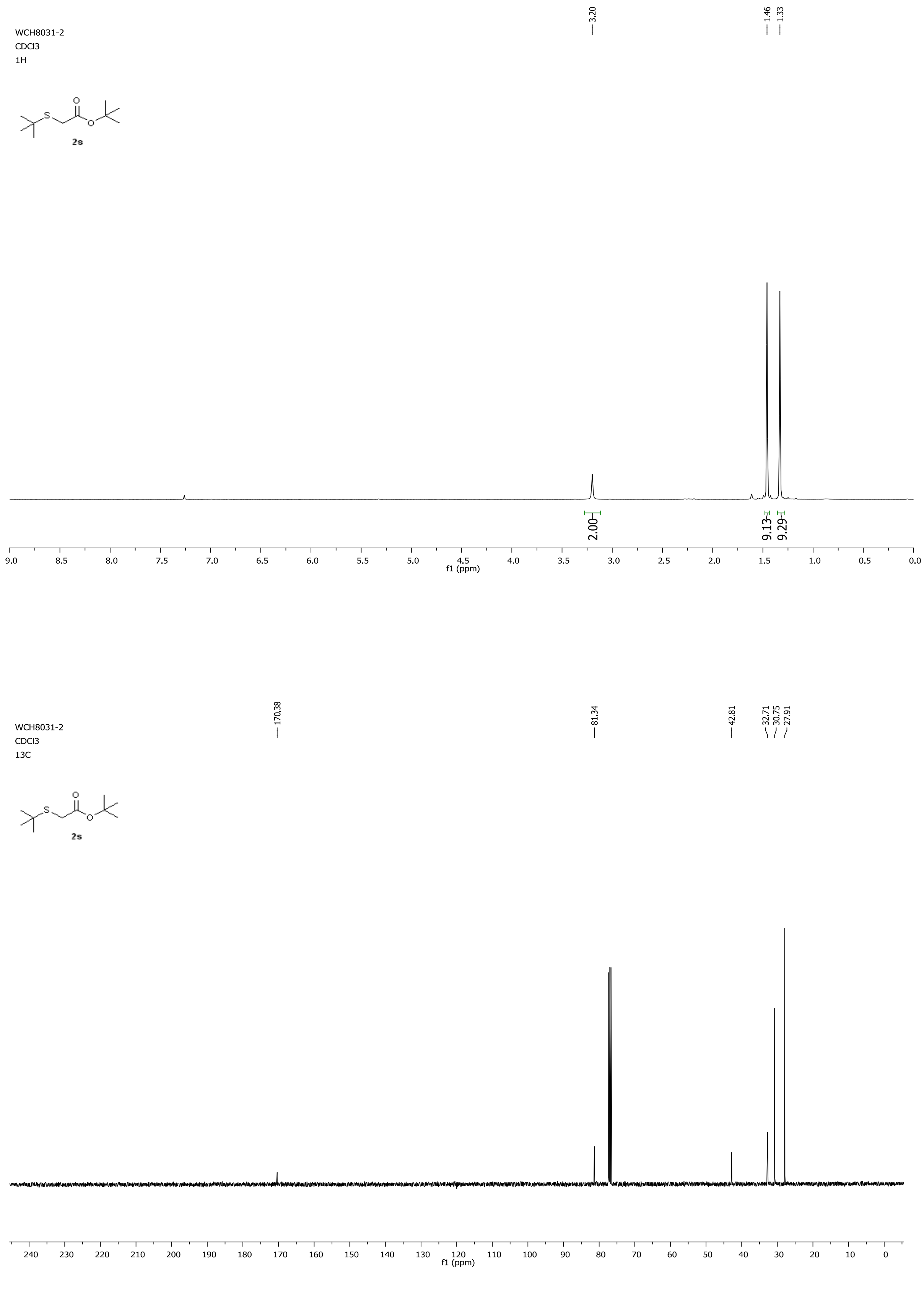


**Supplementary Figure 38.** $^{1}$H and $^{13}$C NMR spectra of sulfide **2s**.

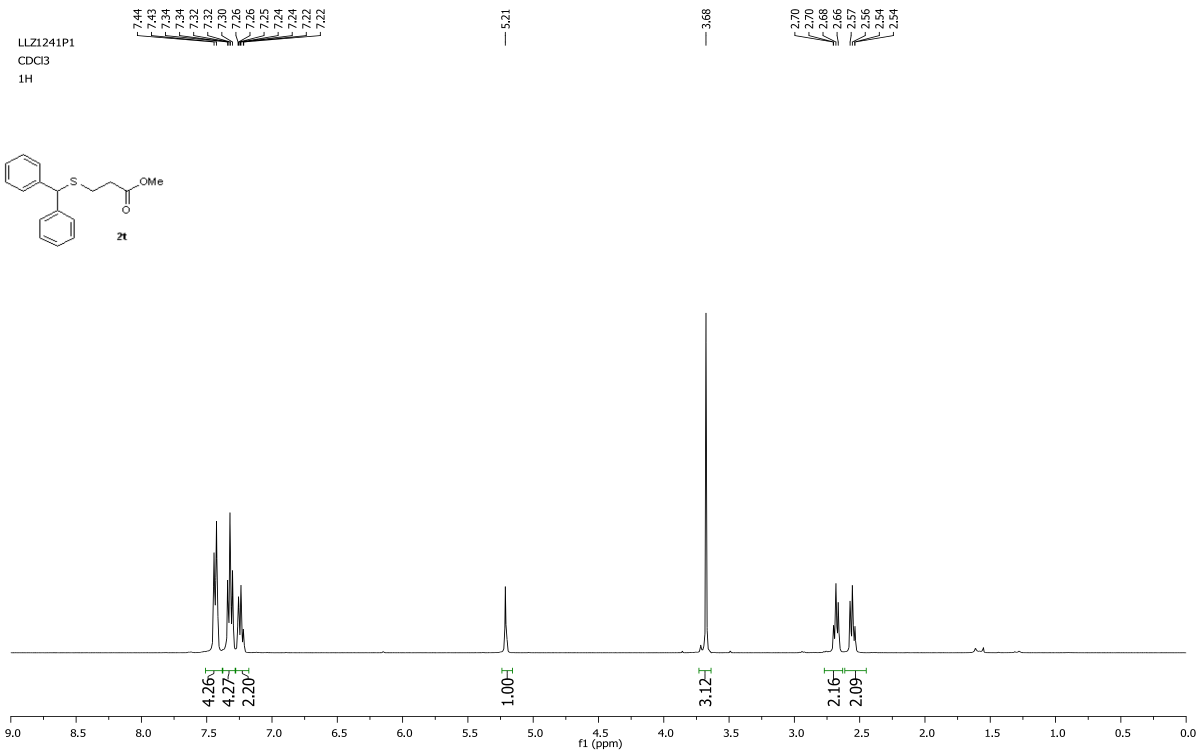


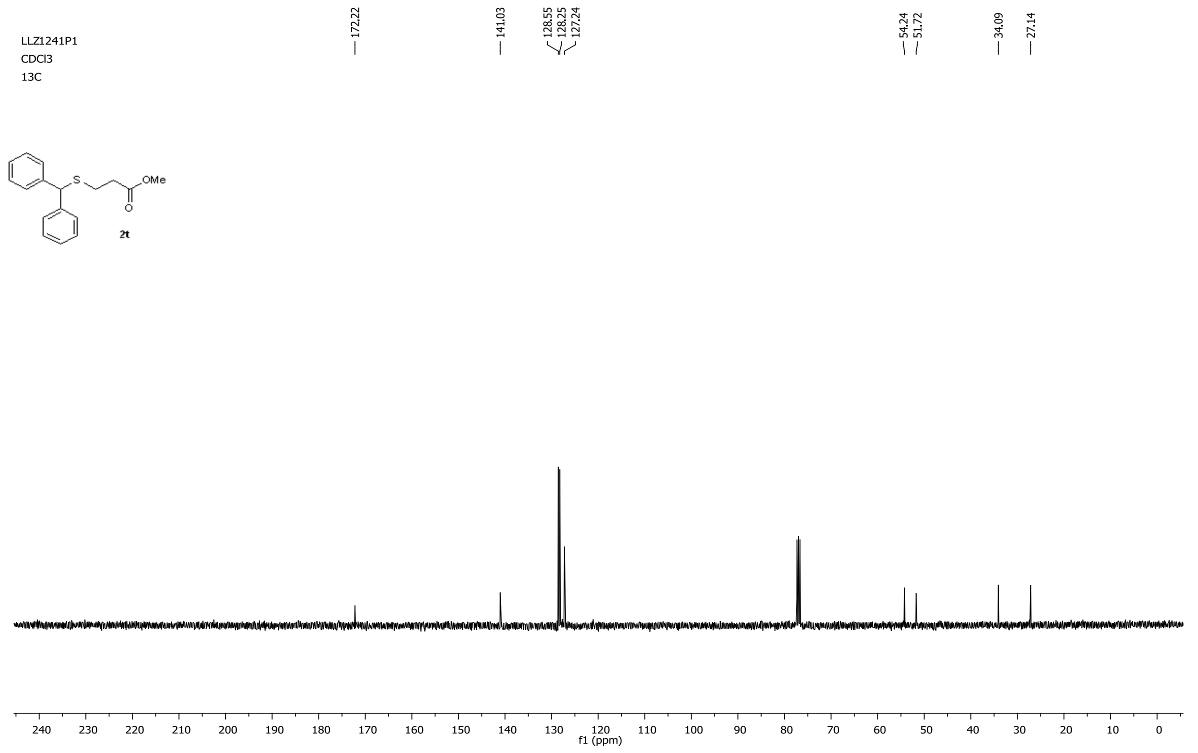


**Supplementary Figure 39.** $^{1}$H and $^{13}$C NMR spectra of sulfide **2t**.

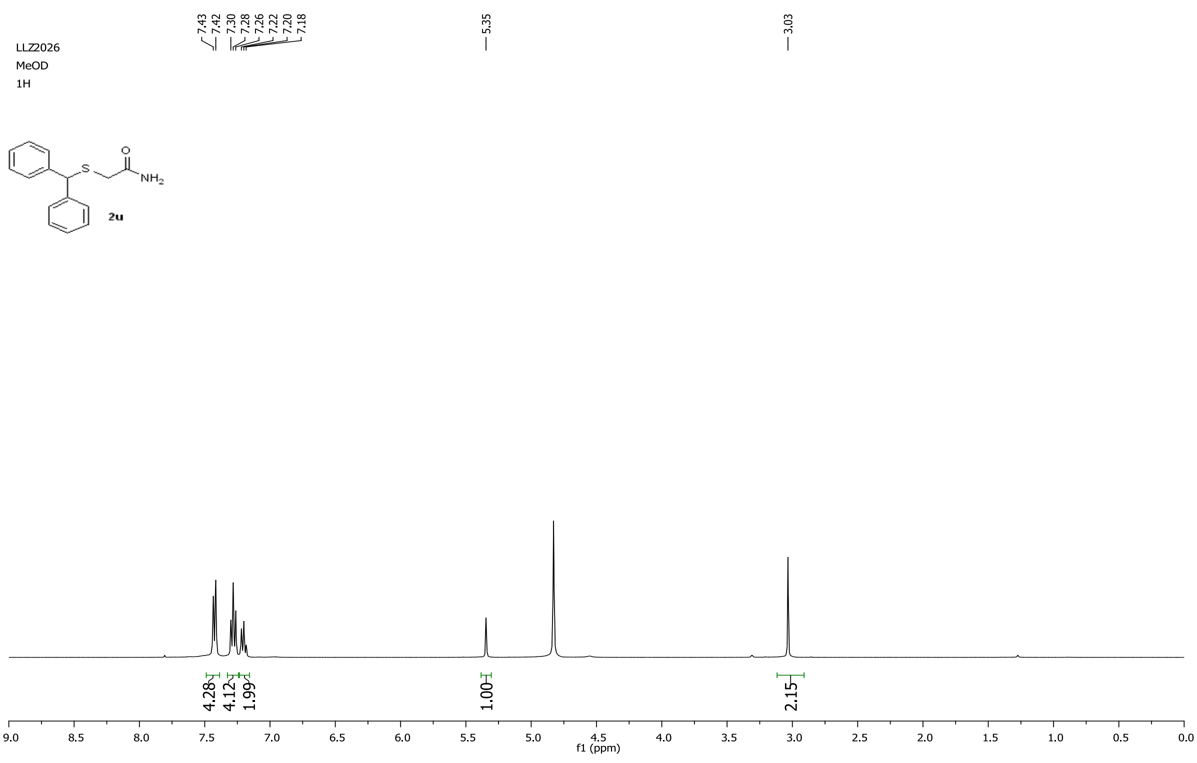


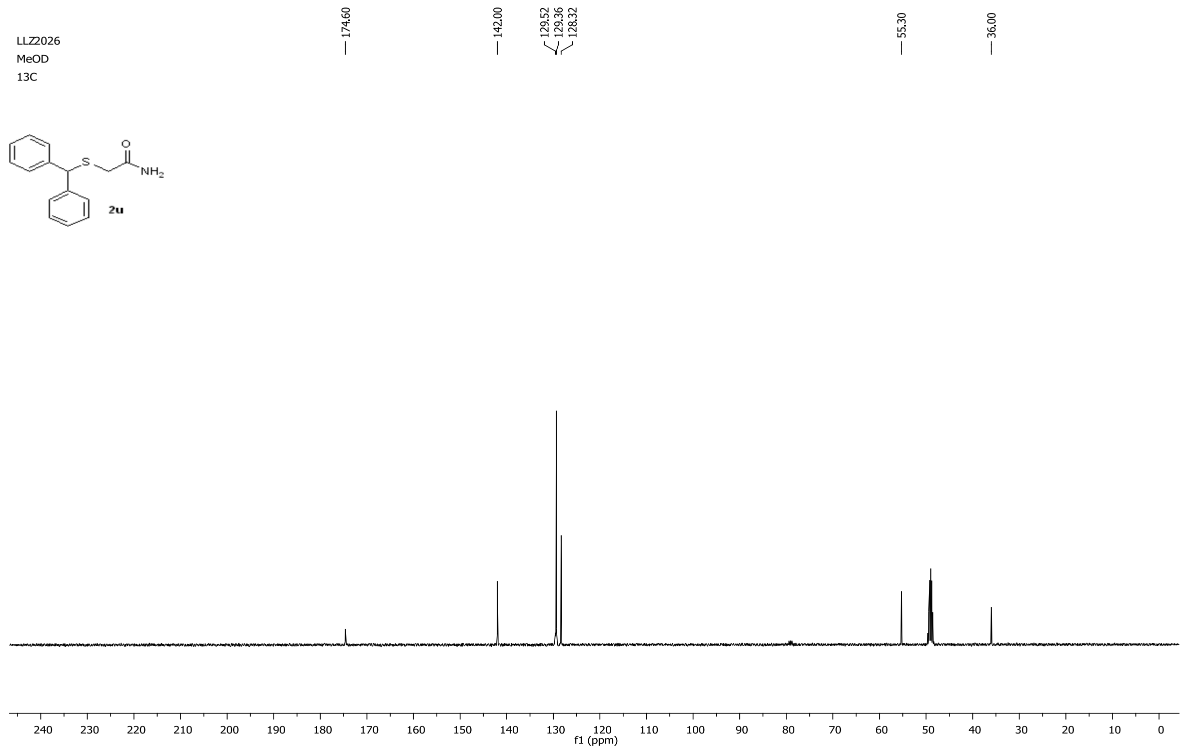


**Supplementary Figure 40.** $^{1}H$ and $^{13}C$ NMR spectra of sulfide **2u**.

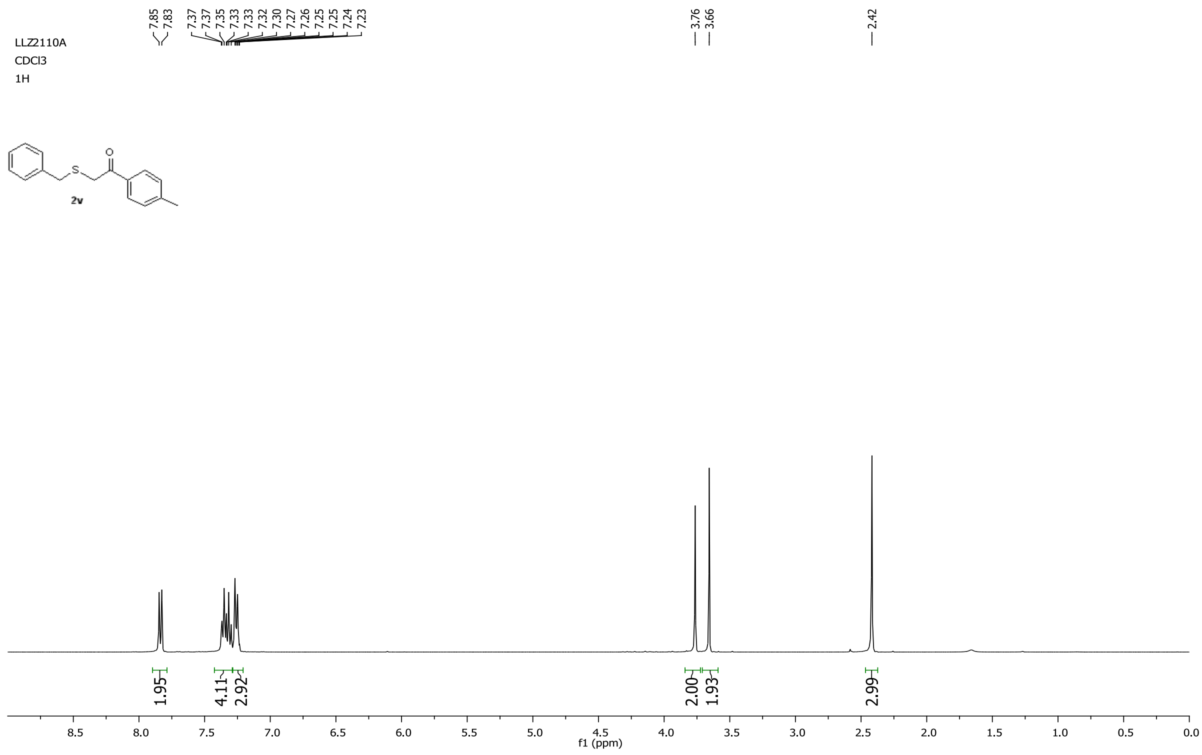


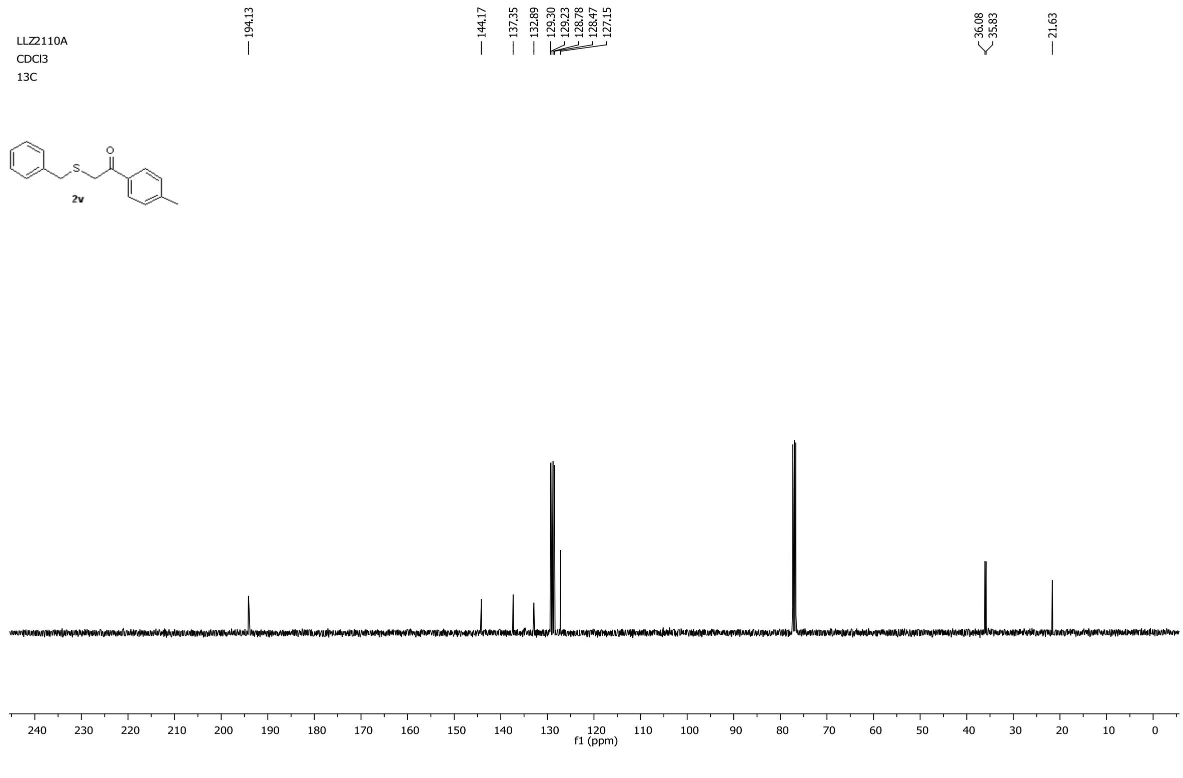


**Supplementary Figure 41.** $^{1}$H and $^{13}$C NMR spectra of sulfide **2v**.

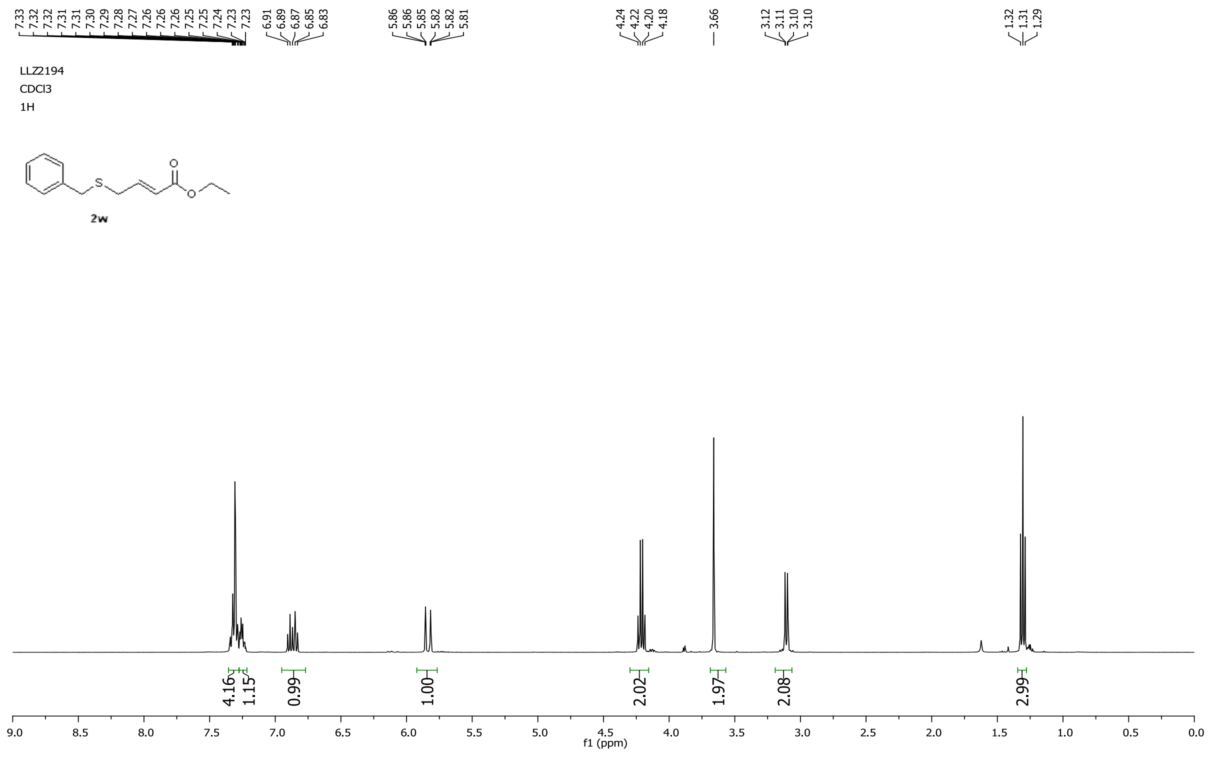


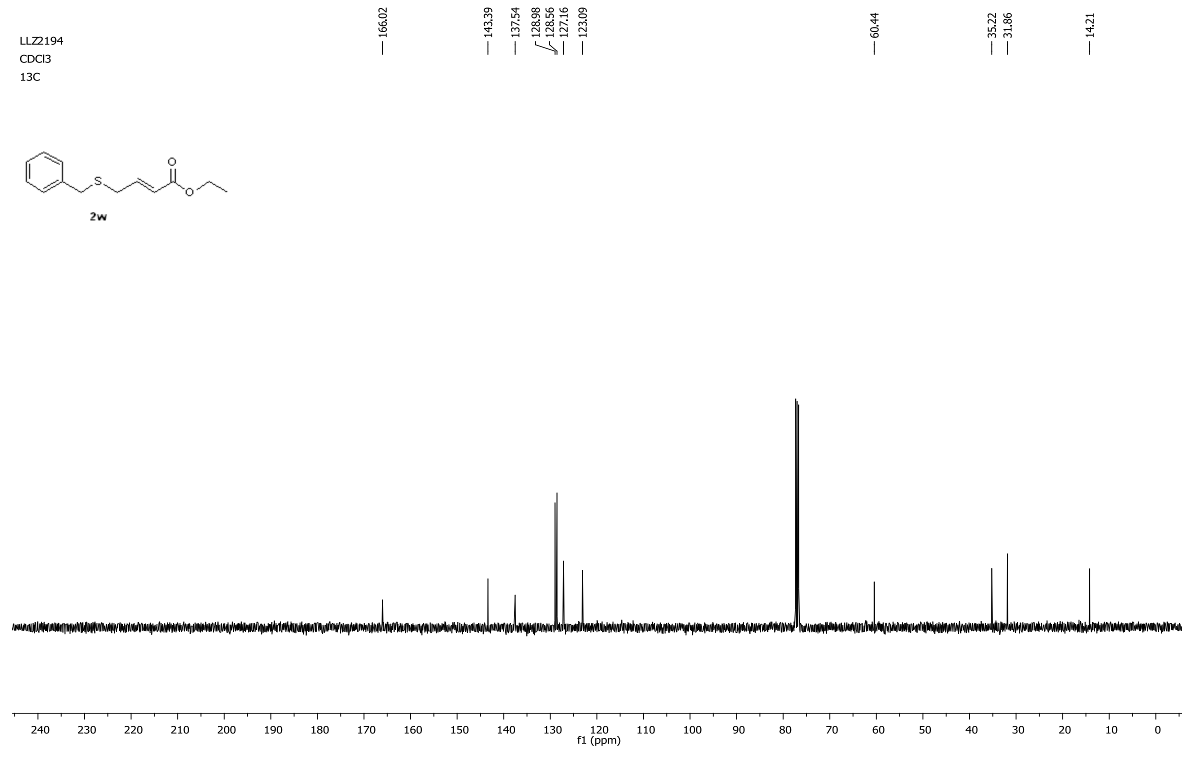


**Supplementary Figure 42.** $^{1}H$ and $^{13}C$ NMR spectra of sulfide **2w**.

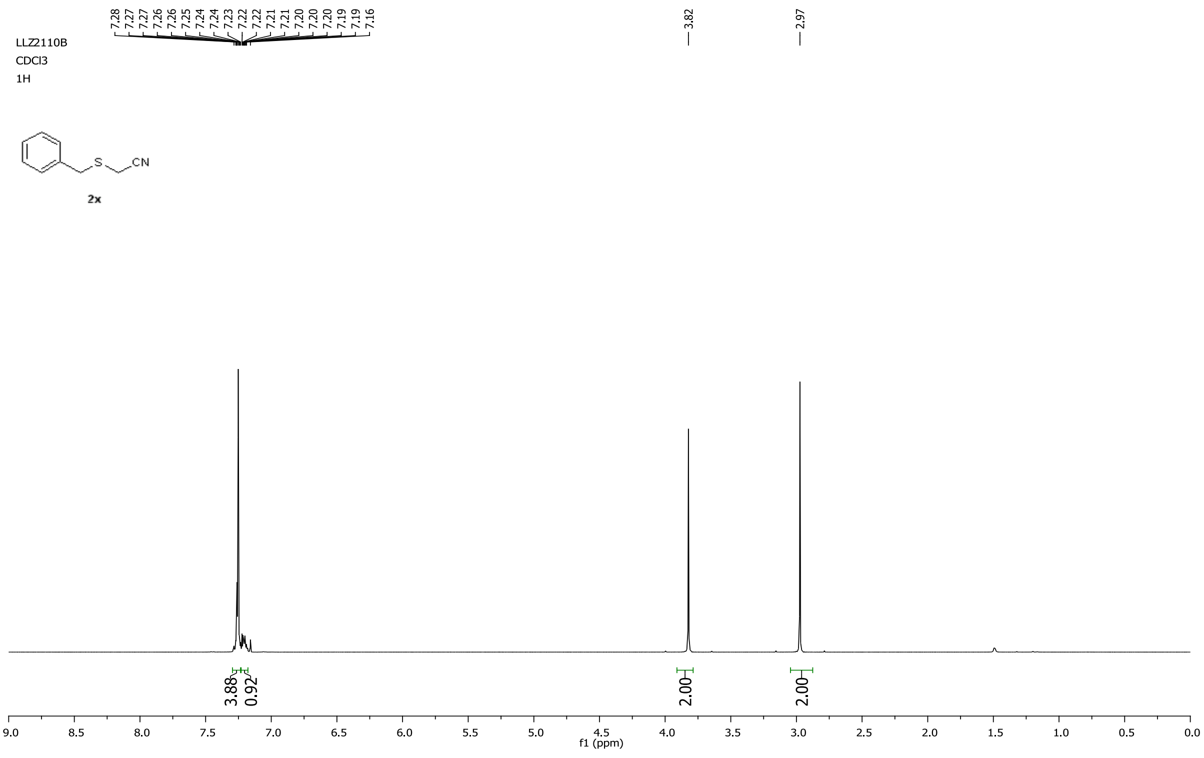


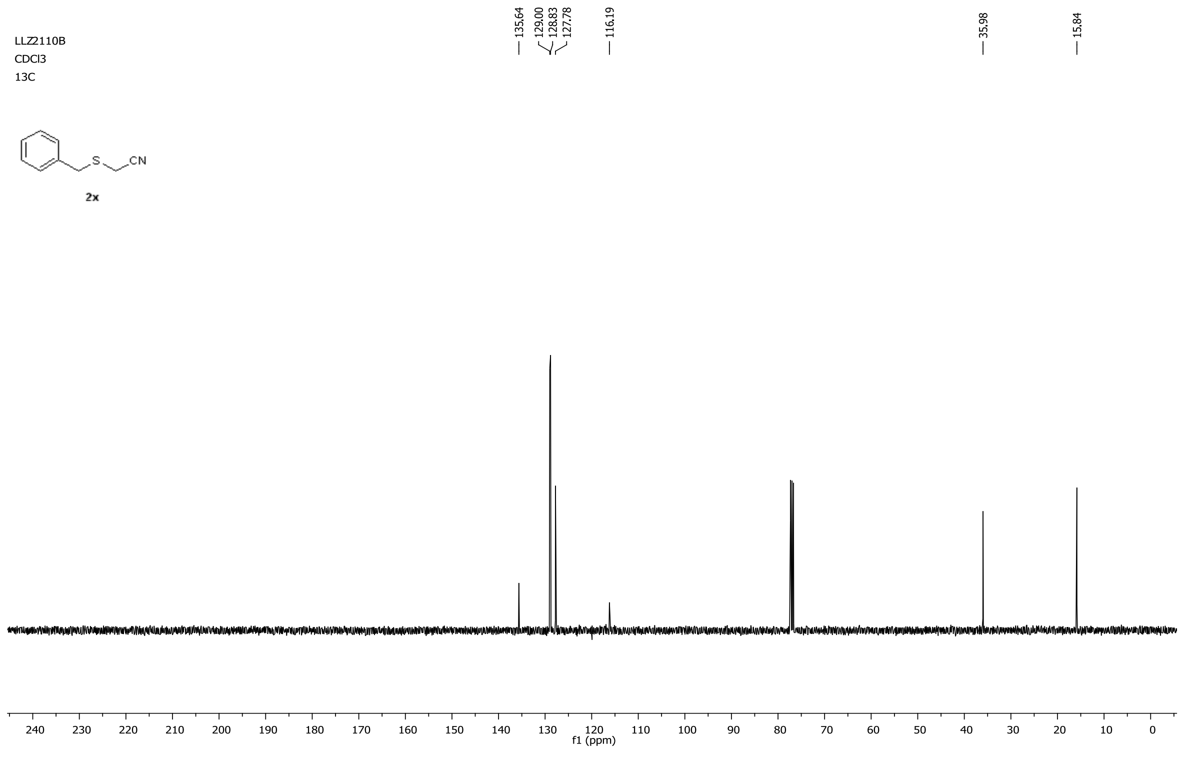


**Supplementary Figure 43.** $^{1}$H and $^{13}$C NMR spectra of sulfide **2x**.

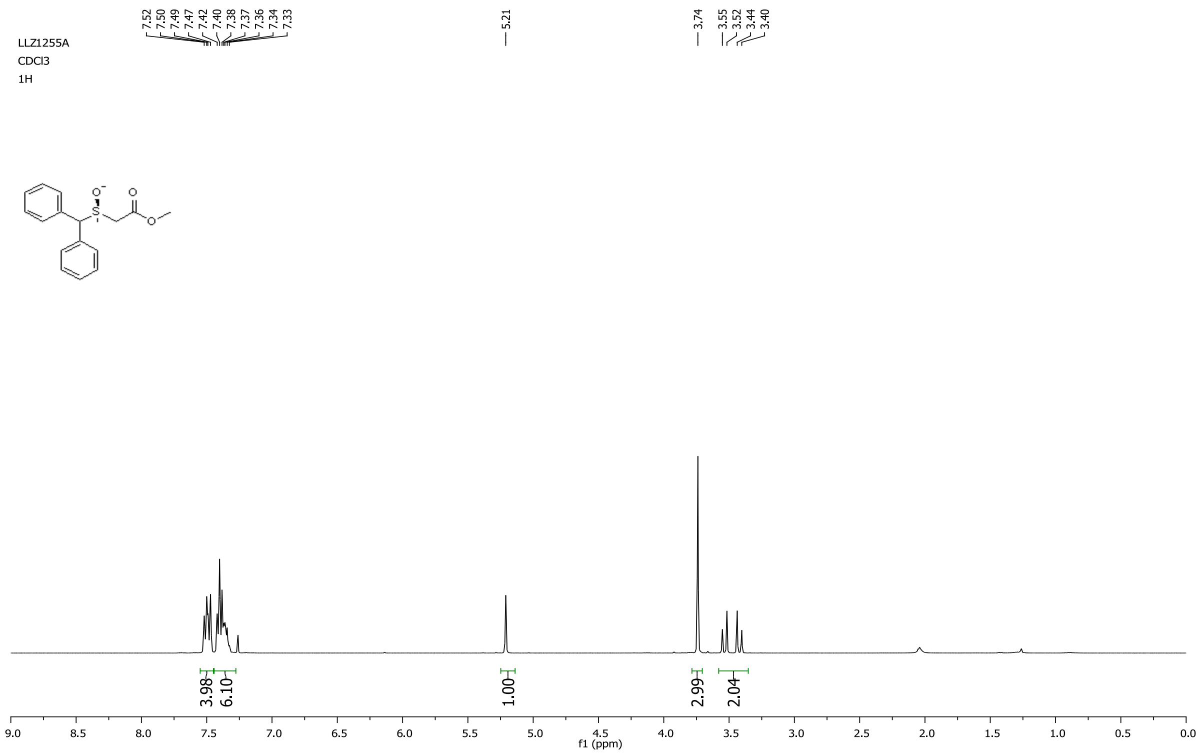


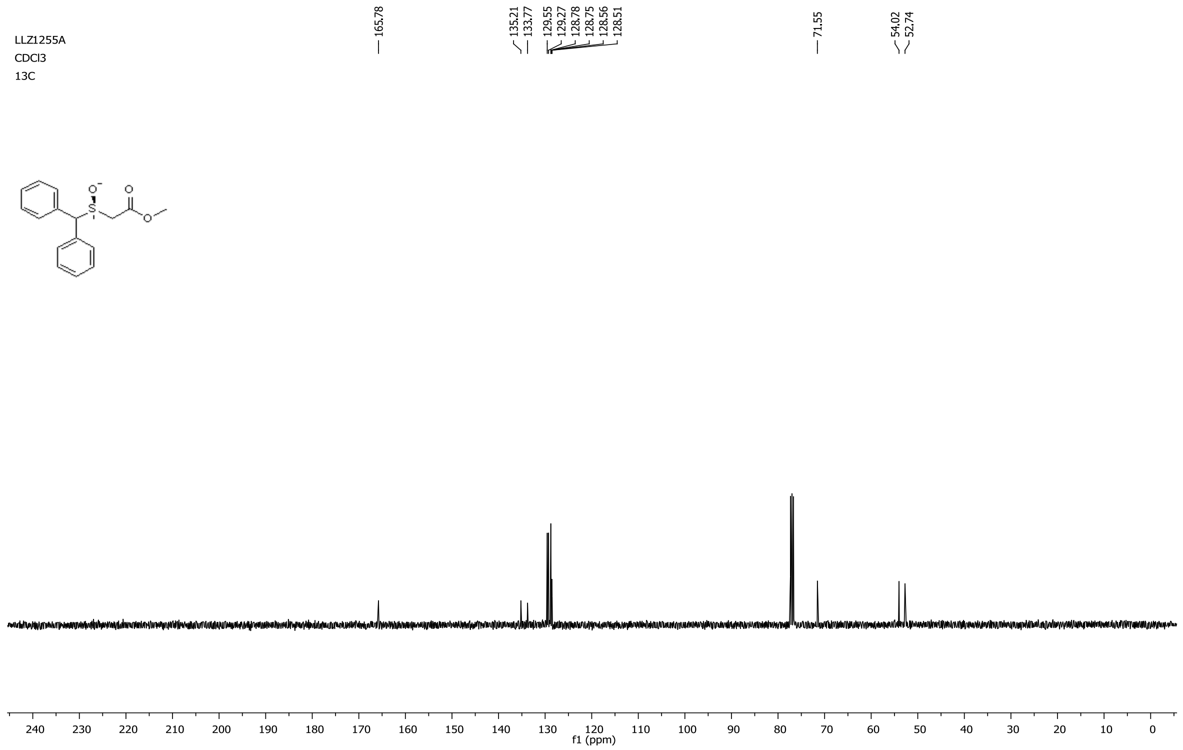


**Supplementary Figure 44.** $^{1}$H and $^{13}$C NMR spectra of sulfoxide **3a**.

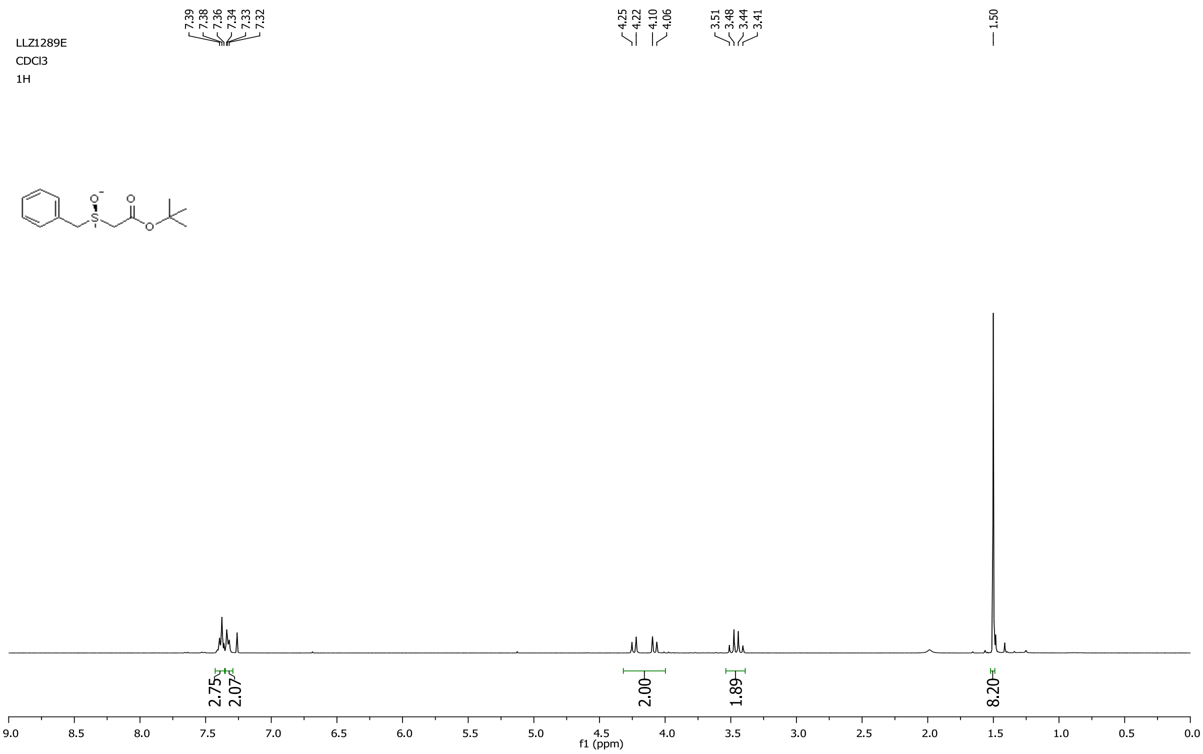


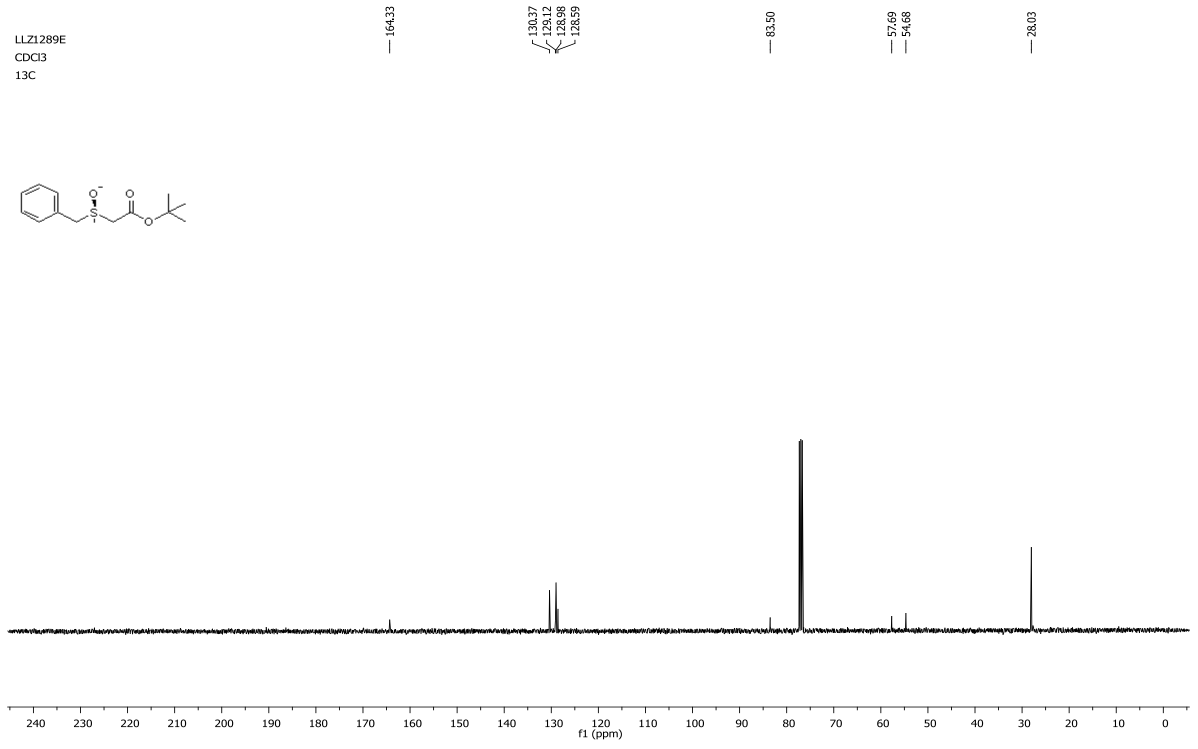


**Supplementary Figure 45.** $^{1}$H and $^{13}$C NMR spectra of sulfoxide **3b**.

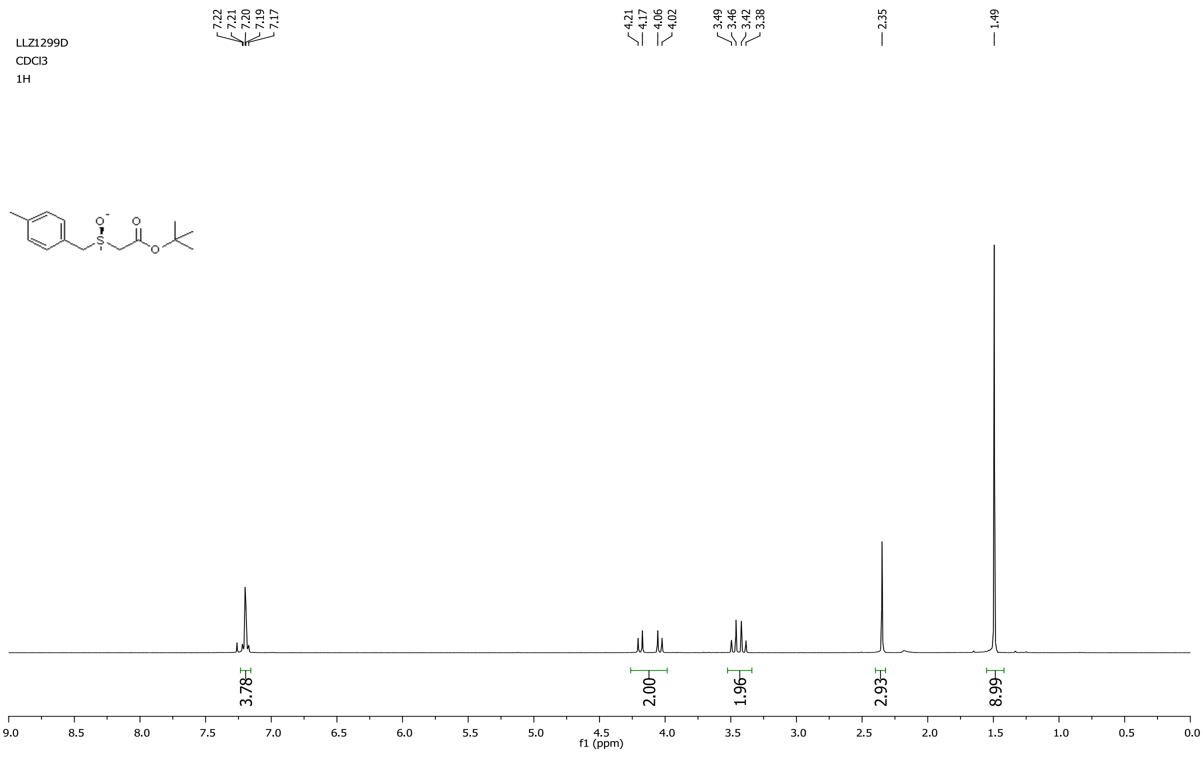


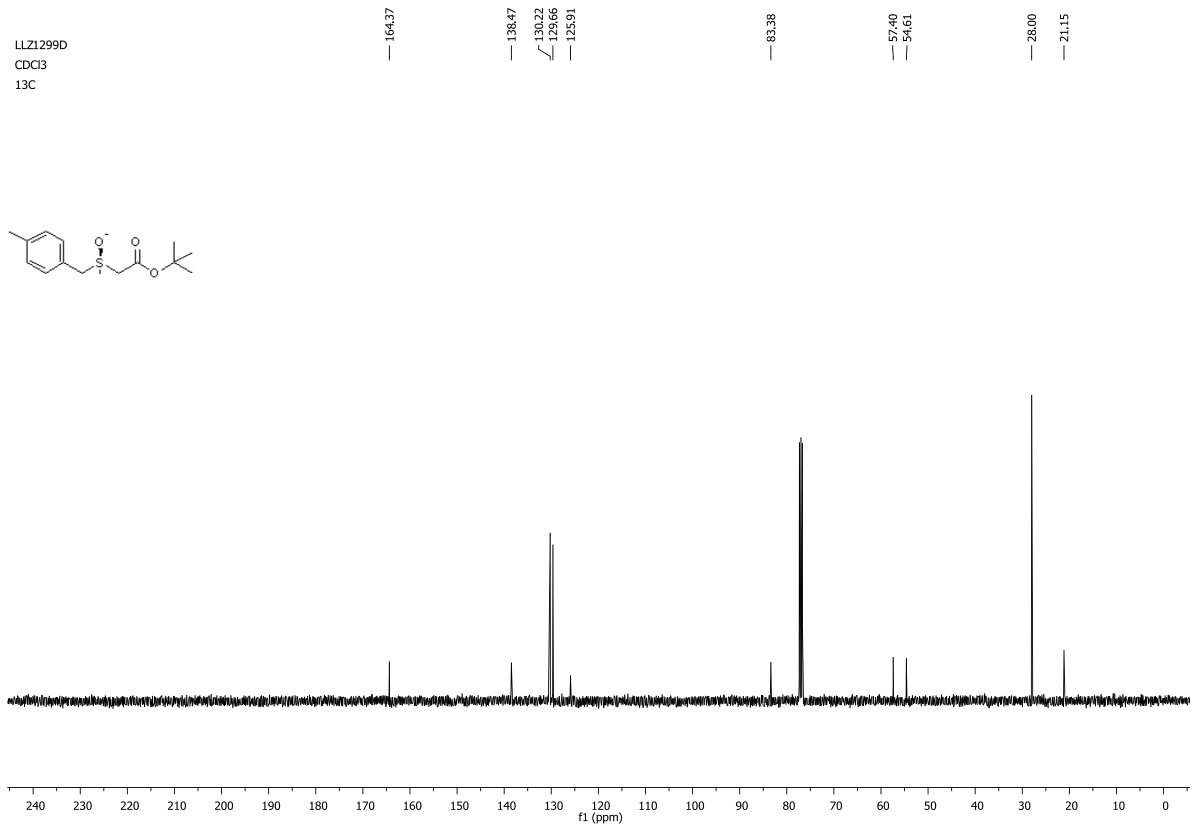


**Supplementary Figure 46.** $^{1}$H and $^{13}$C NMR spectra of sulfoxide **3c**.

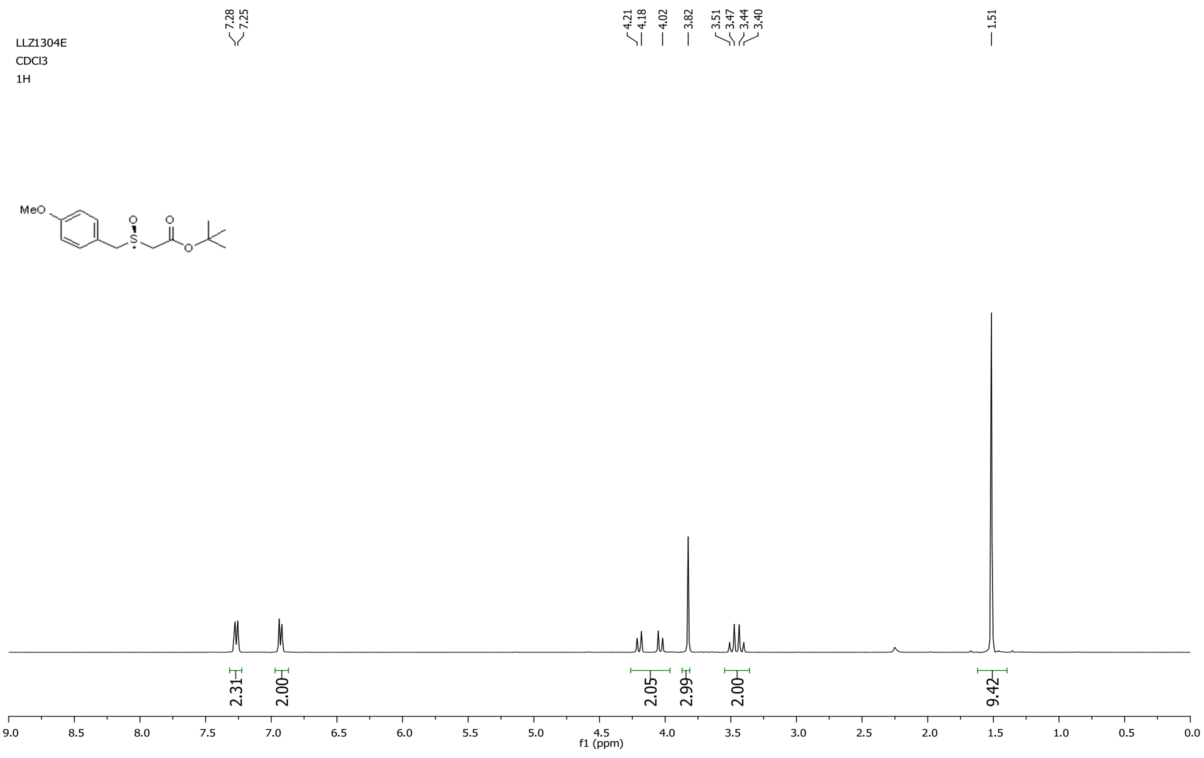


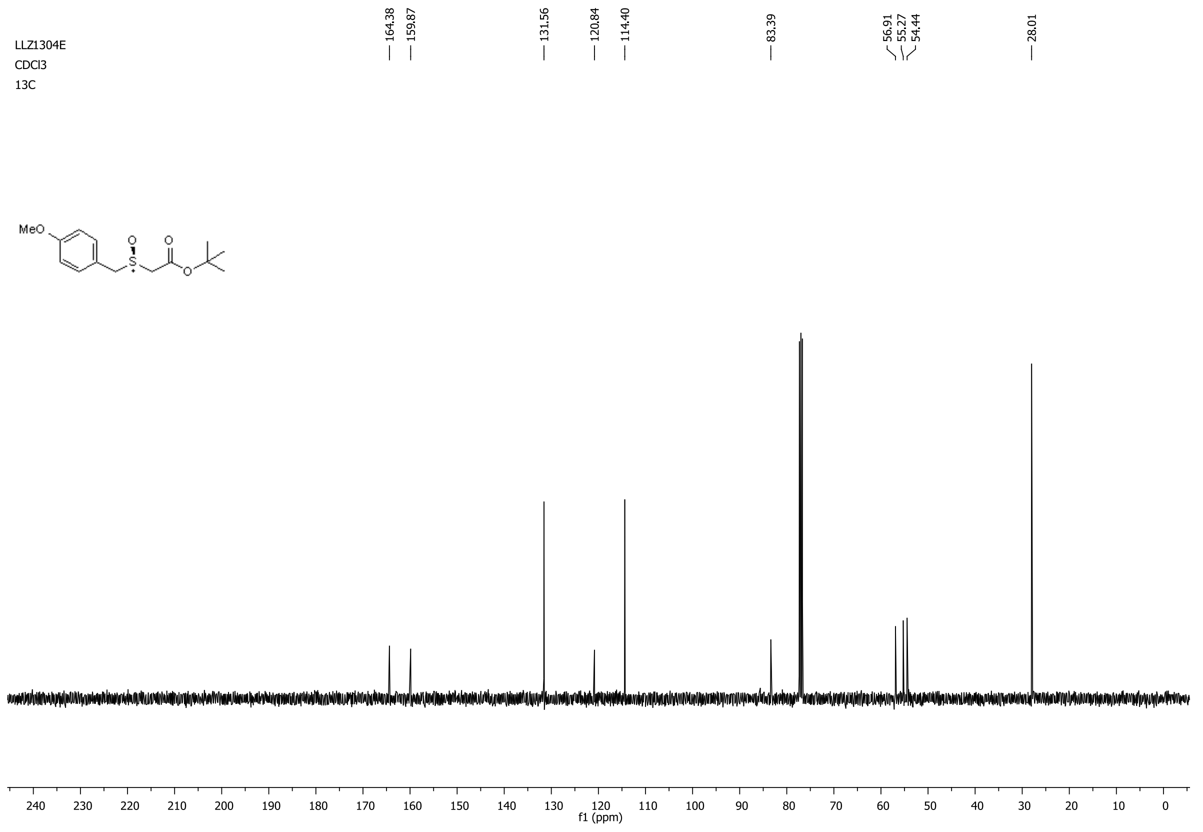


**Supplementary Figure 47.** $^{1}$H and $^{13}$C NMR spectra of sulfoxide **3d**.

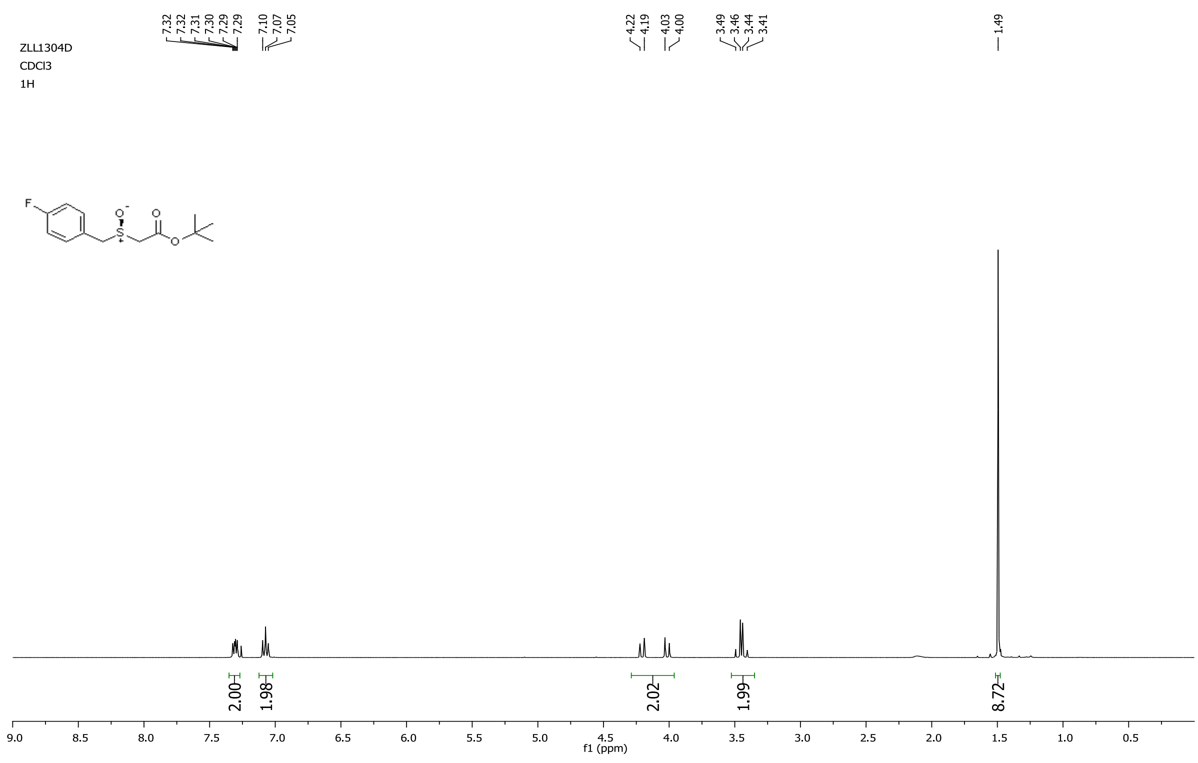


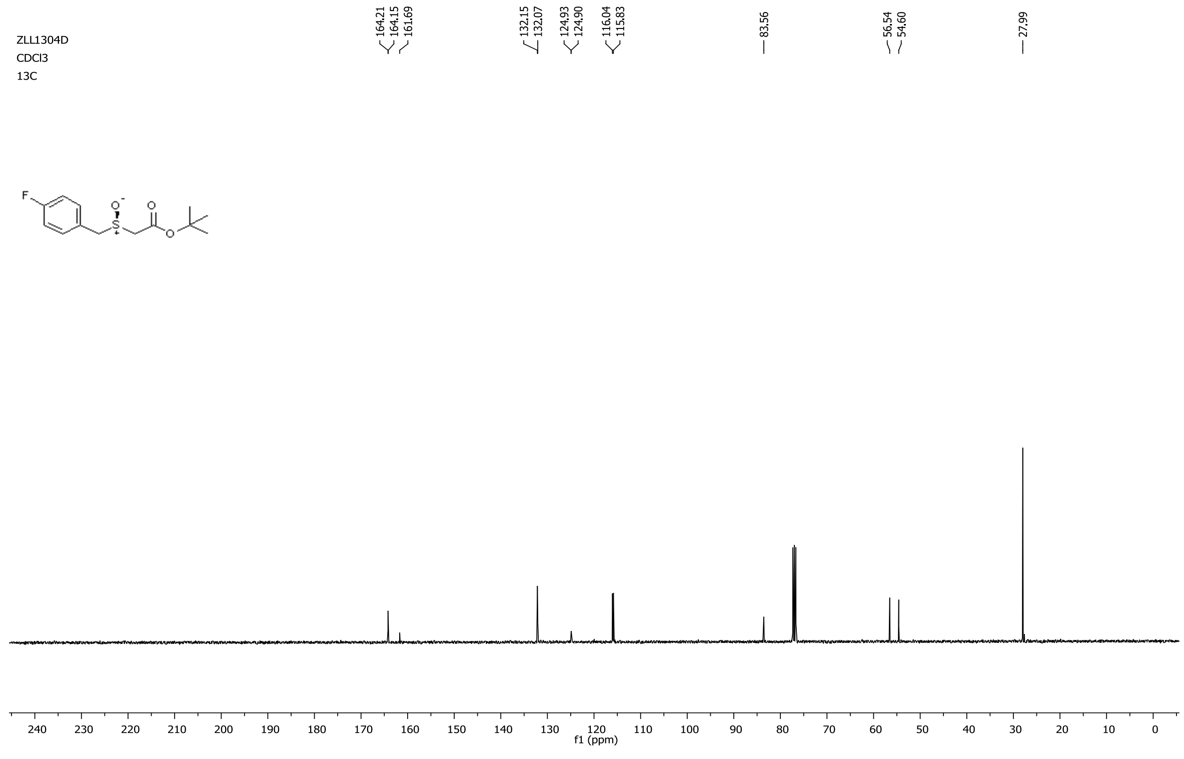


**Supplementary Figure 48.** $^{1}$H and $^{13}$C NMR spectra of sulfoxide **3e**.

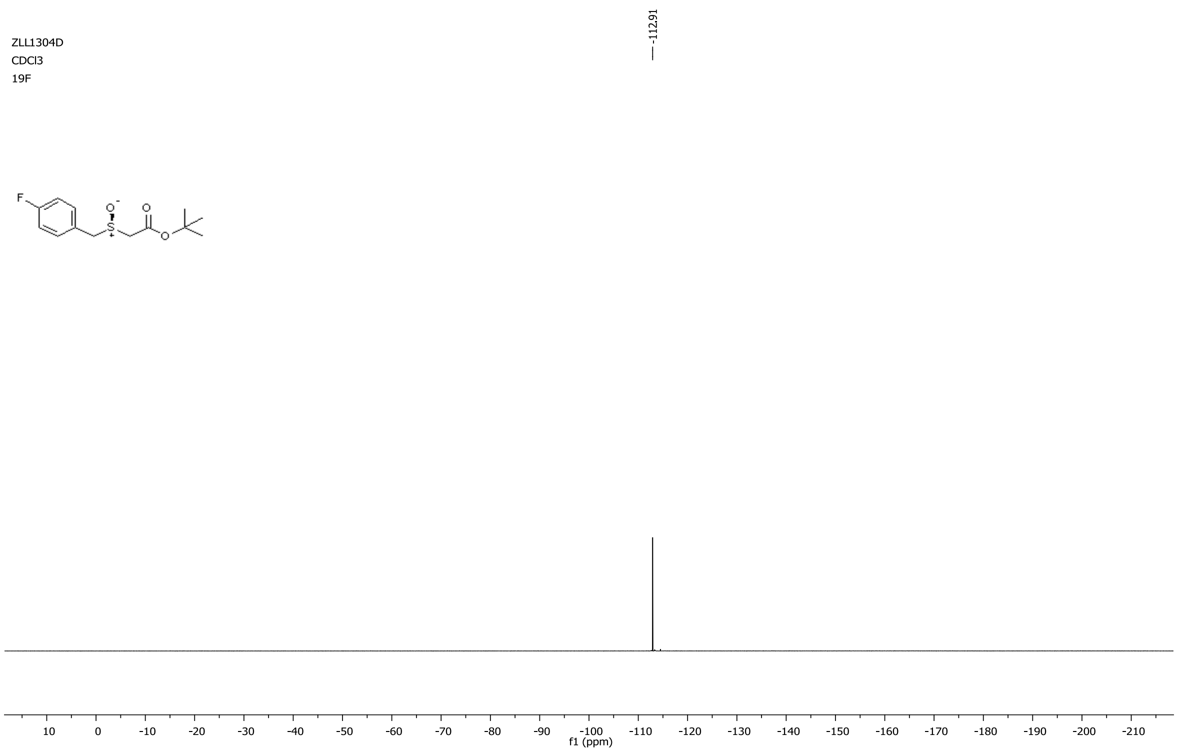


**Supplementary Figure 49.** $^{19}$F NMR spectrum of sulfoxide **3e**.

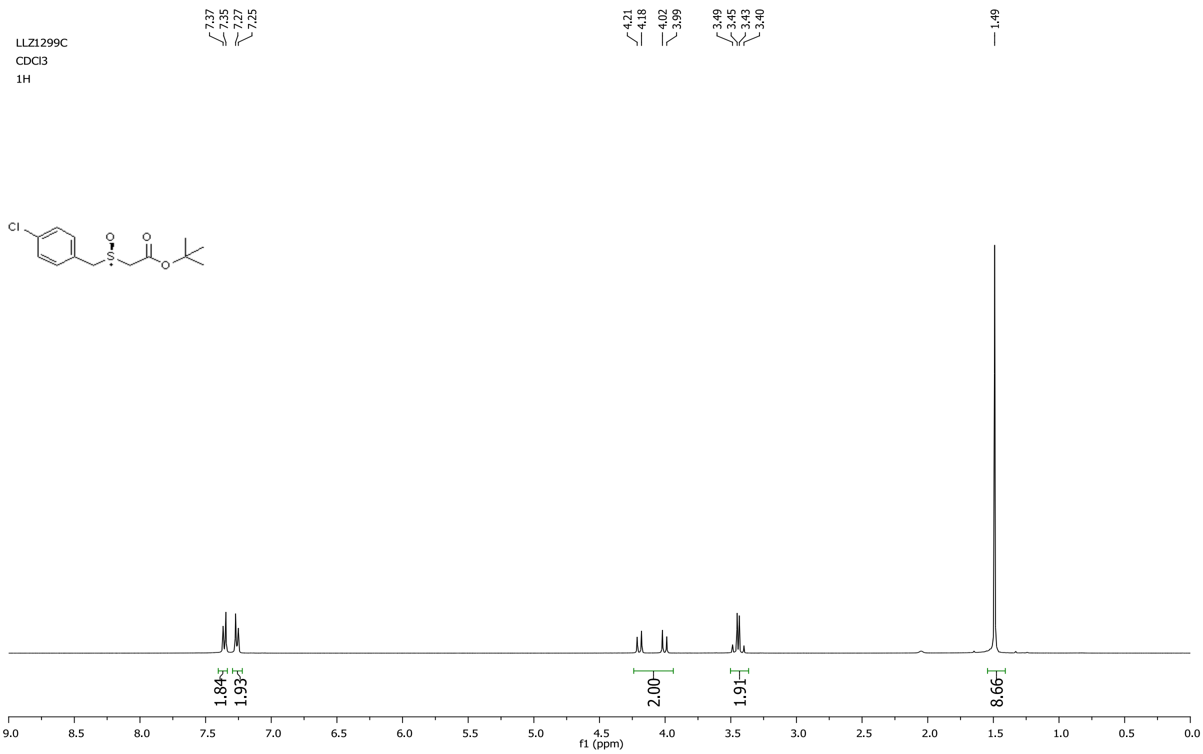


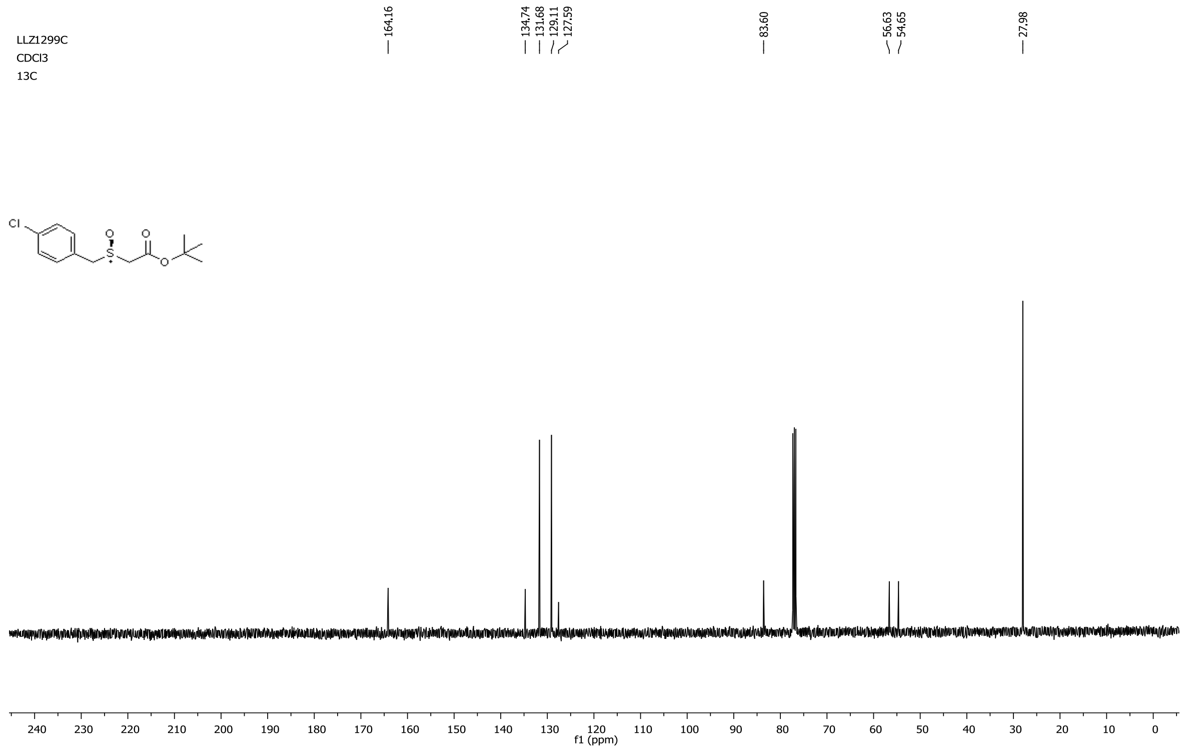


**Supplementary Figure 50.** $^{1}H$ and $^{13}C$ NMR spectra of sulfoxide **3f**.

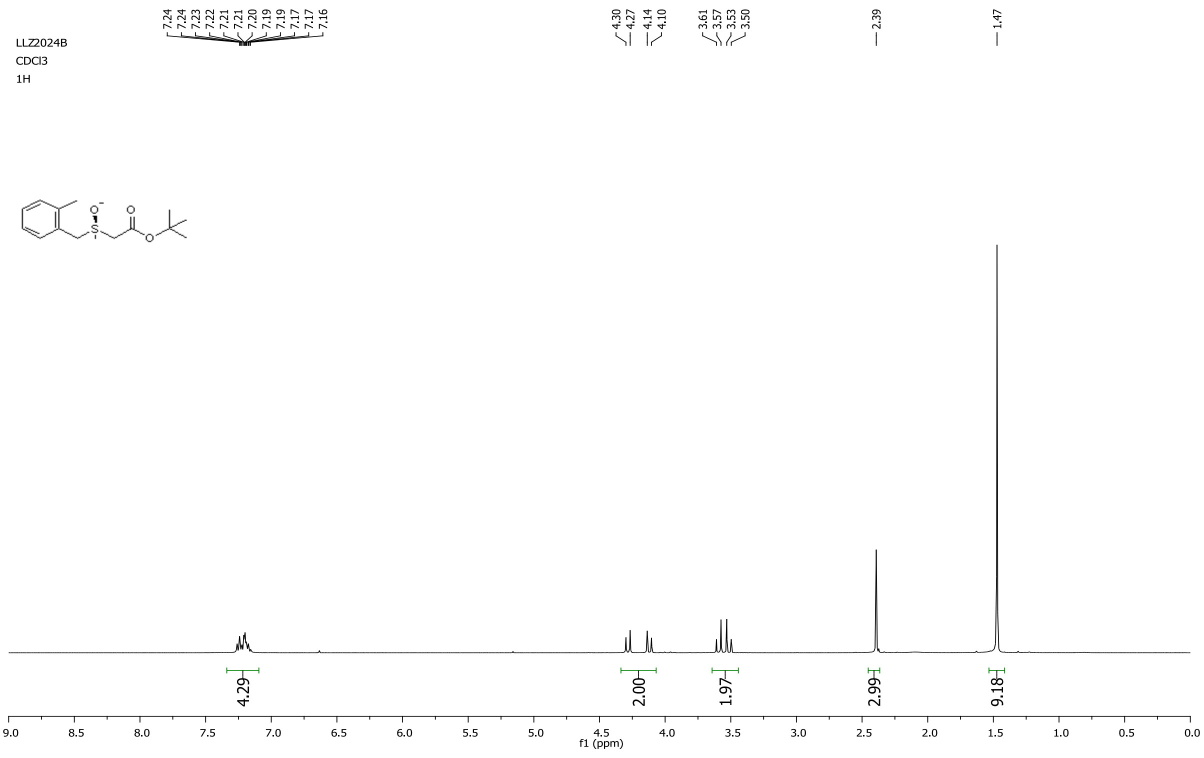


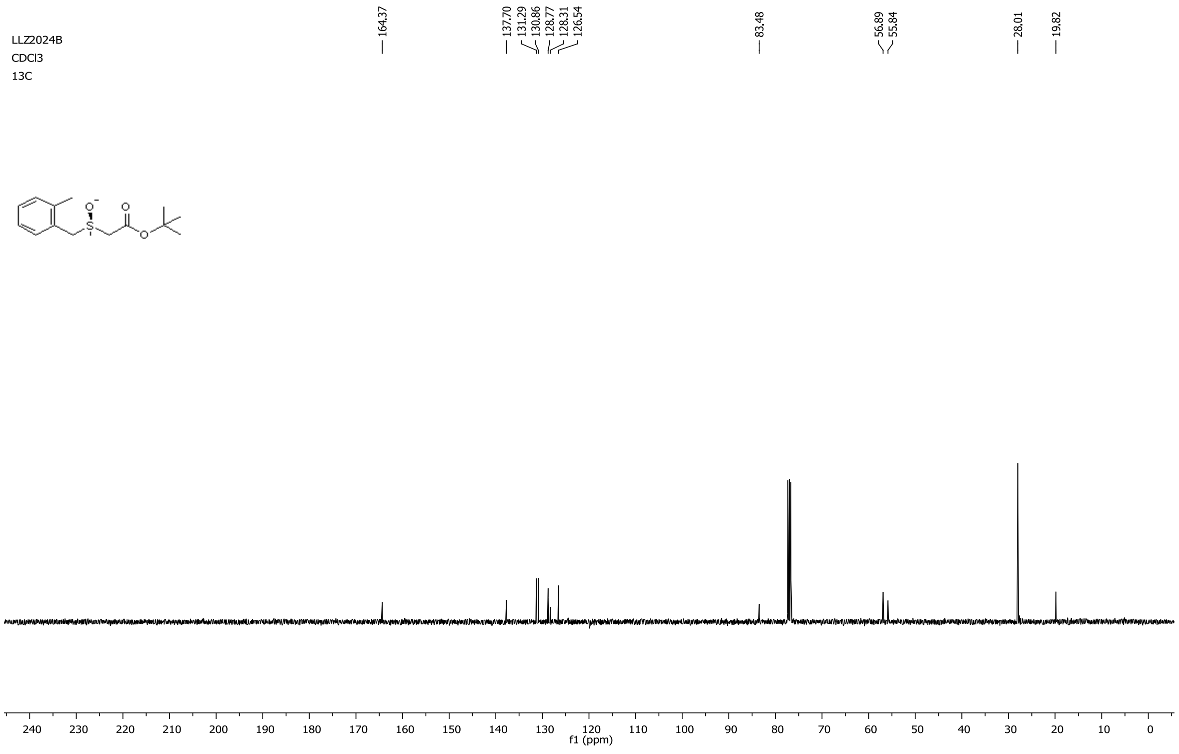


**Supplementary Figure 51.** $^{1}$H and $^{13}$C NMR spectra of sulfoxide **3g**.

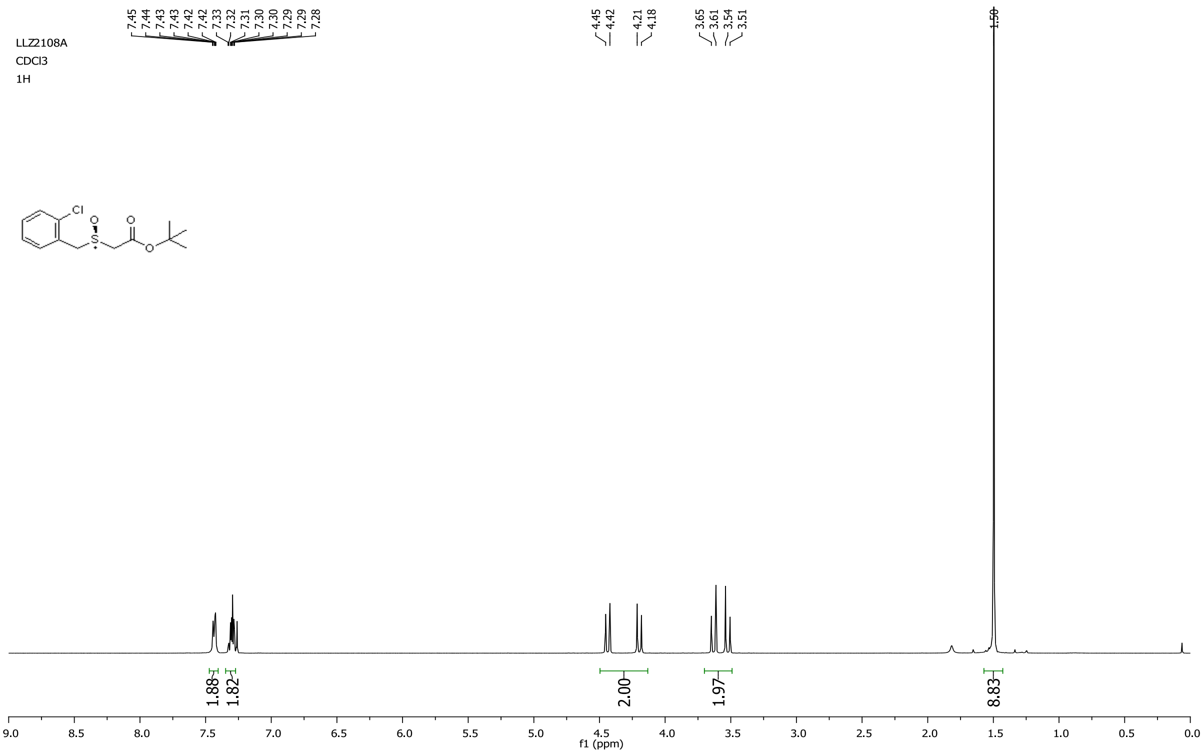


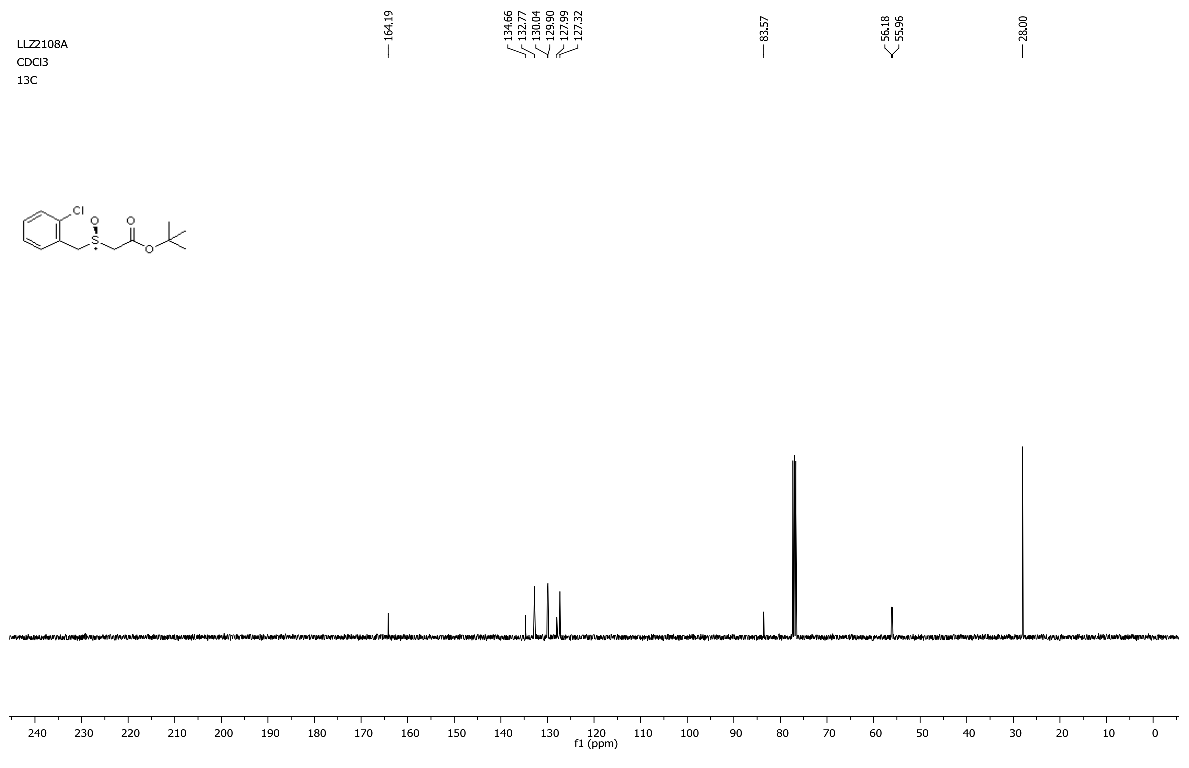


**Supplementary Figure 52.** $^{1}H$ and $^{13}C$ NMR spectra of sulfoxide **3h**.

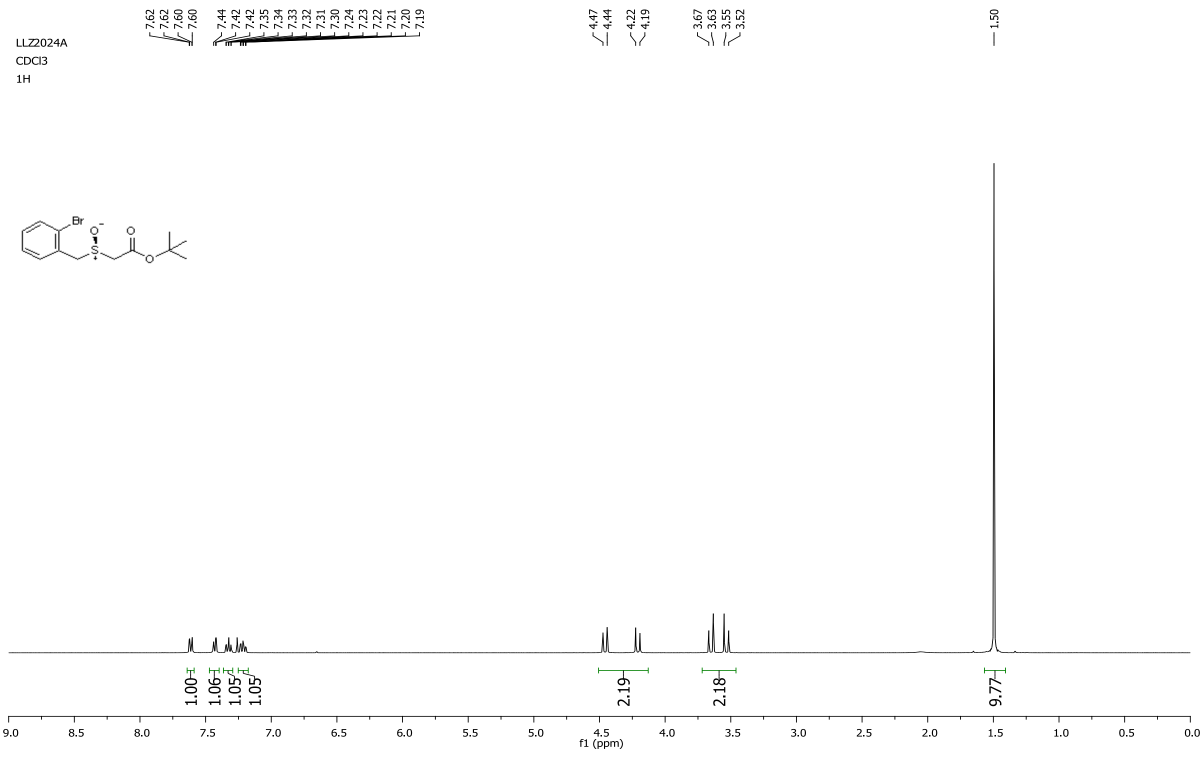


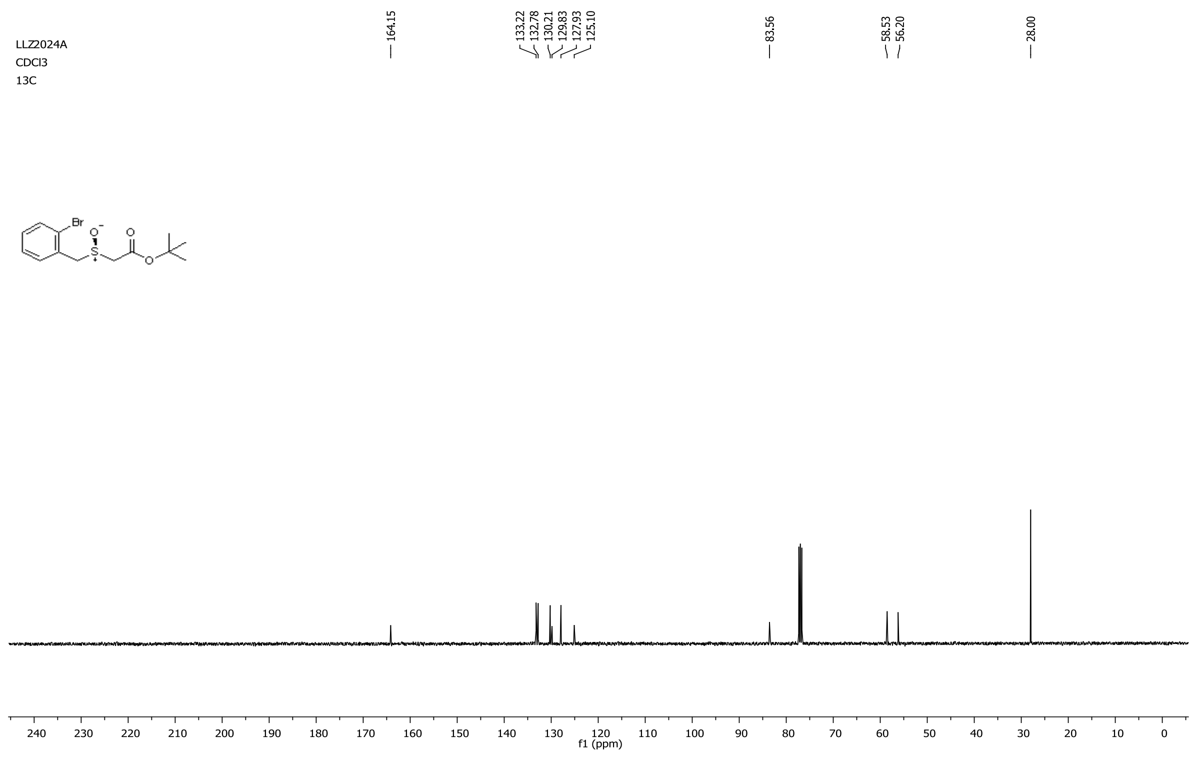


**Supplementary Figure 53.** $^{1}H$ and $^{13}C$ NMR spectra of sulfoxide **3i**.

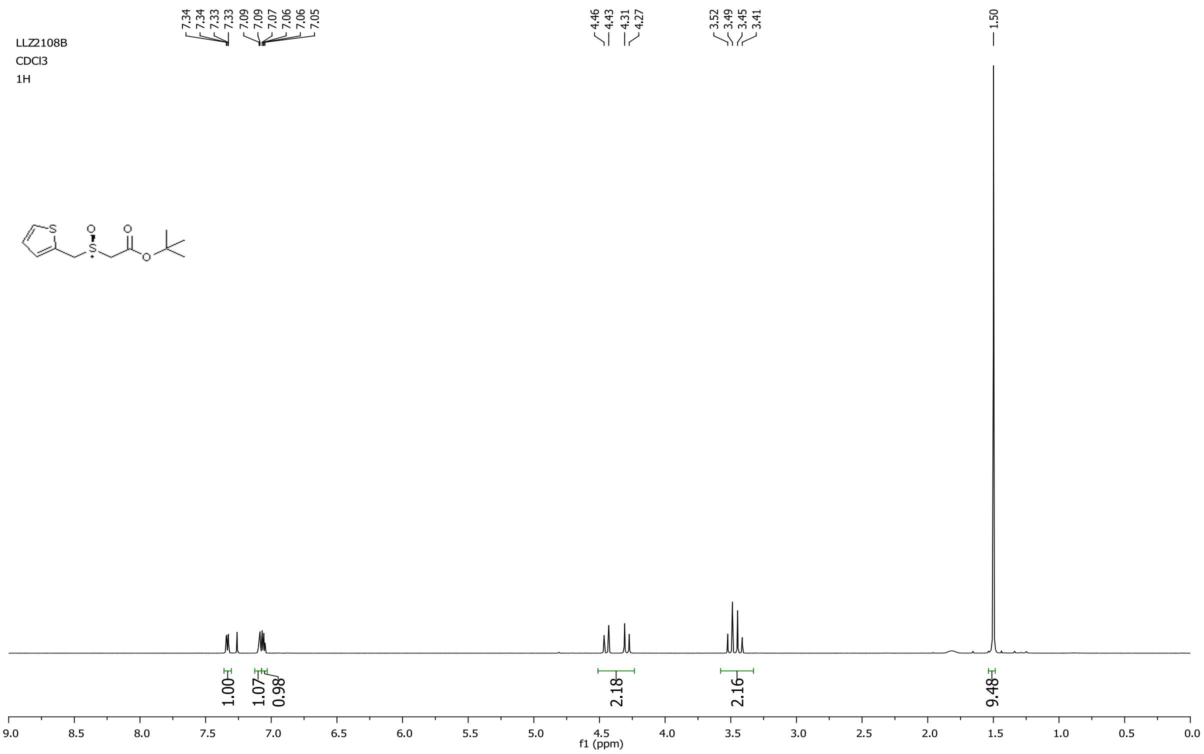


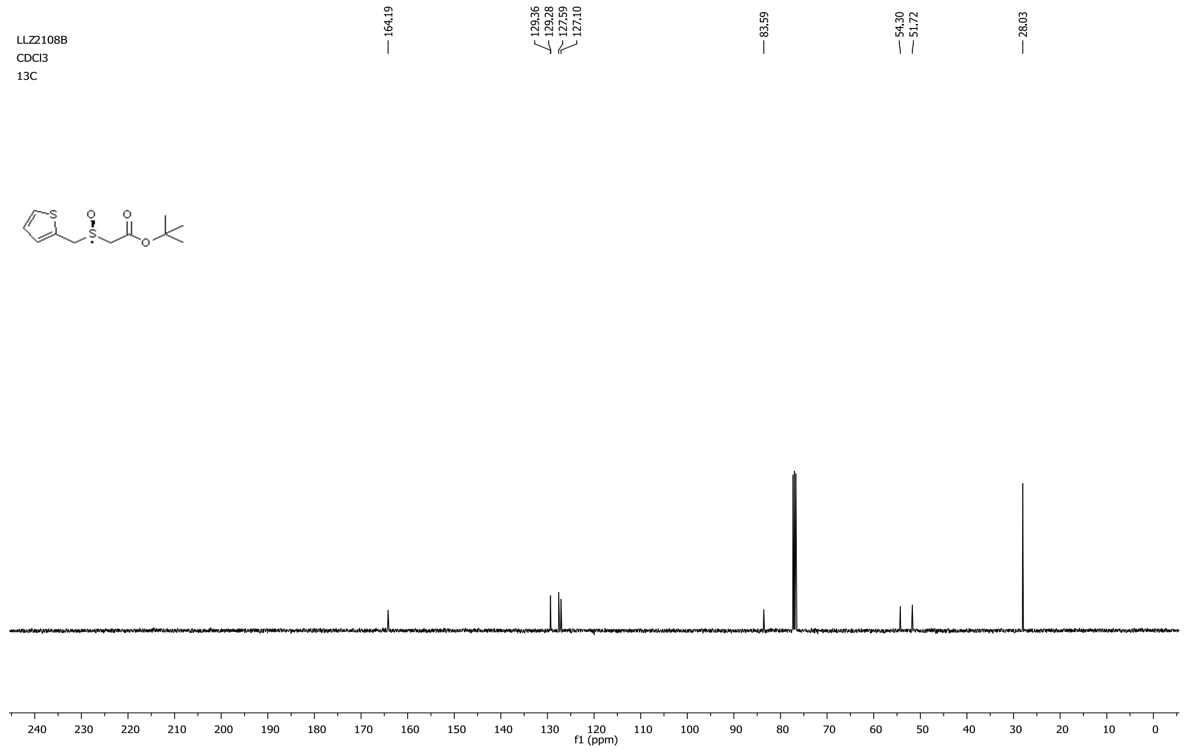


**Supplementary Figure 54.** $^{1}$H and $^{13}$C NMR spectra of sulfoxide **3j**.

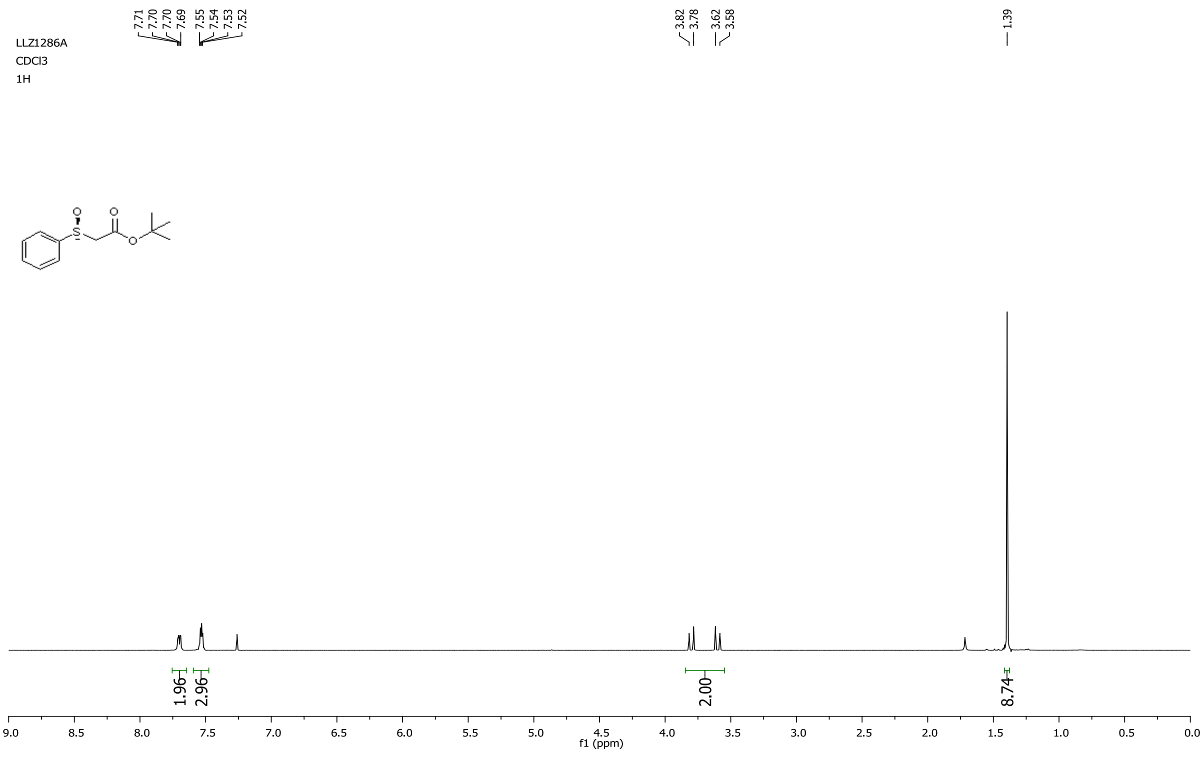


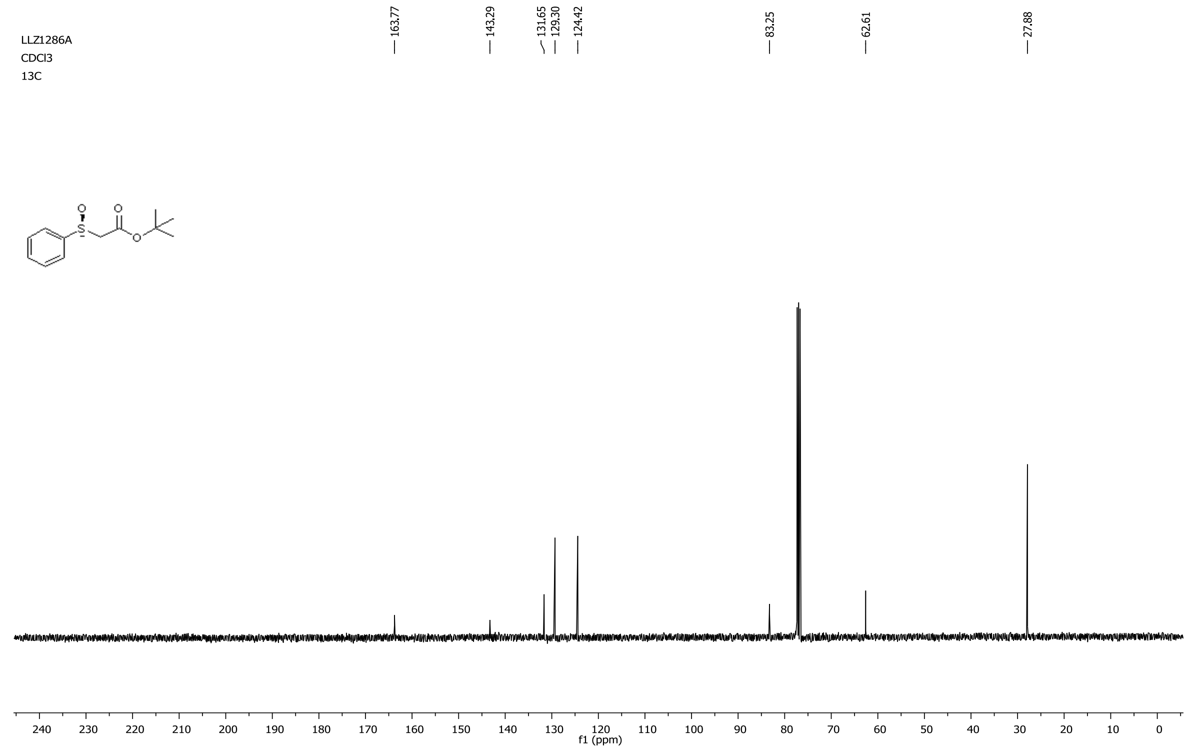


**Supplementary Figure 55.** $^{1}$H and $^{13}$C NMR spectra of sulfoxide **3k**.

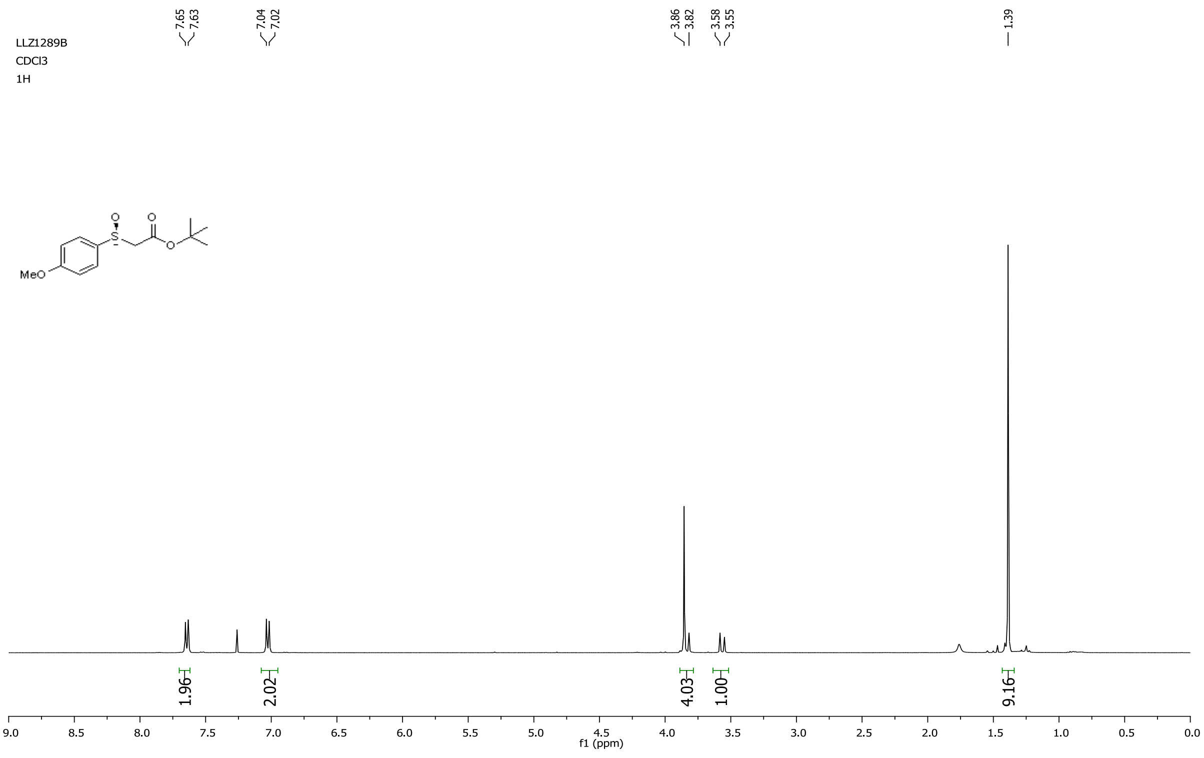


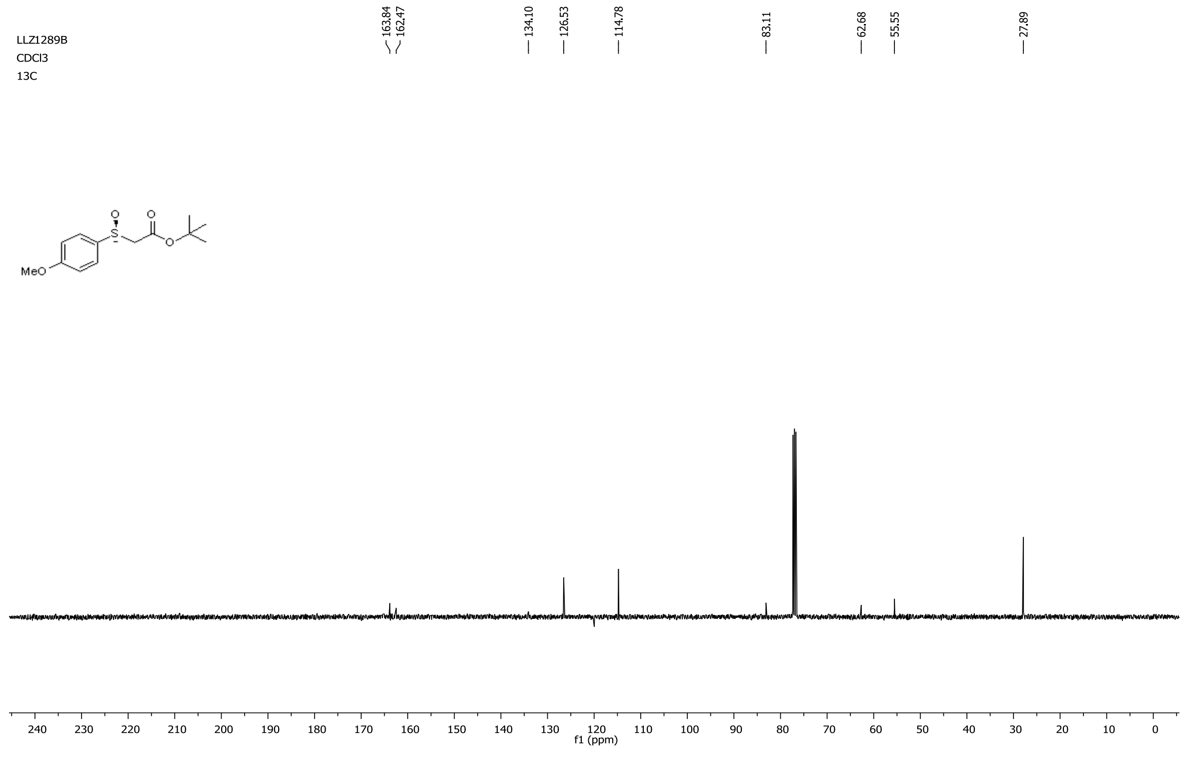


**Supplementary Figure 56.** $^{1}$H and $^{13}$C NMR spectra of sulfoxide **3l**.

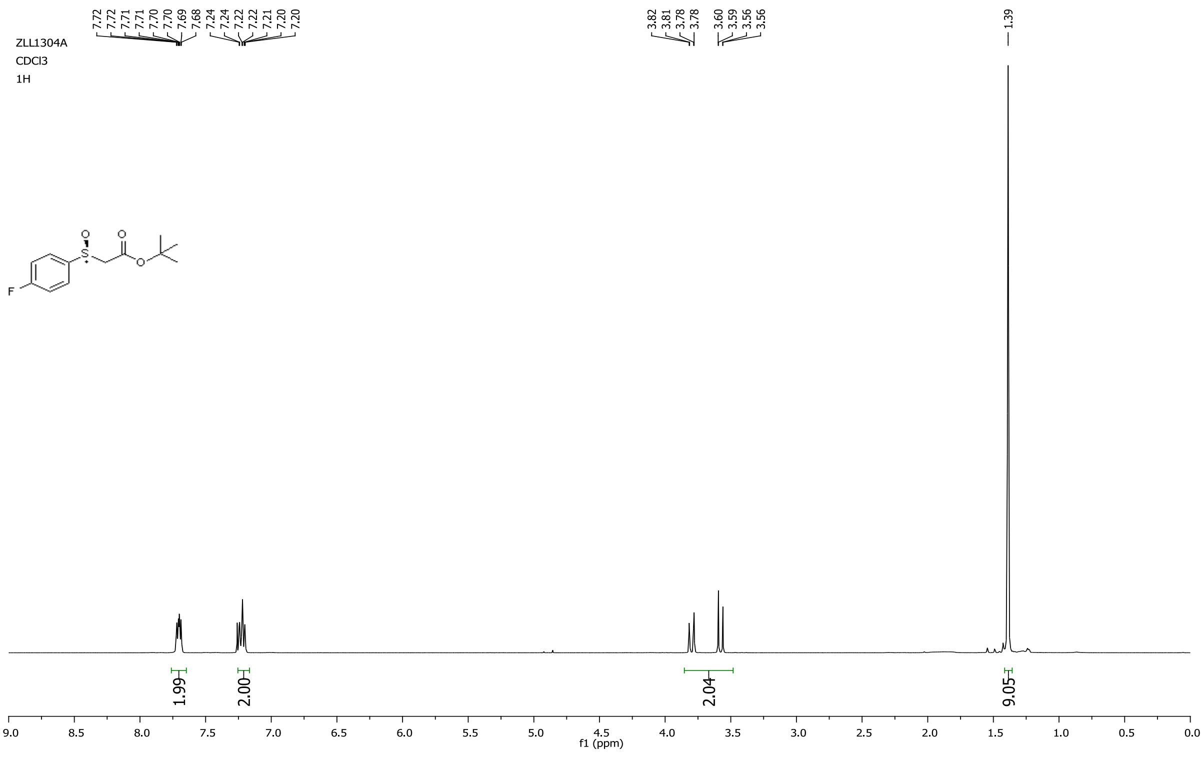


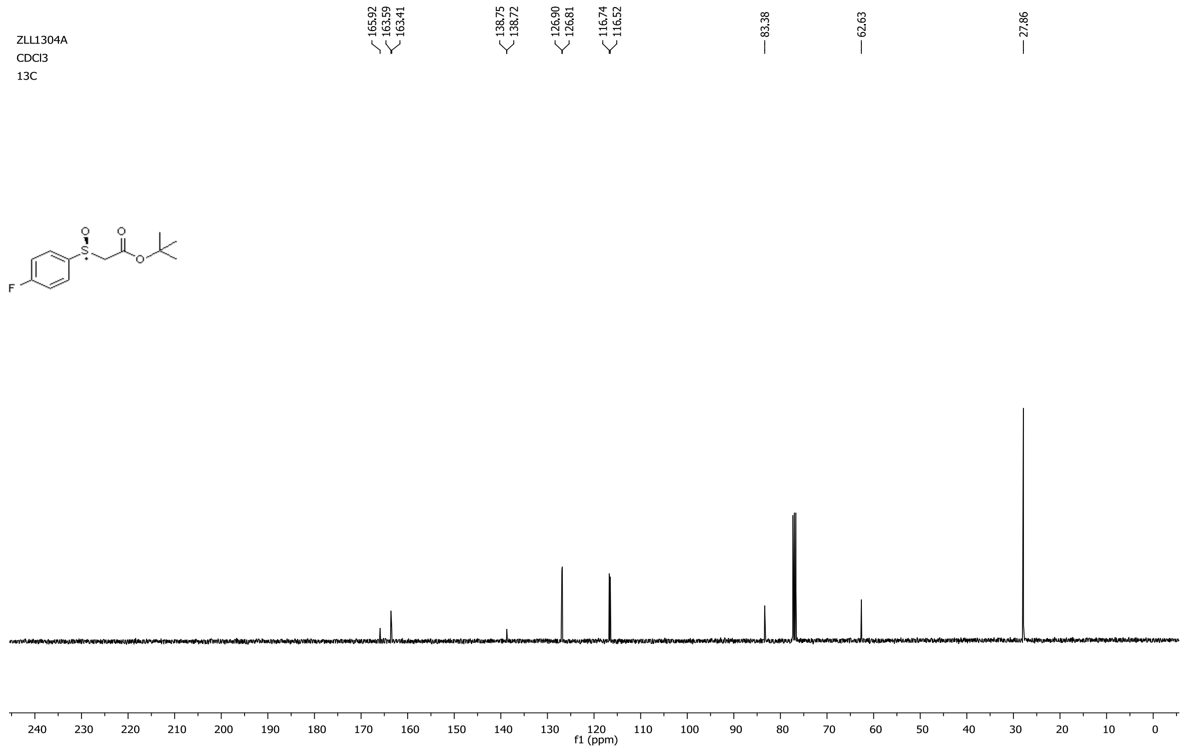


**Supplementary Figure 57.** $^{1}$H and $^{13}$C NMR spectra of sulfoxide **3m**.

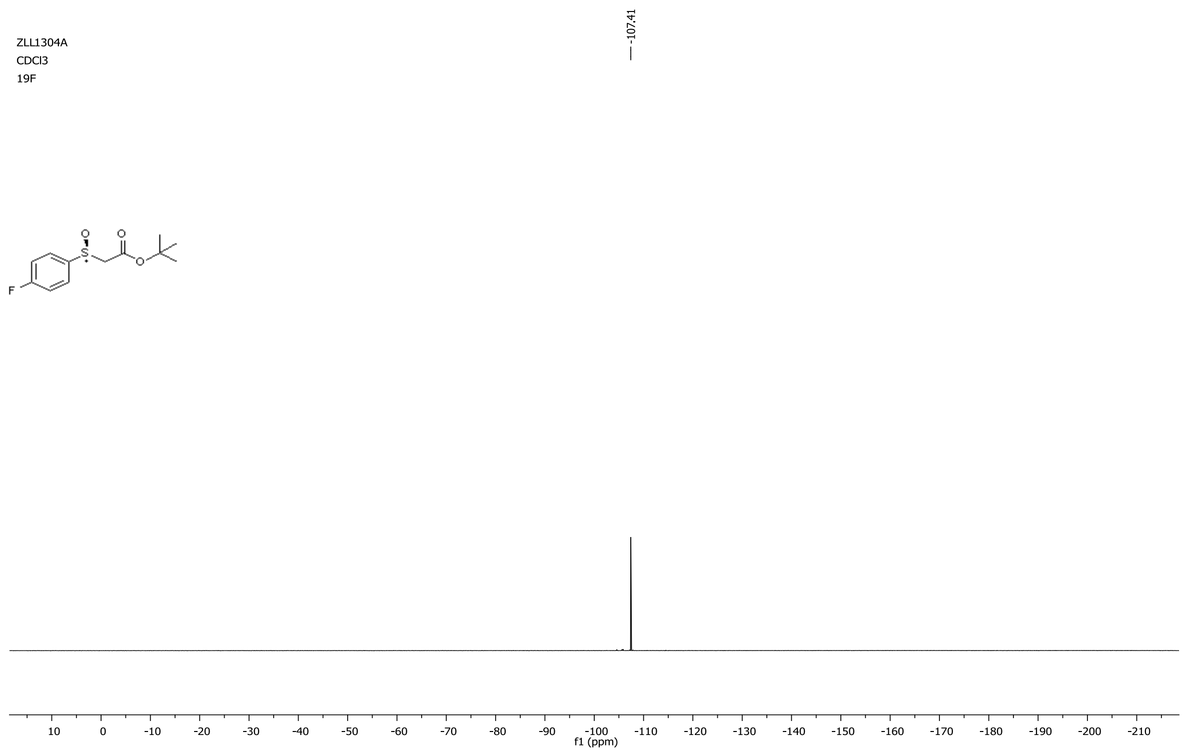


**Supplementary Figure 58.** ${}^{19}F$ NMR spectrum of sulfoxide **3m**.

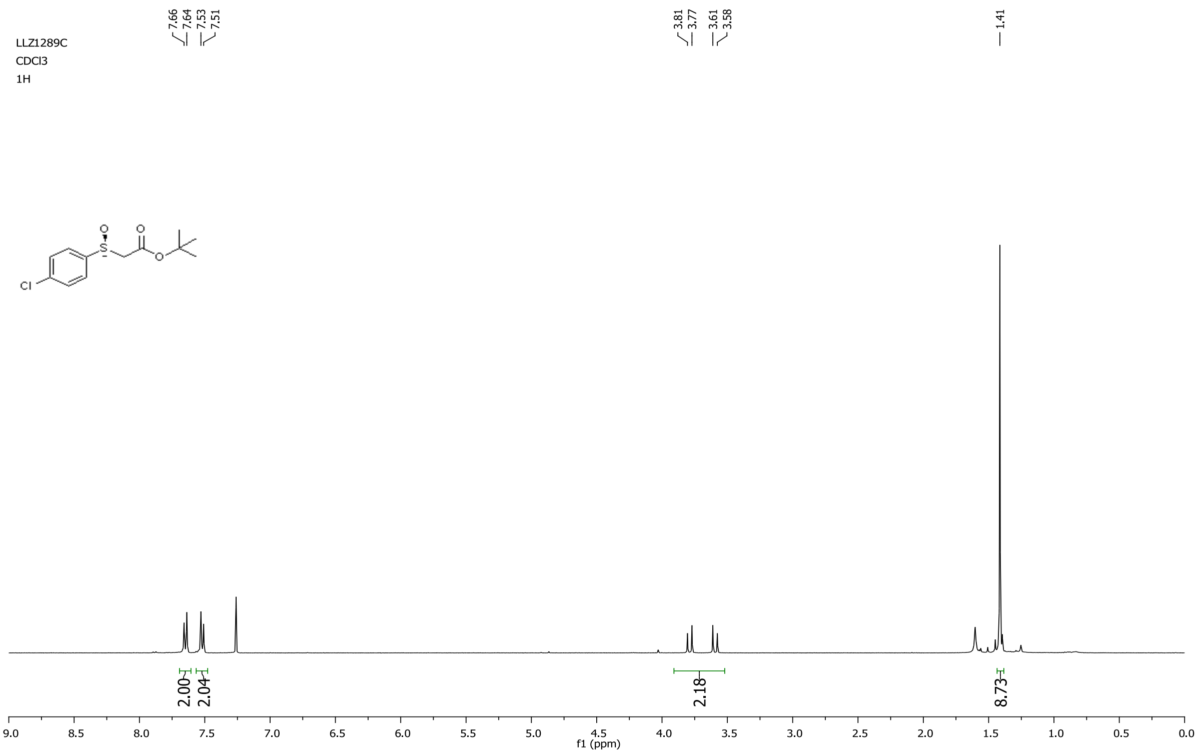


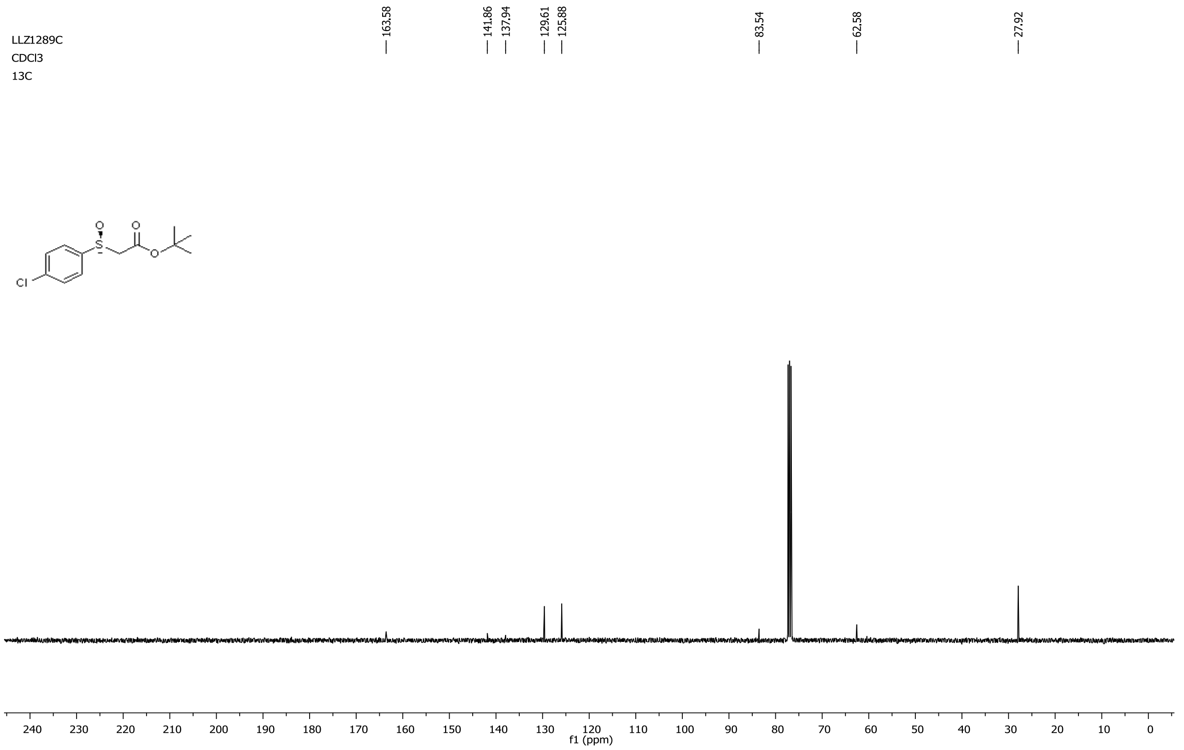


**Supplementary Figure 59.** $^{1}$H and $^{13}$C NMR spectra of sulfoxide **3n**.

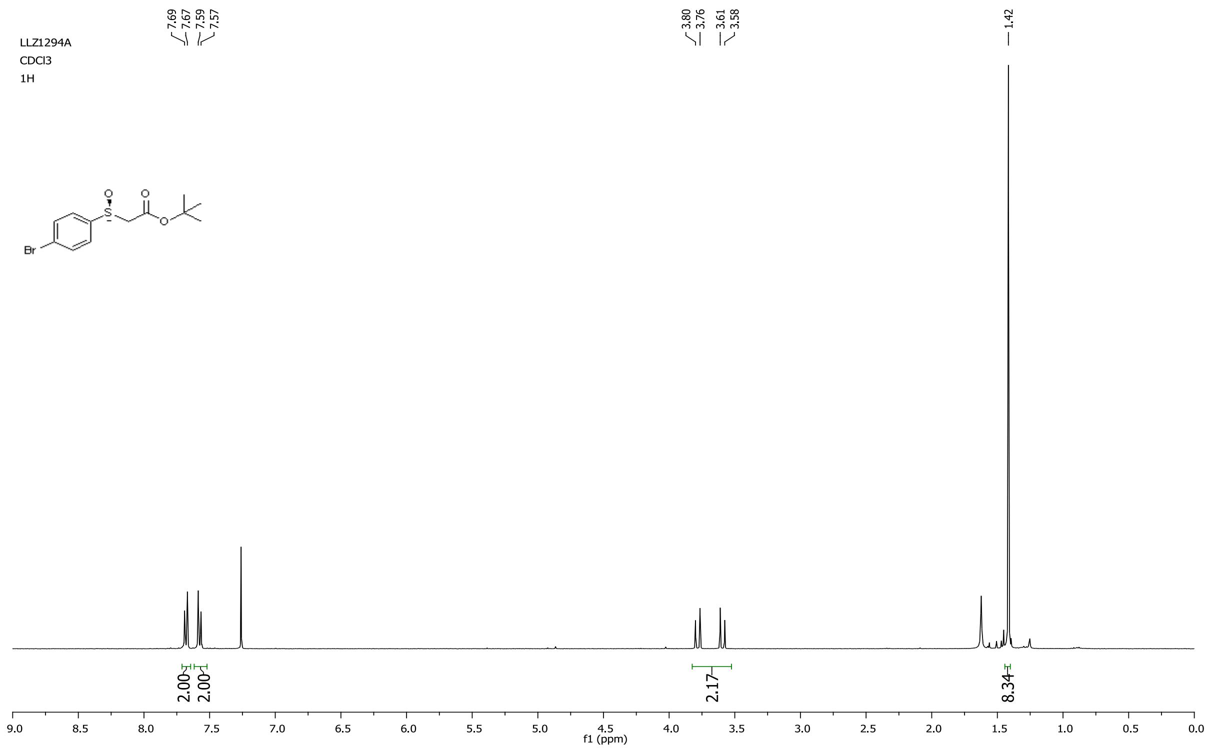


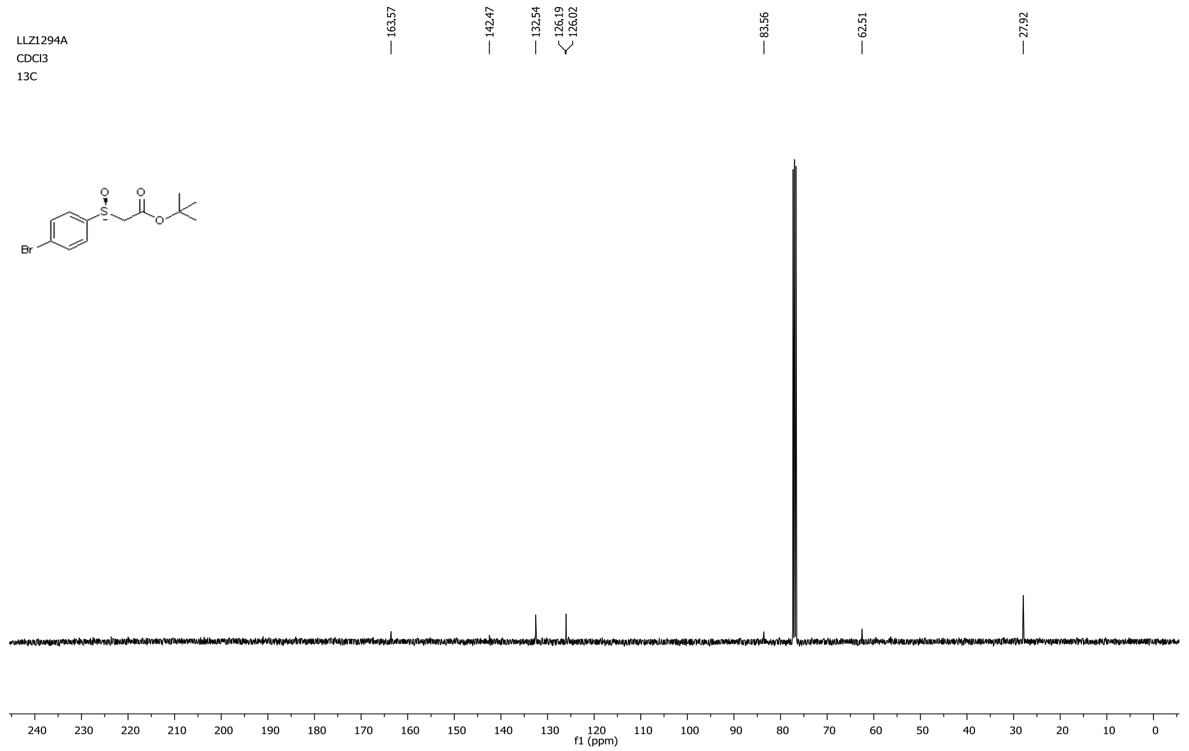


**Supplementary Figure 60.** $^{1}$H and $^{13}$C NMR spectra of sulfoxide **3o**.

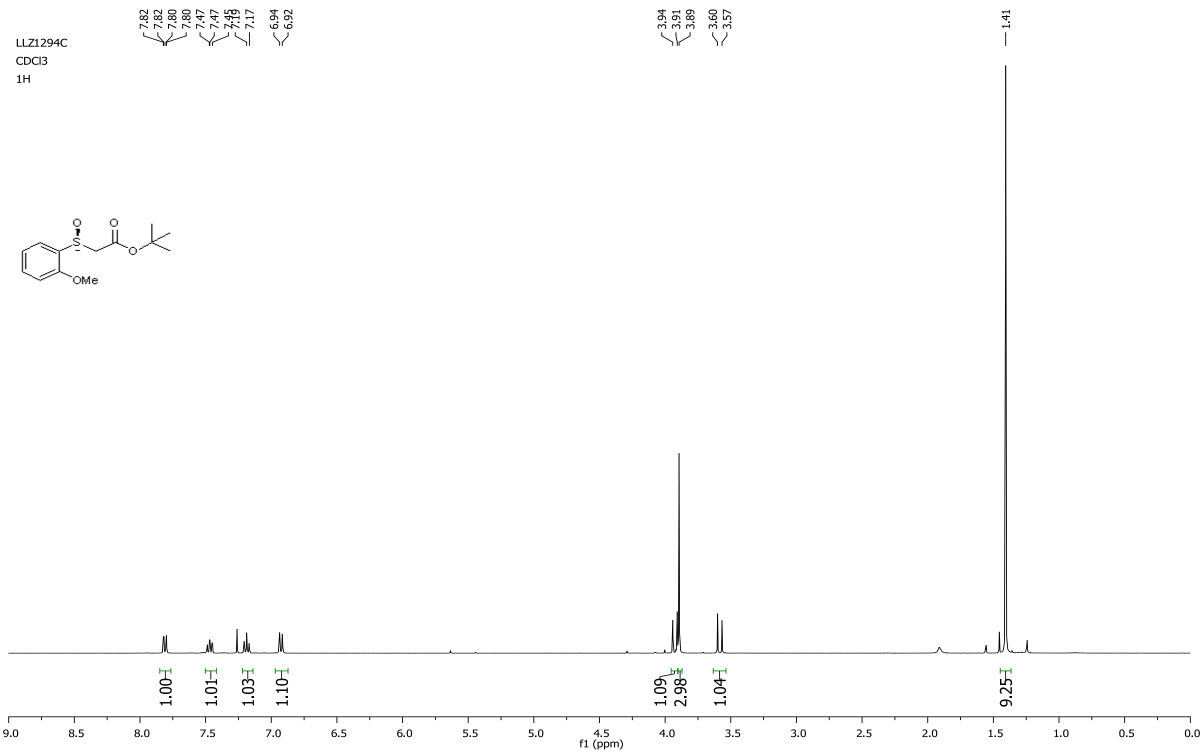


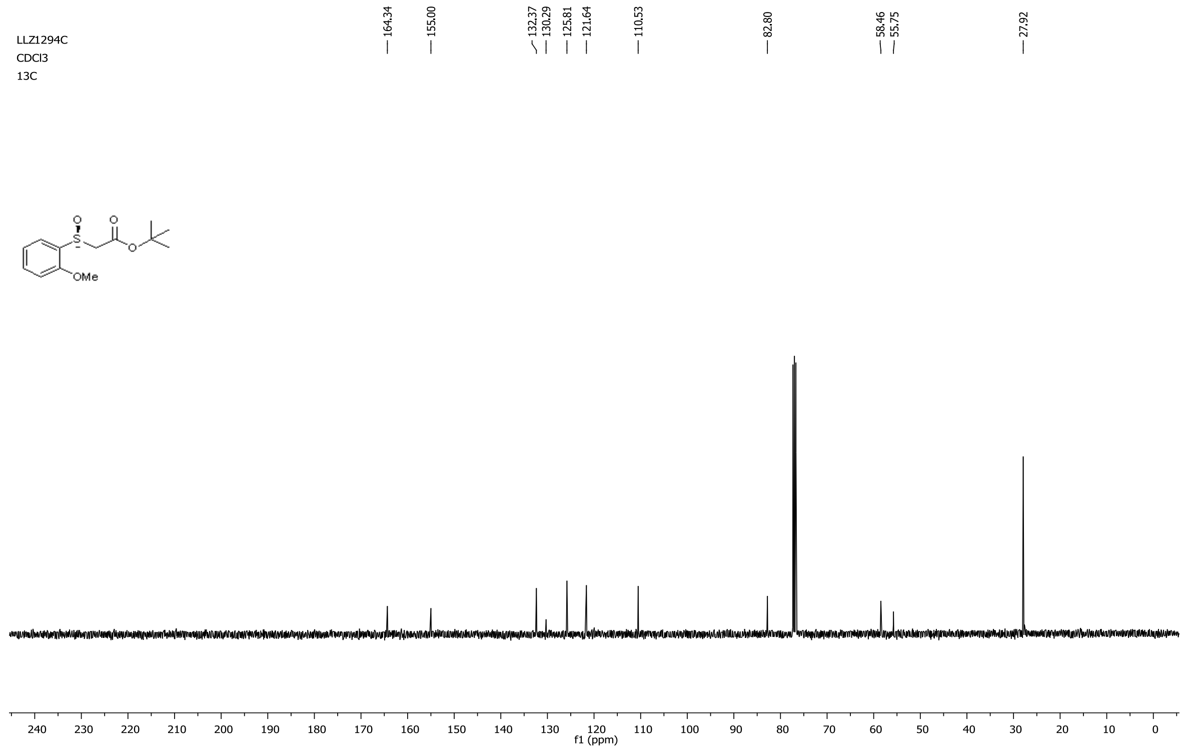


**Supplementary Figure 61.** $^{1}$H and $^{13}$C NMR spectra of sulfoxide **3p**.

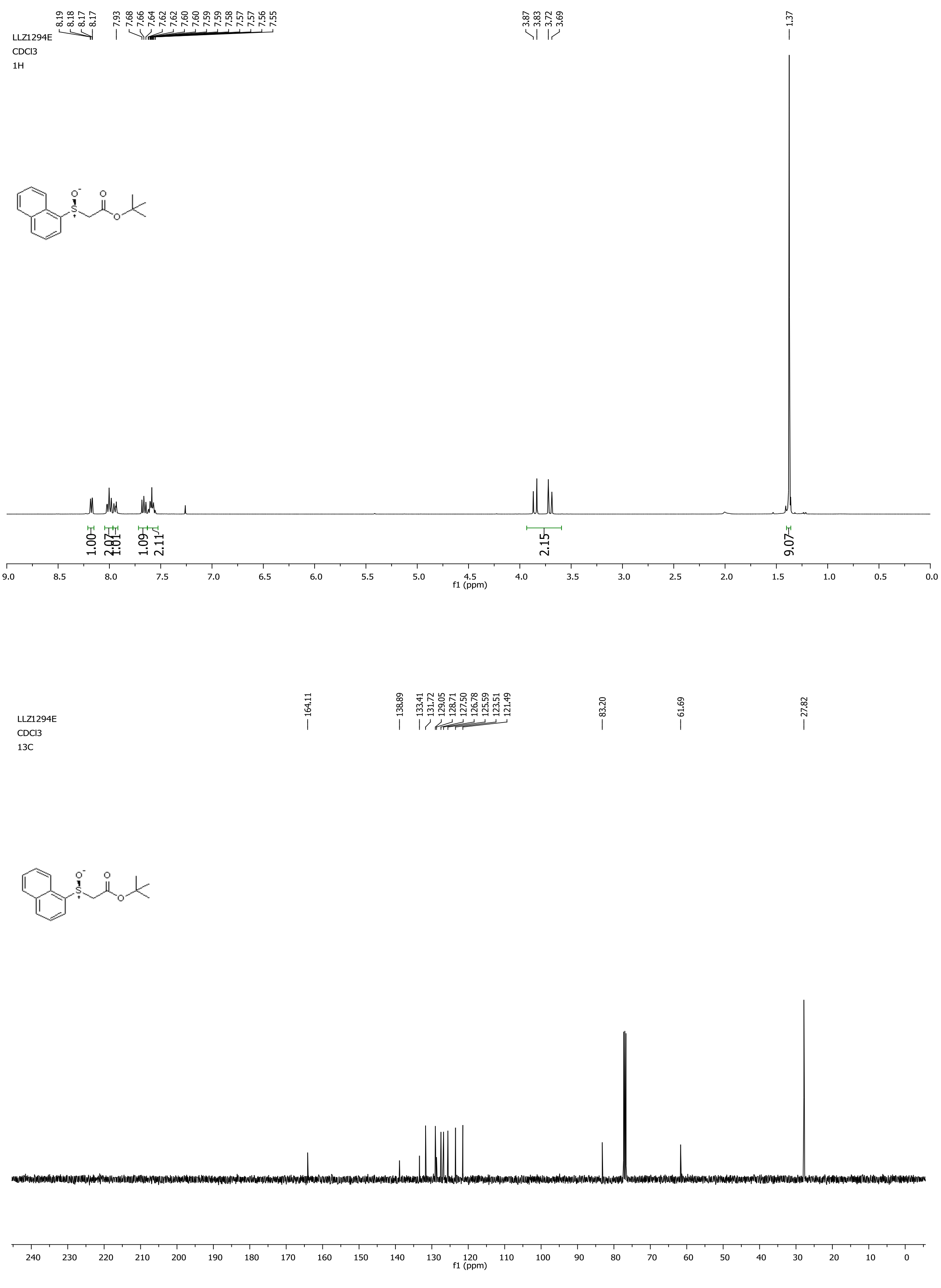


**Supplementary Figure 62.** $^{1}$H and $^{13}$C NMR spectra of sulfoxide **3q**.

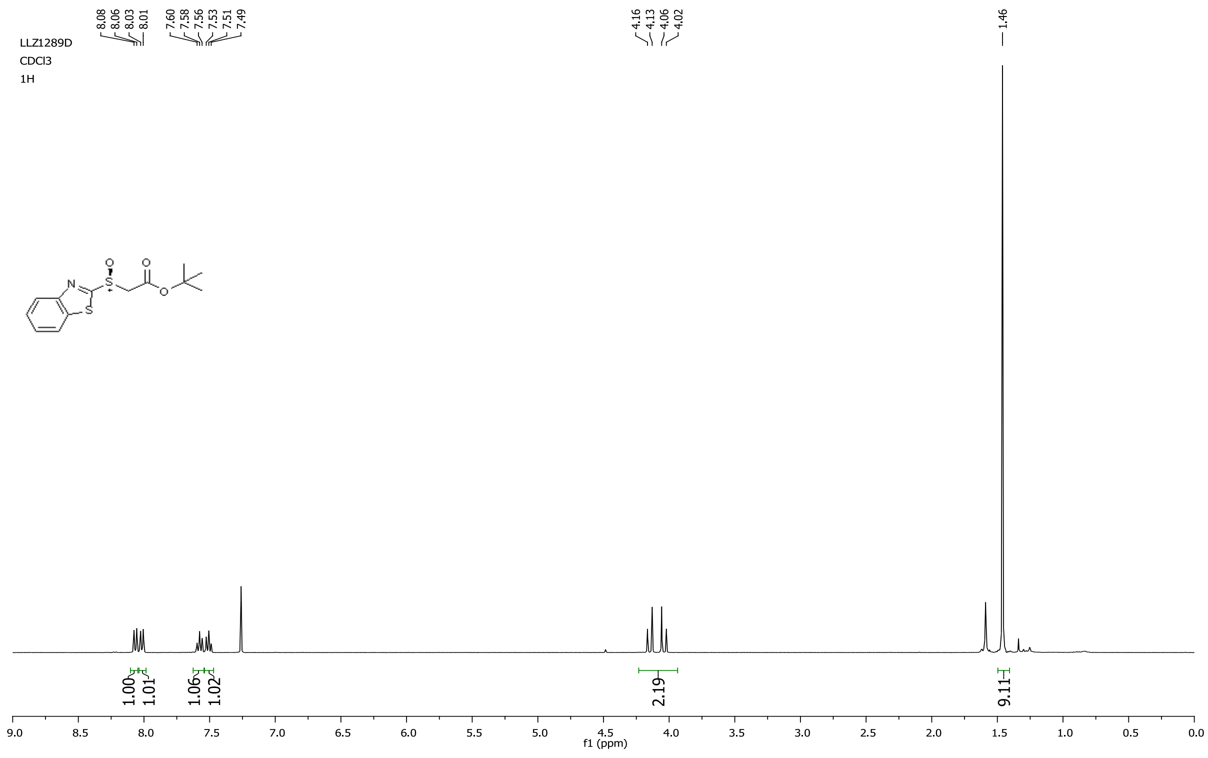


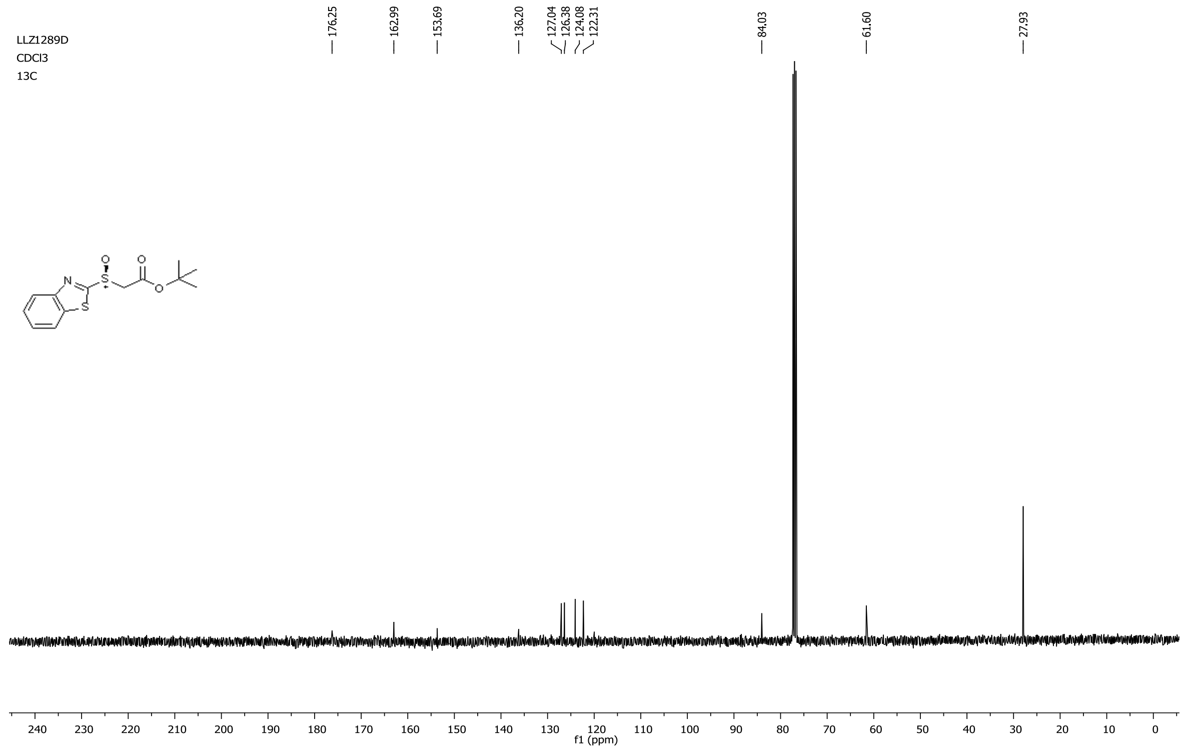


**Supplementary Figure 63.** $^{1}$H and $^{13}$C NMR spectra of sulfoxide **3r**.

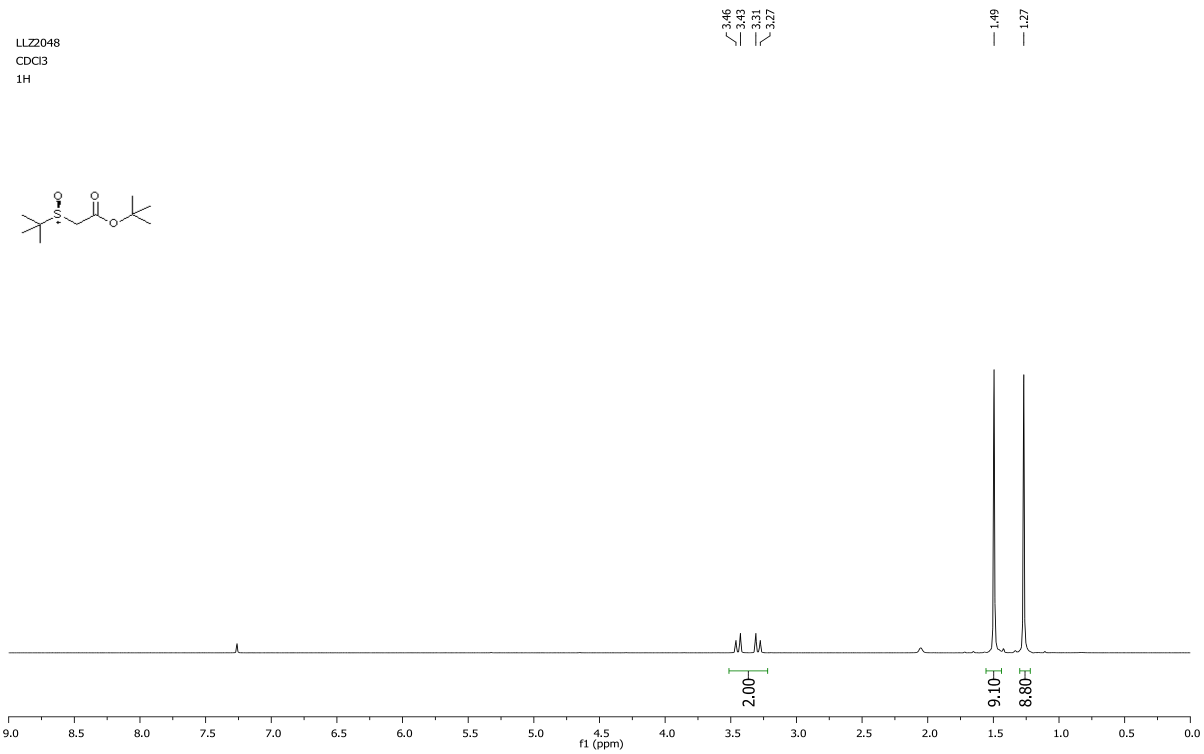


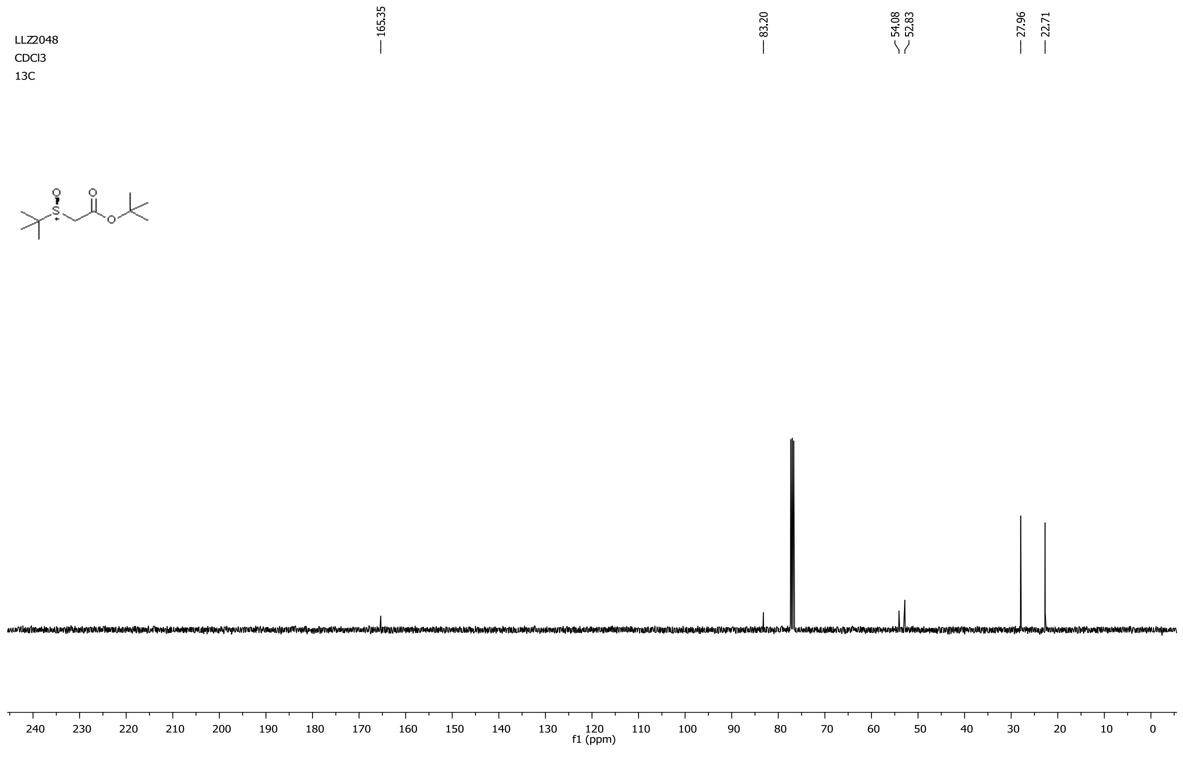


**Supplementary Figure 64.** $^{1}H$ and $^{13}C$ NMR spectra of sulfoxide **3s**.

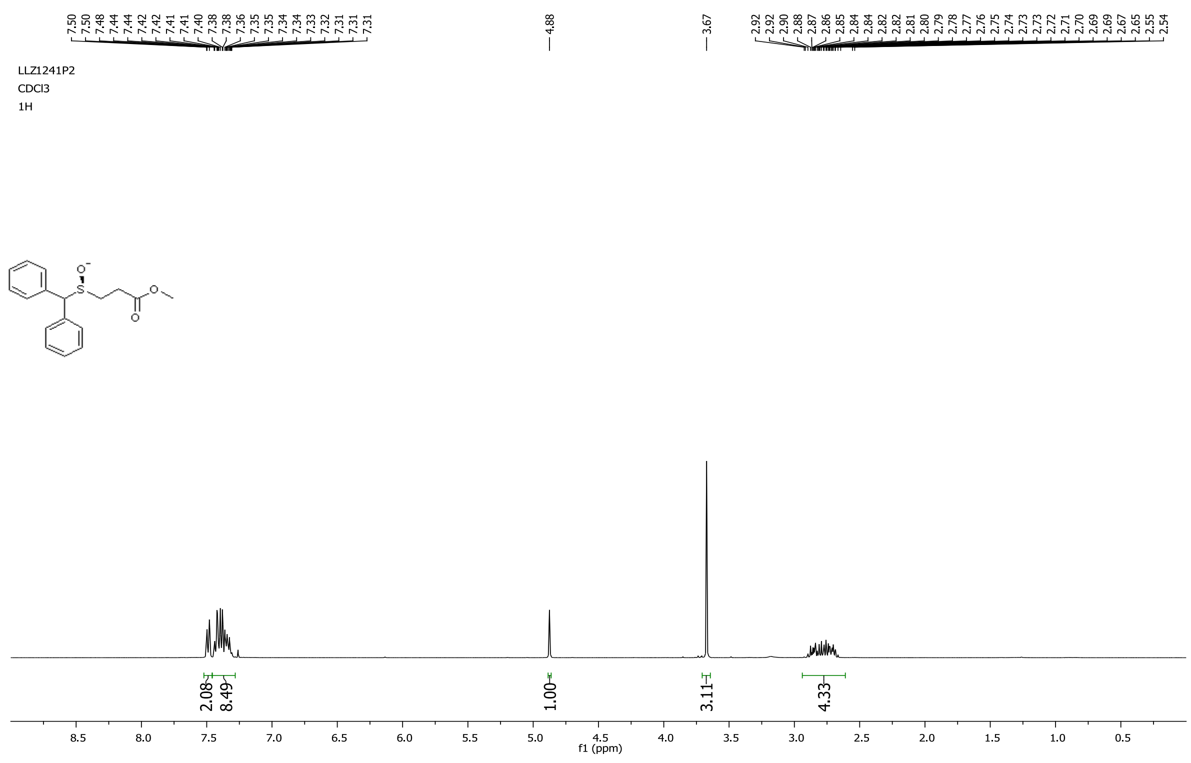


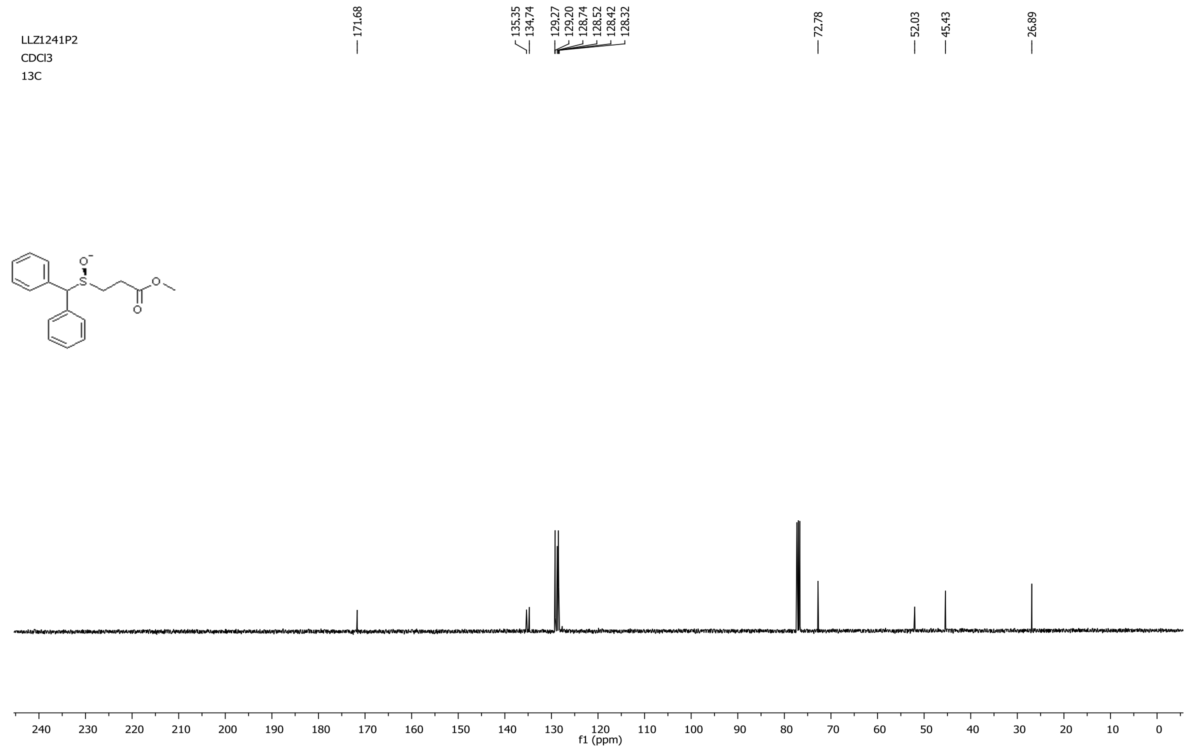


**Supplementary Figure 65.** $^{1}$H and $^{13}$C NMR spectra of sulfoxide **3t**.

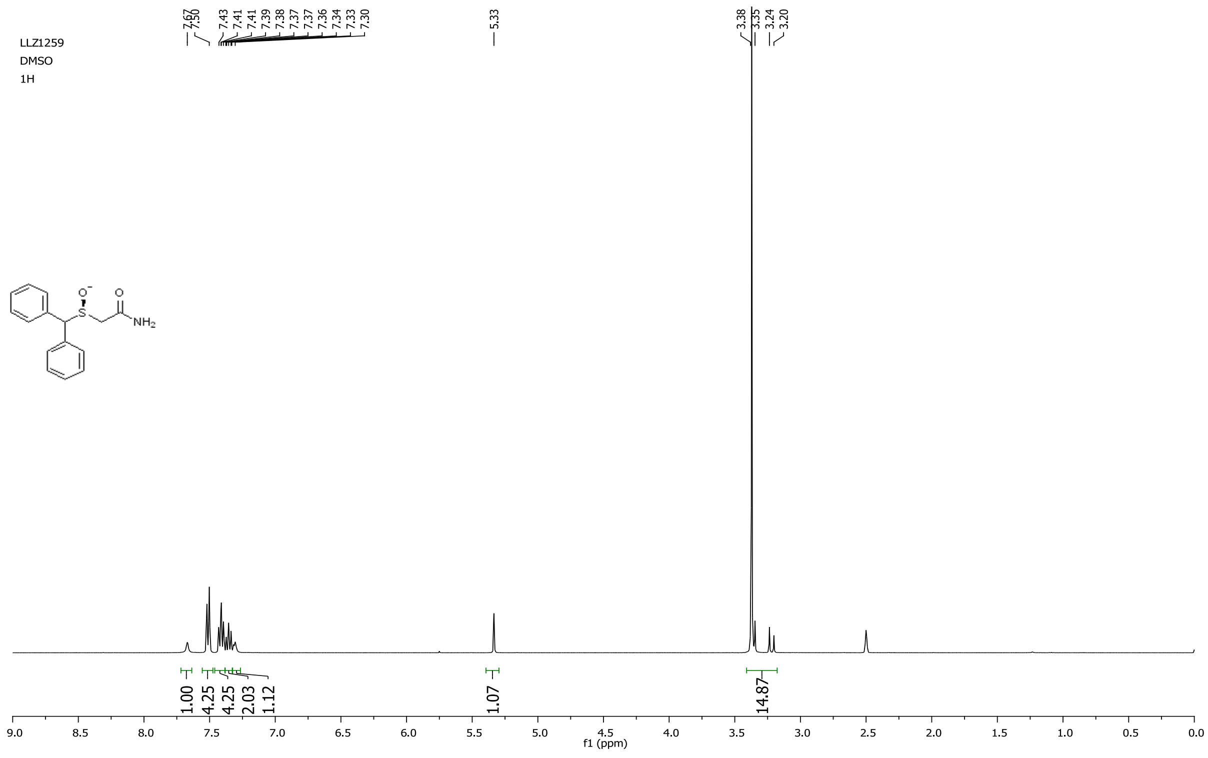


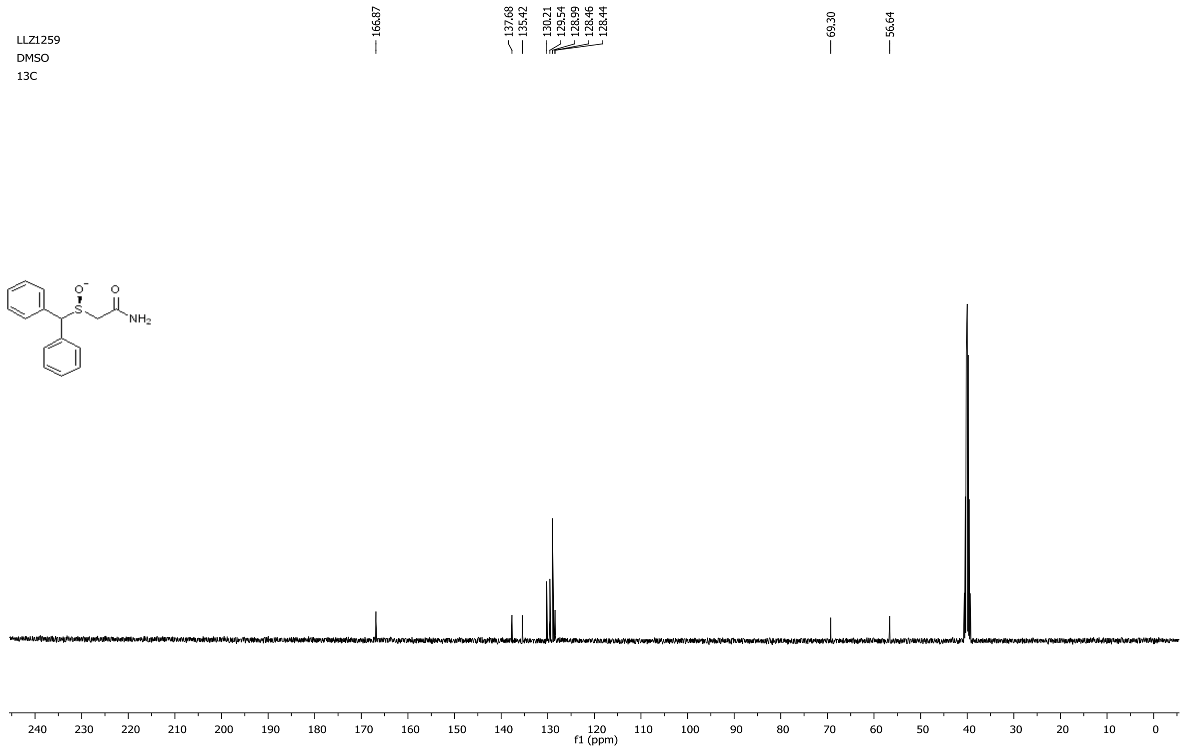


**Supplementary Figure 66.** $^{1}$H and $^{13}$C NMR spectra of sulfoxide **3u**.

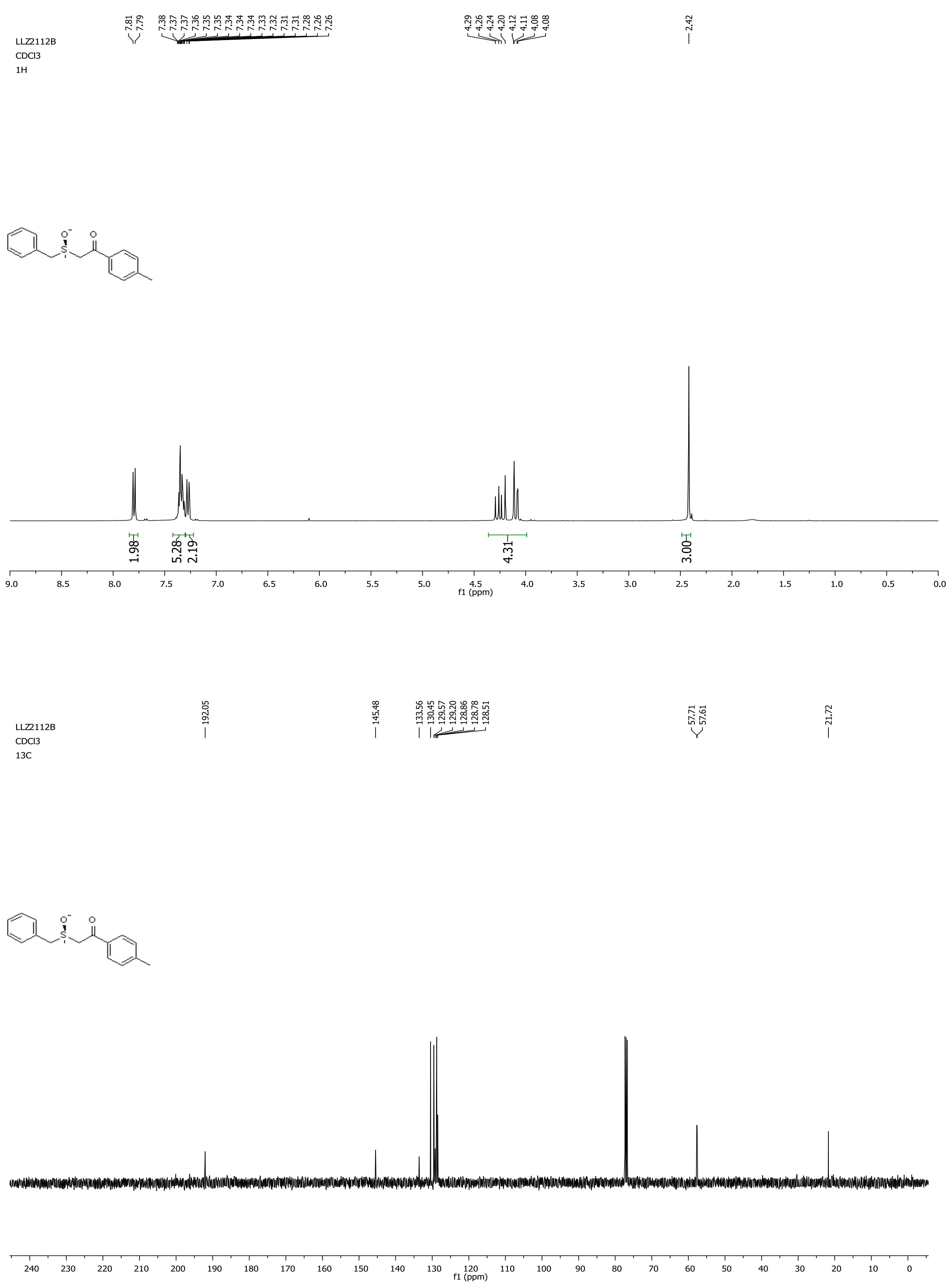


**Supplementary Figure 67.** $^{1}H$ and $^{13}C$ NMR spectra of sulfoxide **3v**.

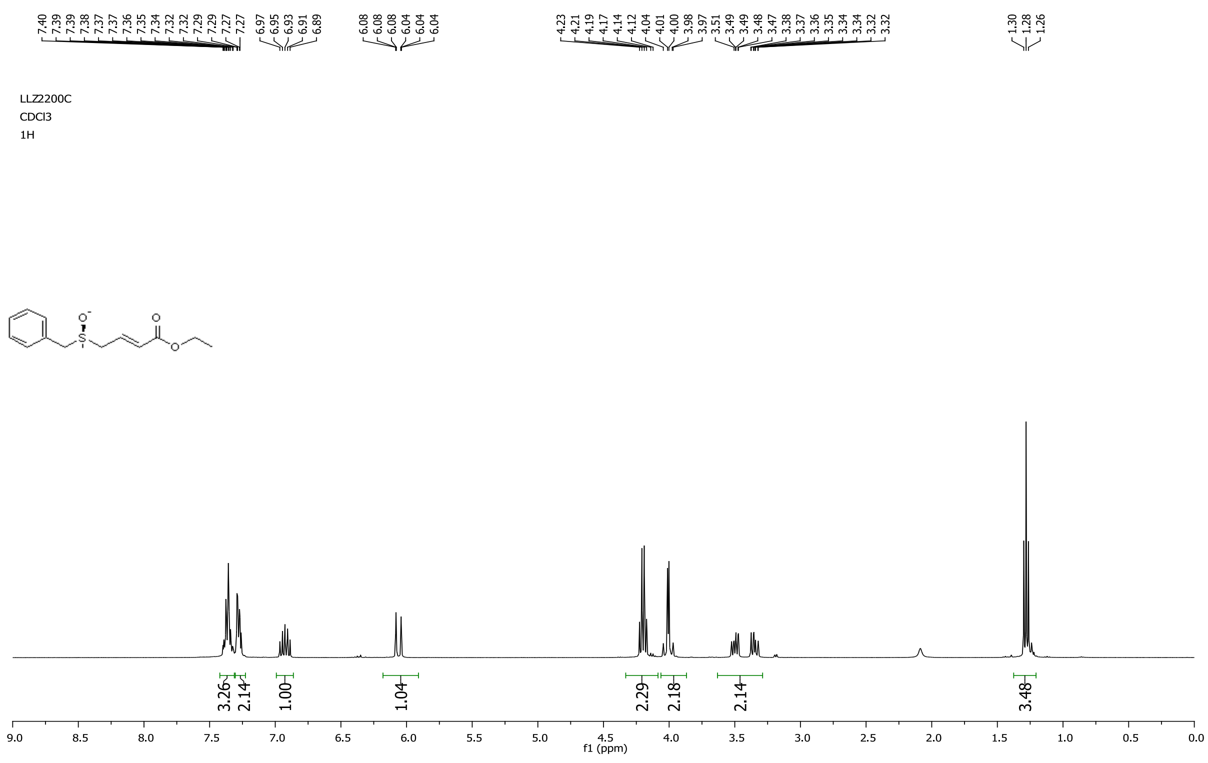

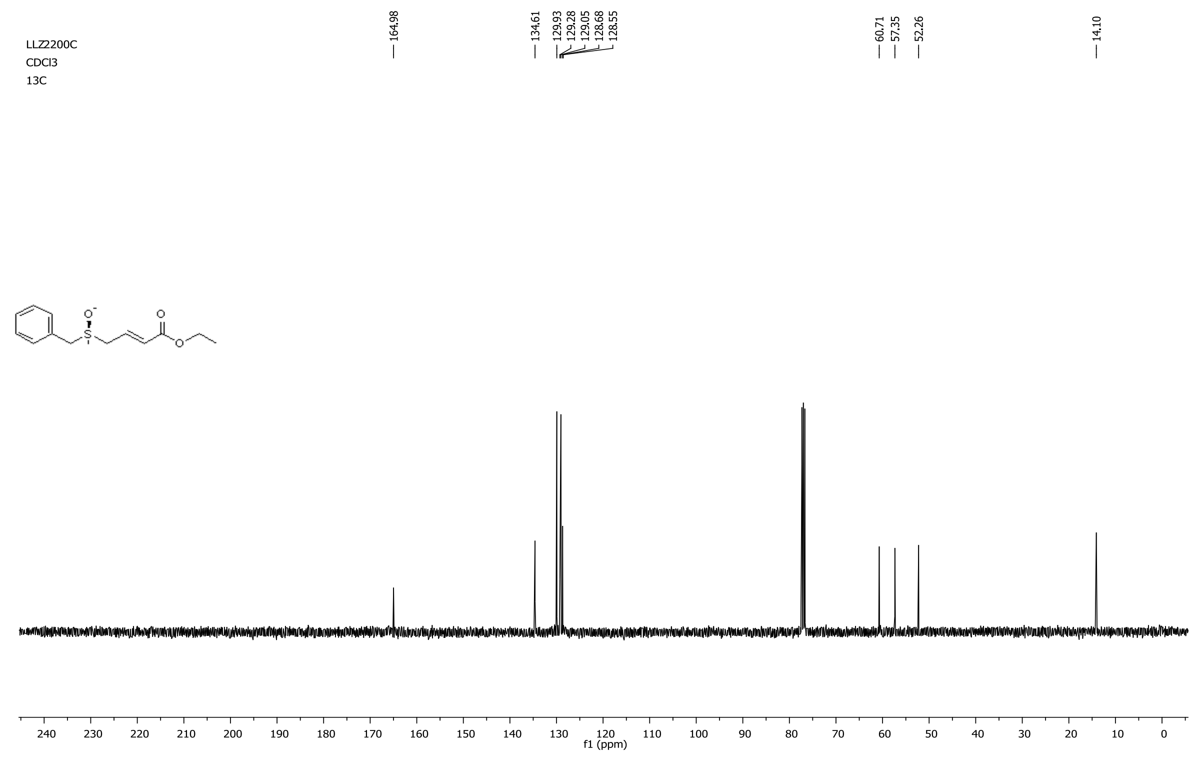

**Supplementary Figure 68.** $^{1}$H and $^{13}$C NMR spectra of sulfoxide **3w**.

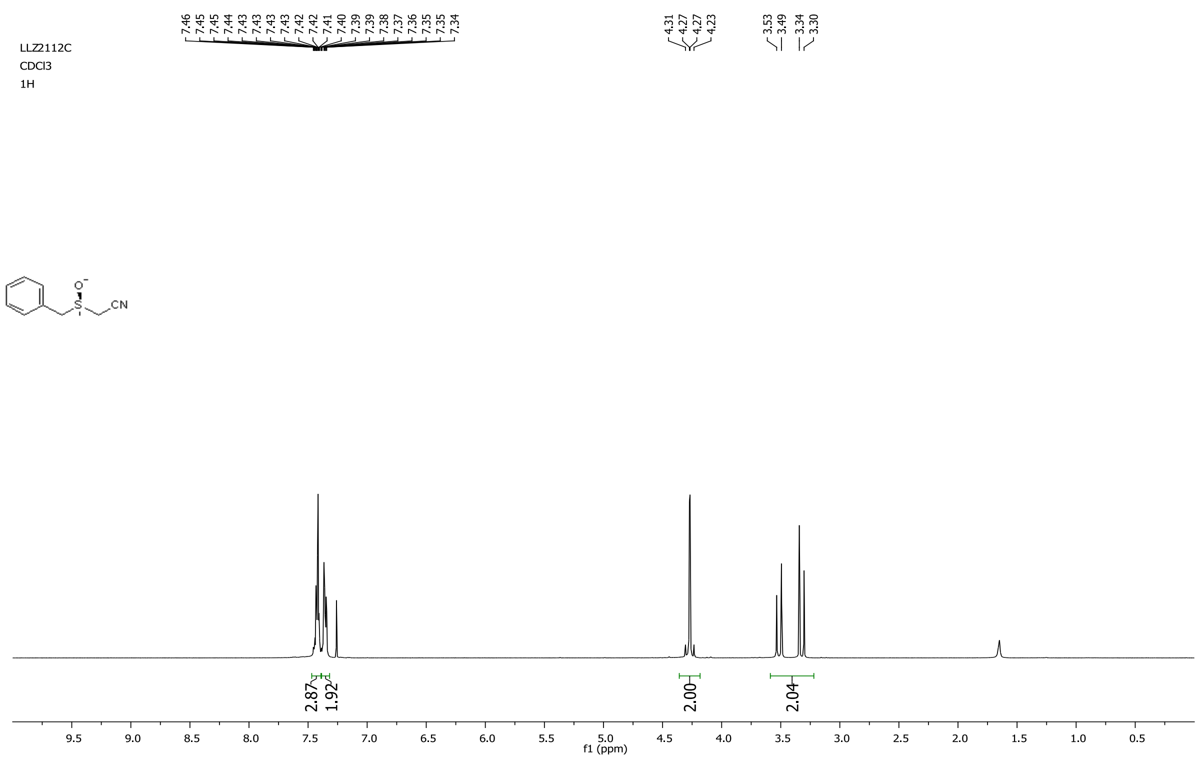


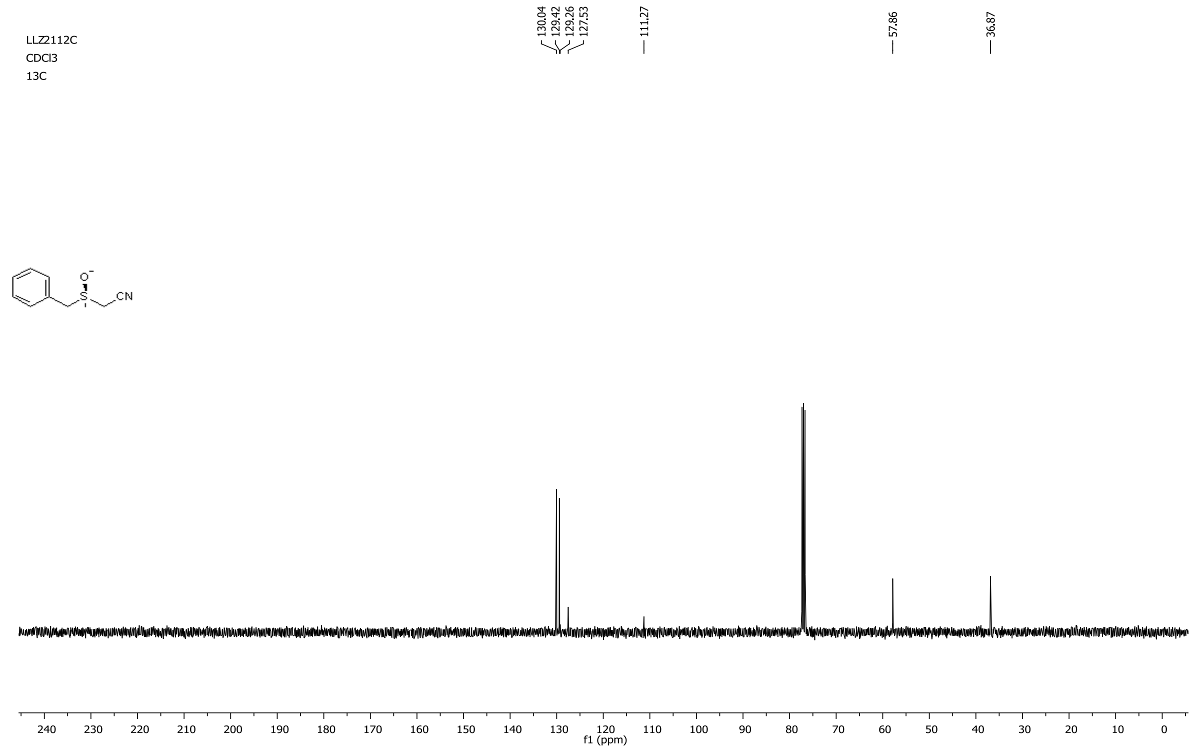


**Supplementary Figure 69.** $^{1}$H and $^{13}$C NMR spectra of sulfoxide **3x**.

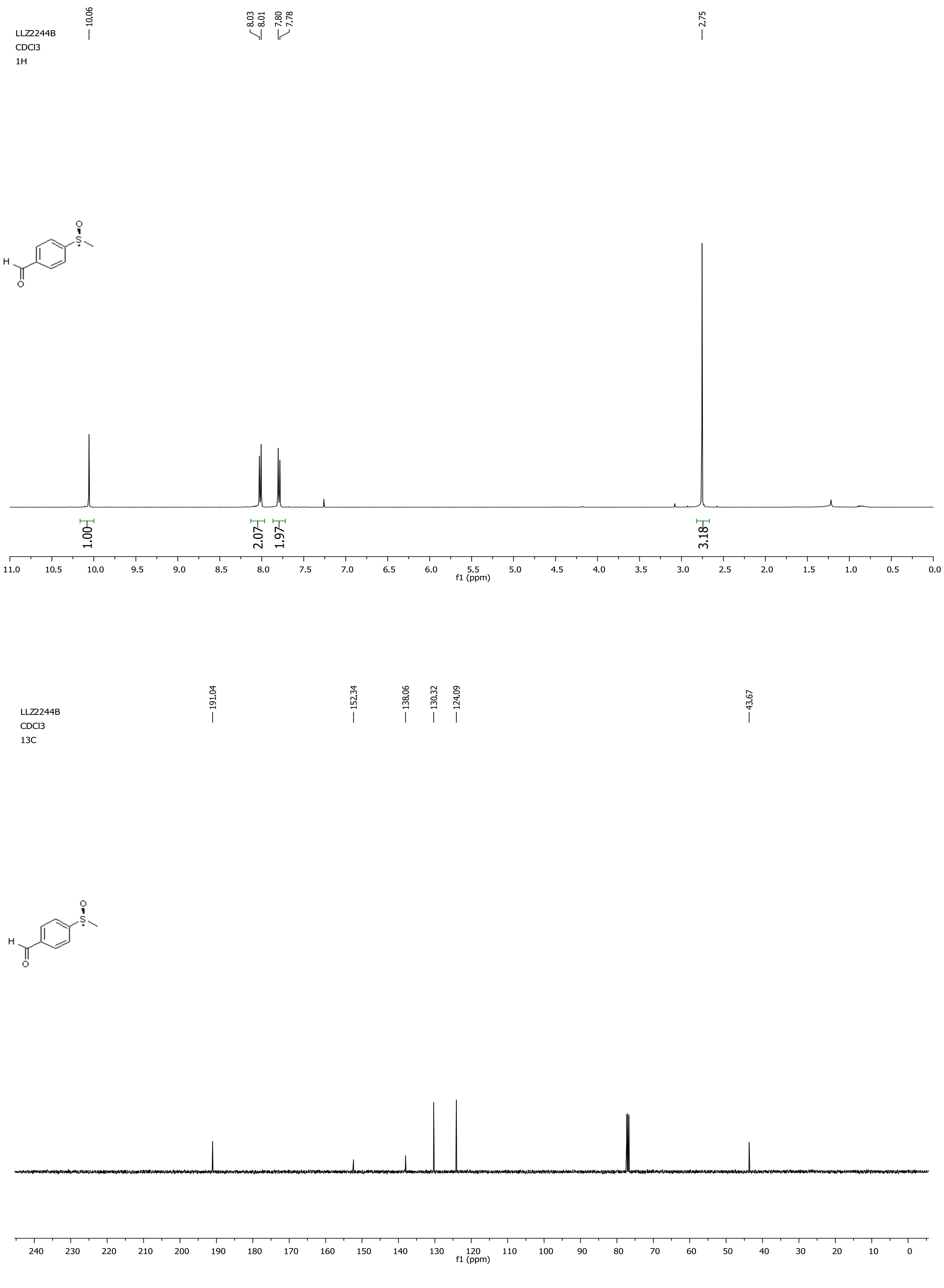


**Supplementary Figure 70.** $^{1}$H and $^{13}$C NMR spectra of sulfoxide **3y**.

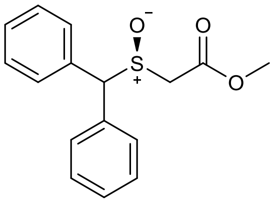

**3a**

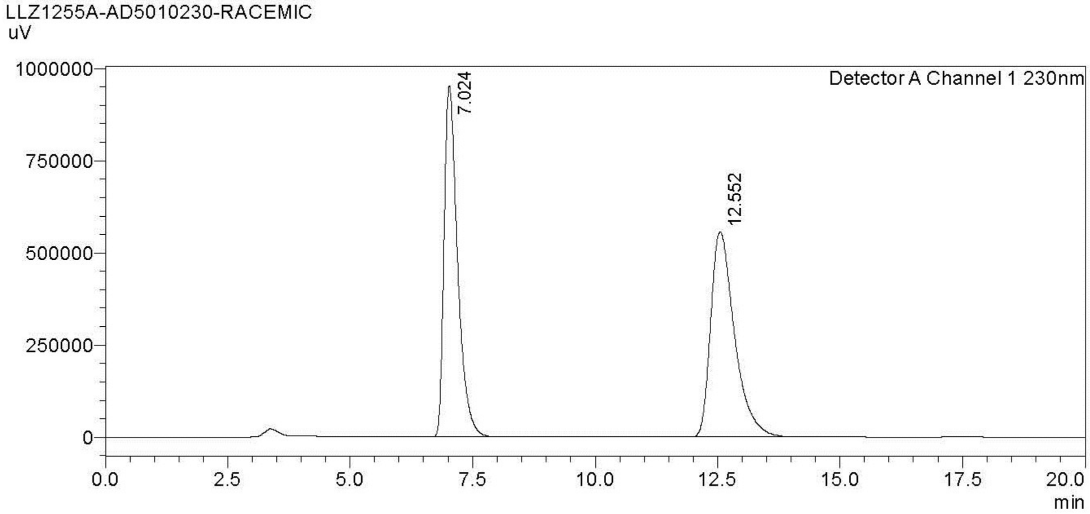


Detector A Channel 1 230nm

| Peak# | Ret. Time | Area | Height | Height% | Area% |
|---|---|---|---|---|---|
| 1 | 7.024 | 18271701 | 952483 | 63.090 | 49.401 |
| 2 | 12.552 | 18714833 | 557236 | 36.910 | 50.599 |
| Total | | 36986534 | 1509719 | 100.000 | 100.000 |

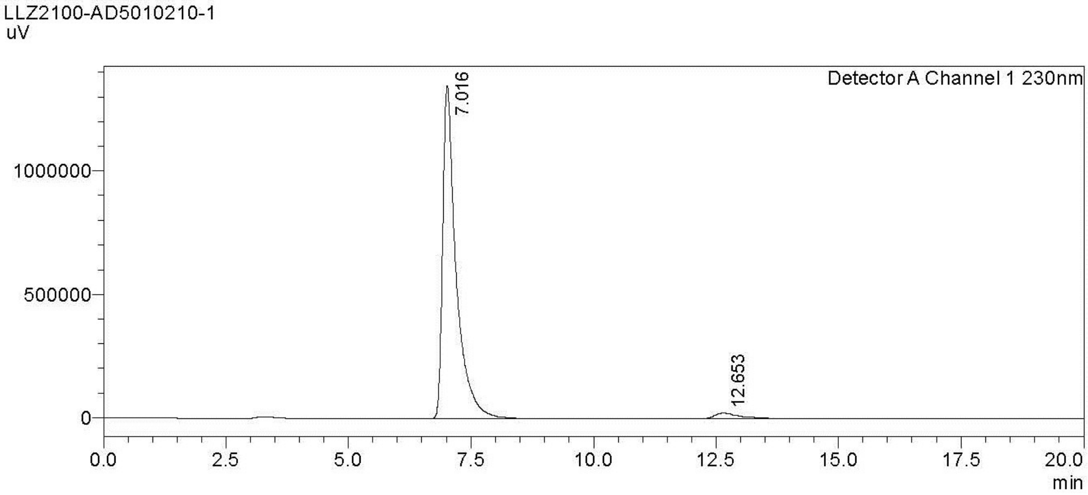


Detector A Channel 1 230nm

| Peak# | Ret. Time | Area | Height | Height% | Area% |
|---|---|---|---|---|---|
| 1 | 7.016 | 25549880 | 1347974 | 98.353 | 97.067 |
| 2 | 12.653 | 772010 | 22567 | 1.647 | 2.933 |
| Total | | 26321890 | 1370541 | 100.000 | 100.000 |

**Supplementary Figure 71.** HPLC spectra for **3a**.

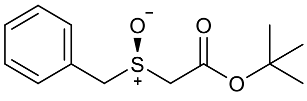

**3b**

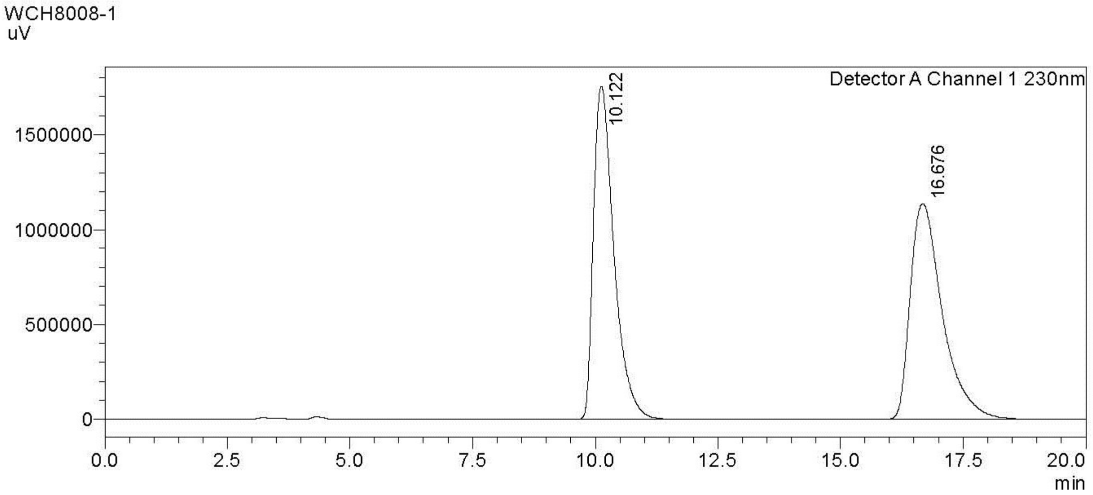


Detector A Channel 1 230nm

| Peak# | Ret. Time | Area | Height | Height% | Area% |
|---|---|---|---|---|---|
| 1 | 10.122 | 50547880 | 1755542 | 60.703 | 49.585 |
| 2 | 16.676 | 51394324 | 1136455 | 39.297 | 50.415 |
| Total | | 101942204 | 2891997 | 100.000 | 100.000 |

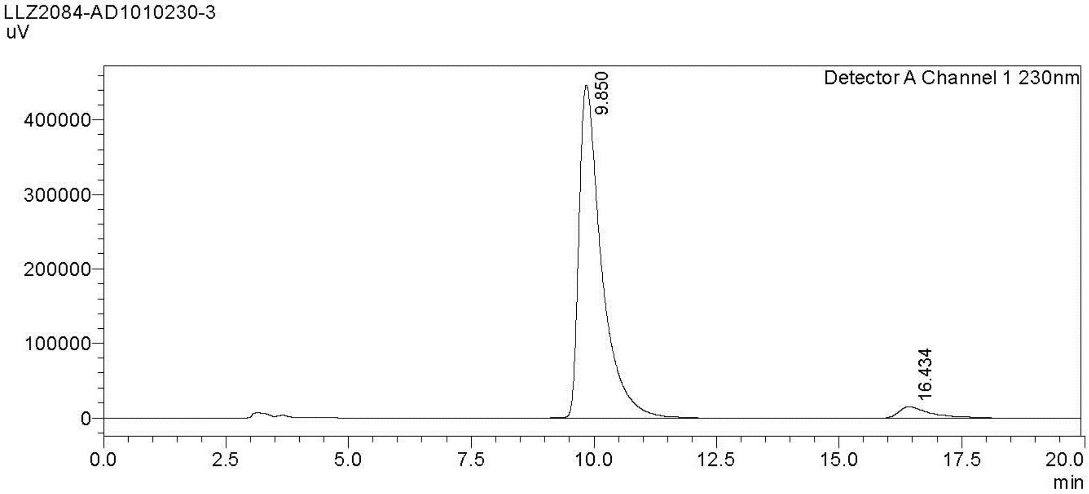


Detector A Channel 1 230nm

| Peak# | Ret. Time | Area | Height | Height% | Area% |
|---|---|---|---|---|---|
| 1 | 9.850 | 14244967 | 447503 | 96.713 | 95.333 |
| 2 | 16.434 | 697347 | 15209 | 3.287 | 4.667 |
| Total | | 14942314 | 462712 | 100.000 | 100.000 |

**Supplementary Figure 72.** HPLC spectra for **3b**.

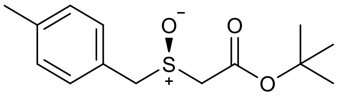

**3c**

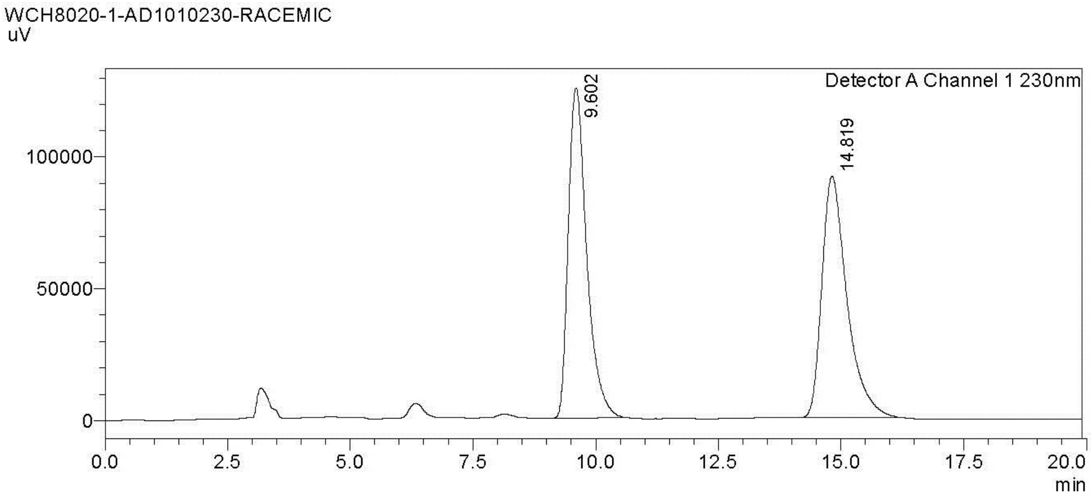


Detector A Channel 1 230nm

| Peak# | Ret. Time | Area | Height | Height% | Area% |
|---|---|---|---|---|---|
| 1 | 9.602 | 3270197 | 125431 | 57.833 | 50.103 |
| 2 | 14.819 | 3256707 | 91454 | 42.167 | 49.897 |
| Total | | 6526904 | 216885 | 100.000 | 100.000 |

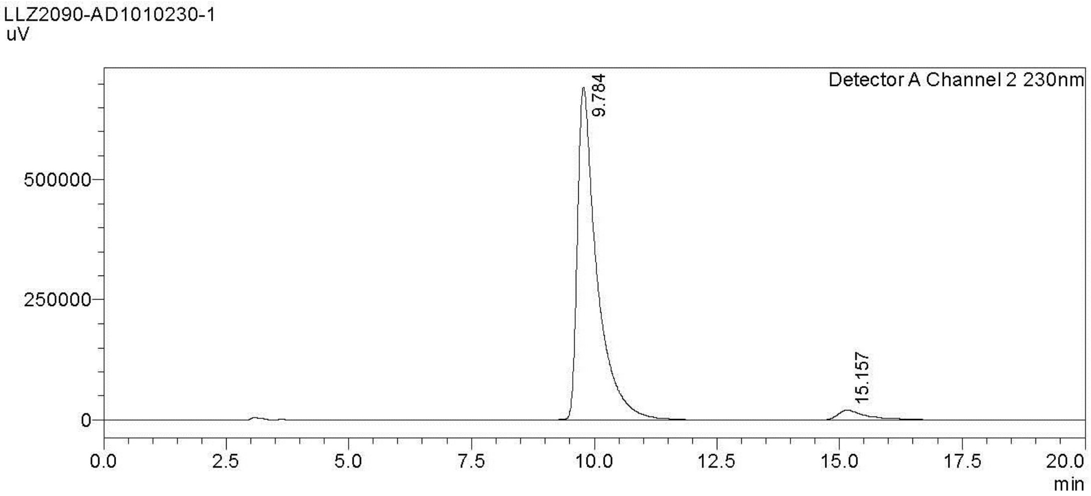


Detector A Channel 2 230nm

| Peak# | Ret. Time | Area | Height | Height% | Area% |
|---|---|---|---|---|---|
| 1 | 9.784 | 18976527 | 692825 | 97.210 | 96.042 |
| 2 | 15.157 | 782028 | 19884 | 2.790 | 3.958 |
| Total | | 19758554 | 712710 | 100.000 | 100.000 |

**Supplementary Figure 73.** HPLC spectra for **3c**.

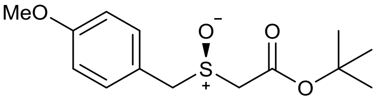

3d

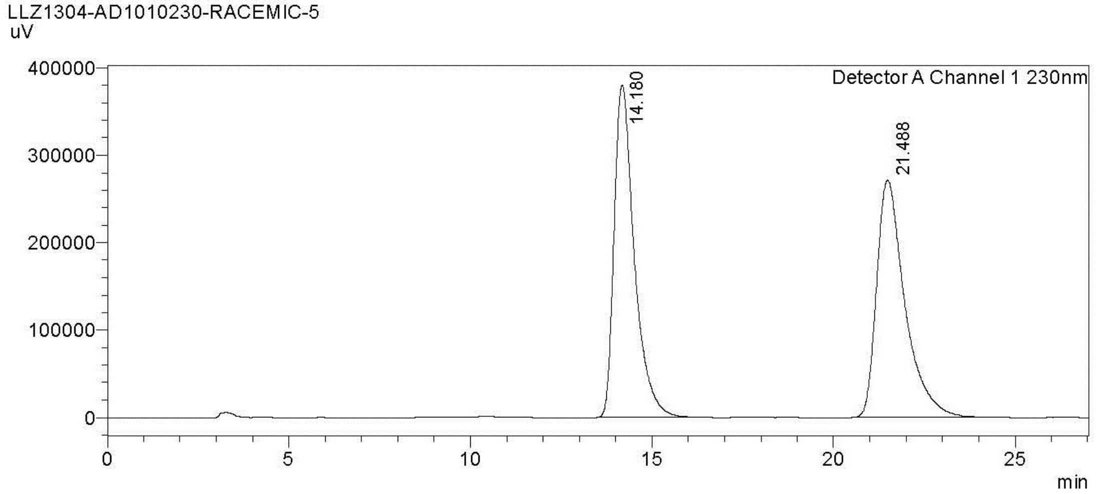


Detector A Channel 1 230nm

| Peak# | Ret. Time | Area | Height | Height% | Area% |
|---|---|---|---|---|---|
| 1 | 14.180 | 14631746 | 380618 | 58.406 | 50.084 |
| 2 | 21.488 | 14582705 | 271061 | 41.594 | 49.916 |
| Total | | 29214451 | 651680 | 100.000 | 100.000 |

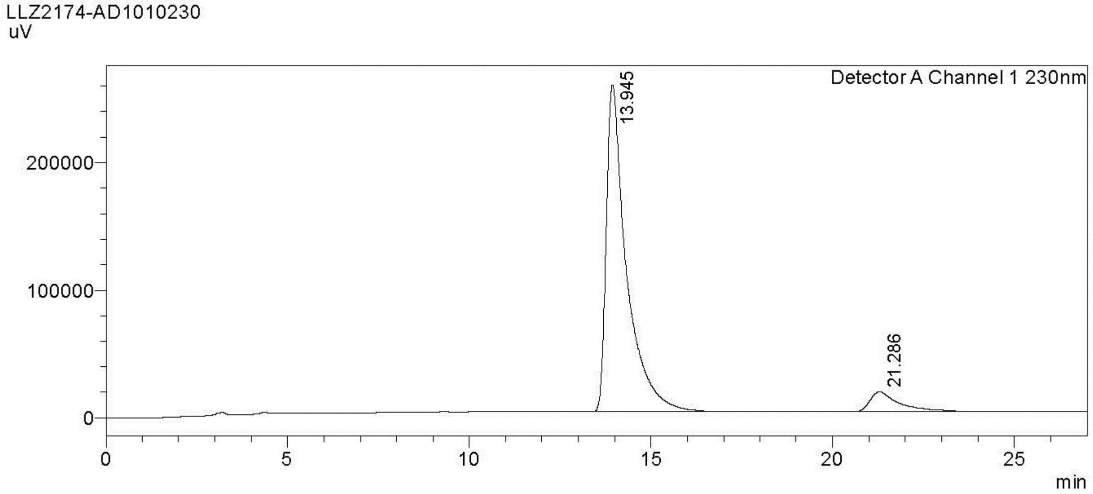


Detector A Channel 1 230nm

| Peak# | Ret. Time | Area | Height | Height% | Area% |
|---|---|---|---|---|---|
| 1 | 13.945 | 9909206 | 256415 | 94.283 | 91.771 |
| 2 | 21.286 | 888585 | 15548 | 5.717 | 8.229 |
| Total | | 10797792 | 271963 | 100.000 | 100.000 |

**Supplementary Figure 74.** HPLC spectra for **3d**.

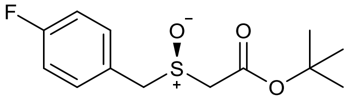

3e

LLZ1304-AD1010230-RACEMIC-4

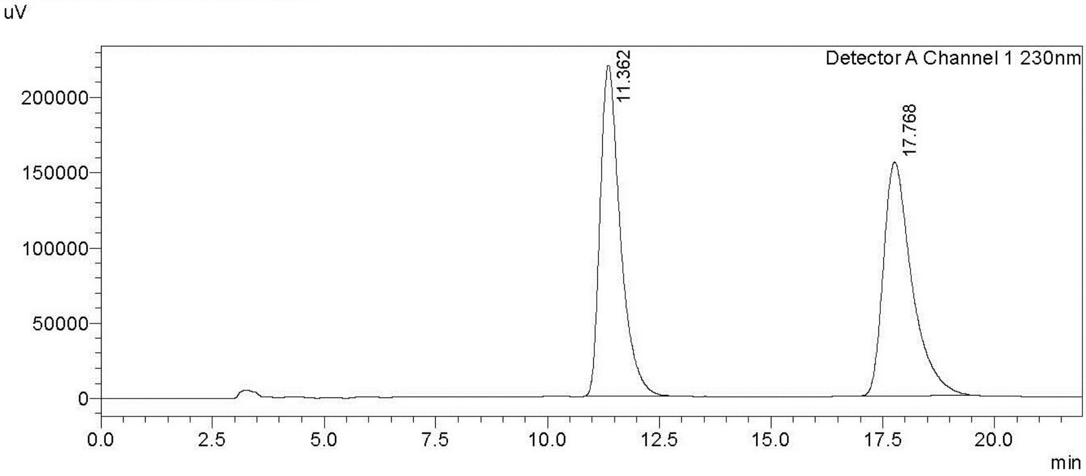


Detector A Channel 1 230nm

| Peak# | Ret. Time | Area | Height | Height% | Area% |
|---|---|---|---|---|---|
| 1 | 11.362 | 6914783 | 219969 | 58.623 | 50.102 |
| 2 | 17.768 | 6886713 | 155259 | 41.377 | 49.898 |
| Total | | 13801496 | 375228 | 100.000 | 100.000 |

LLZ2092-AD1010210--1

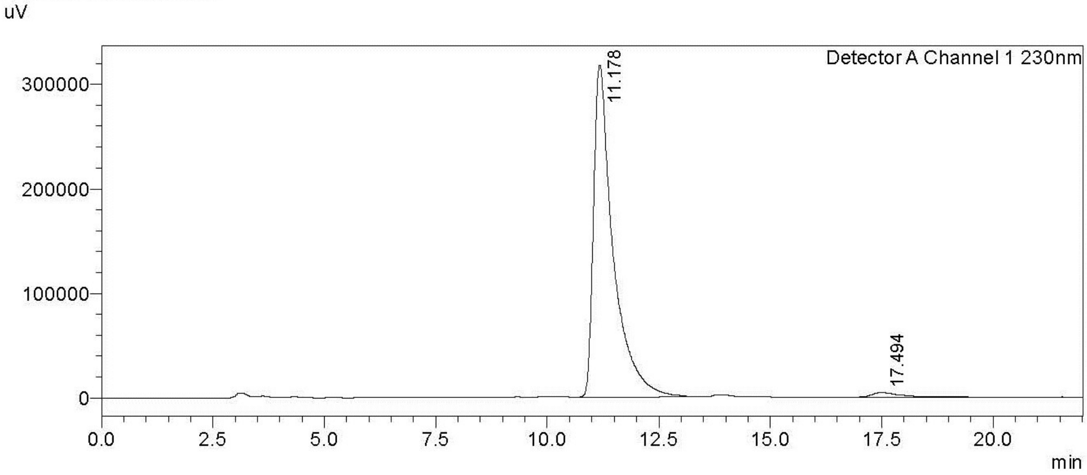


Detector A Channel 1 230nm

| Peak# | Ret. Time | Area | Height | Height% | Area% |
|---|---|---|---|---|---|
| 1 | 11.178 | 9696600 | 317425 | 98.567 | 97.916 |
| 2 | 17.494 | 206358 | 4616 | 1.433 | 2.084 |
| Total | | 9902958 | 322041 | 100.000 | 100.000 |

**Supplementary Figure 75.** HPLC spectra for **3e**.

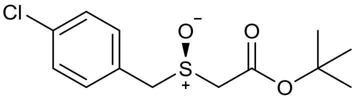

**3f**

WCH8020-2-AD1010230-RACEMIC

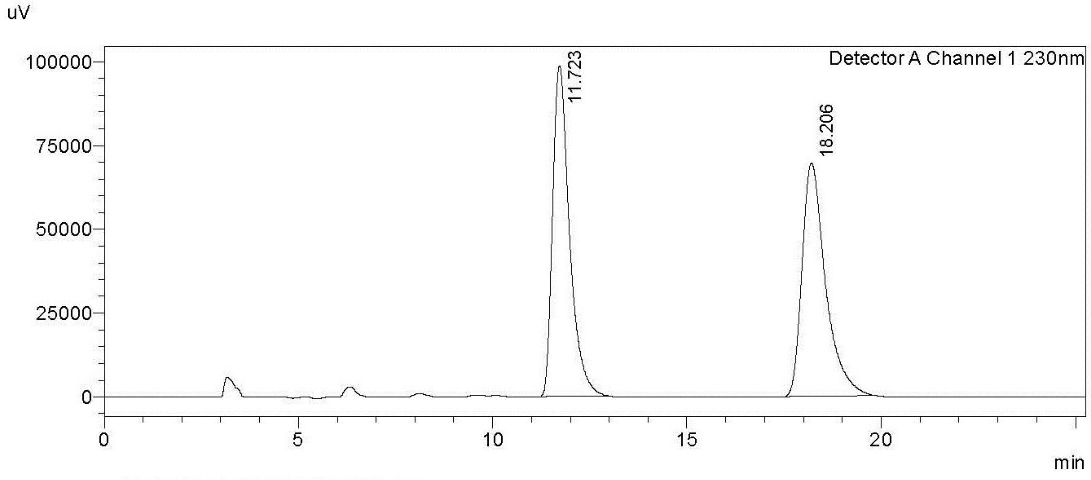


Detector A Channel 1 230nm

| Peak# | Ret. Time | Area | Height | Height% | Area% |
|---|---|---|---|---|---|
| 1 | 11.723 | 2962062 | 98777 | 58.668 | 50.350 |
| 2 | 18.206 | 2920850 | 69589 | 41.332 | 49.650 |
| Total | | 5882912 | 168366 | 100.000 | 100.000 |

LLZ2092-AD1010210--2

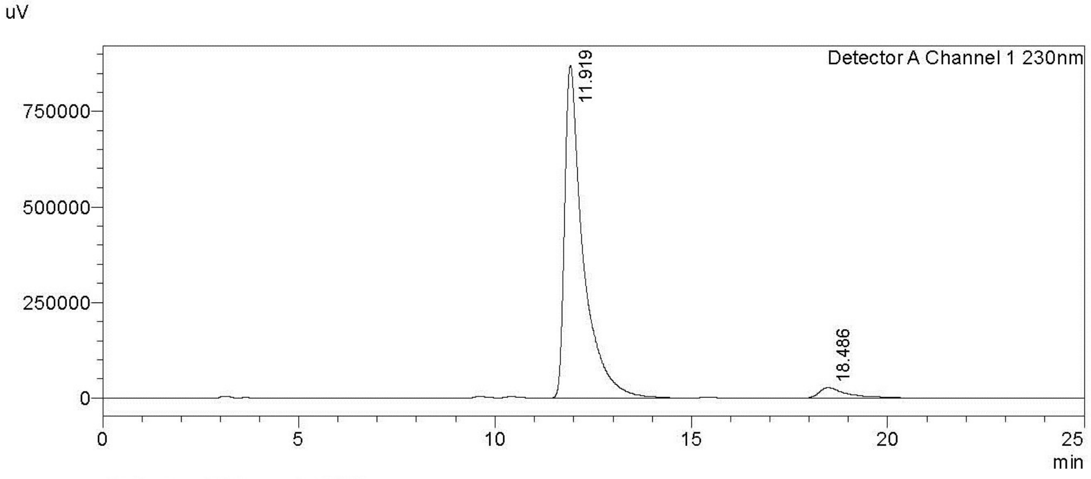


Detector A Channel 1 230nm

| Peak# | Ret. Time | Area | Height | Height% | Area% |
|---|---|---|---|---|---|
| 1 | 11.919 | 28507932 | 871329 | 96.982 | 95.610 |
| 2 | 18.486 | 1309013 | 27118 | 3.018 | 4.390 |
| Total | | 29816946 | 898447 | 100.000 | 100.000 |

**Supplementary Figure 76.** HPLC spectra for **3f**.

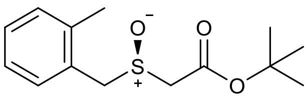

**3g**

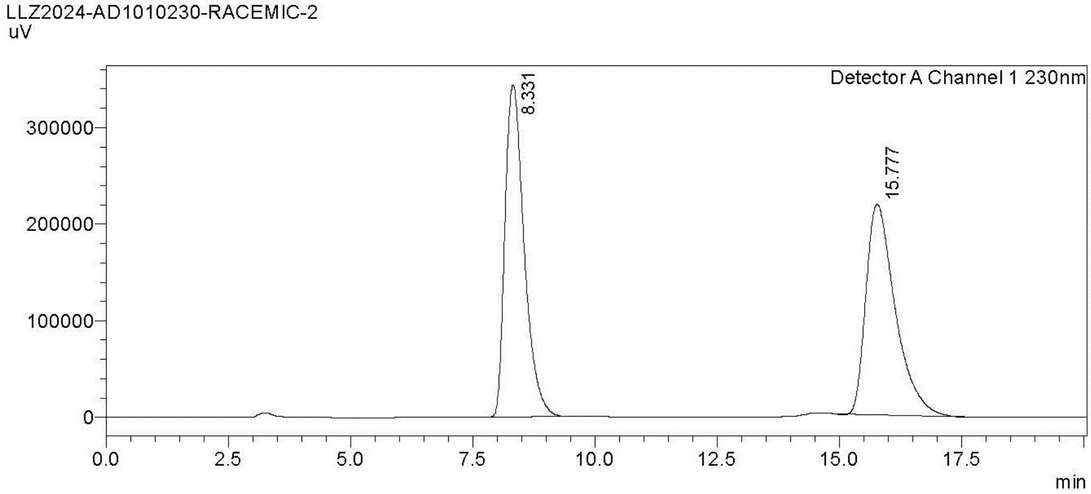


Detector A Channel 1 230nm

| Peak# | Ret. Time | Area | Height | Height% | Area% |
|---|---|---|---|---|---|
| 1 | 8.331 | 9381779 | 344181 | 61.215 | 50.767 |
| 2 | 15.777 | 9098186 | 218069 | 38.785 | 49.233 |
| Total | | 18479965 | 562250 | 100.000 | 100.000 |

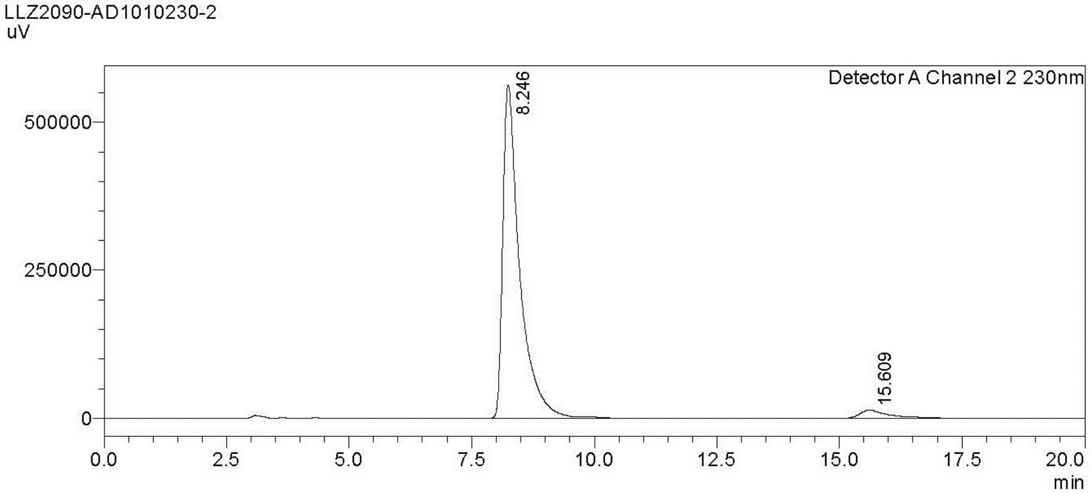


Detector A Channel 2 230nm

| Peak# | Ret. Time | Area | Height | Height% | Area% |
|---|---|---|---|---|---|
| 1 | 8.246 | 13508447 | 563284 | 97.693 | 96.137 |
| 2 | 15.609 | 542855 | 13302 | 2.307 | 3.863 |
| Total | | 14051302 | 576585 | 100.000 | 100.000 |

**Supplementary Figure 77.** HPLC spectra for **3g**.

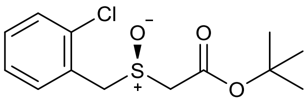

**3h**

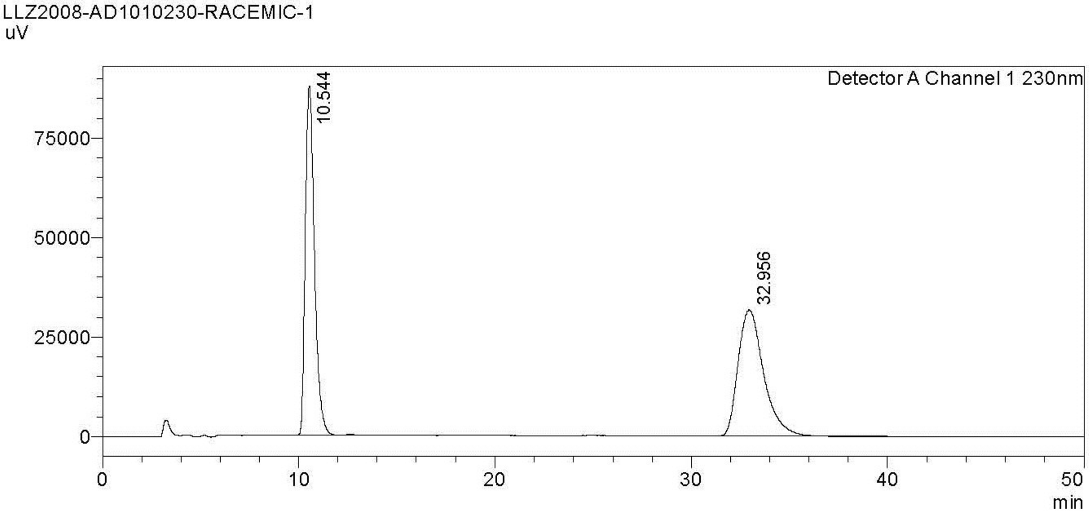


Detector A Channel 1 230nm

| Peak# | Ret. Time | Area | Height | Height% | Area% |
|---|---|---|---|---|---|
| 1 | 10.544 | 2874941 | 87807 | 73.473 | 50.081 |
| 2 | 32.956 | 2865618 | 31702 | 26.527 | 49.919 |
| Total | | 5740560 | 119509 | 100.000 | 100.000 |

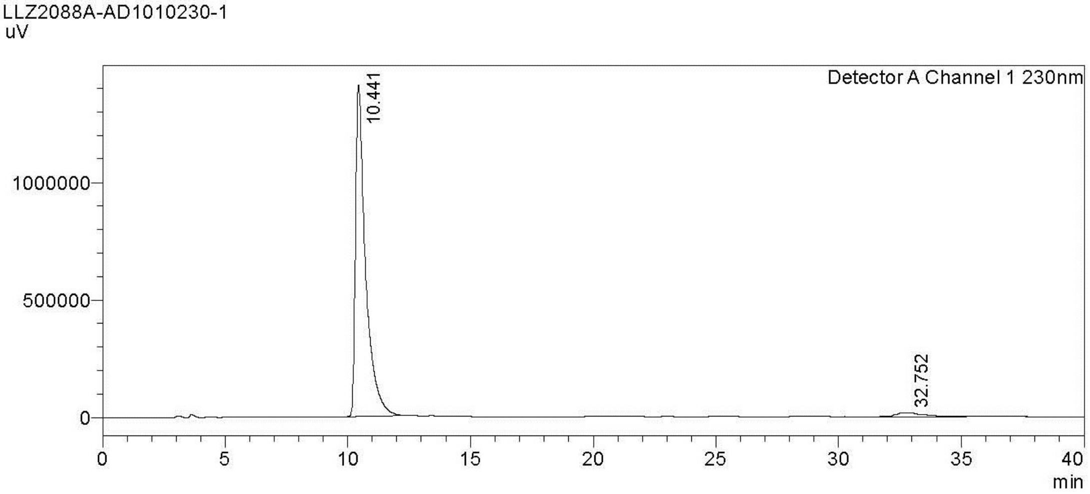


Detector A Channel 1 230nm

| Peak# | Ret. Time | Area | Height | Height% | Area% |
|---|---|---|---|---|---|
| 1 | 10.441 | 40176179 | 1411036 | 98.755 | 96.432 |
| 2 | 32.752 | 1486339 | 17783 | 1.245 | 3.568 |
| Total | | 41662518 | 1428819 | 100.000 | 100.000 |

**Supplementary Figure 78.** HPLC spectra for **3h**.

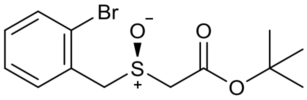

**3i**

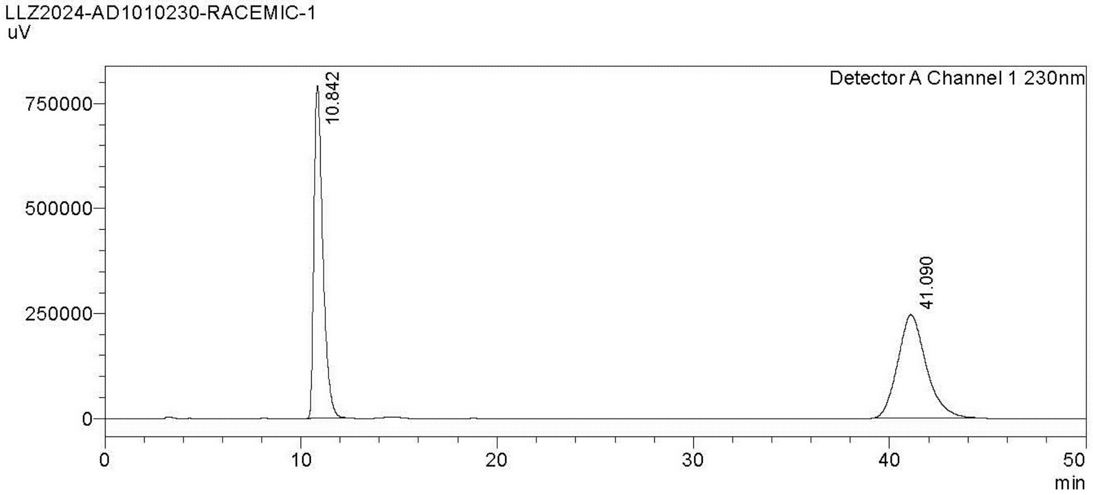


Detector A Channel 1 230nm

| Peak# | Ret. Time | Area | Height | Height% | Area% |
|---|---|---|---|---|---|
| 1 | 10.842 | 24954185 | 792661 | 76.323 | 50.167 |
| 2 | 41.090 | 24788157 | 245894 | 23.677 | 49.833 |
| Total | | 49742343 | 1038555 | 100.000 | 100.000 |

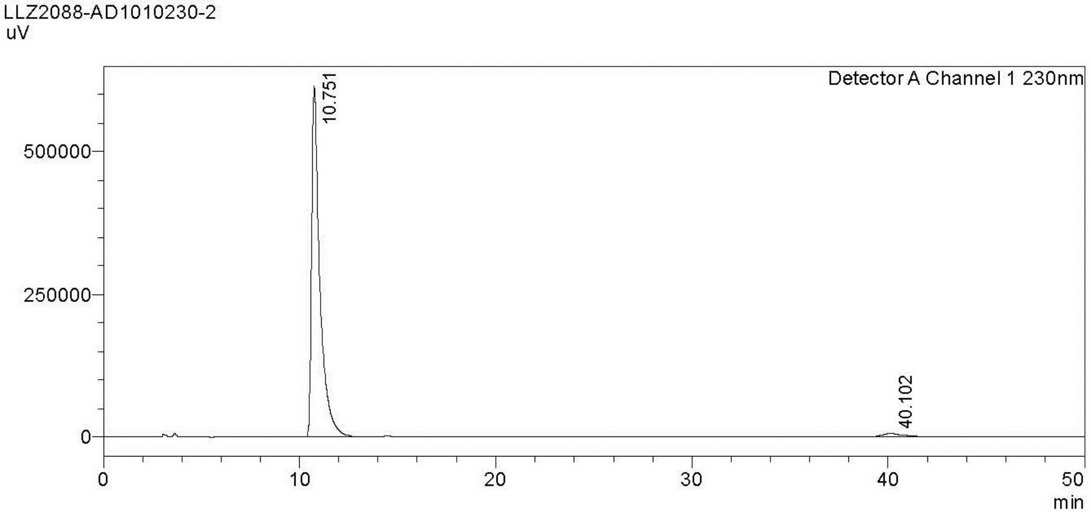


Detector A Channel 1 230nm

| Peak# | Ret. Time | Area | Height | Height% | Area% |
|---|---|---|---|---|---|
| 1 | 10.751 | 17925966 | 614842 | 99.050 | 96.702 |
| 2 | 40.102 | 611294 | 5895 | 0.950 | 3.298 |
| Total | | 18537260 | 620737 | 100.000 | 100.000 |

**Supplementary Figure 79.** HPLC spectra for **3i**.

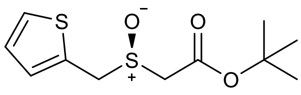


**3j**

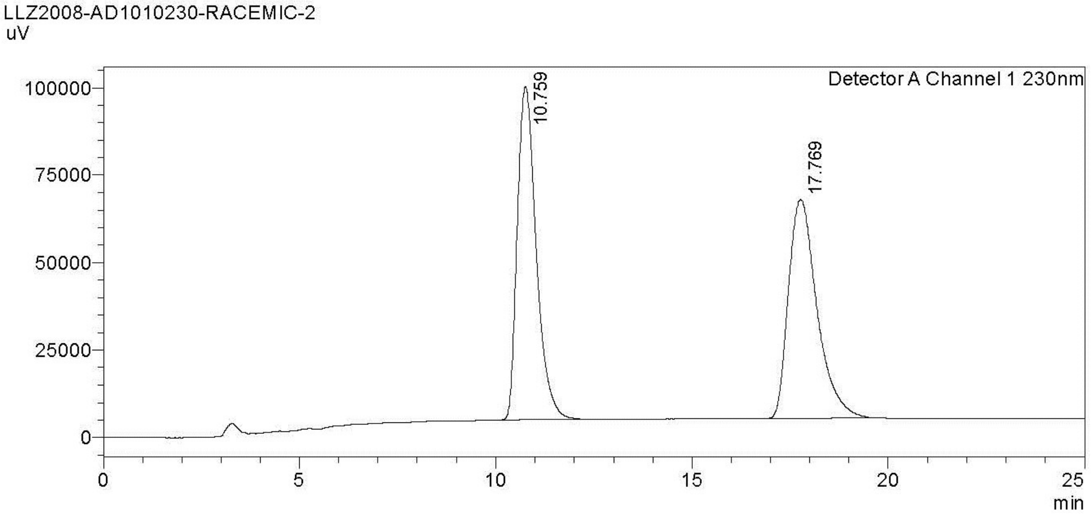


Detector A Channel 1 230nm

| Peak# | Ret. Time | Area | Height | Height% | Area% |
|---|---|---|---|---|---|
| 1 | 10.759 | 3184041 | 95278 | 60.368 | 50.114 |
| 2 | 17.769 | 3169538 | 62550 | 39.632 | 49.886 |
| Total | | 6353579 | 157828 | 100.000 | 100.000 |

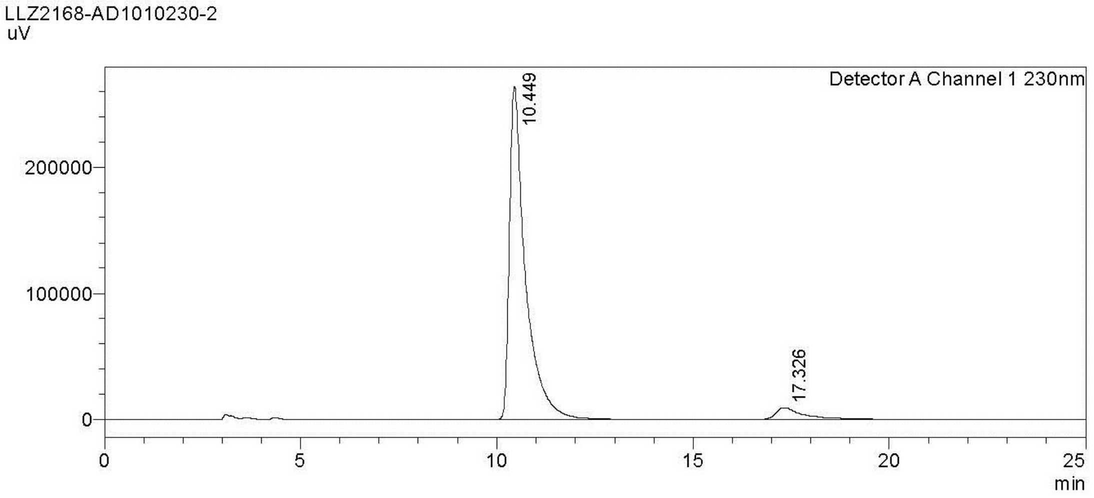


Detector A Channel 1 230nm

| Peak# | Ret. Time | Area | Height | Height% | Area% |
|---|---|---|---|---|---|
| 1 | 10.449 | 7441648 | 264152 | 96.643 | 94.637 |
| 2 | 17.326 | 421703 | 9176 | 3.357 | 5.363 |
| Total | | 7863351 | 273328 | 100.000 | 100.000 |

**Supplementary Figure 80.** HPLC spectra for **3j**.

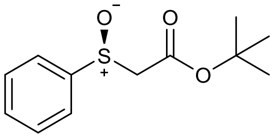

**3k**

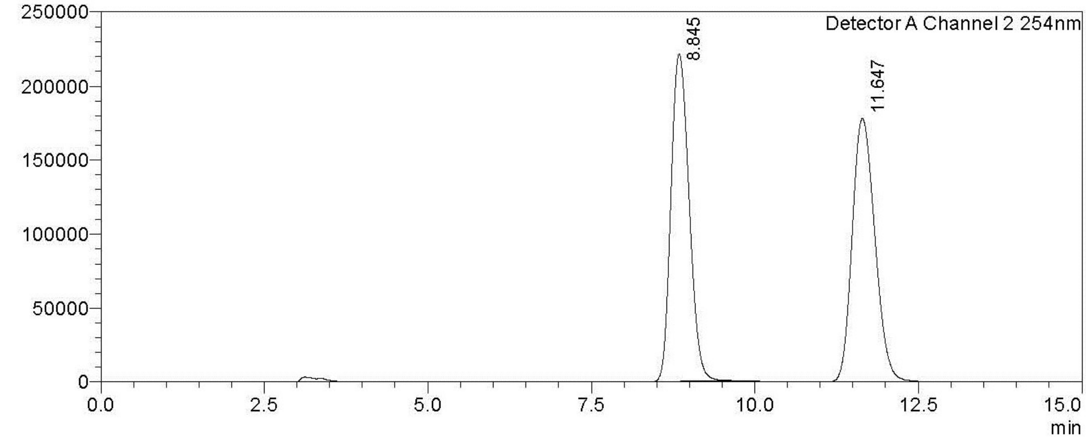


Detector A Channel 2 254nm

| Peak# | Ret. Time | Area | Height | Height% | Area% |
|---|---|---|---|---|---|
| 1 | 8.845 | 4271617 | 221034 | 55.404 | 49.816 |
| 2 | 11.647 | 4303236 | 177915 | 44.596 | 50.184 |
| Total | | 8574853 | 398948 | 100.000 | 100.000 |

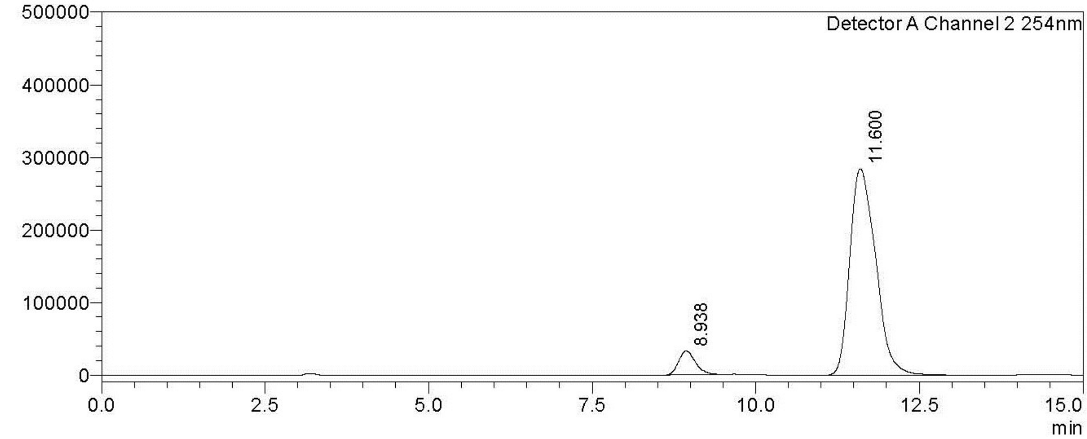


Detector A Channel 2 254nm

| Peak# | Ret. Time | Area | Height | Height% | Area% |
|---|---|---|---|---|---|
| 1 | 8.938 | 590059 | 32703 | 10.326 | 7.018 |
| 2 | 11.600 | 7817801 | 283992 | 89.674 | 92.982 |
| Total | | 8407860 | 316695 | 100.000 | 100.000 |

**Supplementary Figure 81.** HPLC spectra for **3k**.

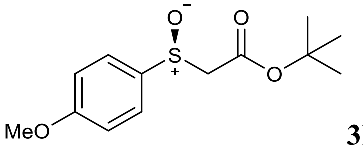


**3l**

LLZ1289B-OB1010230-WCH8012-2-RACEMIC

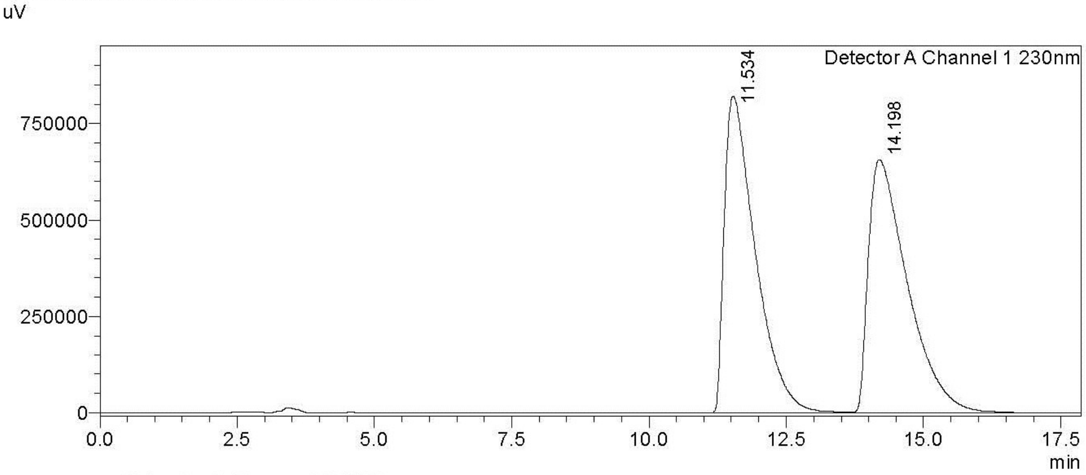


Detector A Channel 1 230nm

| Peak# | Ret. Time | Area | Height | Height% | Area% |
|---|---|---|---|---|---|
| 1 | 11.534 | 32336555 | 820966 | 55.591 | 50.007 |
| 2 | 14.198 | 32328104 | 655821 | 44.409 | 49.993 |
| Total | | 64664659 | 1476787 | 100.000 | 100.000 |

LLZ2222-OB1010230-2

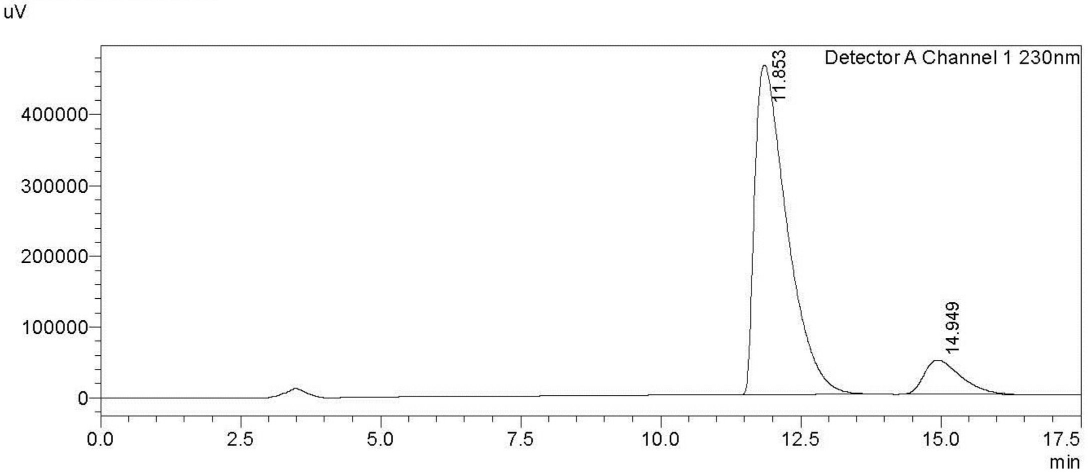


Detector A Channel 1 230nm

| Peak# | Ret. Time | Area | Height | Height% | Area% |
|---|---|---|---|---|---|
| 1 | 11.853 | 18658011 | 466284 | 90.617 | 89.503 |
| 2 | 14.949 | 2188123 | 48283 | 9.383 | 10.497 |
| Total | | 20846134 | 514567 | 100.000 | 100.000 |

**Supplementary Figure 82.** HPLC spectra for **3l**.

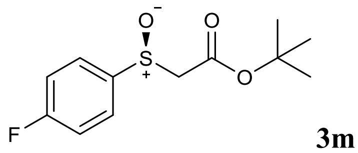

**3m**

LLZ1304-OD1010230-RACEMIC-1

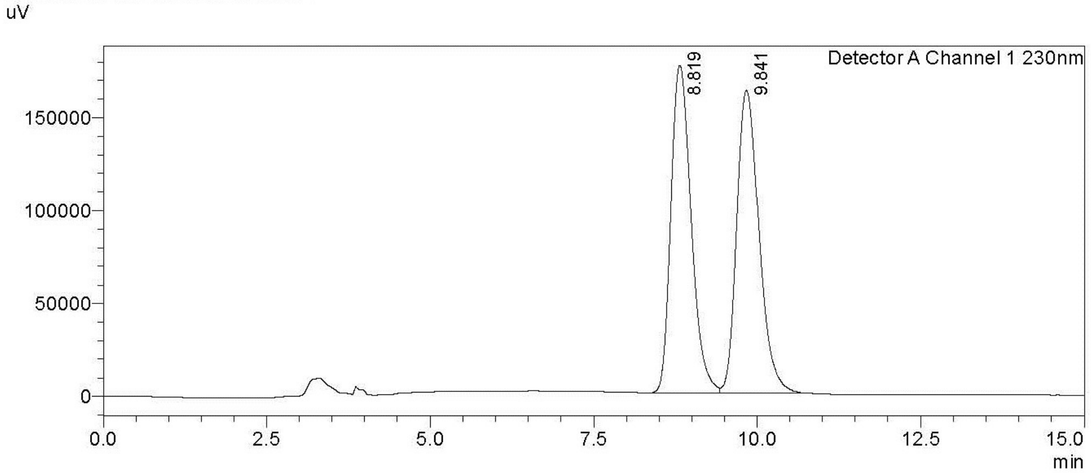


Detector A Channel 1 230nm

| Peak# | Ret. Time | Area | Height | Height% | Area% |
|---|---|---|---|---|---|
| 1 | 8.819 | 3814532 | 176377 | 51.991 | 49.873 |
| 2 | 9.841 | 3833964 | 162869 | 48.009 | 50.127 |
| Total | | 7648496 | 339245 | 100.000 | 100.000 |

LLZ2188-OD1010230-1

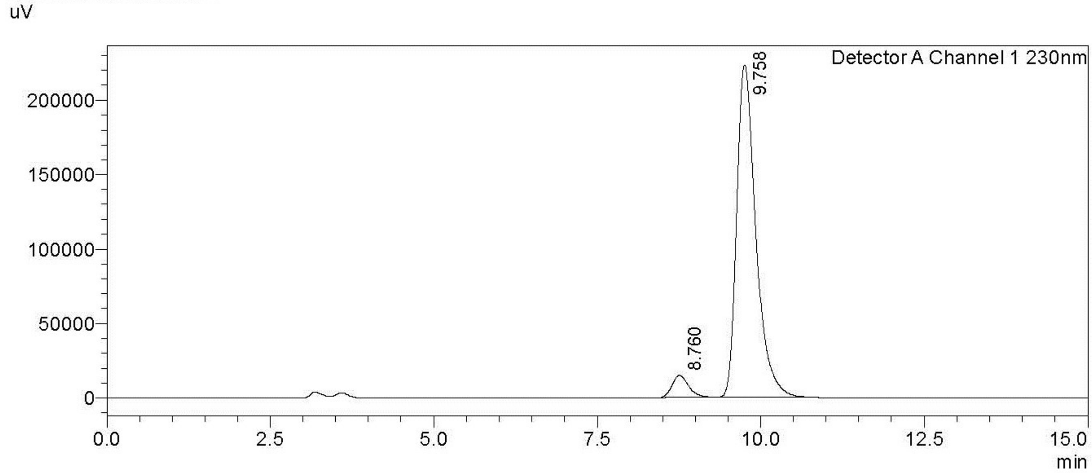


Detector A Channel 1 230nm

| Peak# | Ret. Time | Area | Height | Height% | Area% |
|---|---|---|---|---|---|
| 1 | 8.760 | 254123 | 14678 | 6.168 | 5.333 |
| 2 | 9.758 | 4511179 | 223308 | 93.832 | 94.667 |
| Total | | 4765302 | 237986 | 100.000 | 100.000 |

**Supplementary Figure 83.** HPLC spectra for **3m**.

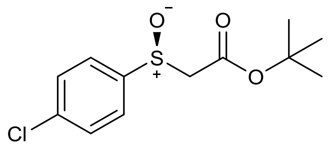


**3n**

LLZ1289C-OB1010WCH8009-1-RACEMIC.lcd

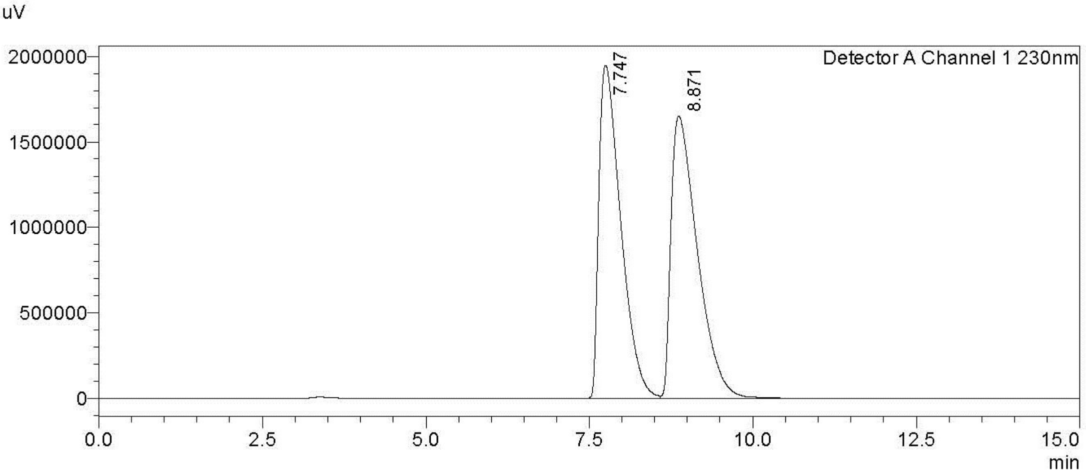


Detector A Channel 1 230nm

| Peak# | Ret. Time | Area | Height | Height% | Area% |
|---|---|---|---|---|---|
| 1 | 7.747 | 45435830 | 1948739 | 54.174 | 49.539 |
| 2 | 8.871 | 46281252 | 1648456 | 45.826 | 50.461 |
| Total | | 91717082 | 3597195 | 100.000 | 100.000 |

LLZ2144-OB1010230-2

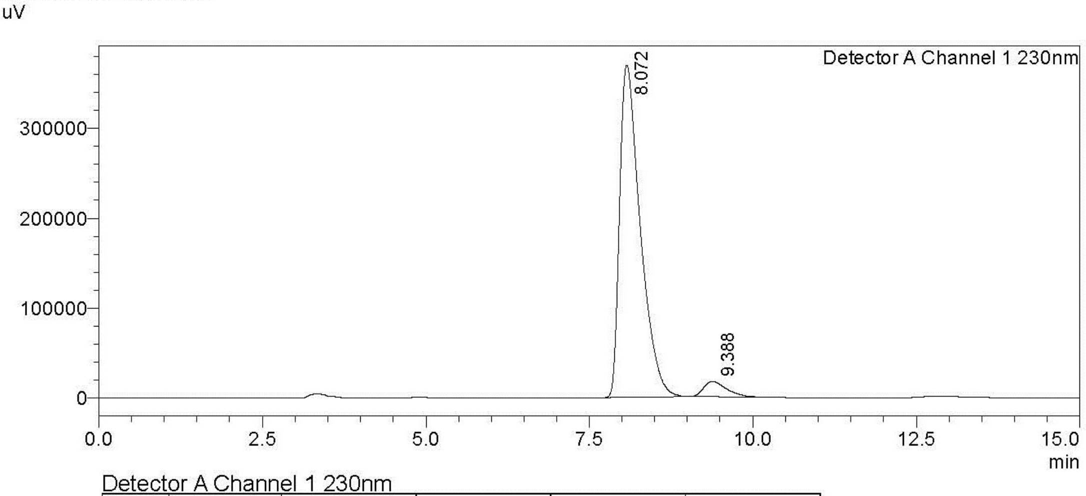


Detector A Channel 1 230nm

| Peak# | Ret. Time | Area | Height | Height% | Area% |
|---|---|---|---|---|---|
| 1 | 8.072 | 8124280 | 369722 | 95.600 | 95.166 |
| 2 | 9.388 | 412650 | 17016 | 4.400 | 4.834 |
| Total | | 8536930 | 386738 | 100.000 | 100.000 |

**Supplementary Figure 84.** HPLC spectra for **3n**.

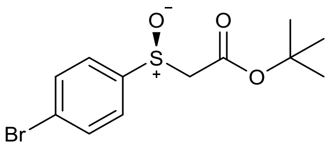

**3o**

LLZ1294A-OB1010230-RACEMMIC

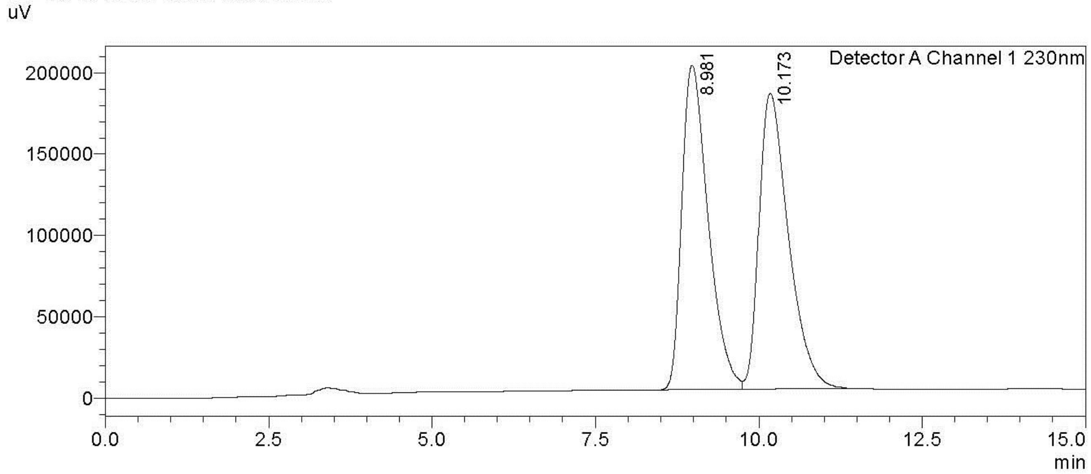


Detector A Channel 1 230nm

| Peak# | Ret. Time | Area | Height | Height% | Area% |
|---|---|---|---|---|---|
| 1 | 8.981 | 5599451 | 199463 | 52.315 | 49.766 |
| 2 | 10.173 | 5652105 | 181813 | 47.685 | 50.234 |
| Total | | 11251556 | 381276 | 100.000 | 100.000 |

LLZ2144-OB1010230-3

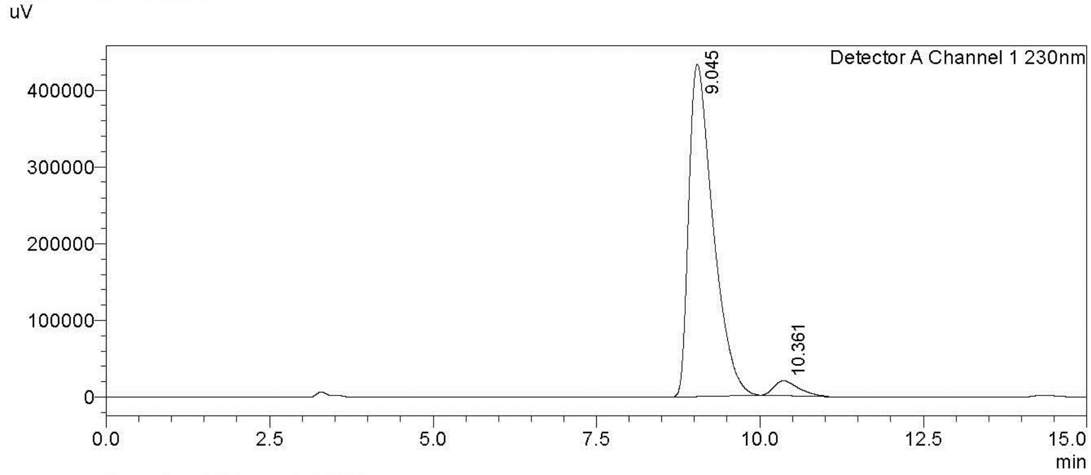


Detector A Channel 1 230nm

| Peak# | Ret. Time | Area | Height | Height% | Area% |
|---|---|---|---|---|---|
| 1 | 9.045 | 11224214 | 432969 | 95.803 | 95.778 |
| 2 | 10.361 | 494798 | 18967 | 4.197 | 4.222 |
| Total | | 11719013 | 451936 | 100.000 | 100.000 |

**Supplementary Figure 85.** HPLC spectra for **3o**.

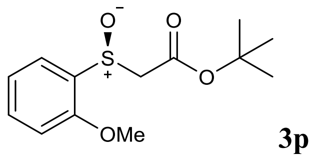


**3p**

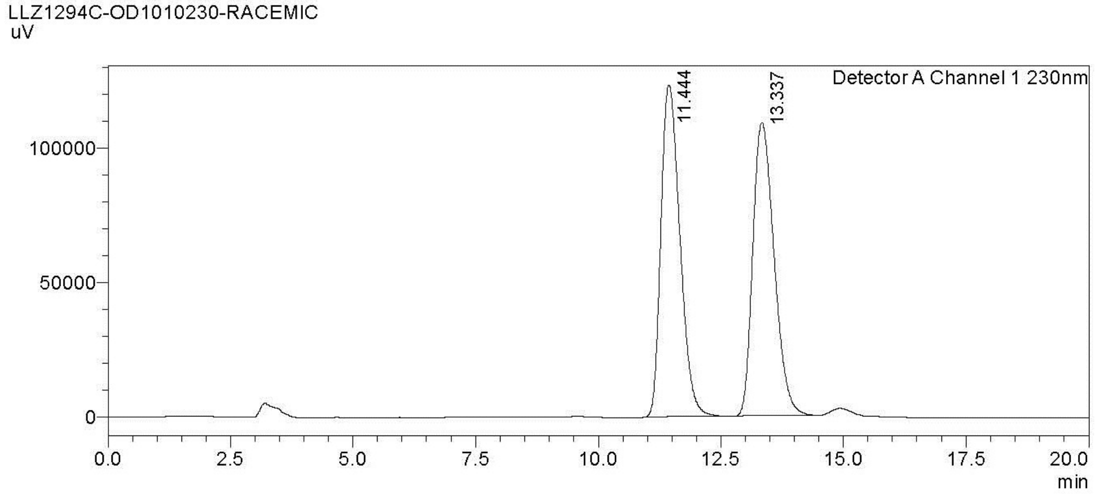


Detector A Channel 1 230nm

| Peak# | Ret. Time | Area | Height | Height% | Area% |
|---|---|---|---|---|---|
| 1 | 11.444 | 3299147 | 123326 | 53.097 | 50.043 |
| 2 | 13.337 | 3293446 | 108940 | 46.903 | 49.957 |
| Total | | 6592594 | 232265 | 100.000 | 100.000 |

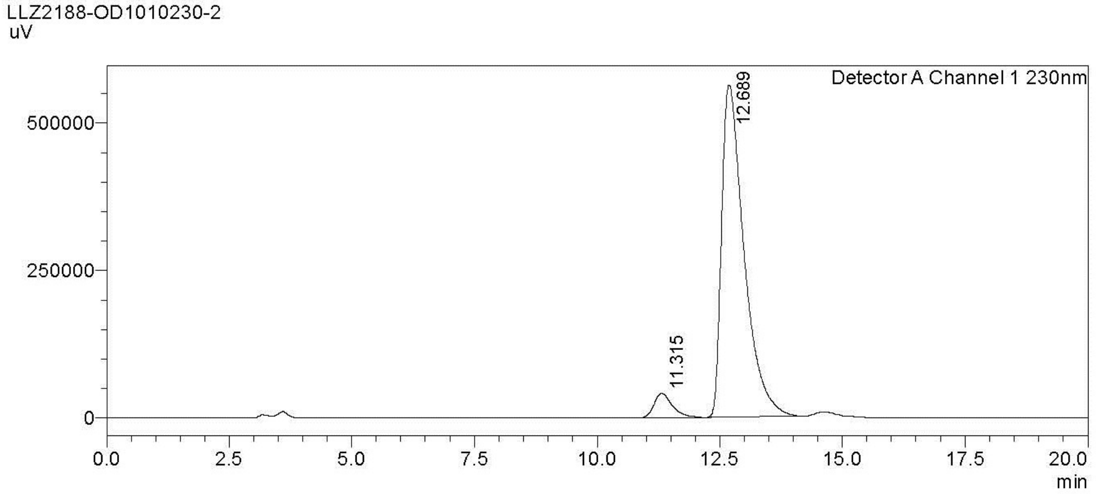


Detector A Channel 1 230nm

| Peak# | Ret. Time | Area | Height | Height% | Area% |
|---|---|---|---|---|---|
| 1 | 11.315 | 1043423 | 40769 | 6.738 | 5.497 |
| 2 | 12.689 | 17937560 | 564262 | 93.262 | 94.503 |
| Total | | 18980983 | 605032 | 100.000 | 100.000 |

**Supplementary Figure 86.** HPLC spectra for **3p**.

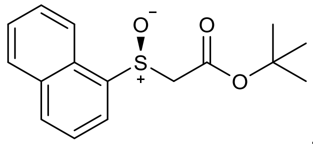

**3q**

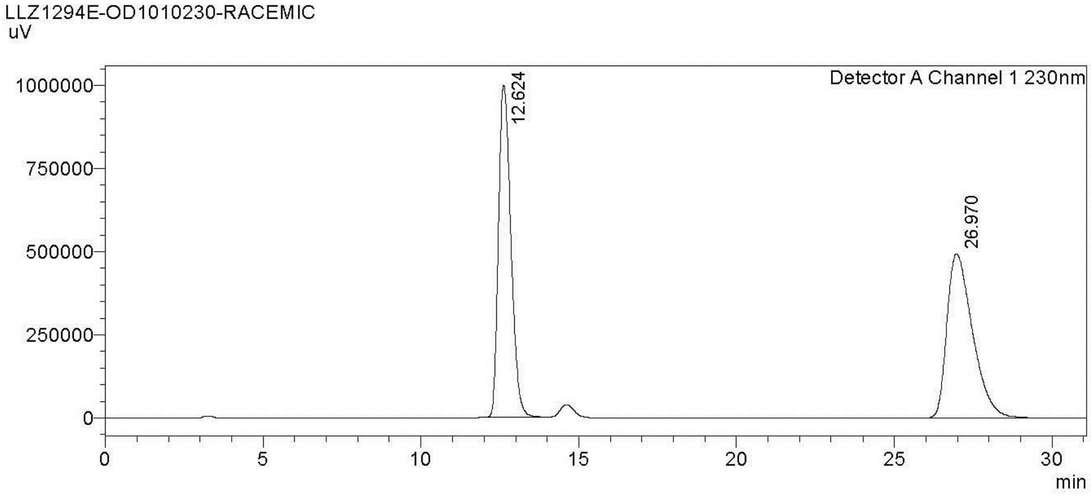


Detector A Channel 1 230nm

| Peak# | Ret. Time | Area | Height | Height% | Area% |
|---|---|---|---|---|---|
| 1 | 12.624 | 27227730 | 999203 | 67.003 | 49.645 |
| 2 | 26.970 | 27617072 | 492074 | 32.997 | 50.355 |
| Total | | 54844802 | 1491277 | 100.000 | 100.000 |

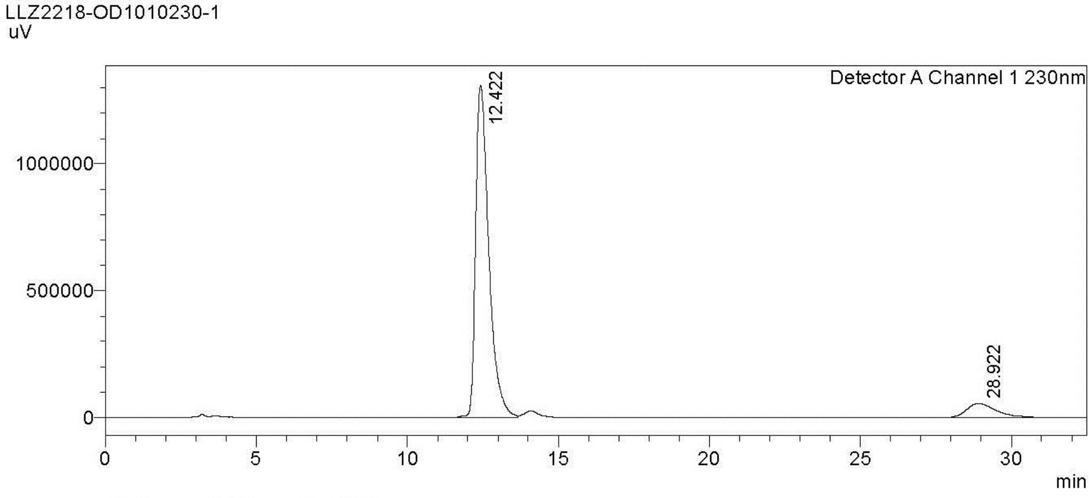


Detector A Channel 1 230nm

| Peak# | Ret. Time | Area | Height | Height% | Area% |
|---|---|---|---|---|---|
| 1 | 12.422 | 39625648 | 1311781 | 95.994 | 91.654 |
| 2 | 28.922 | 3608374 | 54745 | 4.006 | 8.346 |
| Total | | 43234022 | 1366527 | 100.000 | 100.000 |

**Supplementary Figure 87.** HPLC spectra for **3q**.

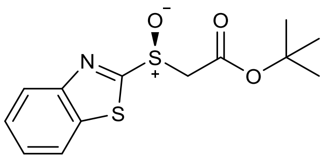

**3r**

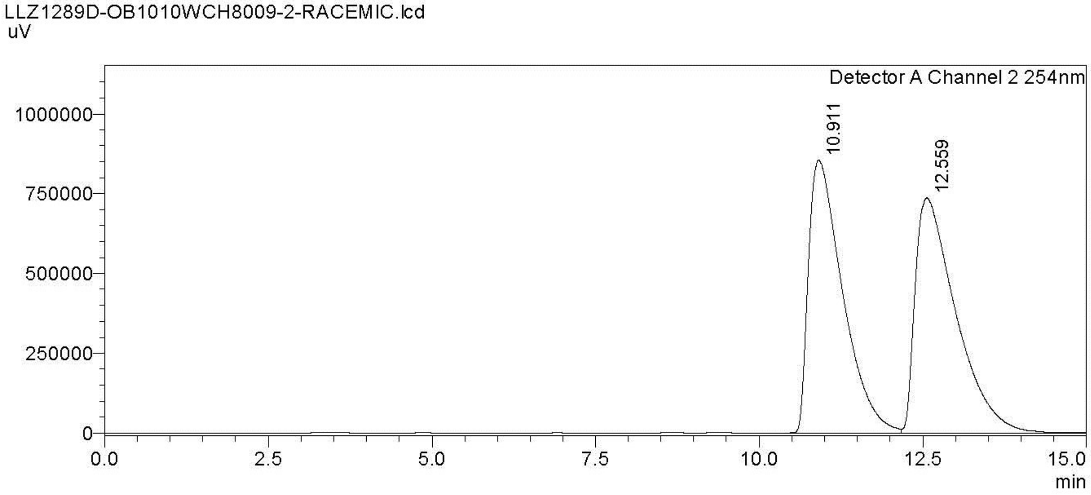


Detector A Channel 2 254nm

| Peak# | Ret. Time | Area | Height | Height% | Area% |
|---|---|---|---|---|---|
| 1 | 10.911 | 31092814 | 854227 | 53.712 | 49.537 |
| 2 | 12.559 | 31673643 | 736167 | 46.288 | 50.463 |
| Total | | 62766457 | 1590394 | 100.000 | 100.000 |

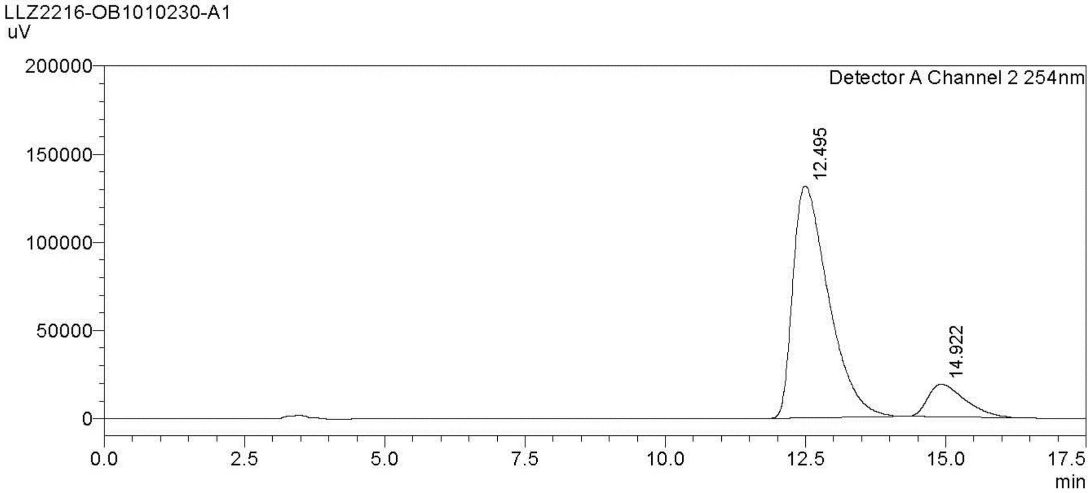


Detector A Channel 2 254nm

| Peak# | Ret. Time | Area | Height | Height% | Area% |
|---|---|---|---|---|---|
| 1 | 12.495 | 5825149 | 131799 | 87.723 | 87.311 |
| 2 | 14.922 | 846582 | 18445 | 12.277 | 12.689 |
| Total | | 6671732 | 150244 | 100.000 | 100.000 |

**Supplementary Figure 88.** HPLC spectra for **3r**.

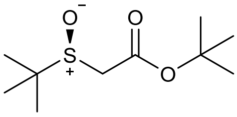

**3s**

LLZ2048-OD0510230-RACEMIC

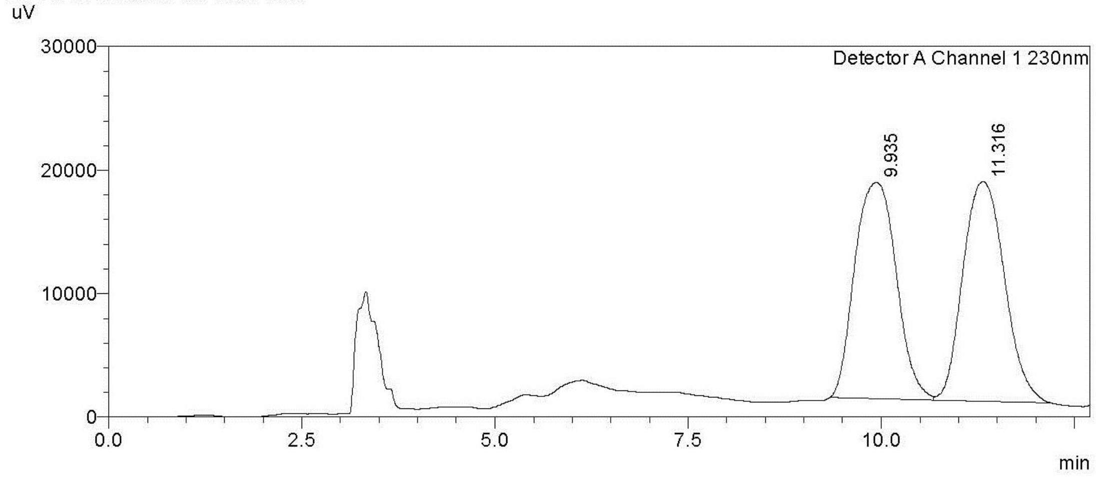


Detector A Channel 1 230nm

| Peak# | Ret. Time | Area | Height | Height% | Area% |
|---|---|---|---|---|---|
| 1 | 9.935 | 654120 | 17513 | 49.642 | 49.597 |
| 2 | 11.316 | 664743 | 17765 | 50.358 | 50.403 |
| Total | | 1318863 | 35278 | 100.000 | 100.000 |

LLZ2048-OD0510230-2

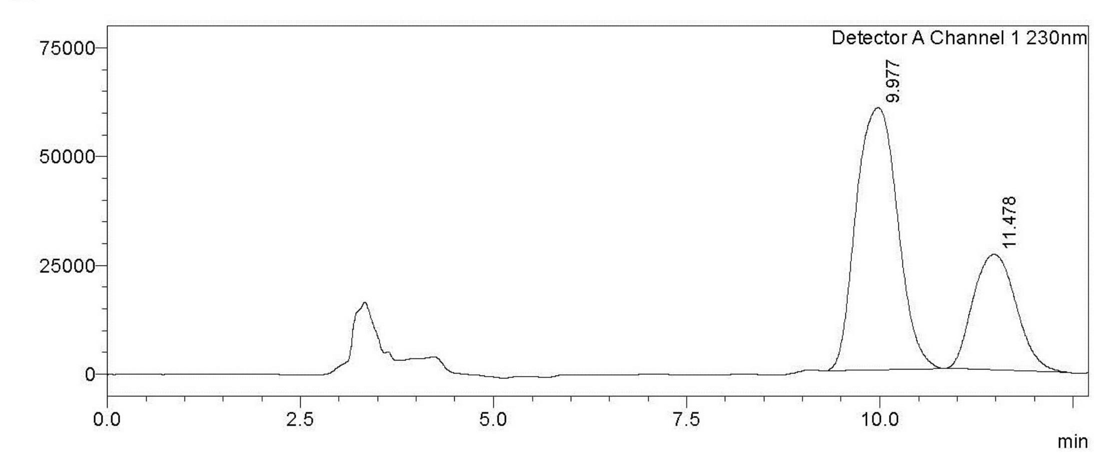


Detector A Channel 1 230nm

| Peak# | Ret. Time | Area | Height | Height% | Area% |
|---|---|---|---|---|---|
| 1 | 9.977 | 2258187 | 60154 | 69.401 | 68.699 |
| 2 | 11.478 | 1028888 | 26521 | 30.599 | 31.301 |
| Total | | 3287075 | 86675 | 100.000 | 100.000 |

**Supplementary Figure 89.** HPLC spectra for **3s**.

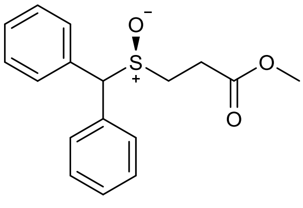

**3t**

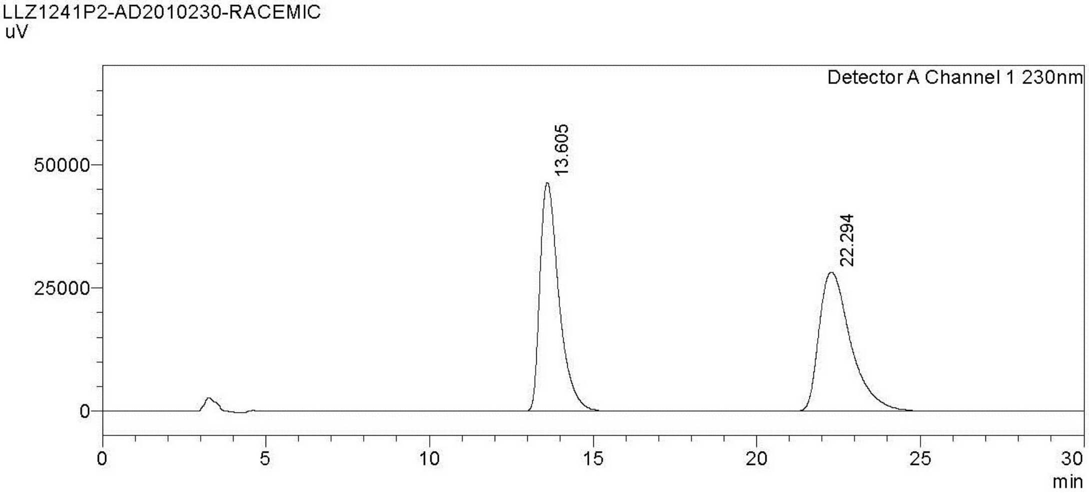


Detector A Channel 1 230nm

| Peak# | Ret. Time | Area | Height | Height% | Area% |
|---|---|---|---|---|---|
| 1 | 13.605 | 1877928 | 46395 | 62.220 | 49.982 |
| 2 | 22.294 | 1879283 | 28171 | 37.780 | 50.018 |
| Total | | 3757211 | 74566 | 100.000 | 100.000 |

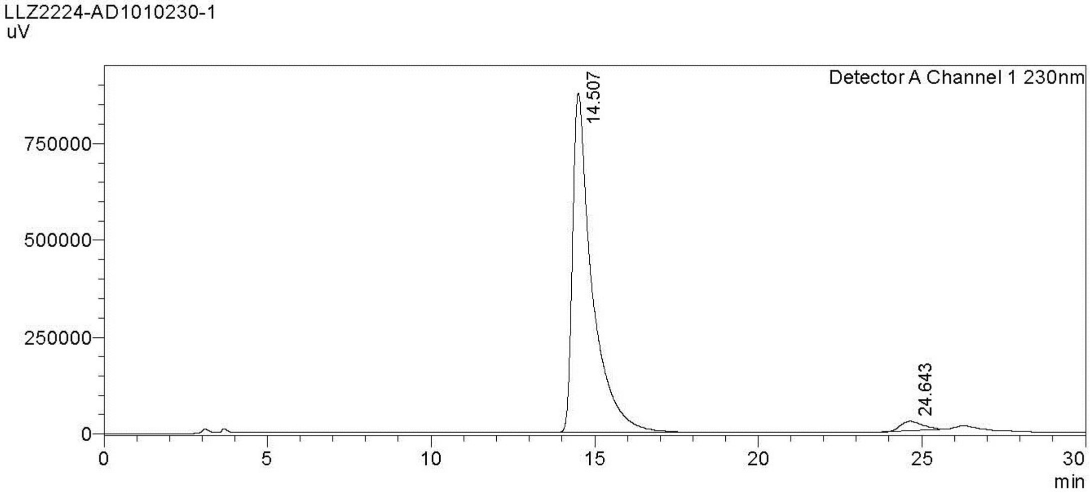


Detector A Channel 1 230nm

| Peak# | Ret. Time | Area | Height | Height% | Area% |
|---|---|---|---|---|---|
| 1 | 14.507 | 35994828 | 875798 | 97.303 | 96.944 |
| 2 | 24.643 | 1134818 | 24271 | 2.697 | 3.056 |
| Total | | 37129645 | 900070 | 100.000 | 100.000 |

**Supplementary Figure 90.** HPLC spectra for **3t**.

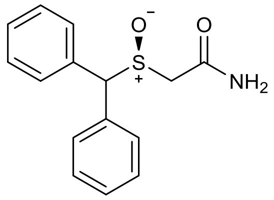

**3u**

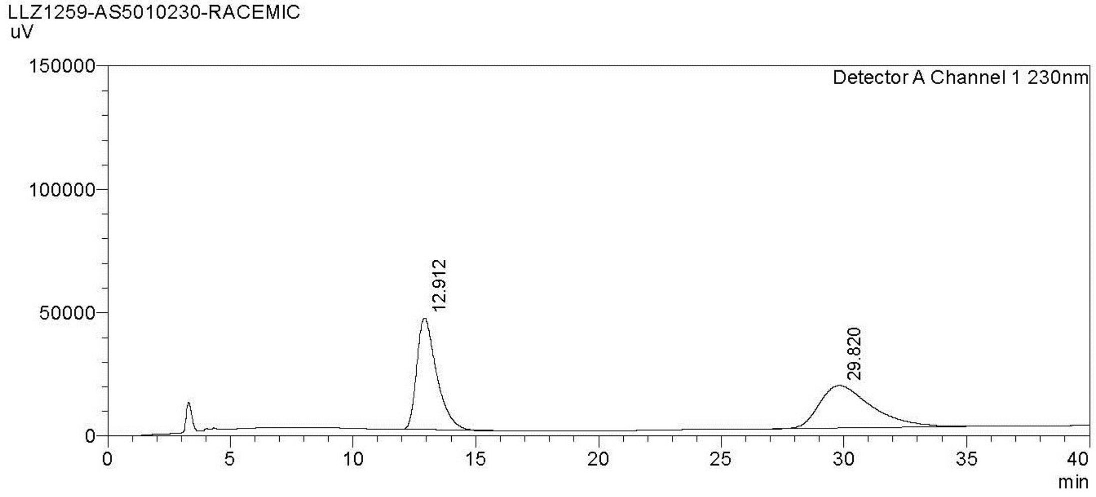


Detector A Channel 1 230nm

| Peak# | Ret. Time | Area | Height | Height% | Area% |
|---|---|---|---|---|---|
| 1 | 12.912 | 2511425 | 45293 | 72.490 | 49.529 |
| 2 | 29.820 | 2559161 | 17189 | 27.510 | 50.471 |
| Total | | 5070586 | 62481 | 100.000 | 100.000 |

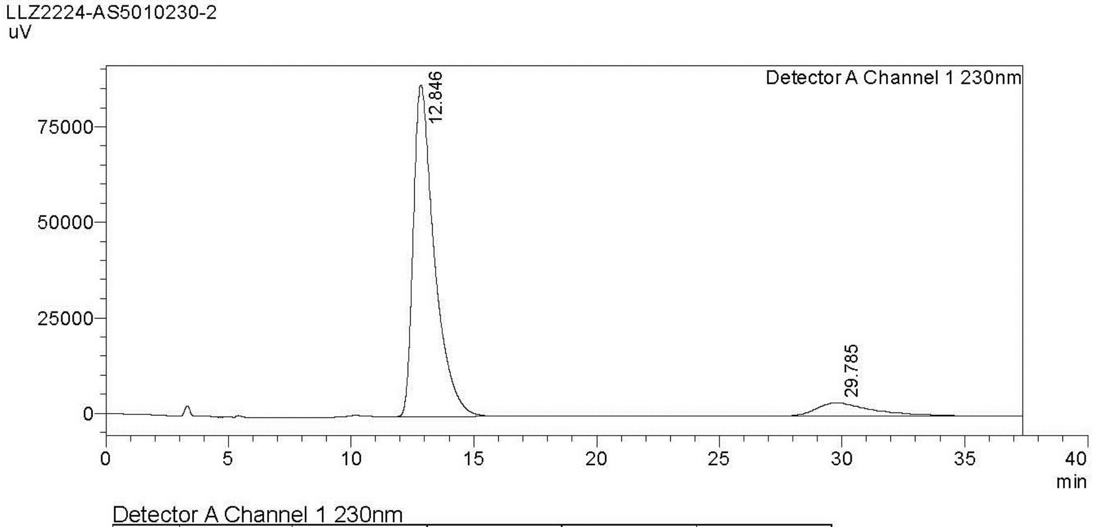


Detector A Channel 1 230nm

| Peak# | Ret. Time | Area | Height | Height% | Area% |
|---|---|---|---|---|---|
| 1 | 12.846 | 5190090 | 86860 | 96.252 | 90.903 |
| 2 | 29.785 | 519414 | 3382 | 3.748 | 9.097 |
| Total | | 5709504 | 90242 | 100.000 | 100.000 |

**Supplementary Figure 91.** HPLC spectra for **3u**.

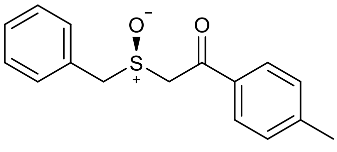

**3v**

LLZ2112-AD1010210-racemic-2

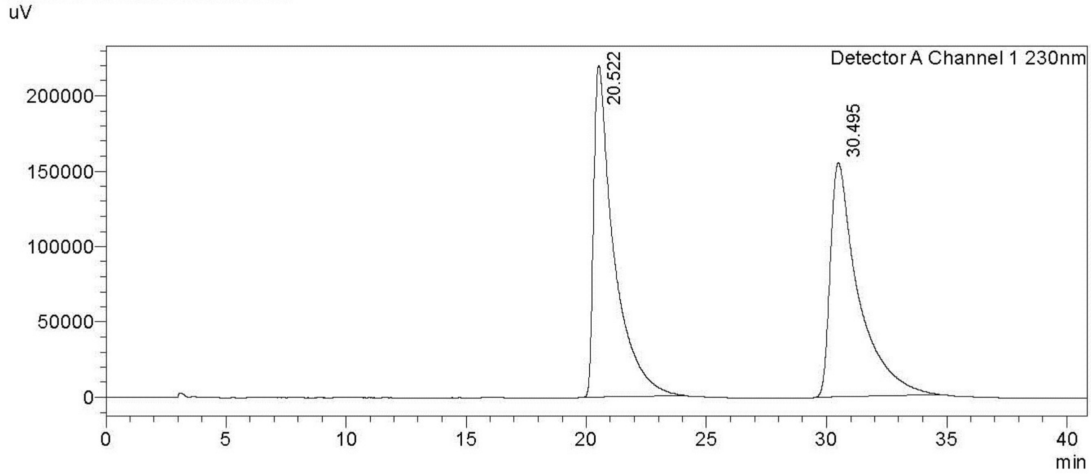


Detector A Channel 1 230nm

| Peak# | Ret. Time | Area | Height | Height% | Area% |
|---|---|---|---|---|---|
| 1 | 20.522 | 12855420 | 220121 | 58.650 | 50.503 |
| 2 | 30.495 | 12599407 | 155192 | 41.350 | 49.497 |
| Total | | 25454827 | 375313 | 100.000 | 100.000 |

LLZ2202-AD1010230-2

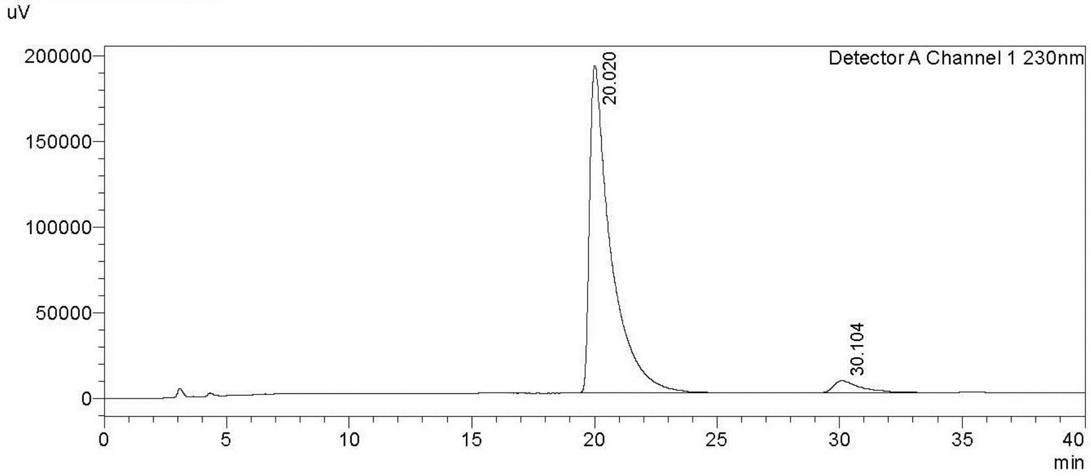


Detector A Channel 1 230nm

| Peak# | Ret. Time | Area | Height | Height% | Area% |
|---|---|---|---|---|---|
| 1 | 20.020 | 11482708 | 191400 | 96.399 | 95.260 |
| 2 | 30.104 | 571332 | 7150 | 3.601 | 4.740 |
| Total | | 12054040 | 198550 | 100.000 | 100.000 |

**Supplementary Figure 92.** HPLC spectra for **3v**.

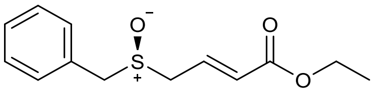


**3w**

LLZ2200-OB5010230-RACEMIC-3

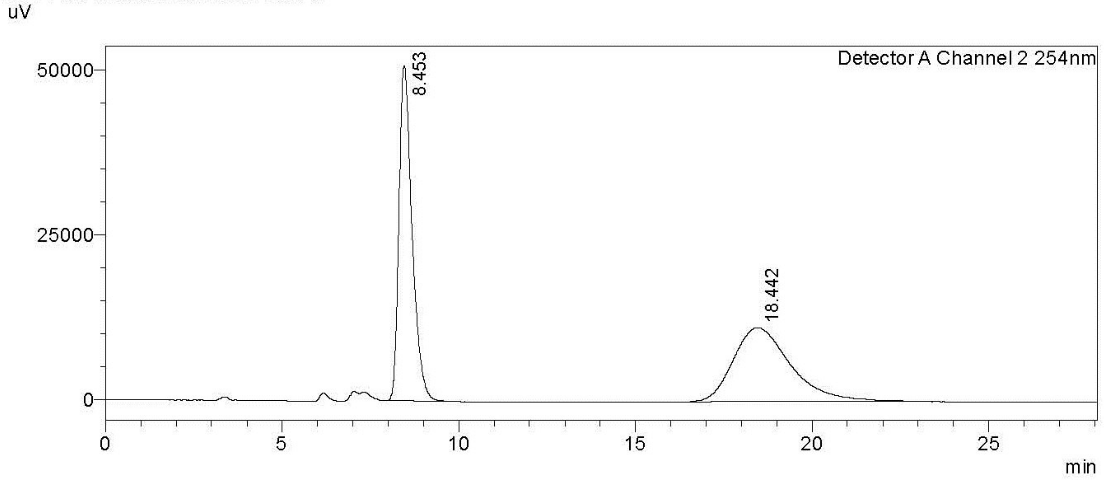


Detector A Channel 2 254nm

| Peak# | Ret. Time | Area | Height | Height% | Area% |
|---|---|---|---|---|---|
| 1 | 8.453 | 1306886 | 50921 | 81.941 | 50.842 |
| 2 | 18.442 | 1263624 | 11222 | 18.059 | 49.158 |
| Total | | 2570510 | 62143 | 100.000 | 100.000 |

LLZ2224-OB5010230-3

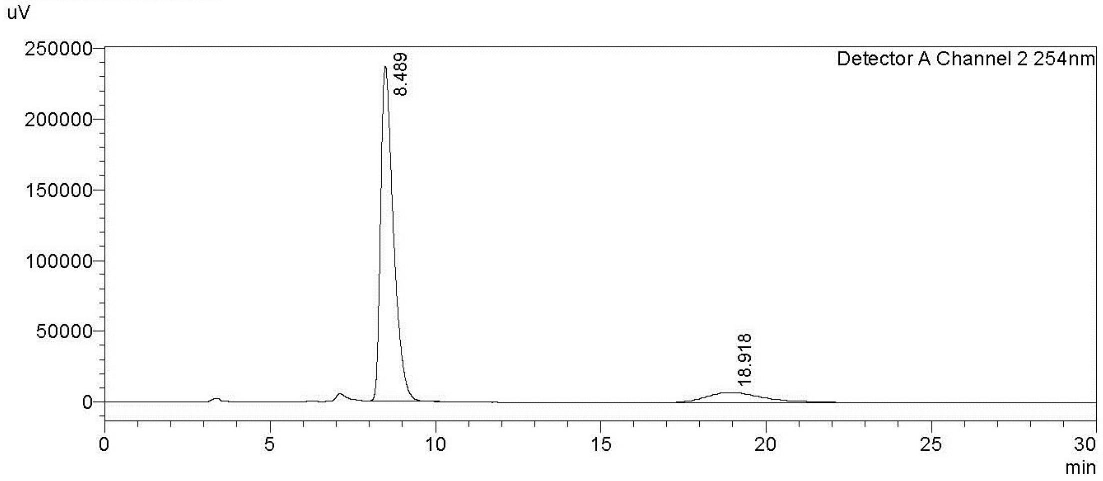


Detector A Channel 2 254nm

| Peak# | Ret. Time | Area | Height | Height% | Area% |
|---|---|---|---|---|---|
| 1 | 8.489 | 6372456 | 236889 | 97.103 | 88.602 |
| 2 | 18.918 | 819770 | 7067 | 2.897 | 11.398 |
| Total | | 7192227 | 243956 | 100.000 | 100.000 |

**Supplementary Figure 93.** HPLC spectra for **3w**.

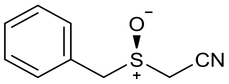


3x

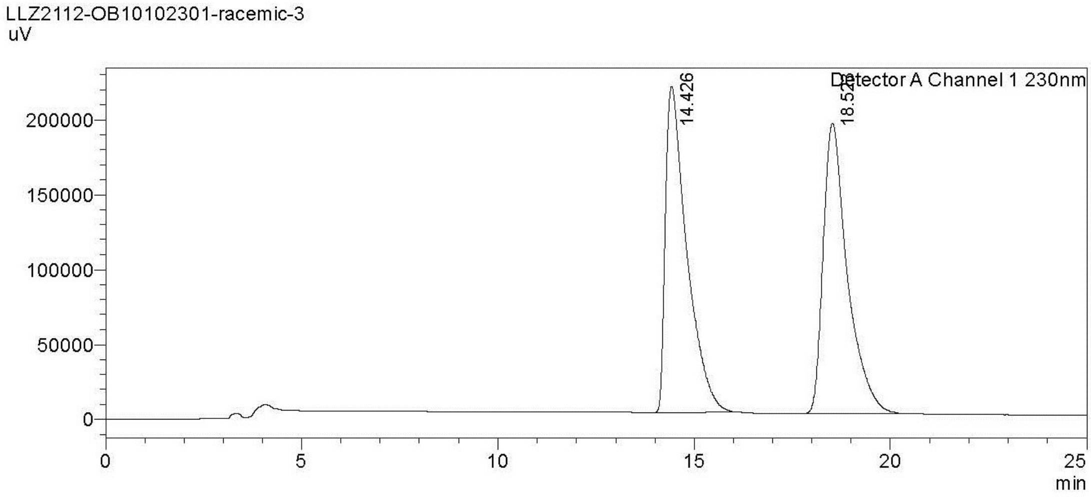


Detector A Channel 1 230nm

| Peak# | Ret. Time | Area | Height | Height% | Area% |
|---|---|---|---|---|---|
| 1 | 14.426 | 8293177 | 217624 | 52.914 | 50.023 |
| 2 | 18.523 | 8285455 | 193657 | 47.086 | 49.977 |
| Total | | 16578632 | 411281 | 100.000 | 100.000 |

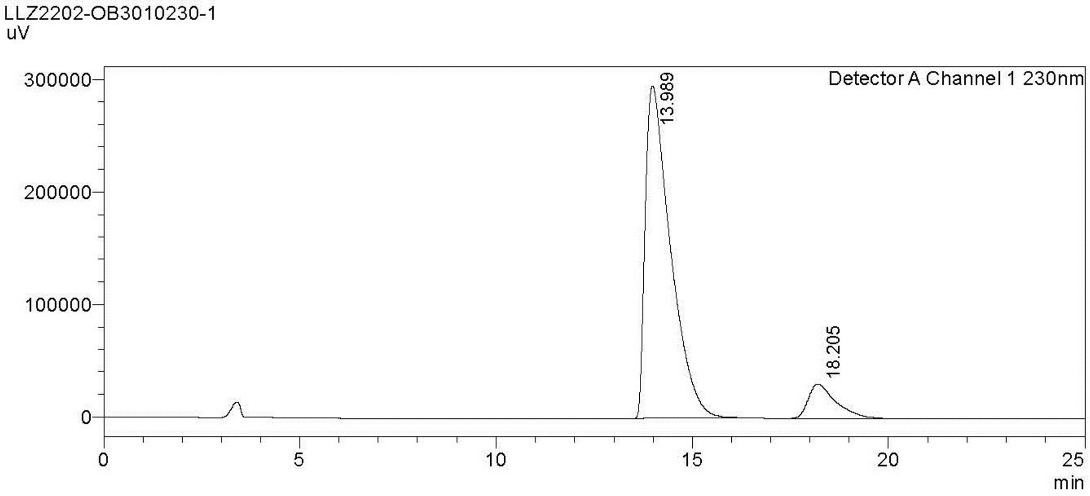


Detector A Channel 1 230nm

| Peak# | Ret. Time | Area | Height | Height% | Area% |
|---|---|---|---|---|---|
| 1 | 13.989 | 13269614 | 295746 | 90.661 | 90.042 |
| 2 | 18.205 | 1467581 | 30465 | 9.339 | 9.958 |
| Total | | 14737196 | 326212 | 100.000 | 100.000 |

**Supplementary Figure 94.** HPLC spectra for **3x**.

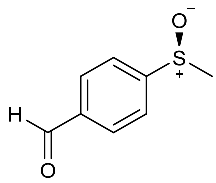

**3y**

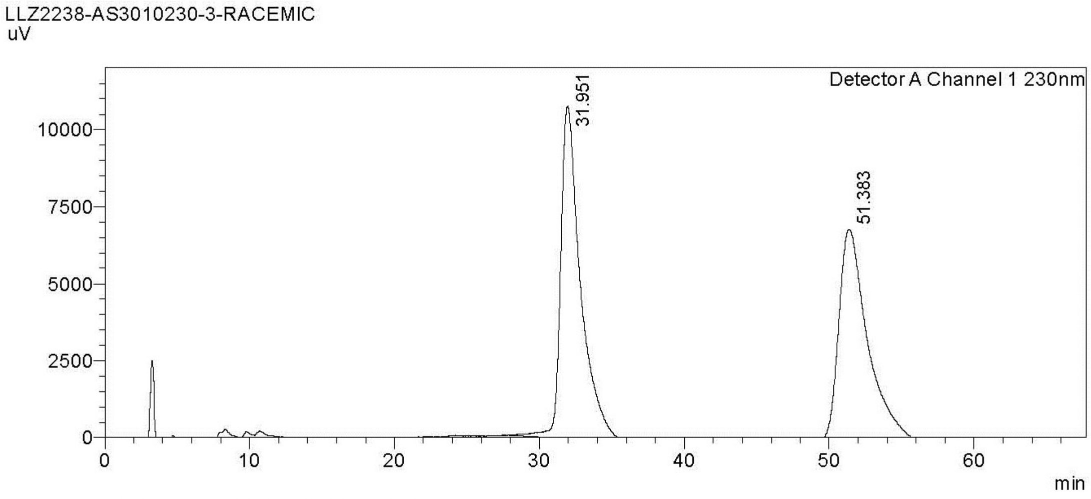


Detector A Channel 1 230nm

| Peak# | Ret. Time | Area | Height | Height% | Area% |
|---|---|---|---|---|---|
| 1 | 31.951 | 1032154 | 10792 | 60.754 | 50.600 |
| 2 | 51.383 | 1007694 | 6971 | 39.246 | 49.400 |
| Total | | 2039848 | 17763 | 100.000 | 100.000 |

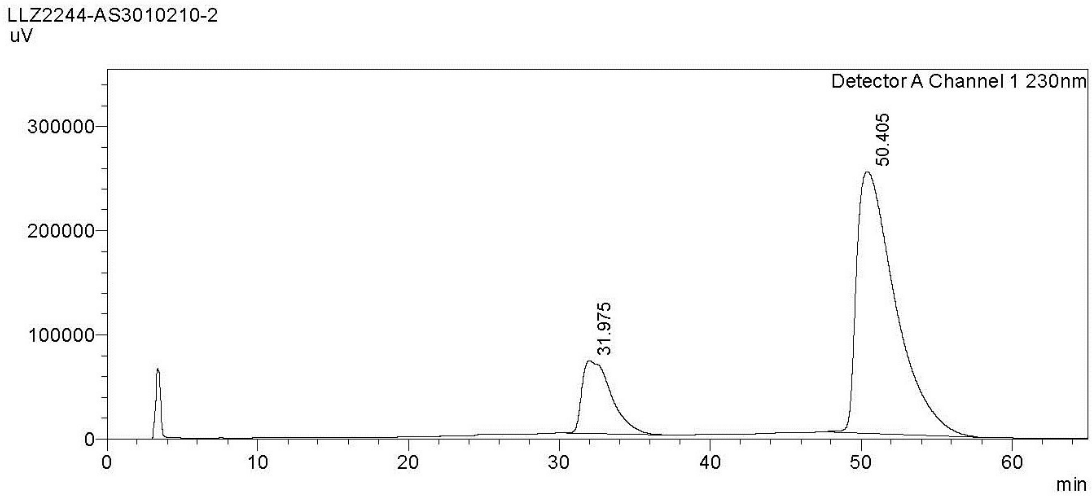


Detector A Channel 1 230nm

| Peak# | Ret. Time | Area | Height | Height% | Area% |
|---|---|---|---|---|---|
| 1 | 31.975 | 9475351 | 69873 | 21.748 | 17.421 |
| 2 | 50.405 | 44913807 | 251410 | 78.252 | 82.579 |
| Total | | 54389158 | 321283 | 100.000 | 100.000 |

**Supplementary Figure 95.** HPLC spectra for **3y**.

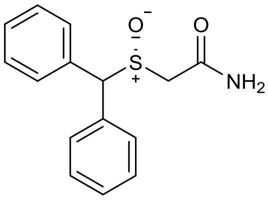


**4**

LLZ1259-AS5010230-RACEMIC

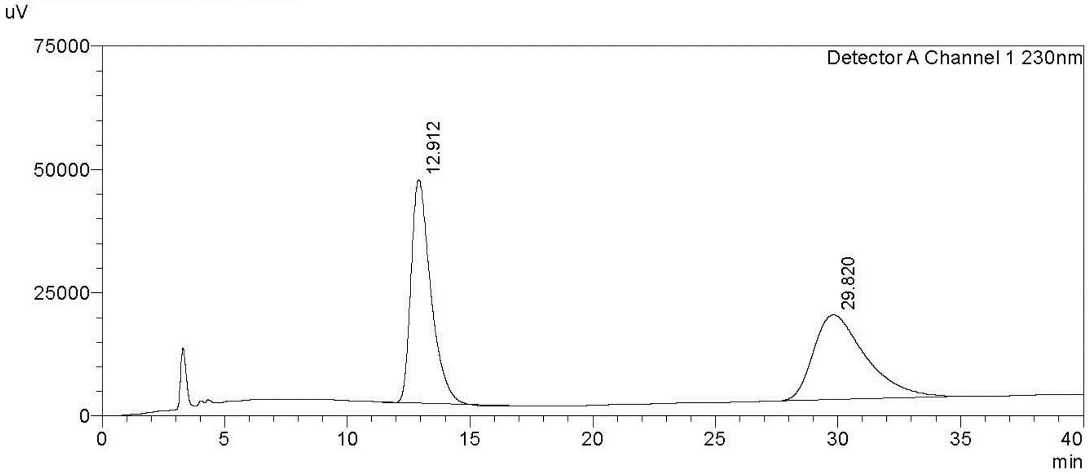


Detector A Channel 1 230nm

| Peak# | Ret. Time | Area | Height | Height% | Area% |
|---|---|---|---|---|---|
| 1 | 12.912 | 2511425 | 45293 | 72.490 | 49.529 |
| 2 | 29.820 | 2559161 | 17189 | 27.510 | 50.471 |
| Total | | 5070586 | 62481 | 100.000 | 100.000 |

LLZ2212-AS5010230-REPEAT

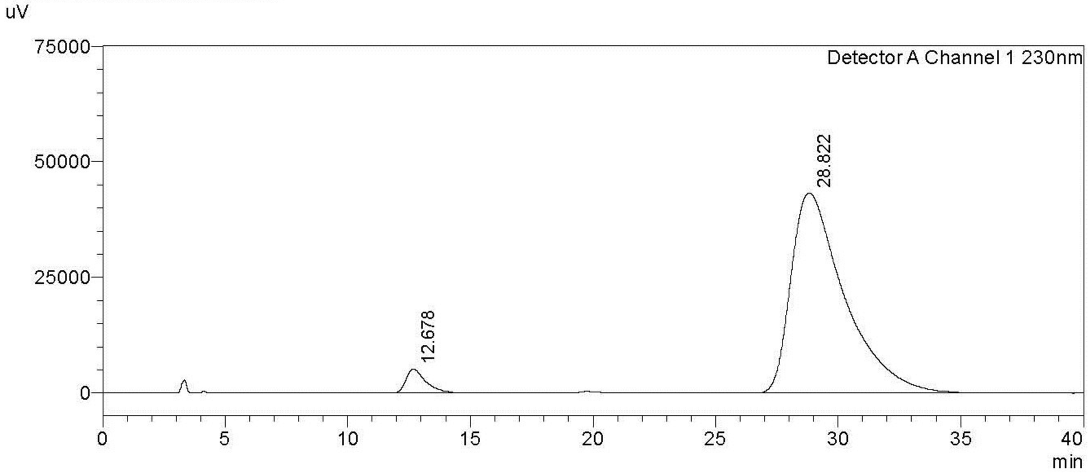


Detector A Channel 1 230nm

| Peak# | Ret. Time | Area | Height | Height% | Area% |
|---|---|---|---|---|---|
| 1 | 12.678 | 288657 | 5188 | 10.700 | 4.207 |
| 2 | 28.822 | 6573035 | 43301 | 89.300 | 95.793 |
| Total | | 6861693 | 48489 | 100.000 | 100.000 |

**Supplementary Figure 96.** HPLC spectra for **4**.

## Supplementary Tables

**Supplementary Table 1.** Control experiments by using (*R*,*R*)-**1a** with various acidic additives

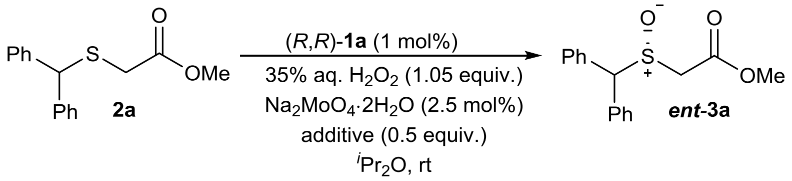


| Entry | Cat | Additive | Time (min) | Yield (%)* | ee (%)† |
|---|---|---|---|---|---|
| 1 | (*R*,*R*)-**1a** | - | 1440 | 15 | 5 |
| 2 | (*R*,*R*)-**1a** | $KHSO_4$ | 30 | 94 | 92 |
| 3 | (*R*,*R*)-**1a** | $TBAHSO_4$ | 60 | 89 | 89 |
| 4 | (*R*,*R*)-**1a** | $CH_3SO_3H$ | 120 | 84 | 5 |
| 5 | (*R*,*R*)-**1a** | $CF_3SO_3H$ | 60 | 98 | 0 |
| 6‡ | (*R*,*R*)-**1a** | $H_3PO_4$ | 240 | 89 | 17 |
| 7‡ | (*R*,*R*)-**1a** | $H_2SO_4$ | 10 | 99 | 95 |
| 8‡ | (*R*,*R*)-**1a** | HCl | 240 | 17 | 2 |
| 9‡ | (*R*,*R*)-**1a** | $H_2SO_4$ (0.1 equiv.) | 10 | 99 | 90 |
| 10‡ | (*R*,*R*)-**1a** | $H_2SO_4$ (0.05 equiv.) | 90 | 80 | 91 |
| 11‡ | (*R*,*R*)-**1a** | $H_2SO_4$ (0.01 equiv.) | 1440 | 27 | 2 |
| 12‡,§ | (*R*,*R*)-**1a** | $H_2SO_4$ | 240 | 0 | NA |

Conditions: reaction was performed with 0.05 mmol of **2a** in the presence of 1 mol% of chiral bisguanidinium (*R*,*R*)-**1a** and 2.5 mol% of $Na_2MoO_4{\cdot}2H_2O$ in 1.0 mL of $^i$Pr$_2$O at room temperature. *Yield of the isolated product. †Determined by HPLC analysis. ‡Aqueous $H_3PO_4$, $H_2SO_4$, HCl solution were freshly prepared with the concentration of 1.0 M. §Without $Na_2MoO_4{\cdot}2H_2O$. TBA= tetrabutylammonium.

**Supplementary Table 2.** (*R*,*R*)-**1b** as the sole oxidant.

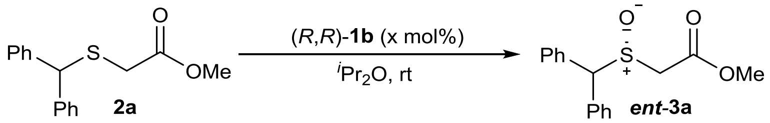


| Entry | Cat | x | Time (min) | Yield (%)* | *ee* (%)† |
|---|---|---|---|---|---|
| 1 | (*R*,*R*)-**1b** | 100 | 30 | 97 | 80 |
| 2 | (*R*,*R*)-**1b** | 50 | 120 | 79‡ | 37 |
| 3 | (*R*,*R*)-**1b** | 25 | 120 | 50‡ | 31 |
| 4§ | - | 1 | 120 | 0 | - |
| 5‖ | - | 1 | 120 | 99 | 89 |

Conditions: reaction was performed with 0.05 mmol of **2a** in the presence of chiral bisguanidinium (*R*,*R*)-**1b** in 1.0 mL of $^i$Pr$_2$O at room temperature. *Yield of the isolated product. †Determined by HPLC analysis. ‡Deternined by $^1$H NMR analysis. §Catalyst recycled from reaction in entries 2 and 3 was used with the addition of one equivalent of aqueous $H_2O_2$. ||Catalyst recycled from reaction in entries 2 and 3 was used with the addition of 0.5 equivalent of $KHSO_4$ and one equivalent of aqueous $H_2O_2$.

**Supplementary Table 3.** Comparison of DFT energies of crystal and optimized ion-pairing geometry

| Optimization Scheme | SCF Energies (Hartree) | |
|---|---|---|
| | B3LYP/B1, gas | B3LYP/B1, SCRF |
| Crystal | -5572.38793095 | -5572.44123746 |
| ONIOM (QM:QM'), gas | -5572.73584719 | -5572.75104888 |
| ONIOM (QM:QM'), SCRF | -5572.76059146 | -5572.77879677 |

**Supplementary Table 4.** Comparison of energies and ZPE values for all TSs found in conformational sampling studies. Energies are shown in hartrees.

| # | TS | Energy (ONIOM(B3LYP/B2:PM6) //ONIOM(B3LYP/B1:PM6) with SCRF) + Edisp | ZPE (ONIOM(B3LYP /B1:PM6) with SCRF) | Energy + Edisp + ZPE |
|---|---|---|---|---|
| 1 | TSR-01 | -2756.49169582 | 2.15225400 | -2754.33944182 |
| 2 | TSR-02 | -2756.49159293 | 2.15282400 | -2754.33876893 |
| 3 | TSR-03 | -2756.48752580 | 2.15271500 | -2754.33481080 |
| 4 | TSR-04 | -2756.49237962 | 2.15273800 | -2754.33964162 |
| 5 | TSR-05 | -2756.49380733 | 2.15244200 | -2754.34136533 |
| 6 | TSR-06 | -2756.48688620 | 2.15227500 | -2754.33461120 |
| 7 | TSR-07 | -2756.48796040 | 2.15283700 | -2754.33512340 |
| 8 | TSS-01 | -2756.48517606 | 2.15311600 | -2754.33206006 |
| 9 | TSS-02 | -2756.48497831 | 2.15278000 | -2754.33219831 |
| 10 | TSS-03 | -2756.48411938 | 2.15295700 | -2754.33116238 |
| 11 | TSS-04 | -2756.47638012 | 2.15235300 | -2754.32402712 |
| 12 | TSS-05 | -2756.48971805 | 2.15172900 | -2754.33798905 |
| 13 | TSS-06 | -2756.47747080 | 2.15172600 | -2754.32574480 |
| 14 | TSS-07 | -2756.48797039 | 2.15207600 | -2754.33589439 |
| 15 | TSS-08 | -2756.48686450 | 2.15287700 | -2754.33398750 |
| 16 | TSS-09 | -2756.49098334 | 2.15264800 | -2754.33833534 |

**Supplementary Table 5.** Relative energies of all TSs found in conformational sampling studies. Energies are shown in kcal/mol.

| # | TS | Relative Energy (Energy +$E_{disp}$ + ZPE) |
|---|---|---|
| 1 | TSR-01 | 1.21 |
| 2 | TSR-02 | 1.63 |
| 3 | TSR-03 | 4.11 |
| 4 | TSR-04 | 1.08 |
| 5 | TSR-05 | 0.00 |
| 6 | TSR-06 | 4.24 |
| 7 | TSR-07 | 3.92 |
| 8 | TSS-01 | 5.84 |
| 9 | TSS-02 | 5.75 |
| 10 | TSS-03 | 6.40 |
| 11 | TSS-04 | 10.88 |
| 12 | TSS-05 | 2.12 |
| 13 | TSS-06 | 9.80 |
| 14 | TSS-07 | 3.43 |
| 15 | TSS-08 | 4.63 |
| 16 | TSS-09 | 1.90 |

## Supplementary Note 1

The bis-guanidinium catalysts (*S*,*S*)-**1a** and (*R*,*R*)-**1a** were synthesized according to the reported literature.[1]

### Synthesis of sulfide substrate 2a[2]

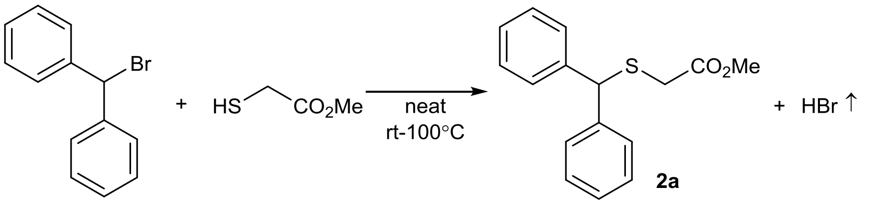


Methyl thioglycolate (447μL, 5.0 mmol, 1.0 equiv.) was slowly added to bromodiphenyl methane (1.359 g, 5.5 mmol, 1.1 equiv.) at room temperature. After the initial reaction had subsided, the mixture was heated to 100 °C for 2 h until there was no further evolution of HBr which was trapped and neutralized by passing over an aqueous saturated $NaHCO_3$ solution. The reaction mixture was then allowed to cool to room temperature and poured into $H_2O$ (10 mL) and extracted with EtOAc (25 mL×3). The combined organic layer was washed by brine and dried by $Na_2SO_4$, filtered and concentrated. The crude residue was subjected to purification by flash column chromatography (silica gel, Hexane: EtOAc, gradient from 100:1 to 20:1) to afford the product as pale yellow oil, 1.238g, 91% yield.

### Synthesis of sulfide substrates 2b-j, 2s[3]:

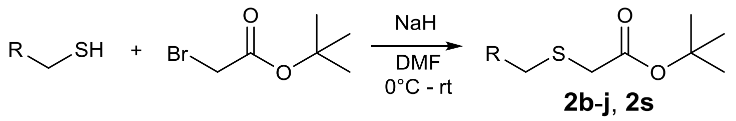


Take the synthesis of **2b** for example: benzyl mercaptan (621 mg, 5.0 mmol, 1.0 equiv.) was dissolved in dry DMF (25 mL) and the solution was cooled to 0 °C. NaH (60% suspension in oil) ( 220 mg, 5.5 mmol, 1.1 equiv.) was then added and the resulting solution was stirred for 30 min. *tert*-butyl bromoacetate (812 μL, 5.5 mmol, 1.1 equiv.) was then added and the solution

was stirred at room temperature for appropriate time. The reaction was quenched by slowly addition of $H_2O$ and DMF solvent was removed by vacuum pump. The resulting residue was subjected to purification by flash column chromatography (silica gel, Hexane: EtOAc, 20: 1) to afford the desired product with 80% to quantitative yield.

**Synthesis of sulfide substrates 2k-q**[4]

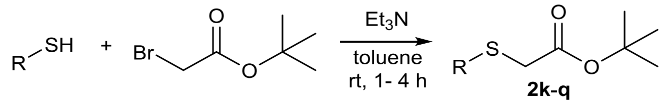


Take the synthesis of **2k** for example: a mixture of thiophenol (511 μL, 5.0 mmol), $Et_3N$ (697 μL, 5.0 mmol), *tert*-butyl bromoacetate (738 μL, 5.0 mmol), and toluene (5 mL) was stirred at room temperature for the appropriate time and monitored by TLC. After the addition of $H_2O$, the mixture was extracted with EtOAc. The combined organic layer was washed by brine and dried by $Na_2SO_4$, filtered and concentrated. The crude residue was subjected to purification by flash column chromatography (silica gel, Hexane: EtOAc, 20: 1) to afford the desired product with 90% to quantitative yield.

**Synthesis of sulfide substrates 2v**[5]

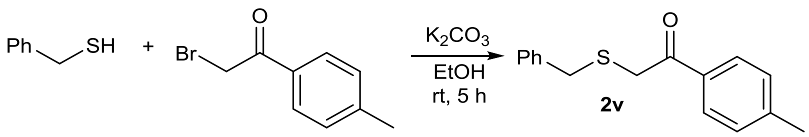


To a suspension of $K_2CO_3$ (1.38 g, 10.0 mmol, 2.0 equiv.) and 2-bromo-1-(*p*-tolyl)ethanone (959 mg, 4.5 mmol, 0.9 equiv.) in EtOH (15 mL), benzyl mercaptan (587 μL, 5.0 mmol, 1.0 equiv.) was added dropwise. After vigorously stirring for 5 h until the complete consumption of 2-bromo-1-(*p*-tolyl)ethanone, EtOAc (50 mL) was added and the reaction mixture was diluted with water (10 mL). The aqueous layer was extracted with EtOAc (2 × 50 mL) and the combined organic layer was washed by brine and dried by $Na_2SO_4$, filtered and concentrated.

The crude residue was subjected to purification by flash column chromatography (silica gel, Hexane: EtOAc, gradient from 50:1 to 20:1) to afford the product as a pale yellow solid.

## Supplementary Note 2

### Control Experiment and Mechanistic Study

With the hope of throwing some light upon mechanism, control experiments were carried out (Supplementary Table 1). In the comparison of entry 2 and 3, with 0.5 equivalent of tetrabutylammonium bisulfate ($TBAHSO_4$) as the additive and only 1 mol% of chiral bisguanidinium (*R*,*R*)-**1a**, the reaction still provided a high degree of stereocontrol (82% vs 92%) but at a relatively slow rate. Additionally, several organic sulfonic acids (Supplementary Table 1, entries 4 and 5) and inorganic acid (Supplementary Table 1, entries 6-8) were examined as additives. Indeed, except hydrochloric acid, they did accelerate the oxidation beyond the background reaction (Supplementary Table 1, entry 1). However, only employing sulfuric acid as additive allowed for the achievement of high enantioselectivity (Supplementary Table 1, entry 7). Further lowering the amount of aqueous $H_2SO_4$ to 5 mol% led to longer reaction time without affecting the enantioselectivity (Supplementary Table 1, entries 9 and 10) but the loss of enantioselectivity and reactivity were observed with only 1 mol% of aqueous $H_2SO_4$ (Supplementary Table 1, entry 11). Moreover, sulfuric acid itself cannot act as an activator towards hydrogen peroxide in the absence of molybdate salt (Supplementary Table 1, entry 12).

The performance of (*R*,*R*)-**1b** as the sole oxidant with different stoichiometry was examined in the asymmetric oxidation of sulfide **2a** (Supplementary Table 2). In the presence of one equivalent of (*R*,*R*)-**1b**, the reaction proceeded well to afford the product in 97% yield with a good enantioselectivity of 80%. However, the lower of its amount to 0.5 equivalent or 0.25 equivalent, a dramatic decrease of enantioselectivity was observed (Supplementary Table 2, entries 2 and 3) and the corresponding conversions were determined by the $^{1}H$ NMR spectra using the crude reaction mixture (Supplementary Figs 3-4).

The significant deterioration of enantioselectivity is probably ascribed to insufficient stereocontrol of the oxomonoperoxosulfato molybdenum dianion of (*R*,*R*)-**1b** in the transfer of second active oxygen. In other words, the second active oxygen of **A** should be out of the catalytic cycle in the presence of terminal oxidant hydrogen peroxide in order to maintain the dimeric structure which highly affect the enantiofacial discrimination process before the formation of **B**. Upon the completion of this stoichiometric reaction, the catalyst was recovered by running a flash silica column, but the reaction has shown loss of activity of the catalyst in the presence of $H_2O_2$ (Supplementary Table 2, entry 4). It was noteworthy that the catalyst can be remarkably regenerated or reactivated by the addition of 0.5 equivalent of $KHSO_4$ in the reaction to afford high enantioselectivity again (Supplementary Table 2, entry 5). Based on the aforementioned experimental results, a plausible mechanistic pathway was tentatively proposed (Supplementary Fig. 5).

## Supplementary Methods

**General information and materials**: $^{1}$H, $^{13}$C, $^{19}$F and $^{95}$Mo NMR spectra were recorded on Bruker Avance III 400 MHz BBFO1 spectrometer at 298 K. Chemical shifts are recorded as δ in units of parts per million (ppm). The residual solvent peak was used as an internal reference. For $^{95}$Mo NMR spectra, the chemical shift is reported relative to an external reference 2 M $Na_2MoO_4 \cdot 2H_2O$ solution in $D_2O$, assigned to 0 ppm. Infrared spectra were recorded on neat compounds or in dispersed KBr pellets using a Shimadzu IR Prestige21 FTIR spectrometer; only strong and selected absorbances ($\nu_{max}$) are reported. High resolution mass spectra (HRMS) were obtained on the Q-Tof Premier mass spectrometer (Waters Corporation). HRMS were reported in units of mass of charge ratio (*m/z*). Enantiomeric excess values were determined by high performance liquid chromatography (HPLC) analysis on Shimadzu LC-20AT and LC-2010CHT HPLC workstations. Melting point was recorded on OptiMelt (MPA100) melting point apparatus. Optical rotations were measured in $CHCl_3$ or MeOH using a 1 mL cell with a 1 dm path length on a JASCO P-1030 polarimeter with a sodium lamp of wavelength 589 nm and reported as follows: $[a]_D^{rt}$ (*c* = g/100 mL, solvent). X-ray crystallography analysis was performed on Bruker Kappa APEX II diffractometer. Flash chromatography separations were performed on Merck 60 (0.040 - 0.063mm) mesh silica gel. Analytical thin-layer chromatography (TLC) was performed on Merck 60 F254 silica gel plates. Visualization was performed using a UV lamp or potassium permanganate stain. Reagents were of analytical grade, obtained from commercial suppliers and used without further purification. Solvents were distilled over Na metal or $CaH_2$.

**Crystallographic Methods:** X-ray data collection and structural refinement. Intensity data for compounds (*R*,*R*)-**1b**, (*R*)-**3f**, (*S*)-**3o** and (*R*)-**4** were collected using a Bruker Kappa APEX II diffractometer with Mo/Kα X-ray source (0.71073 Å) at 103(2) K. The structure was solved and refined using the Bruker APEX 3 Software Package. The structure was solved by SHELXT

(SHELXL-2014/7 (Sheldrick, 2014)) and refined for all data by full-matrix least-squares methods on $F^2$. All data were corrected for absorption effects using the Multi-Scan method (SADABS). All non-hydrogen atoms were subjected to anisotropic refinement. The hydrogen atoms were generated geometrically and allowed to ride in their respective parent atoms; they were assigned appropriate isotropic thermal parameters and included in the structure-factor calculations.

**Crystal data and structure refinement of compounds** (*R*,*R*)-**1b**, (*R*)-**3f**, (*S*)-**3o** and (*R*)-**4**

(*R*,*R*)-**1b**: [$C_{94}H_{124}Mo_2N_6O_{14}S \cdot 2C_3H_7NO$(DMF)$\cdot C_4H_{10}O$($Et_2O$)], $M$ = 2006.24, monoclinic, *P 1 21 1*, a = 9.9398(8), $b$ = 30.471(3), c = 17.5604(15) Å, α = 90, β = 97.837(3)°, γ = 90°, $V$ = 5268.9(8) Å$^3$, $Z$ = 2, $\rho_{calcd}$ = 1.265 g/cm$^3$, μ(MoKα) = 0.324 mm$^{-1}$, $T$ = 103(2) K, Wavelength = 0.71073 Å, yellow plate. Bruker Kappa APEX II diffractometer; 20085 independent measured reflections, $F^2$ refinement, $R_1$(obs) = 0.0715, $wR_2$(all) = 0.1585, 12673 independent observed absorption-corrected reflections, 1426 parameters. Flack parameter (e.s.d.) = 0.00(2). Crystallographic data for this paper have been deposited at the Cambridge Crystallographic Data Centre under deposition number CCDC 1456990. *Handling of disorder*: for the molecule (*R*,*R*)-**1b**, four *tert*-butyl groups and one DMF molecule were disordered over two positions. The four t-Butyl groups (C5-C8, C11-C14, C40-C43, and C90-C93) were modelled with restraints (SAME, RIGU C39 > C43a C10 > C14a C4 > C8a C89 > C93a; SIMU 0.01 C40 > C43a C11 > C14a C5 > C8a C90 > C93a; ISOR 0.01 C5a > C8a) to restrain the groups having similar geometry and anisotropic displacement parameters. The DMF molecule (C102 to O17) was modeled with restraints (SAME; FLAT C102 > O17; FLAT C2a > O17a; RIGU C102 > O17a; SIMU 0.01 C102 > O17a) to restrain the two components having similar geometry and anisotropic displacement parameters.

($R$)-**3f**: [$C_{13}H_{17}ClO_3S$], $M$ = 288.77, orthorhombic, *P 21 21 21*, $a$ = 5.2790(5), $b$ = 11.5315(8), $c$ = 23.4325(19) Å, α = 90, β = 90°, γ = 90°, $V$ = 1426.4(2) Å$^3$, $Z$ = 4, $\rho_{calcd}$ = 1.345 g/cm$^3$, μ(MoKα) = 0.412 mm$^{-1}$, $T$ = 103(2) K, Wavelength = 0.71073 Å, colorless block. Bruker Kappa APEX II diffractometer; 3281 independent measured reflections, $F^2$ refinement, $R_1$(obs) = 0.0377, $wR_2$(all) = 0.0808, 3001 independent observed absorption-corrected reflections, 242 parameters. Flack parameter (e.s.d.) = 0.04(4). Crystallographic data for this paper have been deposited at the Cambridge Crystallographic Data Centre under deposition number CCDC 1456988. *Handling of disorder*: for the molecule ($R$)-**3f**, the ester group C8 to C13, O2 and O3 was disordered over two positions and modelled with restraints (SAME, RIGU O2 > C13a SIMU 0.02 O2 > C13a and SADI C8 S1 C8a S1) to restrain the two components having similar geometry and anisotropic displacement parameters.

($S$)-**3o**: [$C_{12}H_{15}BrO_3S$], $M$ = 319.21, monoclinic, *P 1 21 1*, $a$ = 26.8296(16), $b$ = 9.6925(5), $c$ = 10.5106(6) Å, α = 90, β = 101.2326(19)°, γ = 90°, $V$ = 2680.9(3) Å$^3$, $Z$ = 8, $\rho_{calcd}$ = 1.582 g/cm$^3$, μ(MoKα) = 3.216 mm$^{-1}$, $T$ = 103(2) K, Wavelength = 0.71073 Å, colorless plate. Bruker Kappa APEX II diffractometer; 22922 independent measured reflections, $F^2$ refinement, $R_1$(obs) = 0.0620, $wR_2$(all) = 0.1213, 10957 independent observed absorption-corrected reflections, 626 parameters. Flack parameter (e.s.d.) = 0.008(8). Crystallographic data for this paper have been deposited at the Cambridge Crystallographic Data Centre under deposition number CCDC 1456989. *Handling of disorder*: for the molecule ($S$)-**3o**, no disorder was included. A RIGU restraint was applied globally for handling of the data of ($S$)-**3o**. The crystal for ($S$)-**3o** is twinned with the twin law being 1 0 1 0 -1 0 0 0 -1 and the BASF was refined to 0.294. The restraint RIGU was applied globally to prevent two carbon atoms C20 and C22 to be NPD; if applied to the group containing C20 and C22 atoms, C22 would show very high ADP max/min ratio. The structure could not be solved in space group $C2$ or $C2$/m and the Flack parameter of 0.008(8) in $P2_1$ could be an indication that the space group is correct.

($R$)-**4**: [$C_{15}H_{15}NO_2S$], $M$ = 273.34, monoclinic, $P\ 1\ 21\ 1$, $a$ = 5.6324(3), $b$ = 26.1594(16), $c$ = 9.3139(5) Å, α = 90, β = 105.6796(19)°, γ = 90°, $V$ = 1321.25(13) Å$^3$, $Z$ = 4, $\rho_{calcd}$ = 1.374 g/cm$^3$, μ(MoKα) = 0.242 mm$^{-1}$, $T$ = 103(2) K, Wavelength = 0.71073 Å, colorless block. Bruker Kappa APEX II diffractometer; 8391 independent measured reflections, $F^2$ refinement, $R_1$(obs) = 0.0446, $wR_2$(all) = 0.0968, 7453 independent observed absorption-corrected reflections, 343 parameters. Flack parameter (e.s.d.) = 0.06(3). Crystallographic data for this paper have been deposited at the Cambridge Crystallographic Data Centre under deposition number CCDC 1456987. *Handling of disorder*: for the molecule ($R$)-**4**, no disorder was included.

**Characterization of catalysts** ($S$,$S$)-**1a**, ($R$,$R$)-**1a** and ($R$,$R$)-**1b**

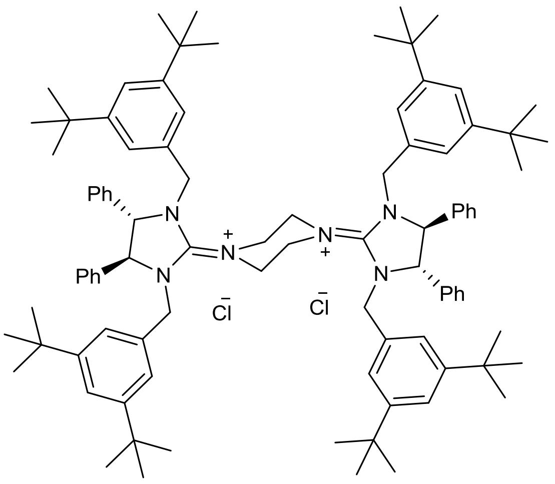


1,4-bis((4$S$,5$S$)-1,3-bis(3,5-di-*tert*-butylbenzyl)-4,5-diphenylimidazolidin-2-ylidene)piperazine-1,4-diium chloride ($S$,$S$)-**1a**: beige powder; 80% yield; mp: 209.3-211.5 °C; $[\alpha]_D^{22}$= –33.9 ($c$ 1.07, $CHCl_3$); $^1$H NMR (400 MHz, $CDCl_3$): $\delta$ = 7.30 (dd, $J$ = 5.0, 1.5 Hz, 12H), 7.20 (s, 4H), 7.05 (dd, $J$ = 6.5, 2.8 Hz, 8H), 6.96 (d, $J$ = 1.5 Hz, 8H), 5.19 (d, $J$ = 14.7 Hz, 4H), 4.82 (d, $J$ = 14.7 Hz, 4H), 4.73 (d, $J$ = 9.5 Hz, 4H), 4.48 (d, $J$ = 9.5 Hz, 4H), 4.32 (s, 1H), 1.14 (s, 72H); $^{13}$C NMR (100 MHz, $CDCl_3$): $\delta$ = 162.68, 151.30, 137.67, 131.89, 129.58, 129.16, 126.51, 123.37, 122.26, 70.39, 54.52, 48.99, 34.69, 31.37, 31.28; HRMS (ESI) calcd for $C_{94}H_{124}Cl_2N_6$ $m/z$ [M-2Cl$^-$]$^{2+}$: 668.4944; found: 668.4941; IR: 2962.66, 1597.06, 1527.62, 1454.33, 1361.74, 1018.41, 910.40, 740.64, 702.09 cm$^{-1}$.

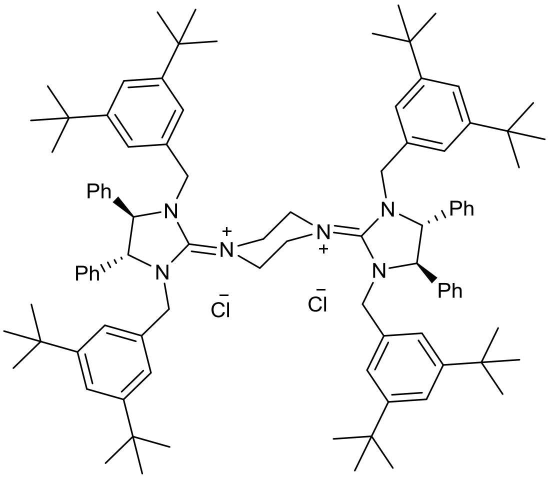

1,4-bis((4*R*,5*R*)-1,3-bis(3,5-di-tert-butylbenzyl)-4,5-diphenylimidazolidin-2-ylidene)piperazine-1,4-diium chloride (*R*,*R*)-**1a**: beige powder; 70% yield; mp: 209.6-212.2 °C; $[a]_D^{22}$= +31.9 (*c* 1.92, $CHCl_3$); $^1H$ NMR (400 MHz, $CDCl_3$): $\delta$ = 7.29 (dd, *J* = 5.1, 1.6 Hz, 12H), 7.20 (s, 4H), 7.08 – 7.00 (m, 8H), 6.95 (d, *J* = 1.6 Hz, 8H), 5.19 (d, *J* = 14.6 Hz, 4H), 4.80 (d, *J* = 14.7 Hz, 4H), 4.72 (d, *J* = 9.6 Hz, 4H), 4.47 (d, *J* = 9.5 Hz, 4H), 4.31 (s, 4H), 1.13 (s, 72H); $^{13}C$ NMR (100 MHz, $CDCl_3$): $\delta$ = 162.65, 151.27, 137.68, 131.89, 129.54, 129.12, 126.48, 123.37, 122.24, 70.32, 54.45, 48.98, 34.66, 31.25; HRMS (ESI) calcd for $C_{94}H_{124}Cl_2N_6$ *m/z* $[M\text{-}2Cl^-]^{2+}$: 668.4944; found: 668.4951; IR: 2962.66, 1600.92, 1519.91, 1454.33, 1361.74, 1280.73, 1199.72, 1153.43, 1018.41, 910.4, 736.81, 702.09 $cm^{-1}$.

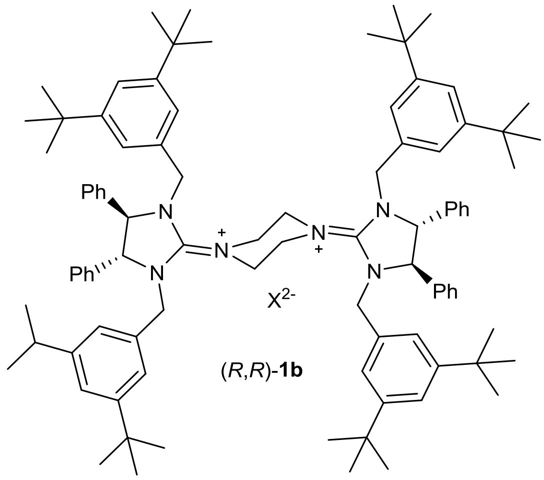


$X^{2-}$ = [(μ2-SO4){Mo2O2(μ2-O2)2(O2)2}]

(*R*,*R*)-**1b**: pale yellow solid; $^1H$ NMR (400 MHz, DMF-$d_7$): $\delta$ = 7.45 – 7.38 (m, 12H), 7.37 (s, 4H), 7.21 (dd, *J* = 6.3, 2.7 Hz, 8H), 7.07 (d, *J* = 1.4 Hz, 8H), 5.22 (d, *J* = 14.5 Hz, 4H), 4.89 (d, *J* = 14.5 Hz, 4H), 4.57 (s, 4H), 4.55 (t, *J* = 12.7 Hz, 8H), 1.17 (s, 72H); $^{13}C$ NMR (100 MHz, DMF-$d_7$): $\delta$ = 164.19, 152.34, 139.37, 133.86, 130.54, 130.01, 127.74, 124.64, 123.49, 70.74, 54.40, 50.25, 35.63, 31.94; $^{95}Mo$ NMR (26 MHz, DMF-$d_7$): $\delta$ = -199.29; IR: 2962.66, 1597.06, 1527.62, 1477.47, 1454.33, 1361.74, 1284.59, 1249.87, 1199.72, 1157.29, 1114.86, 1076.28, 1049.27, 1018.41, 972.12(Mo=O), 937.40, 918.12, 871.82(O-O), 759.55, 702.09, 663.51(Mo–($O_2$)), 590.22(Mo–($O_2$)).

**Characterization of sulfide substrates 2a-x**

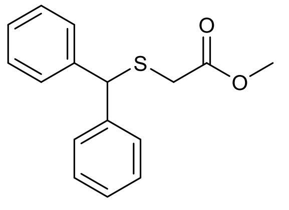

methyl 2-(benzhydrylthio)acetate(**2a**): pale yellow oil; $^1$H NMR (400 MHz, $CDCl_3$): $\delta$ = 7.48 – 7.40 (m, 4H), 7.33 (dt, $J$ = 7.7, 5.2 Hz, 4H), 7.28 – 7.19 (m, 2H), 5.40 (s, 1H), 3.68 (s, 3H), 3.10 (s, 2H); $^{13}$C NMR (100 MHz, $CDCl_3$): $\delta$ = 170.71, 140.29, 128.61, 128.43, 127.45, 54.16, 52.29, 33.48; HRMS (ESI) calcd for $C_{16}H_{16}O_2S$ *m/z* $[M+H]^+$: 273.0949; found: 273.0946; IR: 1732.08, 1597.06, 1492.90, 1450.47, 1276.88, 1195.87, 1130.29, 1006.84, 748.38, 702.09, 628.79, 586.36 cm$^{-1}$.

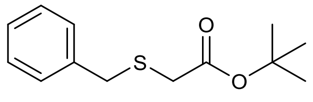

*tert*-butyl 2-(benzylthio)acetate(**2b**): Colorless oil; $^1$H NMR (400 MHz, $CDCl_3$): $\delta$ = 7.37 – 7.29 (m, 4H), 7.28 – 7.21 (m, 1H), 3.83 (s, 2H), 2.98 (s, 2H), 1.49 (s, 9H); $^{13}$C NMR (100 MHz, $CDCl_3$): $\delta$ = 169.53, 137.39, 129.09, 128.46, 127.12, 81.46, 36.07, 33.39, 27.97; HRMS (ESI) calcd for $C_{13}H_{18}O_2S$ *m/z* $[M+H]^+$: 239.1106; found: 239.1103; IR: 1728.22, 1492.90, 1454.33, 1392.61, 1369.46, 1296.16, 1257.59, 1122.57, 948.98, 852.54, 763.81, 702.09 cm$^{-1}$.

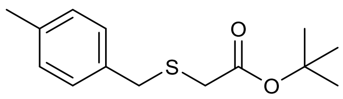

*tert*-butyl 2-((4-methylbenzyl)thio)acetate(**2c**): Colorless oil; $^1$H NMR (400 MHz, $CDCl_3$): $\delta$ = 7.22 (d, $J$ = 8.0 Hz, 2H), 7.13 (d, $J$ = 7.9 Hz, 2H), 3.80 (s, 2H), 2.98 (s, 2H), 2.34 (s, 3H), 1.49 (s, 9H); $^{13}$C NMR (100 MHz, $CDCl_3$): $\delta$ = 169.63, 136.80, 134.29, 129.17, 129.01, 81.45, 35.82, 33.42, 28.00, 21.06; HRMS (ESI) calcd for $C_{14}H_{20}O_2S$ *m/z* $[M+H]^+$: 253.1262; found: 253.1256; IR: 1728.22, 1512.19, 1454.33, 1392.61, 1369.46, 1296.16, 1257.59, 1122.57, 948.98, 817.82, 725.23 cm$^{-1}$.

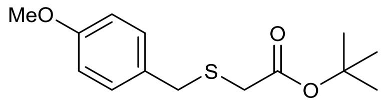


*tert*-butyl 2-((4-methoxybenzyl)thio)acetate(**2d**): Colorless oil; $^1$H NMR (400 MHz, $CDCl_3$): $\delta$ = 7.27 (d, $J$ = 8.3 Hz, 2H), 6.87 (d, $J$ = 8.3 Hz, 2H), 3.81 (s, 3H), 3.80 (s, 2H), 2.99 (s, 2H), 1.51 (s, 9H); $^{13}$C NMR (100 MHz, $CDCl_3$): $\delta$ = 169.63, 158.73, 130.21, 129.32, 113.88, 81.43, 55.22, 35.50, 33.33, 27.99; HRMS (ESI) calcd for $C_{14}H_{20}O_3S$ *m/z* $[M+H]^+$: 269.1211; found: 269.1208; IR: 1728.22,

1716.65, 1612.49, 1585.49, 1512.19, 1458.18, 1369.46, 1300.02, 1249.87, 1172.72, 1122.57, 1033.85, 948.98, 833.25 $cm^{-1}$.

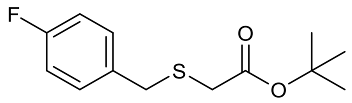

*tert*-butyl 2-((4-fluorobenzyl)thio)acetate(**2e**): Colorless oil; $^{1}H$ NMR (400 MHz, $CDCl_3$): $\delta$ = 7.36 – 7.27 (m, 2H), 7.06 – 6.96 (m, 2H), 3.80 (s, 2H), 2.96 (s, 2H), 1.48 (s, 9H); $^{13}C$ NMR (100 MHz, $CDCl_3$): $\delta$ = 169.47, 161.98 (d, $J$ = 245.6 Hz), 133.15 (d, $J$ = 3.2 Hz), 130.68 (d, $J$ = 8.1 Hz), 115.35 (d, $J$ = 21.5 Hz), 81.61, 35.34, 33.35, 28.00; $^{19}F$ NMR (376 MHz, $CDCl_3$): $\delta$ = -115.35; HRMS (ESI) calcd for $C_{13}H_{17}FO_2S$ $m/z$ $[M+H]^+$: 257.1012; found: 257.1010; IR: 1728.22, 1600.92, 1508.33, 1392.61, 1369.46, 1296.16, 1222.57, 1122.57, 948.98, 837.11, 759.95, 732.95 $cm^{-1}$.

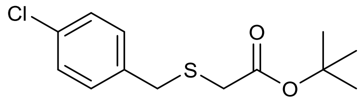

*tert*-butyl 2-((4-chlorobenzyl)thio)acetate(**2f**): Colorless oil; $^{1}H$ NMR (400 MHz, $CDCl_3$): $\delta$ = 7.32 – 7.22 (m, 4H), 3.78 (s, 2H), 2.95 (s, 2H), 1.48 (s, 9H); $^{13}C$ NMR (100 MHz, $CDCl_3$): $\delta$ = 169.39, 135.95, 132.97, 130.46, 128.63, 81.64, 35.37, 33.31, 27.98; HRMS (ESI) calcd for $C_{13}H_{17}ClO_2S$ $m/z$ $[M+H]^+$: 273.0716; found: 273.0710; IR:1728.22, 1489.05, 1454.33, 1369.46, 1296.16, 1257.59, 1122.57, 1091.71, 1014.56, 948.98, 833.25 $cm^{-1}$.

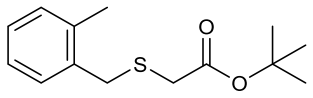

*tert*-butyl 2-((2-methylbenzyl)thio)acetate(**2g**): Colorless oil; $^{1}H$ NMR (400 MHz, $CDCl_3$): $\delta$ = 7.24 (d, $J$ = 6.6 Hz, 1H), 7.20 – 7.11 (m, 3H), 3.85 (s, 2H), 3.03 (s, 2H), 2.41 (s, 3H), 1.51 (s, 10H); $^{13}C$ NMR (100 MHz, $CDCl_3$): $\delta$ = 169.68, 136.85, 135.00, 130.69, 130.00, 127.48, 125.76, 81.49, 34.27, 33.90, 27.99, 19.07; HRMS (ESI) calcd for $C_{14}H_{20}O_2S$ $m/z$ $[M+H]^+$: 253.1262; found: 253.1270; IR: 1716.65, 1454.33, 1392.61, 1369.46, 1292.31, 1257.59, 1161.15, 1122.57, 948.98, 763.81, 732.95, 489.92 $cm^{-1}$.

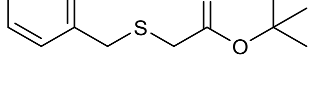

*tert*-butyl 2-((2-chlorobenzyl)thio)acetate(**2h**): Colorless oil; $^{1}H$ NMR (400 MHz, $CDCl_3$): $\delta$ = 7.42 – 7.33 (m, 2H), 7.24 – 7.15 (m,

2H), 3.95 (s, 2H), 3.05 (s, 2H), 1.49 (s, 9H); $^{13}$C NMR (100 MHz, CDCl$_3$): $\delta$ = 169.48, 135.21, 134.16, 131.04, 129.93, 128.62, 126.70, 81.66, 33.88, 33.75, 27.99; HRMS (ESI) calcd for $C_{13}H_{17}ClO_2S$ *m/z* [M+H]$^+$: 273.0716; found: 273.0720; IR: 1728.22, 1473.62, 1446.61, 1392.61, 1369.46, 1296.16, 1257.59, 1161.15, 1130.29, 1037.70, 948.98, 763.81, 740.67 cm$^{-1}$.

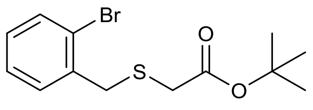


*tert*-butyl 2-((2-bromobenzyl)thio)acetate(**2i**): Colorless oil; $^1$H NMR (400 MHz, CDCl$_3$): $\delta$ = 7.57 (dd, *J* = 8.0, 1.1 Hz, 1H), 7.39 (dd, *J* = 7.6, 1.7 Hz, 1H), 7.27 (td, *J* = 7.5, 1.2 Hz, 1H), 7.12 (td, *J* = 7.7, 1.7 Hz, 1H), 3.96 (s, 2H), 3.05 (s, 2H), 1.49 (s, 9H); $^{13}$C NMR (100 MHz, CDCl$_3$): $\delta$ = 169.51, 136.86, 133.29, 131.04, 128.84, 127.34, 124.61, 81.68, 36.46, 33.87, 28.01; HRMS (ESI) calcd for $C_{13}H_{17}BrO_2S$ *m/z* [M+H]$^+$: 317.0211; found: 317.0202; IR: 1728.22, 1469.76, 1369.46, 1296.16, 1161.15, 1126.43, 1026.13, 948.98, 763.81, 736.81 cm$^{-1}$.

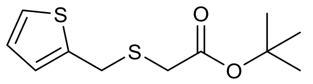


*tert*-butyl 2-((thiophen-2-ylmethyl)thio)acetate(**2j**): Pale yellow oil; $^1$H NMR (400 MHz, CDCl$_3$): $\delta$ = 7.22 (dd, *J* = 5.1, 1.2 Hz, 1H), 6.97 (dd, *J* = 3.4, 0.9 Hz, 1H), 6.92 (dd, *J* = 5.1, 3.5 Hz, 1H), 4.06 (s, 2H), 3.06 (s, 2H), 1.49 (s, 10H); $^{13}$C NMR (100 MHz, CDCl$_3$): $\delta$ = 169.36, 140.48, 126.89, 126.62, 125.19, 81.60, 33.41, 30.43, 27.98; HRMS (ESI) calcd for $C_{11}H_{16}O_2S_2$ *m/z* [M+H]$^+$: 245.0670; found: 245.0678; IR: 1728.22, 1392.61, 1369.46, 1296.16, 1257.59, 1165.00, 1134.14, 948.98, 852.54, 702.09 cm$^{-1}$.

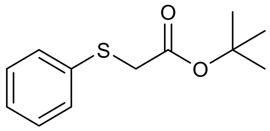


*tert*-butyl 2-(phenylthio)acetate(**2k**): Pale yellow oil; $^1$H NMR (400 MHz, CDCl$_3$): $\delta$ = 7.40 (d, *J* = 7.7 Hz, 2H), 7.28 (t, *J* = 7.5 Hz, 2H), 7.20 (dd, *J* = 8.3, 6.3 Hz, 1H), 3.55 (s, 2H), 1.39 (s, 9H); $^{13}$C NMR (100 MHz, CDCl$_3$): $\delta$ = 168.69, 135.23, 129.77, 128.82, 126.63, 81.76, 37.63, 27.79; HRMS (ESI) calcd for $C_{12}H_{16}O_2S$ *m/z* [M+H]$^+$: 225.0949; found: 225.0948; IR: 1728.22, 1585.49, 1481.33, 1392.61, 1369.46, 1292.31, 1257.59, 1165.00, 1134.14, 948.98, 848.68 740.67, 690.52, 489.92 cm$^{-1}$.

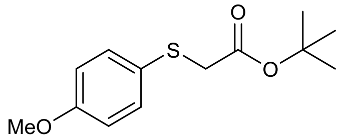

*tert*-butyl 2-((4-methoxyphenyl)thio)acetate(**2l**): Colorless oil; $^{1}$H NMR (400 MHz, $CDCl_3$): $\delta$ = 7.41 (d, $J$ = 8.8 Hz, 2H), 6.84 (d, $J$ = 8.8 Hz, 2H), 3.79 (s, 3H), 3.43 (s, 2H), 1.39 (s, 9H); $^{13}$C NMR (100 MHz, $CDCl_3$): $\delta$ = 169.03, 159.44, 133.90, 125.30, 114.51, 81.56, 55.30, 39.58, 27.88; HRMS (ESI) calcd for $C_{13}H_{18}O_3S$ *m/z* $[M+H]^+$: 255.1055; found: 255.1055; IR: 1728.22, 1593.20, 1492.90, 1462.04, 1392.61, 1369.46, 1288.45, 1246.02, 1172.72, 1130.29, 1029.99, 948.98, 829.39 $cm^{-1}$.

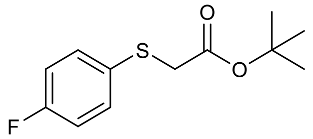

*tert*-butyl 2-((4-fluorophenyl)thio)acetate(**2m**): Colorless oil; $^{1}$H NMR (400 MHz, $CDCl_3$): $\delta$ = 7.51 – 7.32 (m, 2H), 7.06 – 6.89 (m, 2H), 3.47 (s, 2H), 1.38 (s, 9H); $^{13}$C NMR (100 MHz, $CDCl_3$): $\delta$ = 168.64, 162.18 (d, $J$ = 247.3 Hz), 133.11 (d, $J$ = 8.1 Hz), 130.04 (d, $J$ = 3.4 Hz), 115.97 (d, $J$ = 21.9 Hz), 81.82, 38.72, 27.82; $^{19}$F NMR (376 MHz, $CDCl_3$): $\delta$ = -114.44; HRMS (ESI) calcd for $C_{12}H_{15}FO_2S$ *m/z* $[M+H]^+$: 243.0855; found: 243.0853; IR: 1728.22, 1589.34, 1492.90, 1454.33, 1392.61, 1369.46, 1292.31, 1230.58, 1138.00, 1091.71, 948.98, 829.39, 628.79 $cm^{-1}$.

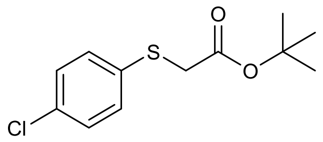

*tert*-butyl 2-((4-chlorophenyl)thio)acetate(**2n**): Colorless oil; $^{1}$H NMR (400 MHz, $CDCl_3$): $\delta$ = 7.33 (d, $J$ = 8.6 Hz, 2H), 7.25 (d, $J$ = 8.6 Hz, 2H), 3.52 (s, 2H), 1.40 (s, 9H); $^{13}$C NMR (100 MHz, $CDCl_3$): $\delta$ = 168.48, 133.81, 132.81, 131.20, 129.01, 82.08, 37.78, 27.86; HRMS (ESI) calcd for $C_{12}H_{15}ClO_2S$ *m/z* $[M+H]^+$: 259.0560; found: 259.0553; IR: 1728.22, 1477.47, 1454.33, 1392.61, 1369.46, 1292.31, 1257.59, 1138.00, 1095.57, 1010.70, 948.98, 817.82 $cm^{-1}$.

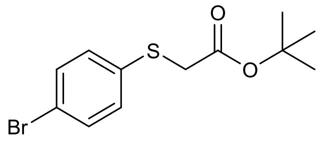

*tert*-butyl 2-((4-bromophenyl)thio)acetate(**2o**): Colorless oil; $^{1}$H NMR (400 MHz, $CDCl_3$): $\delta$ = 7.46 – 7.36 (m, 2H), 7.31 – 7.22 (m, 2H), 3.52 (s, 2H), 1.40 (s, 9H); $^{13}$C NMR (100 MHz, $CDCl_3$): $\delta$ = 168.41, 134.53, 131.91, 131.24, 120.62, 82.08, 37.55, 27.84; HRMS (ESI) calcd for $C_{12}H_{15}BrO_2S$ *m/z* $[M+H]^+$:

303.0054; found: 303.0051; IR: 1716.65, 1454.33, 1392.61, 1369.46, 1288.45, 1257.59, 1130.29, 1091.71, 1068.56, 1006.84, 948.98, 810.10 $cm^{-1}$.

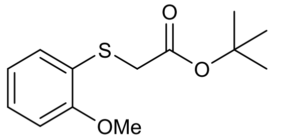


*tert*-butyl 2-((2-methoxyphenyl)thio)acetate(**2p**): Pale yellow oil; $^1$H NMR (400 MHz, $CDCl_3$): $\delta$ = 7.37 (d, $J$ = 7.6 Hz, 1H), 7.23 (t, $J$ = 7.5 Hz, 1H), 6.90 (t, $J$ = 7.6 Hz, 1H), 6.86 (d, $J$ = 8.2 Hz, 1H), 3.89 (s, 3H), 3.54 (s, 2H), 1.36 (s, 9H); $^{13}$C NMR (100 MHz, $CDCl_3$): $\delta$ = 168.86, 157.95, 131.56, 128.41, 122.71, 120.87, 110.59, 81.51, 55.73, 35.99, 27.82; HRMS (ESI) calcd for $C_{13}H_{18}O_3S$ *m/z* $[M+H]^+$: 255.1055; found: 255.1059; IR: 1728.22, 1712.79, 1581.63, 1454.33, 1392.61, 1369.46, 1292.31, 1246.02, 1172.72, 1122.57, 1072.42, 1026.13, 952.84, 848.68, 748.38, 682.80, 578.64 $cm^{-1}$.

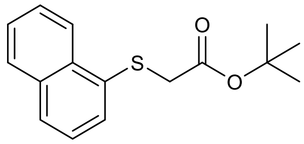


*tert*-butyl 2-(naphthalen-1-ylthio)acetate(**2q**): Pale yellow solid; mp: 43.6-45.3 °C; $^1$H NMR (400 MHz, $CDCl_3$): $\delta$ = 8.44 (d, $J$ = 8.4 Hz, 1H), 7.85 (d, $J$ = 8.1 Hz, 1H), 7.78 (d, $J$ = 8.2 Hz, 1H), 7.70 (d, $J$ = 7.1 Hz, 1H), 7.58 (t, $J$ = 7.1 Hz, 1H), 7.52 (t, $J$ = 7.2 Hz, 1H), 7.41 (t, $J$ = 7.7 Hz, 1H), 3.60 (s, 2H), 1.34 (s, 9H); $^{13}$C NMR (100 MHz, $CDCl_3$): $\delta$ = 168.70, 133.94, 133.10, 132.18, 130.02, 128.58, 128.27, 126.62, 126.25, 125.51, 125.11, 81.76, 38.19, 27.80; HRMS (ESI) calcd for $C_{16}H_{18}O_2S$ *m/z* $[M+H]^+$: 275.1106; found: 275.1100; IR: 1728.22, 1566.20, 1504.48, 1454.33, 1392.61, 1369.46, 1296.16, 1265.30, 1134.14, 948.98, 798.53, 771.53, 740.67, 702.09 $cm^{-1}$.

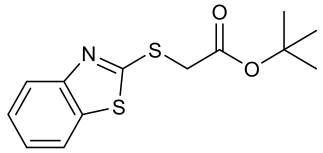


*tert*-butyl 2-(benzo[d]thiazol-2-ylthio)acetate(**2r**)[3]: Pale yellow oil; $^1$H NMR (400 MHz, $CDCl_3$): $\delta$ = 7.84 (d, $J$ = 8.1 Hz, 1H), 7.75 (d, $J$ = 8.0 Hz, 1H), 7.41 (t, $J$ = 7.7 Hz, 1H), 7.29 (t, $J$ = 7.6 Hz, 1H), 4.07 (s, 2H), 1.47 (s, 10H); $^{13}$C NMR (100 MHz, $CDCl_3$): $\delta$ = 167.18, 165.09, 152.94, 135.47, 126.03, 124.34, 121.57, 121.02, 82.57, 36.34, 27.91; HRMS (ESI) calcd for $C_{13}H_{15}NO_2S_2$ *m/z* $[M+H]^+$: 282.0622; found: 282.0618; IR: 2978.09, 2360.87, 1732.08, 1462.04, 1427.32, 1392.61, 1369.46, 1303.88, 1145.72, 1002.98, 948.98, 852.54, 756.10, 725.23, 489.92 $cm^{-1}$.

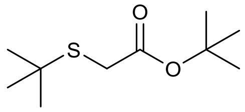

*tert*-butyl 2-(*tert*-butylthio)acetate(**2s**): Colorless oil; $^{1}$H NMR (400 MHz, $CDCl_3$): $\delta$ = 3.20 (s, 2H), 1.46 (s, 9H), 1.33 (s, 9H); $^{13}$C NMR (100 MHz, $CDCl_3$): $\delta$ = 170.38, 81.34, 42.81, 32.71, 30.75, 27.91; HRMS (ESI) calcd for $C_{10}H_{20}O_2S$ *m/z* $[M+H]^+$: 205.1262; found: 205.1259; IR: 1728.22, 1458.18, 1392.61, 1365.60, 1288.45, 1257.59, 1172.72, 1130.29, 952.84, 837.11, 763.81 $cm^{-1}$.

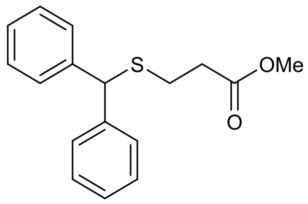

methyl 3-(benzhydrylthio)propanoate(**2t**)[2]: Pale yellow oil; 94% yield; $^{1}$H NMR (400 MHz, $CDCl_3$): $\delta$ = 7.43 (d, *J* = 7.5 Hz, 4H), 7.38 – 7.28 (m, 4H), 7.28 – 7.18 (m, 2H), 5.21 (s, 1H), 3.68 (s, 3H), 2.69 (dd, *J* = 11.2, 4.0 Hz, 2H), 2.55 (dd, *J* = 11.0, 3.9 Hz, 2H); $^{13}$C NMR (100 MHz, $CDCl_3$): $\delta$ = 172.22, 141.03, 128.55, 128.25, 127.24, 54.24, 51.72, 34.09, 27.14; HRMS (ESI) calcd for $C_{17}H_{18}O_2S$ *m/z* $[M+H]^+$: 287.1106; found: 287.1106; IR: 1728.22, 1600.92, 1492.90, 1446.61, 1435.04, 1357.89, 1246.02, 1199.72, 1172.72, 1076.28, 1029.99, 979.84, 829.39, 748.38, 702.09, 628.79, 586.36 $cm^{-1}$.

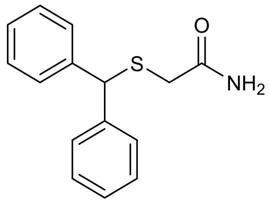

3-(benzhydrylthio)propanamide(**2u**)[6,7]: White solid; mp: 111.0-112.2 °C; $^{1}$H NMR (400 MHz, $CD_3OD$): $\delta$ = 7.42 (d, *J* = 7.7 Hz, 4H), 7.28 (t, *J* = 7.6 Hz, 4H), 7.20 (t, *J* = 7.3 Hz, 2H), 5.35 (s, 1H), 3.03 (s, 2H); $^{13}$C NMR (100 MHz, $CD_3OD$): $\delta$ = 174.60, 142.00, 129.52, 129.36, 128.32, 55.30, 36.00; HRMS (ESI) calcd for $C_{15}H_{15}NOS$ *m/z* $[M+H]^+$: 258.0953; found: 258.0958; IR: 3360.00, 1643.35, 1631. 78, 1489.05, 1373.32, 1080.14, 921.97, 698.23 $cm^{-1}$.

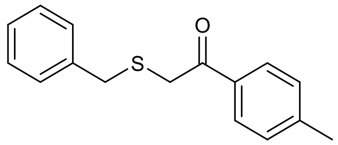

2-(benzylthio)-1-(p-tolyl)ethanone(**2v**): Pale yellow solid; mp: 69.4-70.5 °C; $^{1}$H NMR (400 MHz, $CDCl_3$): $\delta$ = 7.84 (d, *J* = 8.2 Hz, 2H), 7.43 – 7.29 (m, 4H), 7.29 – 7.21 (m, 3H), 3.76 (s, 2H), 3.66 (s, 2H), 2.42 (s, 3H); $^{13}$C NMR (100 MHz, $CDCl_3$): $\delta$ = 194.13, 144.17, 137.35, 132.89, 129.30, 129.23, 128.78, 128.47, 127.15, 36.08, 35.83, 21.63; HRMS (ESI) calcd for $C_{16}H_{16}OS$ *m/z* $[M+H]^+$: 257.1000; found:

257.1000; IR: 1670.35, 1604.77, 1492.90, 1454.33, 1419.61, 1280.73, 1184.29, 1014.56, 837.11, 806.25, 771.53, 702.09, 551.64 cm$^{-1}$.

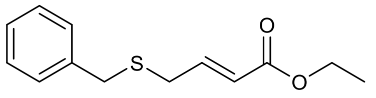


(*E*)-ethyl 4-(benzylthio)but-2-enoate(**2w**)[8,9]: Pale yellow oil; $^{1}$H NMR (400 MHz, $CDCl_3$): $\delta$ = 7.36 – 7.28 (m, 4H), 7.27 – 7.22 (m, 1H), 6.95 – 6.77 (m, 1H), 5.84 (dt, *J* = 15.5, 1.3 Hz, 1H), 4.21 (q, *J* = 7.1 Hz, 2H), 3.66 (s, 2H), 3.11 (dd, *J* = 7.4, 1.3 Hz, 2H), 1.31 (t, *J* = 7.1 Hz, 3H); $^{13}$C NMR (100 MHz, $CDCl_3$): $\delta$ = 166.02, 143.39, 137.54, 128.98, 128.56, 127.16, 123.09, 60.44, 35.22, 31.86, 14.21; HRMS (ESI) calcd for $C_{13}H_{16}O_2S$ *m/z* $[M+H]^+$: 237.0949; found: 237.0951; IR: 3028.24, 1712.79, 1651.07, 1492.90, 1454.33, 1369.46, 1315.45, 1265.30, 1195.87, 1149.57, 1041.56, 979.84, 860.25, 748.38, 702.09 cm$^{-1}$.

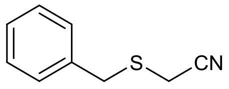


2-(benzylthio)acetonitrile(**2x**)[10]: Pale yellow oil; $^{1}$H NMR (400 MHz, $CDCl_3$): $\delta$ = 7.30 – 7.23 (m, 4H), 7.23 – 7.18 (m, 1H), 3.82 (s, 2H), 2.97 (s, 2H); $^{13}$C NMR (100 MHz, $CDCl_3$): $\delta$ = 135.64, 129.00, 128.83, 127.78, 116.19, 35.98, 15.84; HRMS (ESI) calcd for $C_9H_9NS$ *m/z* $[M+H]^+$:164.0534; found: 164.0548; IR: 2245.14, 1955.82, 1600.92, 1492.90, 1454.33, 1396.46, 1249.87, 1230.58, 1184.29, 1072.42, 1029.99, 921.97, 894.97, 771.53, 725.23, 702.09, 675.09, 563.21 cm$^{-1}$.

**Characterization of sulfoxide products 3a-y and 4**

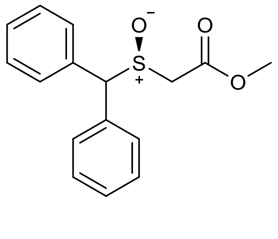


(*S*)-methyl 2-(benzhydrylsulfinyl)acetate(**3a**): White solid; 99% yield; mp: 103.8-105.5 °C; $^{1}$H NMR (400 MHz, $CDCl_3$): $\delta$ = 7.50 (dd, *J* = 11.6, 7.9 Hz, 4H), 7.44 – 7.28 (m, 6H), 5.21 (s, 1H), 3.74 (s, 3H), 3.48 (dd, *J* = 45.5, 14.0 Hz, 2H); $^{13}$C NMR (100 MHz, $CDCl_3$): $\delta$ = 165.78, 135.21, 133.77, 129.55, 129.27, 128.78, 128.75, 128.56, 128.51, 71.55, 54.02, 52.74; $[\alpha]_D^{22}$= +19.24 (*c* 5.51, MeOH); HRMS (ESI) calcd for $C_{16}H_{16}O_3S$ *m/z* $[M+H]^+$: 289.0898; found: 289.0892; IR: 1732.08, 1492.90, 1361.74, 1234.44, 1176.58, 1045.42, 975.98, 702.09 cm$^{-1}$; HPLC analysis: Chiralcel AD-H (Hex/IPA = 50/50, 1.0 mL/min, 230 nm, 22°C), 7.0 (major), 12.6 min, 94% *ee*.

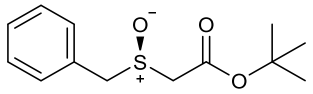

($R$)-*tert*-butyl 2-(benzylsulfinyl)acetate(**3b**): White solid; 90% yield; mp: 83.8-85.1 °C; $^1$H NMR (400 MHz, $CDCl_3$): $\delta$ = 7.38 (t, $J$ = 6.7 Hz, 3H), 7.35 – 7.29 (m, 2H), 4.16 (dd, $J$ = 62.7, 13.0 Hz, 2H), 3.46 (q, $J$ = 14.0 Hz, 2H), 1.50 (s, 9H); $^{13}$C NMR (100 MHz, $CDCl_3$): $\delta$ = 164.33, 130.37, 129.12, 128.98, 128.59, 83.50, 57.69, 54.68, 28.03; $[\alpha]_D^{22}$= +18.77 ($c$ 5.03, MeOH); HRMS (ESI) calcd for $C_{13}H_{18}O_3S$ $m/z$ $[M+H]^+$: 255.1055; found: 255.1054; IR: 1735.93, 1454.33, 1396.46, 1276.88, 1257.59, 1161.15, 1029.99, 952.84, 767.67, 702.09, 416.62 cm$^{-1}$; HPLC analysis: Chiralcel AD-H (Hex/IPA = 90/10, 1.0 mL/min, 230 nm, 22°C), 9.8 (major), 16.4 min, 90% *ee*.

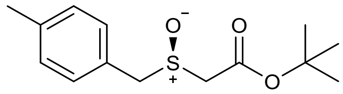

($R$)-*tert*-butyl 2-((4-methylbenzyl)sulfinyl)acetate(**3c**): White solid; 92% yield; mp: 99.8-102.0 °C; $^1$H NMR (400 MHz, $CDCl_3$): $\delta$ = 7.23 – 7.16 (m, 4H), 4.11 (dd, $J$ = 59.9, 13.0 Hz, 2H), 3.44 (q, $J$ = 14.0 Hz, 2H), 2.35 (s, 3H), 1.49 (s, 9H); $^{13}$C NMR (100 MHz, $CDCl_3$): $\delta$ = 164.37, 138.47, 130.22, 129.66, 125.91, 83.38, 57.40, 54.61, 28.00, 21.15; $[\alpha]_D^{22}$= +14.83 ($c$ 4.86, MeOH); HRMS (ESI) calcd for $C_{14}H_{20}O_3S$ $m/z$ $[M+H]^+$: 269.1211; found: 269.1211; IR: 1728.22, 1516.05, 1300.02, 1149.57, 1118.71, 1022.27, 956.69, 821.68, 736.81, 466.77 cm$^{-1}$; HPLC analysis: Chiralcel AD-H (Hex/IPA = 90/10, 1.0 mL/min, 230 nm, 22°C), 9.8 (major), 15.1 min, 92% *ee*.

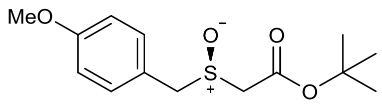

($R$)-*tert*-butyl 2-((4-methoxybenzyl)sulfinyl)acetate(**3d**): White solid; 96% yield; mp: 92.1-93.3 °C; $^1$H NMR (400 MHz, $CDCl_3$): $\delta$ = 7.27 (d, $J$ = 8.4 Hz, 2H), 6.91 (t, $J$ = 10.6 Hz, 2H), 4.12 (dd, $J$ = 64.6, 13.2 Hz, 2H), 3.82 (s, 3H), 3.45 (q, $J$ = 14.0 Hz, 2H), 1.51 (s, 9H); $^{13}$C NMR (100 MHz, $CDCl_3$): $\delta$ = 164.38, 159.87, 131.56, 120.84, 114.40, 83.39, 56.91, 55.27, 54.44, 28.01; $[\alpha]_D^{22}$= +16.13 ($c$ 5.43, MeOH); HRMS (ESI) calcd for $C_{14}H_{20}O_4S$ $m/z$ $[M+H]^+$: 285.1261; found: 285.1262; IR: 1728.22, 1612.49, 1516.05, 1465.90, 1392.61, 1369.46, 1303.88, 1253.73, 1176.58, 1149.57, 1118.71, 1033.85, 837.11, 732.95 cm$^{-1}$; HPLC analysis: Chiralcel AD-H (Hex/IPA = 90/10, 1.0 mL/min, 230 nm, 22°C), 13.9 (major), 21.3 min, 83% *ee*.

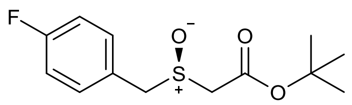

($R$)-*tert*-butyl 2-((4-fluorobenzyl)sulfinyl)acetate(**3e**): White solid; 94% yield; mp: 95.5-96.2 °C; $^1$H NMR (400 MHz, $CDCl_3$): $\delta$ = 7.35 – 7.27 (m, 2H), 7.07 (t, $J$ = 8.6 Hz, 2H), 4.11 (dd, $J$ = 76.1, 13.2 Hz, 2H), 3.53 – 3.35 (m, 2H), 1.49 (s, 9H);

$^{13}C$ NMR (100 MHz, $CDCl_3$): $\delta$ = 164.21, 162.92 (d, $J$ = 248.0 Hz), 132.11 (d, $J$ = 8.3 Hz), 124.92 (d, $J$ = 3.0 Hz), 115.94 (d, $J$ = 21.7 Hz), 83.56, 56.54, 54.60, 27.99; $^{19}F$ NMR (376 MHz, $CDCl_3$): $\delta$ = -112.91; $[\alpha]_D^{22}$= +29.96 (*c* 4.91, MeOH); HRMS (ESI) calcd for $C_{13}H_{17}FO_3S$ *m/z* $[M+H]^+$: 273.0961; found: 273.0952; IR: 1716.65. 1508.33, 1369.46, 1300.02, 1226.73, 1145.72, 1114.86, 1029.99, 840.96, 740.67, 528.50 $cm^{-1}$; HPLC analysis: Chiralcel AD-H (Hex/IPA = 90/10, 1.0 mL/min, 230 nm, 22°C), 11.2 (major), 17.5 min, 96% *ee*.

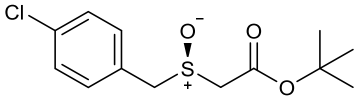

(*R*)-*tert*-butyl 2-((4-chlorobenzyl)sulfinyl)acetate(**3f**): White solid; 92% yield; mp: 84.1-85.3 °C; $^{1}H$ NMR (400 MHz, $CDCl_3$): $\delta$ = 7.36 (d, $J$ = 8.4 Hz, 2H), 7.26 (d, $J$ = 8.4 Hz, 2H), 4.10 (dd, $J$ = 76.9, 13.2 Hz, 2H), 3.50 – 3.36 (m, 1H), 1.49 (s, 9H); $^{13}C$ NMR (100 MHz, $CDCl_3$): $\delta$ = 164.16, 134.74, 131.68, 129.11, 127.59, 83.60, 56.63, 54.65, 27.98; $[\alpha]_D^{22}$= +43.89 (*c* 5.21, MeOH); HRMS (ESI) calcd for $C_{13}H_{17}ClO_3S$ *m/z* $[M+H]^+$: 289.0665; found: 289.0668; IR: 1724.36, 1712.79, 1597.06, 1492.90, 1454.33, 1369.46, 1303.88, 1261.45, 1145.72, 1095.57, 1018.41, 956.69, 914.26, 840.96, 740.67, 702.09, 671.23 $cm^{-1}$; HPLC analysis: Chiralcel AD-H (Hex/IPA = 90/10, 1.0 mL/min, 230 nm, 22°C), 11.9 (major), 18.5 min, 91% *ee*.

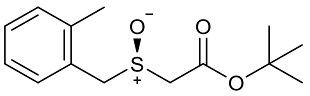

(*R*)-*tert*-butyl 2-((2-methylbenzyl)sulfinyl)acetate(**3g**): Colourless oil; 98% yield; $^{1}H$ NMR (400 MHz, $CDCl_3$): $\delta$ = 7.34 – 7.09 (m, 4H), 4.20 (dd, $J$ = 65.0, 12.9 Hz, 2H), 3.55 (dd, $J$ = 31.3, 13.9 Hz, 2H), 2.39 (s, 3H), 1.47 (s, 9H); $^{13}C$ NMR (100 MHz, $CDCl_3$): $\delta$ = 164.37, 137.70, 131.29, 130.86, 128.77, 128.31, 126.54, 83.48, 56.89, 55.84, 28.01, 19.82; $[\alpha]_D^{22}$= +55.71 (*c* 5.15, MeOH); HRMS (ESI) calcd for $C_{14}H_{20}O_3S$ *m/z* $[M+H]^+$: 269.1211; found: 269.1210; IR: 1732.08, 1712.79, 1492.90, 1454.33, 1392.61, 1369.46, 1288.45, 1261.45, 1161.15, 1041.56, 952.84, 910.40, 837.11, 767.67, 482.20 $cm^{-1}$; HPLC analysis: Chiralcel AD-H (Hex/IPA = 90/10, 1.0 mL/min, 230 nm, 22°C), 8.2 (major), 15.6 min, 92% *ee*.

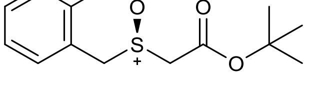

(*R*)-*tert*-butyl 2-((2-chlorobenzyl)sulfinyl)acetate(**3h**): Colourless oil; 98% yield; $^{1}H$ NMR (400 MHz, $CDCl_3$): $\delta$ = 7.47 – 7.40 (m, 2H), 7.35 – 7.27 (m, 2H), 4.32 (dd, $J$ = 96.1, 12.9 Hz, 2H), 3.58 (dd, $J$ = 43.2, 13.9 Hz, 2H), 1.50 (s, 9H); $^{13}C$ NMR (100 MHz, $CDCl_3$): $\delta$ = 164.19, 134.66, 132.77, 130.04, 129.90, 127.99, 127.32, 83.57, 56.18, 55.96, 28.00;

$[\alpha]_D^{22}$= +47.96 (*c* 5.55, MeOH); HRMS (ESI) calcd for $C_{13}H_{17}ClO_3S$ *m/z* $[M+H]^+$: 289.0665; found: 289.0665; IR: 1728.22, 1712.79, 1473.62, 1446.61, 1392.61, 1369.46, 1296.16, 1261.45, 1157.29, 1053.13, 952.84, 910.40, 840.96, 763.81, 682.80, 578.64 $cm^{-1}$; HPLC analysis: Chiralcel AD-H (Hex/IPA = 90/10, 1.0 mL/min, 230 nm, 22°C), 10.4 (major), 32.7 min, 93% *ee*.

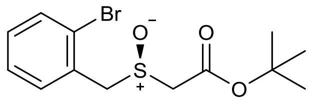

(*R*)-*tert*-butyl 2-((2-bromobenzyl)sulfinyl)acetate(**3i**): Pale yellow oil; 99% yield; $^1$H NMR (400 MHz, $CDCl_3$): $\delta$ = 7.61 (dd, *J* = 8.0, 1.1 Hz, 1H), 7.43 (dd, *J* = 7.6, 1.7 Hz, 1H), 7.32 (td, *J* = 7.5, 1.2 Hz, 1H), 7.21 (td, *J* = 7.7, 1.7 Hz, 1H), 4.33 (dd, *J* = 99.7, 12.9 Hz, 2H), 3.59 (dd, *J* = 46.8, 13.8 Hz, 2H), 1.50 (s, 9H); $^{13}$C NMR (100 MHz, $CDCl_3$): $\delta$ = 164.15, 133.22, 132.78, 130.21, 129.83, 127.93, 125.10, 83.56, 58.53, 56.20, 28.00; $[\alpha]_D^{22}$= +45.04 (*c* 6.6, MeOH); HRMS (ESI) calcd for $C_{13}H_{17}BrO_3S$ *m/z* $[M+H]^+$: 333.0160; found: 333.0170; IR: 1728.22, 1712.79, 1566.20, 1469.76, 1392.61, 1369.46, 1296.16, 1261.45, 1157.29, 1045.42, 1029.99, 952.84, 910.40, 837.11, 763.81, 659.66 $cm^{-1}$; HPLC analysis: Chiralcel AD-H (Hex/IPA = 90/10, 1.0 mL/min, 230 nm, 22°C), 10.7 (major), 40.1 min, 93% *ee*.

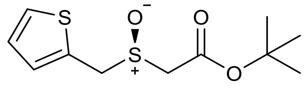

(*S*)-*tert*-butyl 2-((thiophen-2-ylmethyl)sulfinyl)acetate(**3j**): White solid; 94% yield; mp: 67.7-68.4 °C; $^1$H NMR (400 MHz, $CDCl_3$): $\delta$ = 7.33 (dd, *J* = 5.1, 1.2 Hz, 1H), 7.09 (d, *J* = 2.8 Hz, 1H), 7.06 (dd, *J* = 5.0, 3.5 Hz, 1H), 4.37 (dd, *J* = 62.5, 14.1 Hz, 2H), 3.47 (q, *J* = 14.2 Hz, 2H), 1.50 (s, 9H); $^{13}$C NMR (100 MHz, $CDCl_3$): $\delta$ = 164.19, 129.36, 129.28, 127.59, 127.10, 83.59, 54.30, 51.72, 28.03; $[\alpha]_D^{22}$= +18.2 (*c* 4.80, MeOH); HRMS (ESI) calcd for $C_{11}H_{16}O_3S_2$ *m/z* $[M+H]^+$: 261.0619; found: 261.0611; IR: 1728.22, 1712.79, 1454.33, 1392.61, 1369.46, 1288.45, 1257.59, 1161.15, 1041.56, 952.84, 906.54, 840.96, 582.50, 474.49 $cm^{-1}$; HPLC analysis: Chiralcel AD-H (Hex/IPA = 90/10, 1.0 mL/min, 230 nm, 22°C), 10.4 (major), 17.3 min, 89% *ee*.

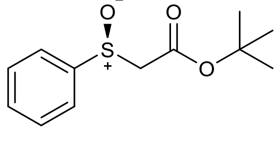

(*S*)-*tert*-butyl 2-(phenylsulfinyl)acetate(**3k**): Yellow oil; 91% yield; $^1$H NMR (400 MHz, $CDCl_3$): $\delta$ = 7.70 (dd, *J* = 6.7, 3.0 Hz, 2H), 7.54 (dd, *J* = 6.5, 2.7 Hz, 3H), 3.70 (dd, *J* = 80.0, 13.7 Hz, 2H), 1.39 (s, 9H); $^{13}$C NMR (100 MHz, $CDCl_3$): $\delta$ = 163.77, 143.29, 131.65, 129.30, 124.42, 83.25, 62.61, 27.88; $[\alpha]_D^{22}$= –122.64 (*c* 4.31, MeOH); HRMS (ESI) calcd for $C_{12}H_{16}O_3S$ *m/z* $[M+H]^+$: 241.0898; found: 241.0900; IR: 1728.22.

1712.79, 1477.47, 1446.61, 1392.61, 1369.46, 1296.16, 1261.45, 1157.29, 1126.43, 1049.28, 952.84, 902.69, 840.96, 690.52, 667.37 $cm^{-1}$; HPLC analysis: Chiralcel OD-H (Hex/IPA = 90/10, 1.0 mL/min, 254 nm, 22°C), 8.9, 11.6 (major) min, 86% *ee*.

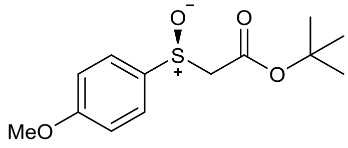


(*S*)-*tert*-butyl 2-((4-methoxyphenyl)sulfinyl)acetate(**3l**): Pale yellow oil; 94% yield; $^1$H NMR (400 MHz, $CDCl_3$): $\delta$ = 7.64 (d, $J$ = 8.7 Hz, 2H), 7.03 (d, $J$ = 8.7 Hz, 2H), 3.86 (s, 3H), 3.70(dd, $J$ = 108, 13.6 Hz, 2H), 1.39 (s, 9H); $^{13}$C NMR (100 MHz, $CDCl_3$): $\delta$ = 163.84, 162.47, 134.10, 126.53, 114.78, 83.11, 62.68, 55.55, 27.89; $[\alpha]_D^{22}$= –91.45 (*c* 4.97, MeOH); HRMS (ESI) calcd for $C_{13}H_{18}O_4S$ *m/z* $[M+H]^+$: 271.1004; found: 271.1007; IR: 1728.22, 1712.79, 1593.20, 1496.76, 1462.04, 1392.61, 1369.46, 1296.16, 1257.59, 1161.15, 1122.57, 1087.85, 1029.99, 952.84, 902.69, 833.25, 798.53, 756.10, 667.37 $cm^{-1}$; HPLC analysis: Chiralcel OB-H (Hex/IPA = 90/10, 1.0 mL/min, 230 nm, 22°C), 11.8 (major), 14.9 min, 79% *ee*.

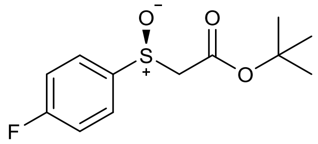


(*S*)-*tert*-butyl 2-((4-fluorophenyl)sulfinyl)acetate(**3m**): White solid; 93% yield; mp: 95.1-95.8 °C; $^1$H NMR (400 MHz, $CDCl_3$): $\delta$ = 7.70 (ddd, $J$ = 8.4, 5.1, 1.3 Hz, 2H), 7.25 – 7.17 (m, 2H), 3.69 (ddd, $J$ = 87.8, 13.7, 1.0 Hz, 2H), 1.39 (s, 9H); $^{13}$C NMR (100 MHz, $CDCl_3$): $\delta$ = 165.92, 163.50 (d, $J$ = 18.4 Hz), 138.73 (d, $J$ = 3.0 Hz), 126.86 (d, $J$ = 9.0 Hz), 116.63 (d, $J$ = 22.6 Hz), 83.38, 62.63, 27.86; $^{19}$F NMR (376 MHz, $CDCl_3$): $\delta$ = -107.41; $[\alpha]_D^{22}$= –115.46 (*c* 4.74, MeOH); HRMS (ESI) calcd for $C_{12}H_{15}FO_3S$ *m/z* $[M+H]^+$: 259.0804; found: 259.0804; IR: 1724.36, 1585.49, 1492.90, 1469.76, 1369.46, 1296.16, 1257.59, 1215.15, 1157.29, 1080.14, 1037.70, 837.11 $cm^{-1}$; HPLC analysis: Chiralcel OD-H (Hex/IPA = 90/10, 1.0 mL/min, 230 nm, 22°C), 8.7, 9.7 (major) min, 89% *ee*.

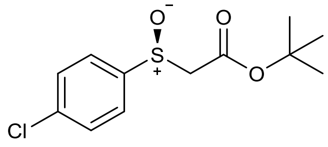


(*S*)-*tert*-butyl 2-((4-chlorophenyl)sulfinyl)acetate(**3n**): Pale yellow solid; 93% yield; mp: 115.4-116.9 °C; $^1$H NMR (400 MHz, $CDCl_3$): $\delta$ = 7.65 (d, $J$ = 8.5 Hz, 2H), 7.52 (d, $J$ = 8.5 Hz, 2H), 3.69 (dd, $J$ = 77.7, 13.8 Hz, 2H), 1.41 (s, 9H); $^{13}$C NMR (100 MHz, $CDCl_3$): $\delta$ = 163.58, 141.86, 137.94, 129.61, 125.88, 83.54, 62.58, 27.92; $[\alpha]_D^{22}$= –136.61 (*c* 4.96, MeOH); HRMS (ESI) calcd for $C_{12}H_{15}ClO_3S$ *m/z* $[M+H]^+$: 275.0509; found: 275.0507; IR: 1724.36, 1573.91, 1477.47, 1369.46, 1157.29, 1087.85, 1037.70,

1010.70, 825.53, 740.67 $cm^{-1}$; HPLC analysis: Chiralcel OB-H (Hex/IPA = 90/10, 1.0 mL/min, 230 nm, 22°C), 8.1 (major), 9.4 min, 90% *ee*.

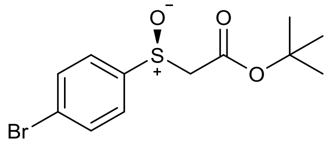

(*S*)-*tert*-butyl 2-((4-bromophenyl)sulfinyl)acetate(**3o**): Pale yellow solid; 95% yield; mp: 99.2-100.4 °C; $^1$H NMR (400 MHz, $CDCl_3$): $\delta$ = 7.68 (d, $J$ = 8.6 Hz, 2H), 7.58 (d, $J$ = 8.6 Hz, 2H), 3.69 (dd, $J$ = 75.2, 13.8 Hz, 2H), 1.42 (s, 9H); $^{13}$C NMR (100 MHz, $CDCl_3$): $\delta$ = 163.57, 142.47, 132.54, 126.19, 126.02, 83.56, 62.51, 27.92; $[\alpha]_D^{22}$= –123.13 (*c* 4.95, MeOH); HRMS (ESI) calcd for $C_{12}H_{15}BrO_3S$ *m/z* $[M+H]^+$: 319.0004; found: 319.0013; IR: 1724.36, 1570.06, 1469.76, 1369.46, 1300.02, 1257.59, 1149.57, 1045.42, 1006.84, 821.68, 721.38 $cm^{-1}$; HPLC analysis: Chiralcel OB-H (Hex/IPA = 90/10, 1.0 mL/min, 230 nm, 22°C), 9.0 (major), 10.4 min, 91% *ee*.

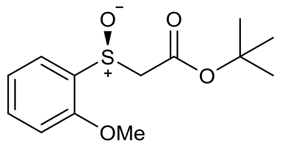

(*S*)-*tert*-butyl 2-((2-methoxyphenyl)sulfinyl)acetate(**3p**): Pale yellow oil; 99% yield; $^1$H NMR (400 MHz, $CDCl_3$): $\delta$ = 7.81 (dd, $J$ = 7.7, 1.7 Hz, 1H), 7.50 – 7.42 (m, 1H), 7.19 (td, $J$ = 7.6, 0.8 Hz, 1H), 6.93 (d, $J$ = 8.2 Hz, 1H), 3.89 (s, 3H), 3.75 (dd, $J$ = 136.5, 13.7 Hz, 2H), 1.41 (s, 9H); $^{13}$C NMR (100 MHz, $CDCl_3$): $\delta$ = 164.34, 155.00, 132.37, 130.29, 125.81, 121.64, 110.53, 82.80, 58.46, 55.75, 27.92; $[\alpha]_D^{22}$= –303.09 (*c* 5.28, MeOH); HRMS (ESI) calcd for $C_{13}H_{18}O_4S$ *m/z* $[M+H]^+$: 271.1004; found: 271.1004; IR: 1728.22, 1712.79, 1585.49, 1477.47, 1392.61, 1276.88, 1161.15, 1122.57, 1072.42, 1041.56, 1018.41, 952.84, 902.69, 837.11, 759.95, 663.51 $cm^{-1}$; HPLC analysis: Chiralcel OD-H (Hex/IPA = 90/10, 1.0 mL/min, 230 nm, 22°C), 11.3, 12.7 (major) min, 89% *ee*.

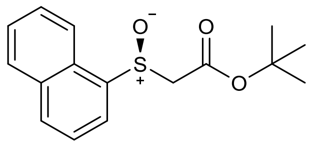

(*S*)-*tert*-butyl 2-(naphthalen-1-ylsulfinyl)acetate(**3q**): Pale yellow solid; 97% yield; mp: 95.6-96.3 °C; $^1$H NMR (400 MHz, $CDCl_3$): $\delta$ = 8.18 (dd, $J$ = 7.3, 1.0 Hz, 1H), 8.01 (dd, $J$ = 12.1, 5.1 Hz, 2H), 7.94 (dd, $J$ = 6.9, 2.6 Hz, 1H), 7.72 – 7.63 (m, 1H), 7.63 – 7.53 (m, 2H), 3.78 (dd, $J$ = 58.6, 13.8 Hz, 2H), 1.37 (s, 9H); $^{13}$C NMR (100 MHz, $CDCl_3$): $\delta$ = 164.11, 138.89, 133.41, 131.72, 129.05, 128.71, 127.50, 126.78, 125.59, 123.51, 121.49, 83.20, 61.69, 27.82; $[\alpha]_D^{22}$=–280.80 (*c* 5.60, MeOH); HRMS (ESI) calcd for $C_{16}H_{18}O_3S$ *m/z* $[M+H]^+$: 291.1055; found: 291.1058; IR: 1728.22, 1504.48, 1454.33, 1369.46, 1296.16, 1161.15,

1114.86, 1053.13, 952.84, 898.83, 837.11, 802.39, 771.53, 736.81, 702.09 $cm^{-1}$; HPLC analysis: Chiralcel OD-H (Hex/IPA = 90/10, 1.0 mL/min, 230 nm, 22°C), 12.4 (major), 28.9 min, 83% *ee*.

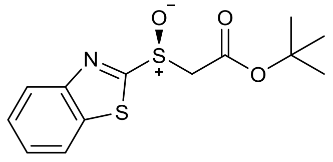

(*S*)-*tert*-butyl 2-(benzo[d]thiazol-2-ylsulfinyl)acetate(**3r**): Pale yellow solid; 79% yield; mp: 77.9-78.8 °C; $^1H$ NMR (400 MHz, $CDCl_3$): $\delta$ = 8.07 (d, $J$ = 8.2 Hz, 1H), 8.02 (d, $J$ = 7.9 Hz, 1H), 7.58 (t, $J$ = 7.6 Hz, 1H), 7.51 (t, $J$ = 7.6 Hz, 1H), 4.09 (dd, $J$ = 43.2, 14.3 Hz, 2H), 1.46 (s, 9H); $^{13}C$ NMR (100 MHz, $CDCl_3$): $\delta$ = 176.25, 162.99, 153.69, 136.20, 127.04, 126.38, 124.08, 122.31, 84.03, 61.60, 27.93; $[\alpha]_D^{22}$= –42.92 (*c* 4.72, MeOH); HRMS (ESI) calcd for $C_{13}H_{15}NO_3S_2$ *m/z* $[M+H]^+$: 298.0572; found: 298.0572; IR: 1728.22, 1458.18, 1392.61, 1369.46, 1288.45, 1261.45, 1157.29, 1068.56, 844.82, 759.95, 729.09 $cm^{-1}$; HPLC analysis: Chiralcel OB-H (Hex/IPA = 90/10, 1.0 mL/min, 254 nm, 22°C), 12.5 (major), 14.9 min, 74% *ee*.

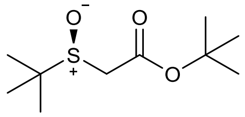

(*S*)-*tert*-butyl 2-(tert-butylsulfinyl)acetate(**3s**): White solid; 83% yield; mp: 95.7-96.8 °C; $^1H$ NMR (400 MHz, $CDCl_3$): $\delta$ = 3.37 (dd, $J$ = 60.6, 13.6 Hz, 2H), 1.49 (s, 9H), 1.27 (s, 9H); $^{13}C$ NMR (100 MHz, $CDCl_3$): $\delta$ = 165.35, 83.20, 54.08, 52.83, 27.96, 22.71; $[\alpha]_D^{22}$= –33.03 (*c* 3.6, MeOH); HRMS (ESI) calcd for $C_{10}H_{20}O_3S$ *m/z* $[M+H]^+$: 221.1211; found: 221.1209; IR: 1728.22, 1716.65, 1462.04, 1392.61, 1365.60, 1288.45, 1261.45, 1165.00, 1141.86, 1041.56, 956.69, 894.97, 840.96, 736.81 $cm^{-1}$; HPLC analysis: Chiralcel OD-H (Hex/IPA = 95/5, 1.0 mL/min, 230 nm, 22°C), 10.0 (major), 11.5 min, 37% *ee*.

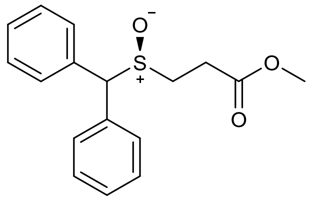

(*S*)-methyl 3-(benzhydrylsulfinyl)propanoate(**3t**): White solid; 88% yield; mp: 98.7-99.6 °C; $^1H$ NMR (400 MHz, $CDCl_3$): $\delta$ = 7.52 – 7.46 (m, 2H), 7.46 – 7.28 (m, 8H), 4.88 (s, 1H), 3.67 (s, 3H), 2.94 – 2.61 (m, 4H); $^{13}C$ NMR (100 MHz, $CDCl_3$): $\delta$ = 171.68, 135.35, 134.74, 129.27, 129.20, 128.74, 128.52, 128.42, 128.32, 72.78, 52.03, 45.43, 26.89; $[\alpha]_D^{22}$= +11.16 (*c* 5.25, MeOH); HRMS (ESI) calcd for $C_{17}H_{18}O_3S$ *m/z* $[M+H]^+$: 303.1055; found: 303.1046; IR: 1732.08, 1492.90, 1361.74, 1238.30, 1176.58, 1045.42, 736.81, 702.09 $cm^{-1}$; HPLC analysis: Chiralcel AD-H (Hex/IPA = 80/20, 1.0 mL/min, 230 nm, 22°C), 14.5 (major), 24.6 min, 94% *ee*.

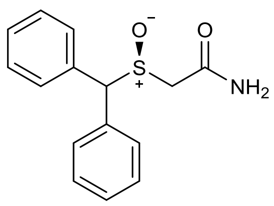

(*S*)-2-(benzhydrylsulfinyl)acetamide(**3u**): White solid; 96% yield; mp: 160.4-161.2 °C; $^1$H NMR (400 MHz, DMSO): $\delta$ = 7.67 (s, 1H), 7.56 – 7.47 (m, 4H), 7.46 – 7.38 (m, 4H), 7.38 – 7.33 (m, 2H), 7.30 (s, 1H), 5.33 (s, 1H), 3.29 (dd, *J* = 57.5, 13.6 Hz, 2H); $^{13}$C NMR (100 MHz, DMSO): $\delta$ = 166.87, 137.68, 135.42, 130.21, 129.54, 128.99, 128.46, 128.44, 69.30, 56.64; $[\alpha]_D^{22}$= +14.44 (*c* 5.24, MeOH); HRMS (ESI) calcd for $C_{15}H_{15}NO_2S$ *m/z* [M+H]$^+$: 274.0902; found: 274.0905; IR: 3170.97, 1693.50, 1612.49, 1454.33, 1400.32, 1033.85, 740.67, 482.20. 455.20 cm$^{-1}$; HPLC analysis: Chiralcel AS-H (Hex/IPA = 50/50, 1.0 mL/min, 230 nm, 22°C), 12.8 (major), 29.8 min, 82% *ee*.

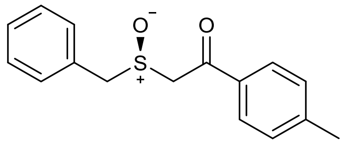

(*R*)-2-(benzylsulfinyl)-1-(p-tolyl)ethanone(**3v**): Yellow solid; 99% yield; mp: 104.8-106.1 °C; $^1$H NMR (400 MHz, $CDCl_3$): $\delta$ = 7.80 (d, *J* = 8.3 Hz, 2H), 7.42 – 7.30 (m, 5H), 7.30 – 7.22 (m, 2H), 4.17 (ddd, *J* = 19.0, 16.5, 8.3 Hz, 4H), 2.42 (s, 3H); $^{13}$C NMR (100 MHz, $CDCl_3$): $\delta$ = 192.05, 145.48, 133.56, 130.45, 129.57, 129.20, 128.86, 128.78, 128.51, 57.71, 57.61, 21.72; $[\alpha]_D^{22}$= +37.79 (*c* 5.48, MeOH); HRMS (ESI) calcd for $C_{16}H_{16}O_2S$ *m/z* [M+H]$^+$: 272.0949; found: 272.0944; IR: 1670.35, 1604.77, 1492.90, 1454.33, 1411.89, 1315.45, 1280.73, 1184.29, 1072.42, 1029.99, 975.98, 840.96, 763.81, 702.09 cm$^{-1}$; HPLC analysis: Chiralcel AD-H (Hex/IPA = 90/10, 1.0 mL/min, 230 nm, 22°C), 20.0 (major), 30.1 min, 90% *ee*.

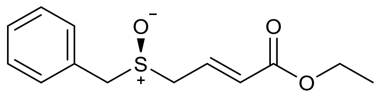

(*S*,*E*)-ethyl 4-(benzylsulfinyl)but-2-enoate(**3w**): Pale yellow oil; 84% yield; $^1$H NMR (400 MHz, $CDCl_3$): $\delta$ = 7.42 – 7.31 (m, 3H), 7.28 (dd, *J* = 7.6, 1.7 Hz, 2H), 6.93 (dt, *J* = 15.6, 7.8 Hz, 1H), 6.06 (dt, *J* = 15.6, 1.1 Hz, 1H), 4.33 – 4.08 (m, 2H), 4.06 – 3.87 (m, 2H), 3.42 (dddd, *J* = 60.4, 13.2, 7.8, 1.2 Hz, 2H), 1.28 (t, *J* = 7.1 Hz, 3H); $^{13}$C NMR (100 MHz, $CDCl_3$): $\delta$ = 164.98, 134.61, 129.93, 129.28, 129.05, 128.68, 128.55, 60.71, 57.35, 52.26, 14.10; $[\alpha]_D^{22}$= –6.73 (*c* 4.19, MeOH); HRMS (ESI) calcd for $C_{13}H_{16}O_3S$ *m/z* [M+H]$^+$: 253.0898; found: 253.0896; IR: 3032.10, 1712.79, 1651.07, 1496.76, 1454.33, 1396.46, 1369.46, 1319.31, 1273.02, 1199.72, 1149.57, 1041.56, 979.84, 767.67, 702.09 cm$^{-1}$; HPLC analysis: Chiralcel OB-H (Hex/IPA = 50/50, 1.0 mL/min, 230 nm, 22°C), 8.5 (major), 18.9 min, 77% *ee*.

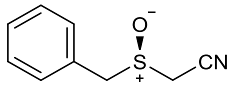

(*R*)-2-(benzylsulfinyl)acetonitrile(**3x**): White solid; 87% yield; mp: 110.4-111.8 °C; $^{1}H$ NMR (400 MHz, $CDCl_3$): $\delta$ = 7.47 – 7.39 (m, 3H), 7.36 (dt, $J$ = 4.9, 4.0 Hz, 2H), 4.36 – 4.18 (m, 2H), 3.42 (dd, $J$ = 76.6, 16.1 Hz, 2H); $^{13}C$ NMR (100 MHz, $CDCl_3$): $\delta$ = 130.04, 129.42, 129.26, 127.53, 111.27, 57.86, 36.87; $[\alpha]_D^{22}$= +58.46 (*c* 3.05, MeOH); HRMS (ESI) calcd for $C_9H_9NOS$ *m/z* $[M+H^-]^+$: 180.0483; found: 180.0484; IR: 2306.86, 1419.61, 1076.28, 894.97, 740.67, 702.09 $cm^{-1}$; HPLC analysis: Chiralcel OB-H (Hex/IPA = 90/10, 1.0 mL/min, 230 nm, 22°C), 14.0 (major), 18.2 min, 80% *ee*.

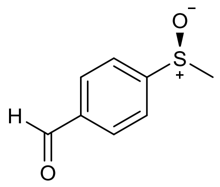

(*S*)-4-(methylsulfinyl)benzaldehyde(**3y**): White solid; 82% yield; mp: 70.2-72.0 °C; $^{1}H$ NMR (400 MHz, $CDCl_3$): $\delta$ = 10.06 (s, 1H), 8.02 (d, $J$ = 8.4 Hz, 2H), 7.79 (d, $J$ = 8.2 Hz, 2H), 2.75 (s, 3H); $^{13}C$ NMR (100 MHz, $CDCl_3$): $\delta$ = 191.04, 152.34, 138.06, 130.32, 124.09, 43.67;$[\alpha]_D^{22}$= –70.0 (*c* 2.76, MeOH); HRMS (ESI) calcd for $C_8H_8O_2S$ *m/z* $[M+H]^+$:169.0323; found: 169.0326; IR: 2850.79, 2738.92, 1701.22, 1593.20, 1573.91, 1415.75, 1384.89, 1296.16, 1273.02, 1199.72, 1168.86, 1149.57, 1087.85, 956.69, 825.53, 736.81, 694.37 $cm^{-1}$; HPLC analysis: Chiralcel AS-H (Hex/IPA = 70/30, 1.0 mL/min, 230 nm, 22°C), 31.9, 50.4 (major) min, 65% *ee*.

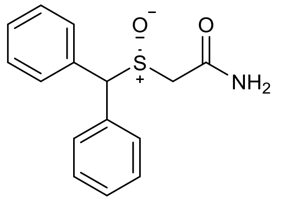

(*R*)-2-(benzhydrylsulfinyl)acetamide(**4**): $[\alpha]_D^{22}$ = –16.25 (*c* 2.0, MeOH); HPLC analysis: Chiralcel AS-H (Hex/IPA = 50/50, 1.0 mL/min, 230 nm, 22°C), 12.7, 28.8 (major) min, 91% *ee*.

**Computational Methods**: Geometry optimization was performed with a two-layer ONIOM(QM:QM')[11] method using the Gaussian09 software package.[12] The initial structure of the ion-pairing model for multiscale geometry optimization was taken from a crystal structure. The B3LYP density functional theory (DFT) method[13-15] with the B1 basis set and the semiempirical PM6[16] method were used for the QM and QM' calculations, respectively. Here, B1 is a combination of the SDD effective core potential basis set[17] for Mo and the 6-31G* basis set[18] for remaining atoms. Vibrational analyses were done for ONIOM(B3LYP/B1:PM6)-

optimized geometries to confirm their nature and to obtain zero-point energy (ZPE) corrections. To improve the accuracy of energies, single-point energy calculations were performed on the ONIOM(B3LYP/B1:PM6) geometries with the ONIOM(B3LYP/B2:PM6) method, where B2 is a combination of SDD effective core potential basis set for Mo and 6-311+G(df,p) for other atoms. Dispersion effects were included in ONIOM(B3LYP/B2:PM6) single point energy calculations using Grimme's D3 correction scheme with zero damping.[19] Solvent effects of diisopropyl ether on the reactions were taken into account in both geometry optimization and single point calculations, using a self-consistent reaction field (SCRF) method called IEFPCM,[20] as implemented in Gaussian 09.[12] UCSF Chimera was used to prepare molecular drawings, unless stated otherwise.[21] Non-covalent interaction (NCI) analysis[22] was performed for relevant species with NCIplot 3.0[23] to provide visualization of NCIs as isosurfaces. Visual Molecular Dynamics (VMD) software[24] was used for visualizing NCI analysis results.

**Multiscale ONIOM Geometry Optimization: Stable Ion-Pairing Arrangement**

ONIOM partitioning used in multiscale calculations was shown in Supplementary Fig. 10. To determine a suitable ion-pairing structure to be used in transition state (TS) structure search, ONIOM geometry optimization was performed on the initial crystal structure in both gaseous and solvent environments. The electronic energy of each structure was evaluated by performing a single-point (SP) energy calculation using the DFT (B3LYP/B1) method. Supplementary Table 3 summarizes the SCF energies obtained from SP calculations. The results show that multiscale ONIOM optimization with the solvent effect included gives a lower energy structure, and thus ONIOM structures were used for subsequent NCI analysis and TS structure search.

**Multiscale ONIOM Geometry Optimization: Conformational Sampling of TS Structures**

TS conformational sampling methodology was shown in Supplementary Fig. 11. To determine the most stable TS structures leading to chiral products, an arbitrary TS structure en route to a

specific enantiomer was initially located for both *R* and *S* products. While keeping the structure of bisguanidinium unchanged, dihedral angles D1, D2 and D3 were varied systematically to find other TS structures. In theory, at least 3 staggered conformations are possible for each dihedral angle considered. However, due to the presence of bulky substituents around the reaction site as well as steric requirement of the reaction, the number of possibilities diminished significantly for each enantiomer. The result of TS conformational sampling is summarized in Supplementary Tables 4-5. The results show that TSR-05 and TSS-09 are the most stable TSs leading to *R* and *S* products, respectively, and were used for NCI analysis.

**Non-Covalent Interactions (NCI) Analysis**

NCI analysis was performed to visually identify non-covalent interactions between the sulfide substrate and the ion-pairing structure during the formation of transition states leading to both enantiomeric products. NCI isosurfaces were generated with the promolecular density of NCIplot 3.0,[21-22] and VMD software was used for visualizing results.[22] Intermolecular non-covalent interactions are shown as colored isosurfaces. Qualitatively speaking, red to yellow colors indicate repulsive interactions, green indicates a weakly attractive interaction, and blue indicates a strongly attractive interaction. Both TS structures have comparable angles and bond lengths, which indicates that the substrate orientation plays a significant role in determining the relative stability of transition states. The energy barrier differed by 1.90 kcal/mol, with the TS of *R* enantiomer having a lower energy, which is in agreement with the experimentally observed product ratio. For the most part, the NCI isosurfaces between the substrate and the ion-pairing in both TS structures correspond to weak vdW interactions (green isosurface). An isosurface arising from CH---O interactions between the ester group in the sulfide substrate and a side chain in bisguanidinium was found for TSR-05 (Supplementary Fig. 12). Due to different substrate orientations, such interactions were absent in TSS-09 (Supplementary Fig. 13), which would explain the relatively greater stability of TSR-05.

## Supplementary References


1. Wang, C., Zong, L. & Tan, C.-H. Enantioselective oxidation of alkenes with potassium permanganate catalyzed by chiral dicationic bisguanidinium. *J. Am. Chem. Soc.* **137**, 10677-10682 (2015).
2. Altieri, A. *et al.* Sulfur-containing amide-based [2]rotaxanes and molecular shuttles. *Chem. Sci.* **2**, 1922-1928 (2011).
3. Zeng, Q. *et al.* Benzoheterocyclecarboxaldehyde derivatives as IRE-1α inhibitors and their preparation and use for the treatment of diseases. WO2011127070A2 (2011).
4. Miura, K., Fujisawa, N., Saito, H., Wang, D. & Hosomi, A. Synthetic Utility of Stannyl Enolates as Radical Alkylating Agents1. *Org. Lett.* **3**, 2591-2594 (2001).
5. Loghmani-Khouzani, H., Poorheravi, M. R., Sadeghi, M. M. M., Caggiano, L. & Jackson, R. F. W. α-Fluorination of β-ketosulfones by Selectfluor F-TEDA-BF4. *Tetrahedron* **64**, 7419-7425 (2008).
6. Liang, S. Improved process for preparing benzhydrylthioacetamide. WO2004075841A2 (2004).
7. Bhatt, S. B. *et al.* Improved process for the preparation of 2-[(diphenylmethyl)thio]acetamide, intermediate for the preparation of Modafinil, from 2-[(diphenylmethyl)thio]acetic acid, alcohols and ammonia. WO2004075827A2 (2004).
8. Schwenkkraus, P. & Otto, H. H. Properties and reactions of substituted 1,2-thiazetidine 1,1-dioxides: C-3 substituted β-sultams. *Arch. Pharm. (Weinheim, Ger.)* **326**, 519-523 (1993).
9. Marson, C. M. *et al.* Aromatic sulfide inhibitors of histone deacetylase based on arylsulfinyl-2,4-hexadienoic acid hydroxyamides. *J. Med. Chem.* **49**, 800-805 (2006).
10. Tsui, G. C., Glenadel, Q., Lau, C. & Lautens, M. Rhodium(I)-Catalyzed Addition of Arylboronic Acids to (Benzyl-/Arylsulfonyl)acetonitriles: Efficient Synthesis of (Z)-β-Sulfonylvinylamines and β-Keto Sulfones. *Org. Lett.* **13**, 208-211 (2011).
11. Humphrey, W.; Dalke, A.; Schulten, K. *J. Mol. Graph.* **1996**, *14*, 33-38.
12. Frisch, M. J.; Trucks, G. W.; Schlegel, H. B.; Scuseria, G. E.; Robb, M. A.; Cheeseman, J. R.; Scalmani, G.; Barone, V.; Mennucci, B.; Petersson, G. A.; Nakatsuji, H.; Caricato, M.; Li, X.; Hratchian, H. P.; Izmaylov, A. F.; Bloino, J.; Zheng, G.; Sonnenberg, J. L.; Hada, M.; Ehara, M.; Toyota, K.; Fukuda, R.; Hasegawa, J.; Ishida, M.; Nakajima, T.; Honda, Y.; Kitao, O.; Nakai, H.; Vreven, T.; Montgomery, J. A., Jr.; Peralta, J. E.; Ogliaro, F.; Bearpark, M.; Heyd, J. J.; Brothers, E.; Kudin, K. N.; Staroverov, V. N.; Kobayashi, R.; Normand, J.; Raghavachari, K.; Rendell, A.; Burant, J. C.; Iyengar, S. S.; Tomasi, J.; Cossi, M.; Rega, N.; Millam, J. M.; Klene, M.; Knox, J. E.; Cross, J. B.; Bakken, V.; Adamo, C.; Jaramillo, J.; Gomperts, R.; Stratmann, R. E.; Yazyev, O.; Austin, A. J.; Cammi, R.; Pomelli, C.; Ochterski, J. W.; Martin, R. L.; Morokuma, K.; Zakrzewski, V. G.; Voth, G. A.; Salvador, P.; Dannenberg, J. J.; Dapprich, S.; Daniels, A. D.; Farkas, Ö.; Foresman, J. B.; Ortiz, J. V.; Cioslowski, J.; Fox, D. J. Gaussian 09, Gaussian, Inc.: Wallingford, CT, USA, 2009.
13. Becke, A. D. *J. Chem. Phys.* **1993**, *98*, 5648-5652.
14. Lee, C.; Yang, W.; Parr, R. G. *Phys. Rev. B, PRB* **1988**, *37*, 785-789.

15. Vosko, S. H.; Wilk, L.; Nusair, M. *Can. J. Phys.* **1980**, *58*, 1200-1211.
16. Stewart, J. J. P. *J. Mol. Model.* **2007**, *13*, 1173-1213.
17. Dolg, M.; Wedig, U.; Stoll, H.; Preuss, H. *J. Chem. Phys.* **1987**, *86*, 866-872.
18. Wiberg, K. B. *J. Comput. Chem.* **1986**, *7*, 379-379.
19. Grimme, S.; Antony, J.; Ehrlich, S.; Krieg, H. *J. Chem. Phys.* **2010**, *132*, 154104.
20. Tomasi, J.; Mennucci, B.; Cammi, R. *Chem. Rev.* **2005**, *105*, 2999-3094.
21. Pettersen, E. F.; Goddard, T. D.; Huang, C. C.; Couch, G. S.; Greenblatt, D. M.; Meng, E. C.; Ferrin, T. E. *J. Comput. Chem.* **2004**, *25*, 1605-1612.
22. Johnson, E. R.; Keinan, S.; Mori-Sánchez, P.; Contreras-García, J.; Cohen, A. J.; Yang, W. *J. Am. Chem. Soc.* **2010**, *132*, 6498-6506.
23. Contreras-García, J.; Johnson, E. R.; Keinan, S.; Chaudret, R.; Piquemal, J.-P.; Beratan, D. N.; Yang, W. *J. Chem. Theory Comput.* **2011**, *7*, 625-632.
24. Humphrey, W.; Dalke, A.; Schulten, K. *J. Mol. Graph.* **1996**, *14*, 33-38.
