# Supplementary material for: Bisguanidinium dinuclear oxodiperoxomolybdosulfate ion pair-catalyzed enantioselective sulfoxidation
Source: Nat Commun. 2016 Nov 21;7:13455. doi: 10.1038/ncomms13455 (PMC5121337; doi:10.1038/ncomms13455)
Supplement: Supplementary Data 1 — Cartesian Coordinates for TS Structures. [file ncomms13455-s2.docx]

**Cartesian Coordinates for TS Structures**

**TSR-01**

Mo 4.724854 16.040823 3.020061

Mo 5.583857 17.365244 6.381371

O 6.083499 13.673017 6.264334

O 8.074359 14.023145 4.764078

O 6.950225 15.949209 5.764458

O 5.785335 14.607504 4.019121

O 5.390753 15.754094 1.487595

O 3.145921 14.871517 3.168781

O 2.793845 16.208936 2.703522

O 4.759886 17.980541 3.156078

O 5.899443 17.446993 3.871800

O 4.150954 16.227283 5.441561

O 3.701348 17.557134 5.805124

O 5.309311 16.862064 7.985323

O 5.597318 19.228909 6.365547

O 7.194313 18.496744 6.503992

S 6.768045 14.480210 5.242061

C 3.146955 13.609845 6.540094

C 2.911067 13.338947 8.004421

C 2.061766 12.322707 8.462422

C 1.897245 12.108610 9.838093

C 1.004231 10.989528 10.386864

C 2.603647 12.916888 10.739451

C 3.472185 13.925748 10.296043

C 3.609742 14.139941 8.918159

C 2.497654 11.554730 5.098782

C 3.758498 11.501443 4.273875

C 4.786411 10.622662 4.647816

C 5.959749 10.565587 3.890769

C 6.124677 11.402242 2.780991

C 5.101290 12.279925 2.408070

C 3.908924 12.308119 3.137234

C 1.227307 11.220742 4.241310

C 0.535948 9.966261 4.721459

C 0.787762 8.765329 4.040118

C 0.186023 7.579952 4.469798

C -0.671799 7.587197 5.574799

C -0.925335 8.783856 6.251857

C -0.321846 9.972102 5.829581

C -0.817679 12.622331 3.541414

C -0.453847 12.571614 2.074457

C -0.999005 11.544260 1.288420

C -0.700621 11.470393 -0.074985

C -1.266187 10.369486 -0.979783

C -2.041417 9.298866 -0.189542

C -2.222615 11.026817 -1.994816

C 0.137923 12.449811 -0.637647

C 0.681458 13.484011 0.131279

C 1.597957 14.552359 -0.480760

C 0.390943 13.526886 1.508270

C 0.994117 13.405776 5.188269

C 0.308907 15.106659 6.911200

C 1.207236 16.339590 7.136206

C 1.125466 16.814823 4.736288

C 0.196202 15.634054 4.463143

C 1.185442 18.707748 6.311144

C 2.934504 18.748518 8.177126

C 2.878891 19.020999 9.659978

C 4.099362 19.218812 10.319466

C 4.124069 19.449754 11.701787

C 5.450643 19.662498 12.440994

C 5.914929 18.292783 12.970316

C 5.264335 20.638596 13.622305

C 6.522856 20.250339 11.507334

C 2.912053 19.442598 12.407108

C 1.684484 19.228449 11.760250

C 0.397223 19.238966 12.592300

C 0.550042 18.258416 13.773248

C 0.176873 20.671870 13.117547

C -0.837031 18.816605 11.772319

C 1.675223 19.024829 10.374554

C 1.713919 20.801087 7.318286

C 0.736079 21.398224 8.303579

C 1.173075 22.429143 9.148476

C 0.279994 23.024483 10.043803

C -1.048720 22.592662 10.103281

C -1.483328 21.558794 9.267440

C -0.595037 20.960674 8.369673

C 1.278220 21.042694 5.835135

C 2.444802 21.453884 4.964940

C 2.450677 22.738461 4.398539

C 3.527174 23.144841 3.606415

C 4.598311 22.274612 3.368727

C 4.590165 20.993676 3.925898

C 3.517017 20.584327 4.724340

C -0.523686 19.615313 4.658545

C -0.367931 19.387673 3.169581

C -1.493579 18.894778 2.485601

C -1.440067 18.682549 1.105129

C -2.639428 18.091764 0.354136

C -2.470603 16.559715 0.373970

C -2.682644 18.592826 -1.102219

C -3.970407 18.478505 1.027531

C -0.240892 18.950010 0.420466

C 0.890671 19.422217 1.089977

C 0.809538 19.656941 2.476731

N 2.209639 12.949385 5.615056

N 0.363472 12.438181 4.415964

N 0.258846 14.575601 5.520006

N 0.860023 17.370450 6.107790

N 1.753589 19.291980 7.446300

N 0.758362 19.691864 5.408877

C 4.201744 14.809849 11.316159

C 5.478425 15.421533 10.725607

C 4.597946 13.988724 12.561915

C 3.216835 15.924403 11.722295

C 1.927594 9.866620 10.899369

C 0.059207 10.413409 9.315422

C 0.139221 11.530035 11.544049

C 1.535585 14.554628 -2.021063

C 3.036174 14.250915 -0.028798

C 1.163227 15.938994 0.023835

C 2.220830 19.690179 0.373562

C 2.192068 19.242738 -1.099968

C 3.335548 18.910222 1.091660

C 2.500966 21.204874 0.432657

C -0.102484 9.678162 -1.717812

H 2.212830 16.488517 4.667846

H 1.028864 17.578433 3.932708

H 0.477388 15.212368 3.454815

H -0.868880 15.961562 4.417578

H 2.299916 16.060935 7.060616

H 1.030613 16.733324 8.163668

H 0.597950 14.306543 7.633160

H -0.753508 15.374983 7.145905

H 1.495877 11.117384 3.151092

H 2.575454 10.867731 5.986030

H 1.454741 8.750299 3.176620

H -0.525866 10.900650 6.362931

H -1.592831 8.791500 7.113857

H -1.140171 6.661018 5.907828

H 0.386968 6.647362 3.941432

H 6.759053 9.884631 4.181239

H 4.683834 9.994412 5.531866

H 3.093435 12.971462 2.817024

H 5.239627 12.965235 1.565513

H 7.057145 11.382244 2.216814

H -1.340052 13.576678 3.785489

H -1.540919 11.806959 3.803531

H -1.652800 10.808425 1.753775

H 0.843525 14.311491 2.133645

H 0.369615 12.388465 -1.702790

H -1.406573 8.805359 0.554085

H -2.408093 18.676107 3.034326

H 1.687639 20.034762 3.005799

H -0.200635 18.770526 -0.655328

H -1.087957 20.571410 4.808915

H -1.161326 18.806719 5.096718

H 2.747040 21.214199 7.490867

H 0.456569 21.803199 5.779329

H -0.940008 20.150879 7.729718

H -2.517706 21.219086 9.315793

H -1.743802 23.059369 10.800868

H 0.623460 23.826064 10.698440

H 2.210097 22.766989 9.115986

H 1.622021 23.422643 4.572954

H 3.532580 24.143346 3.171475

H 5.435893 22.595846 2.750757

H 3.546094 19.567691 5.140455

H 5.412836 20.285874 3.754146

H 3.879800 19.194860 7.735591

H 3.042359 17.642812 8.013694

H 5.034544 19.163183 9.750420

H 2.922525 19.601274 13.486587

H 0.738679 18.881286 9.839719

H 4.292210 14.926861 8.554501

H 2.481614 12.752160 11.811464

H 1.544293 11.683733 7.752601

H 3.168477 14.720605 6.321505

H 4.210153 13.276747 6.273323

H 3.428033 21.454150 -0.094352

H 1.692322 21.780957 -0.026337

H 2.617454 21.555754 1.465537

H 3.478813 19.253729 2.129244

H 3.115973 17.830481 1.132644

H 4.303509 19.019912 0.590185

H 2.002570 18.158209 -1.180489

H 1.428937 19.770415 -1.678566

H 3.156467 19.432790 -1.584336

H -1.803670 18.270691 -1.672637

H -3.563617 18.209284 -1.627871

H -2.723646 19.686443 -1.147567

H -4.092927 19.566024 1.071637

H -4.824140 18.075065 0.472059

H -4.042274 18.093652 2.050140

H -1.476661 16.259972 -0.005194

H -2.562404 16.157371 1.387128

H -3.222146 16.064808 -0.248260

H 1.648720 16.748152 -0.545998

H 0.075807 16.086980 -0.050781

H 1.459799 16.093694 1.076768

H 3.737092 15.035399 -0.346438

H 3.113017 14.224989 1.073597

H 3.399823 13.297279 -0.416584

H 2.198412 15.324830 -2.432412

H 1.861402 13.599383 -2.445726

H 0.525222 14.763728 -2.384828

H 0.625740 9.262866 -1.012909

H -0.461628 8.854236 -2.343427

H 0.436707 10.369018 -2.374888

H -2.907580 9.721044 0.330504

H -2.420445 8.516865 -0.857505

H -1.708524 11.760351 -2.624783

H -2.666526 10.280952 -2.662482

H -3.043335 11.547449 -1.490430

H 2.594079 10.221126 11.693301

H 1.352542 9.028207 11.304147

H 2.563016 9.475543 10.096885

H 0.751631 11.871750 12.386227

H -0.476000 12.375451 11.221121

H -0.533956 10.757760 11.930357

H 0.614587 9.946482 8.493825

H -0.587659 9.637748 9.740745

H -0.591391 11.186104 8.895250

H 2.284023 15.519203 12.121383

H 3.650246 16.580561 12.485755

H 2.969626 16.569835 10.870004

H 5.179187 14.598771 13.261185

H 3.725473 13.612386 13.104307

H 5.217697 13.128550 12.286604

H 6.235919 14.662582 10.510241

H 5.293071 15.954824 9.769169

H 5.923282 16.166151 11.402983

H 6.787659 19.562765 10.687089

H 6.190617 21.188041 11.053660

H 7.454635 20.449557 12.045996

H 6.216414 20.826358 14.129520

H 4.875523 21.603651 13.282553

H 4.571144 20.245775 14.373528

H 6.893178 18.363634 13.455376

H 5.210026 17.879439 13.697471

H 6.011671 17.556968 12.150417

H 1.008003 21.006442 13.746693

H 0.086043 21.385581 12.289967

H -0.735541 20.740778 13.717474

H -0.728699 17.802387 11.375470

H -1.741132 18.829005 12.390315

H -1.012737 19.495552 10.929571

H 1.344906 18.566835 14.460659

H -0.373302 18.190255 14.356815

H 0.796765 17.250711 13.421728

S 9.217316 17.622834 7.006312

C 10.070616 16.946913 5.473956

C 9.793531 19.344664 7.108972

H 9.295264 16.260354 5.126734

C 11.288613 16.109230 5.831891

C 10.285308 17.992557 4.387779

H 9.631206 19.859744 6.159991

H 10.866077 19.396441 7.343749

C 9.040856 20.060288 8.218366

C 11.395855 14.825966 5.275909

C 12.312671 16.558128 6.679370

C 9.187732 18.387849 3.603974

C 11.536087 18.572418 4.132337

O 8.615930 19.545555 9.231000

O 8.948289 21.373355 7.943213

C 12.508470 14.023678 5.541074

H 10.590376 14.449116 4.653229

C 13.425933 15.758883 6.943296

H 12.239154 17.533782 7.151588

C 9.341660 19.343509 2.598644

H 8.211271 17.951715 3.778384

C 11.688283 19.528709 3.124425

H 12.404406 18.264814 4.708246

C 8.289486 22.170003 8.944011

C 13.530430 14.488281 6.370780

H 12.569561 13.029545 5.104297

H 14.209069 16.127078 7.602953

C 10.591588 19.919452 2.354496

H 8.480452 19.631944 2.002546

H 12.669730 19.962028 2.939306

H 8.362646 23.199343 8.589230

H 7.244287 21.869740 9.039590

H 8.789488 22.063271 9.911707

H 14.396162 13.863118 6.579852

H 10.711051 20.660846 1.566916

**TSR-02**

Mo 4.720287 16.041375 3.022791

Mo 5.593163 17.369233 6.376883

O 6.081125 13.691372 6.271835

O 8.070740 14.011971 4.764599

O 6.964948 15.957383 5.749932

O 5.787331 14.609234 4.018290

O 5.386223 15.757669 1.490120

O 3.145166 14.867908 3.172674

O 2.789112 16.203843 2.706407

O 4.748125 17.981214 3.159814

O 5.891606 17.452626 3.872693

O 4.158136 16.223581 5.446158

O 3.705967 17.551275 5.814108

O 5.338093 16.853744 7.981205

O 5.586304 19.227976 6.390586

O 7.212216 18.505026 6.483839

S 6.769366 14.484612 5.241170

C 3.146955 13.609845 6.540093

C 2.911068 13.338947 8.004421

C 2.061766 12.322707 8.462422

C 1.897245 12.108610 9.838093

C 1.004231 10.989528 10.386864

C 2.603647 12.916888 10.739451

C 3.472185 13.925748 10.296043

C 3.609743 14.139941 8.918161

C 2.497654 11.554730 5.098782

C 3.758498 11.501443 4.273875

C 4.786411 10.622662 4.647816

C 5.959749 10.565587 3.890769

C 6.124677 11.402242 2.780991

C 5.101290 12.279925 2.408070

C 3.908924 12.308119 3.137235

C 1.227307 11.220742 4.241310

C 0.535948 9.966261 4.721459

C 0.787762 8.765329 4.040118

C 0.186023 7.579952 4.469798

C -0.671799 7.587197 5.574799

C -0.925335 8.783856 6.251857

C -0.321846 9.972102 5.829581

C -0.817679 12.622331 3.541414

C -0.453847 12.571614 2.074457

C -0.999005 11.544260 1.288420

C -0.700621 11.470393 -0.074985

C -1.266187 10.369486 -0.979783

C -2.041417 9.298866 -0.189542

C -2.222615 11.026817 -1.994816

C 0.137923 12.449811 -0.637647

C 0.681458 13.484011 0.131279

C 1.597957 14.552359 -0.480760

C 0.390943 13.526886 1.508270

C 0.994117 13.405776 5.188269

C 0.308907 15.106659 6.911200

C 1.207236 16.339589 7.136205

C 1.125466 16.814823 4.736287

C 0.196202 15.634054 4.463143

C 1.185442 18.707748 6.311144

C 2.934504 18.748519 8.177126

C 2.878890 19.020999 9.659978

C 4.099362 19.218812 10.319466

C 4.124069 19.449754 11.701787

C 5.450643 19.662498 12.440994

C 5.914929 18.292783 12.970316

C 5.264335 20.638596 13.622305

C 6.522856 20.250339 11.507334

C 2.912053 19.442598 12.407108

C 1.684484 19.228449 11.760250

C 0.397223 19.238966 12.592300

C 0.550042 18.258416 13.773248

C 0.176873 20.671870 13.117547

C -0.837031 18.816605 11.772319

C 1.675223 19.024829 10.374554

C 1.713919 20.801087 7.318286

C 0.736079 21.398224 8.303579

C 1.173075 22.429143 9.148476

C 0.279994 23.024483 10.043803

C -1.048720 22.592662 10.103281

C -1.483328 21.558794 9.267440

C -0.595037 20.960674 8.369673

C 1.278220 21.042694 5.835135

C 2.444801 21.453883 4.964937

C 2.450677 22.738461 4.398539

C 3.527174 23.144841 3.606415

C 4.598311 22.274612 3.368727

C 4.590164 20.993676 3.925897

C 3.517008 20.584326 4.724339

C -0.523686 19.615313 4.658545

C -0.367931 19.387673 3.169581

C -1.493579 18.894778 2.485601

C -1.440067 18.682549 1.105129

C -2.639428 18.091764 0.354136

C -2.470603 16.559715 0.373970

C -2.682644 18.592826 -1.102219

C -3.970407 18.478505 1.027531

C -0.240892 18.950010 0.420466

C 0.890671 19.422217 1.089977

C 0.809538 19.656941 2.476731

N 2.209639 12.949385 5.615056

N 0.363472 12.438181 4.415964

N 0.258846 14.575601 5.520006

N 0.860023 17.370450 6.107790

N 1.753589 19.291980 7.446299

N 0.758362 19.691864 5.408877

C 4.201744 14.809850 11.316159

C 5.478432 15.421565 10.725623

C 4.597946 13.988724 12.561915

C 3.216835 15.924403 11.722295

C 1.927594 9.866620 10.899369

C 0.059207 10.413409 9.315422

C 0.139221 11.530035 11.544049

C 1.535585 14.554628 -2.021063

C 3.036174 14.250915 -0.028798

C 1.163227 15.938994 0.023835

C 2.220830 19.690179 0.373562

C 2.192068 19.242738 -1.099968

C 3.335549 18.910222 1.091660

C 2.500966 21.204874 0.432657

C -0.102484 9.678162 -1.717812

H 2.212830 16.488520 4.667845

H 1.028864 17.578432 3.932707

H 0.477388 15.212368 3.454815

H -0.868880 15.961562 4.417578

H 2.299915 16.060938 7.060617

H 1.030613 16.733324 8.163668

H 0.597950 14.306543 7.633160

H -0.753508 15.374983 7.145905

H 1.495877 11.117384 3.151092

H 2.575454 10.867731 5.986030

H 1.454741 8.750299 3.176620

H -0.525866 10.900650 6.362931

H -1.592831 8.791500 7.113857

H -1.140171 6.661018 5.907828

H 0.386968 6.647362 3.941432

H 6.759053 9.884631 4.181239

H 4.683834 9.994412 5.531866

H 3.093436 12.971463 2.817024

H 5.239627 12.965235 1.565513

H 7.057145 11.382244 2.216814

H -1.340052 13.576678 3.785489

H -1.540919 11.806959 3.803531

H -1.652800 10.808425 1.753775

H 0.843525 14.311491 2.133645

H 0.369615 12.388465 -1.702790

H -1.406573 8.805359 0.554085

H -2.408093 18.676107 3.034326

H 1.687639 20.034762 3.005799

H -0.200635 18.770526 -0.655328

H -1.087957 20.571410 4.808915

H -1.161326 18.806719 5.096718

H 2.747040 21.214199 7.490867

H 0.456569 21.803199 5.779329

H -0.940008 20.150879 7.729718

H -2.517706 21.219086 9.315793

H -1.743802 23.059369 10.800868

H 0.623460 23.826064 10.698440

H 2.210097 22.766989 9.115986

H 1.622021 23.422643 4.572954

H 3.532580 24.143346 3.171475

H 5.435893 22.595846 2.750757

H 3.546109 19.567696 5.140464

H 5.412836 20.285875 3.754147

H 3.879801 19.194859 7.735595

H 3.042359 17.642803 8.013692

H 5.034544 19.163183 9.750420

H 2.922525 19.601274 13.486587

H 0.738679 18.881286 9.839719

H 4.292200 14.926861 8.554499

H 2.481614 12.752160 11.811464

H 1.544293 11.683733 7.752601

H 3.168477 14.720608 6.321506

H 4.210154 13.276747 6.273324

H 3.428033 21.454150 -0.094352

H 1.692322 21.780957 -0.026337

H 2.617454 21.555754 1.465537

H 3.478812 19.253728 2.129244

H 3.115973 17.830481 1.132644

H 4.303509 19.019912 0.590185

H 2.002570 18.158209 -1.180489

H 1.428937 19.770415 -1.678566

H 3.156467 19.432790 -1.584336

H -1.803670 18.270691 -1.672637

H -3.563617 18.209284 -1.627871

H -2.723646 19.686443 -1.147567

H -4.092927 19.566024 1.071637

H -4.824140 18.075065 0.472059

H -4.042274 18.093652 2.050140

H -1.476661 16.259972 -0.005194

H -2.562404 16.157371 1.387128

H -3.222146 16.064808 -0.248260

H 1.648720 16.748152 -0.545998

H 0.075807 16.086980 -0.050781

H 1.459799 16.093694 1.076768

H 3.737092 15.035399 -0.346438

H 3.113017 14.224989 1.073597

H 3.399823 13.297279 -0.416584

H 2.198412 15.324830 -2.432412

H 1.861402 13.599383 -2.445726

H 0.525222 14.763728 -2.384828

H 0.625740 9.262866 -1.012909

H -0.461628 8.854236 -2.343427

H 0.436707 10.369018 -2.374888

H -2.907580 9.721044 0.330504

H -2.420445 8.516865 -0.857505

H -1.708524 11.760351 -2.624783

H -2.666526 10.280952 -2.662482

H -3.043335 11.547449 -1.490430

H 2.594079 10.221126 11.693301

H 1.352542 9.028207 11.304147

H 2.563016 9.475543 10.096885

H 0.751631 11.871750 12.386227

H -0.476000 12.375451 11.221121

H -0.533956 10.757760 11.930357

H 0.614587 9.946482 8.493825

H -0.587659 9.637748 9.740745

H -0.591391 11.186104 8.895250

H 2.284023 15.519203 12.121383

H 3.650246 16.580561 12.485755

H 2.969626 16.569835 10.870004

H 5.179187 14.598771 13.261185

H 3.725473 13.612386 13.104307

H 5.217697 13.128550 12.286604

H 6.235919 14.662582 10.510241

H 5.293067 15.954789 9.769145

H 5.923284 16.166150 11.402982

H 6.787659 19.562765 10.687089

H 6.190617 21.188041 11.053660

H 7.454635 20.449557 12.045996

H 6.216414 20.826358 14.129520

H 4.875523 21.603651 13.282553

H 4.571144 20.245775 14.373528

H 6.893178 18.363634 13.455376

H 5.210026 17.879439 13.697471

H 6.011671 17.556968 12.150417

H 1.008003 21.006442 13.746693

H 0.086043 21.385581 12.289967

H -0.735541 20.740778 13.717474

H -0.728699 17.802387 11.375470

H -1.741132 18.829005 12.390315

H -1.012737 19.495552 10.929571

H 1.344906 18.566835 14.460659

H -0.373302 18.190255 14.356815

H 0.796765 17.250711 13.421728

S 9.257213 17.710115 6.894185

C 10.096684 16.920547 5.392730

C 9.861873 19.437875 6.952734

H 9.306511 16.219435 5.114739

C 11.313802 16.103787 5.794153

C 10.283351 17.889411 4.236932

H 8.965760 20.056735 7.037624

H 10.388832 19.676636 6.025795

C 10.789725 19.670622 8.125654

C 11.354086 14.753825 5.413946

C 12.397520 16.633854 6.512447

C 9.164407 18.221869 3.452774

C 11.523902 18.457126 3.911000

O 12.004036 19.682215 8.067643

O 10.096434 19.855376 9.264238

C 12.460489 13.958671 5.723734

H 10.504998 14.323728 4.891750

C 13.503080 15.838323 6.818603

H 12.380410 17.666006 6.850416

C 9.286743 19.102826 2.378586

H 8.195509 17.793369 3.681332

C 11.643882 19.340720 2.833708

H 12.408272 18.196950 4.484826

C 10.884780 20.026828 10.455080

C 13.541568 14.498459 6.422539

H 12.471191 12.913423 5.423202

H 14.334303 16.267388 7.374724

C 10.526693 19.667721 2.064574

H 8.409577 19.342229 1.784068

H 12.617017 19.766512 2.595826

H 10.166218 20.147840 11.265689

H 11.513961 19.149337 10.626996

H 11.519897 20.913649 10.370367

H 14.403078 13.879602 6.665884

H 10.621770 20.351613 1.223510

**TSR-03**

Mo 4.724266 16.044065 3.025632

Mo 5.592995 17.373702 6.372859

O 6.080519 13.682126 6.267677

O 8.079300 14.009199 4.774911

O 6.961563 15.950021 5.752974

O 5.797877 14.606172 4.014632

O 5.386870 15.763769 1.491163

O 3.152530 14.865054 3.175814

O 2.791495 16.199421 2.709055

O 4.740369 17.981232 3.171351

O 5.890204 17.457677 3.877820

O 4.158303 16.222067 5.451166

O 3.702602 17.548721 5.820109

O 5.353737 16.855460 7.980046

O 5.580414 19.228419 6.401901

O 7.221221 18.510954 6.452208

S 6.771395 14.479942 5.241943

C 3.146955 13.609845 6.540094

C 2.911067 13.338947 8.004421

C 2.061766 12.322707 8.462422

C 1.897245 12.108610 9.838093

C 1.004231 10.989528 10.386864

C 2.603647 12.916888 10.739451

C 3.472185 13.925748 10.296043

C 3.609742 14.139941 8.918159

C 2.497654 11.554730 5.098782

C 3.758498 11.501443 4.273875

C 4.786411 10.622662 4.647816

C 5.959749 10.565587 3.890769

C 6.124677 11.402242 2.780991

C 5.101290 12.279925 2.408070

C 3.908924 12.308119 3.137234

C 1.227307 11.220742 4.241310

C 0.535948 9.966261 4.721459

C 0.787762 8.765329 4.040118

C 0.186023 7.579952 4.469798

C -0.671799 7.587197 5.574799

C -0.925335 8.783856 6.251857

C -0.321846 9.972102 5.829581

C -0.817679 12.622331 3.541414

C -0.453847 12.571614 2.074457

C -0.999005 11.544260 1.288420

C -0.700621 11.470393 -0.074985

C -1.266187 10.369486 -0.979783

C -2.041417 9.298866 -0.189542

C -2.222615 11.026817 -1.994816

C 0.137923 12.449811 -0.637647

C 0.681458 13.484011 0.131279

C 1.597957 14.552359 -0.480760

C 0.390943 13.526886 1.508270

C 0.994117 13.405776 5.188269

C 0.308907 15.106659 6.911200

C 1.207236 16.339590 7.136206

C 1.125466 16.814823 4.736288

C 0.196202 15.634054 4.463143

C 1.185442 18.707748 6.311144

C 2.934504 18.748518 8.177126

C 2.878891 19.020999 9.659978

C 4.099362 19.218812 10.319466

C 4.124069 19.449754 11.701787

C 5.450643 19.662498 12.440994

C 5.914929 18.292783 12.970316

C 5.264335 20.638596 13.622305

C 6.522856 20.250339 11.507334

C 2.912053 19.442598 12.407108

C 1.684484 19.228449 11.760250

C 0.397223 19.238966 12.592300

C 0.550042 18.258416 13.773248

C 0.176873 20.671870 13.117547

C -0.837031 18.816605 11.772319

C 1.675223 19.024829 10.374554

C 1.713919 20.801087 7.318286

C 0.736079 21.398224 8.303579

C 1.173075 22.429143 9.148476

C 0.279994 23.024483 10.043803

C -1.048720 22.592662 10.103281

C -1.483328 21.558794 9.267440

C -0.595037 20.960674 8.369673

C 1.278220 21.042694 5.835135

C 2.444802 21.453884 4.964940

C 2.450677 22.738461 4.398539

C 3.527174 23.144841 3.606415

C 4.598311 22.274612 3.368727

C 4.590165 20.993676 3.925898

C 3.517017 20.584327 4.724340

C -0.523686 19.615313 4.658545

C -0.367931 19.387673 3.169581

C -1.493579 18.894778 2.485601

C -1.440067 18.682549 1.105129

C -2.639428 18.091764 0.354136

C -2.470603 16.559715 0.373970

C -2.682644 18.592826 -1.102219

C -3.970407 18.478505 1.027531

C -0.240892 18.950010 0.420466

C 0.890671 19.422217 1.089977

C 0.809538 19.656941 2.476731

N 2.209639 12.949385 5.615056

N 0.363472 12.438181 4.415964

N 0.258846 14.575601 5.520006

N 0.860023 17.370450 6.107790

N 1.753589 19.291980 7.446300

N 0.758362 19.691864 5.408877

C 4.201744 14.809849 11.316159

C 5.478425 15.421533 10.725607

C 4.597946 13.988724 12.561915

C 3.216835 15.924403 11.722295

C 1.927594 9.866620 10.899369

C 0.059207 10.413409 9.315422

C 0.139221 11.530035 11.544049

C 1.535585 14.554628 -2.021063

C 3.036174 14.250915 -0.028798

C 1.163227 15.938994 0.023835

C 2.220830 19.690179 0.373562

C 2.192068 19.242738 -1.099968

C 3.335548 18.910222 1.091660

C 2.500966 21.204874 0.432657

C -0.102484 9.678162 -1.717812

H 2.212830 16.488517 4.667846

H 1.028864 17.578433 3.932708

H 0.477388 15.212368 3.454815

H -0.868880 15.961562 4.417578

H 2.299916 16.060935 7.060616

H 1.030613 16.733324 8.163668

H 0.597950 14.306543 7.633160

H -0.753508 15.374983 7.145905

H 1.495877 11.117384 3.151092

H 2.575454 10.867731 5.986030

H 1.454741 8.750299 3.176620

H -0.525866 10.900650 6.362931

H -1.592831 8.791500 7.113857

H -1.140171 6.661018 5.907828

H 0.386968 6.647362 3.941432

H 6.759053 9.884631 4.181239

H 4.683834 9.994412 5.531866

H 3.093435 12.971462 2.817024

H 5.239627 12.965235 1.565513

H 7.057145 11.382244 2.216814

H -1.340052 13.576678 3.785489

H -1.540919 11.806959 3.803531

H -1.652800 10.808425 1.753775

H 0.843525 14.311491 2.133645

H 0.369615 12.388465 -1.702790

H -1.406573 8.805359 0.554085

H -2.408093 18.676107 3.034326

H 1.687639 20.034762 3.005799

H -0.200635 18.770526 -0.655328

H -1.087957 20.571410 4.808915

H -1.161326 18.806719 5.096718

H 2.747040 21.214199 7.490867

H 0.456569 21.803199 5.779329

H -0.940008 20.150879 7.729718

H -2.517706 21.219086 9.315793

H -1.743802 23.059369 10.800868

H 0.623460 23.826064 10.698440

H 2.210097 22.766989 9.115986

H 1.622021 23.422643 4.572954

H 3.532580 24.143346 3.171475

H 5.435893 22.595846 2.750757

H 3.546094 19.567691 5.140455

H 5.412836 20.285874 3.754146

H 3.879800 19.194860 7.735591

H 3.042359 17.642812 8.013694

H 5.034544 19.163183 9.750420

H 2.922525 19.601274 13.486587

H 0.738679 18.881286 9.839719

H 4.292210 14.926861 8.554501

H 2.481614 12.752160 11.811464

H 1.544293 11.683733 7.752601

H 3.168477 14.720605 6.321505

H 4.210153 13.276747 6.273323

H 3.428033 21.454150 -0.094352

H 1.692322 21.780957 -0.026337

H 2.617454 21.555754 1.465537

H 3.478813 19.253729 2.129244

H 3.115973 17.830481 1.132644

H 4.303509 19.019912 0.590185

H 2.002570 18.158209 -1.180489

H 1.428937 19.770415 -1.678566

H 3.156467 19.432790 -1.584336

H -1.803670 18.270691 -1.672637

H -3.563617 18.209284 -1.627871

H -2.723646 19.686443 -1.147567

H -4.092927 19.566024 1.071637

H -4.824140 18.075065 0.472059

H -4.042274 18.093652 2.050140

H -1.476661 16.259972 -0.005194

H -2.562404 16.157371 1.387128

H -3.222146 16.064808 -0.248260

H 1.648720 16.748152 -0.545998

H 0.075807 16.086980 -0.050781

H 1.459799 16.093694 1.076768

H 3.737092 15.035399 -0.346438

H 3.113017 14.224989 1.073597

H 3.399823 13.297279 -0.416584

H 2.198412 15.324830 -2.432412

H 1.861402 13.599383 -2.445726

H 0.525222 14.763728 -2.384828

H 0.625740 9.262866 -1.012909

H -0.461628 8.854236 -2.343427

H 0.436707 10.369018 -2.374888

H -2.907580 9.721044 0.330504

H -2.420445 8.516865 -0.857505

H -1.708524 11.760351 -2.624783

H -2.666526 10.280952 -2.662482

H -3.043335 11.547449 -1.490430

H 2.594079 10.221126 11.693301

H 1.352542 9.028207 11.304147

H 2.563016 9.475543 10.096885

H 0.751631 11.871750 12.386227

H -0.476000 12.375451 11.221121

H -0.533956 10.757760 11.930357

H 0.614587 9.946482 8.493825

H -0.587659 9.637748 9.740745

H -0.591391 11.186104 8.895250

H 2.284023 15.519203 12.121383

H 3.650246 16.580561 12.485755

H 2.969626 16.569835 10.870004

H 5.179187 14.598771 13.261185

H 3.725473 13.612386 13.104307

H 5.217697 13.128550 12.286604

H 6.235919 14.662582 10.510241

H 5.293071 15.954824 9.769169

H 5.923282 16.166151 11.402983

H 6.787659 19.562765 10.687089

H 6.190617 21.188041 11.053660

H 7.454635 20.449557 12.045996

H 6.216414 20.826358 14.129520

H 4.875523 21.603651 13.282553

H 4.571144 20.245775 14.373528

H 6.893178 18.363634 13.455376

H 5.210026 17.879439 13.697471

H 6.011671 17.556968 12.150417

H 1.008003 21.006442 13.746693

H 0.086043 21.385581 12.289967

H -0.735541 20.740778 13.717474

H -0.728699 17.802387 11.375470

H -1.741132 18.829005 12.390315

H -1.012737 19.495552 10.929571

H 1.344906 18.566835 14.460659

H -0.373302 18.190255 14.356815

H 0.796765 17.250711 13.421728

S 9.185699 17.513766 6.727730

C 10.095307 16.900476 5.199156

C 10.174066 18.897569 7.408128

H 9.383454 16.118468 4.912898

C 11.372697 16.211327 5.642885

C 10.198561 17.880274 4.029904

H 11.168684 18.526348 7.675146

H 9.638198 19.161747 8.324304

C 10.293438 20.157294 6.564155

C 11.361542 14.817415 5.812017

C 12.557716 16.914485 5.919499

C 9.163334 18.788256 3.755539

C 11.269895 17.798740 3.126617

O 9.443243 21.013652 6.460075

O 11.499902 20.219405 5.963691

C 12.502933 14.144568 6.251895

H 10.451103 14.265397 5.595614

C 13.697263 16.239140 6.362002

H 12.591308 17.990576 5.777017

C 9.215341 19.605411 2.623666

H 8.315111 18.858670 4.424344

C 11.318640 18.614962 1.993891

H 12.072367 17.088471 3.300421

C 11.705310 21.351698 5.097402

C 13.674605 14.852271 6.531328

H 12.476169 13.064138 6.373263

H 14.606799 16.799260 6.570842

C 10.292415 19.525695 1.737568

H 8.403327 20.302196 2.435699

H 12.161835 18.532045 1.310331

H 12.737442 21.269585 4.753945

H 11.015227 21.304978 4.252314

H 11.556077 22.285418 5.646262

H 14.564520 14.327791 6.873692

H 10.329440 20.161946 0.855564

**TSR-04**

Mo 4.823269 15.937611 3.115449

Mo 5.659041 17.184784 6.505330

O 5.968009 13.595211 6.385251

O 8.045742 13.734130 4.976909

O 7.009523 15.782200 5.853209

O 5.861329 14.469176 4.085105

O 5.507346 15.684369 1.585255

O 3.240508 14.768515 3.217942

O 2.896167 16.114126 2.771079

O 4.850423 17.871377 3.298487

O 5.989466 17.330033 4.010042

O 4.214156 16.085836 5.534548

O 3.784398 17.414533 5.924685

O 5.366653 16.642440 8.094217

O 5.683861 19.051390 6.564604

O 7.273898 18.316365 6.692543

S 6.762991 14.311587 5.368835

C 3.044411 13.552692 6.677703

C 2.715196 13.342211 8.133385

C 1.818525 12.359601 8.571813

C 1.556980 12.203798 9.940756

C 0.609147 11.121361 10.470686

C 2.214518 13.038325 10.853600

C 3.130784 14.015537 10.430820

C 3.369290 14.168368 9.059799

C 2.393296 11.486812 5.250053

C 3.694989 11.365930 4.499816

C 4.656593 10.442122 4.935993

C 5.862053 10.315998 4.240366

C 6.123611 11.127655 3.130389

C 5.167569 12.053137 2.698404

C 3.943606 12.151753 3.366119

C 1.164970 11.183493 4.322126

C 0.397614 9.965679 4.780083

C 0.607706 8.752477 4.106728

C -0.066128 7.600052 4.519090

C -0.953784 7.652926 5.598892

C -1.165575 8.862185 6.268141

C -0.490719 10.017307 5.862833

C -0.786534 12.643585 3.485047

C -0.351151 12.552717 2.039516

C -0.892724 11.528064 1.247220

C -0.535064 11.418440 -0.099336

C -1.097964 10.320565 -1.009379

C -1.893061 9.259399 -0.226071

C -2.033971 10.987659 -2.037013

C 0.356418 12.362205 -0.640686

C 0.896666 13.394050 0.133717

C 1.867622 14.424944 -0.459085

C 0.549010 13.470186 1.496290

C 0.957884 13.391827 5.218380

C 0.230457 15.130133 6.881880

C 1.161259 16.329771 7.147492

C 1.215376 16.794966 4.741552

C 0.272795 15.635487 4.425584

C 1.242018 18.691127 6.312821

C 2.959385 18.689906 8.193712

C 2.954266 18.989851 9.672012

C 4.207814 19.071542 10.298987

C 4.290872 19.333352 11.670194

C 5.633601 19.444099 12.402709

C 5.738760 18.261790 13.386418

C 5.677572 20.781960 13.170797

C 6.834552 19.394834 11.442799

C 3.100020 19.484599 12.401306

C 1.842516 19.374892 11.791875

C 0.582176 19.540816 12.648345

C 0.698550 18.660487 13.909821

C 0.471102 21.025425 13.049027

C -0.696332 19.128965 11.893515

C 1.777313 19.135196 10.411295

C 1.784529 20.769744 7.338713

C 0.769736 21.400455 8.263915

C 1.169372 22.480111 9.065561

C 0.242128 23.113143 9.898092

C -1.083229 22.668957 9.940384

C -1.480613 21.586878 9.148363

C -0.558739 20.952855 8.310999

C 1.426995 21.016180 5.834642

C 2.648624 21.369200 5.016032

C 2.746625 22.655942 4.462909

C 3.874780 23.009719 3.718997

C 4.908283 22.086052 3.519155

C 4.809691 20.804300 4.065202

C 3.682081 20.445422 4.811408

C -0.384692 19.641570 4.603676

C -0.187180 19.368786 3.127801

C -1.290084 18.848500 2.427390

C -1.196976 18.598245 1.055530

C -2.370685 17.981787 0.284929

C -2.178886 16.452914 0.320637

C -2.388776 18.471805 -1.175805

C -3.722466 18.351833 0.925397

C 0.019613 18.854223 0.397532

C 1.128458 19.354631 1.084124

C 1.007425 19.628097 2.460945

N 2.130446 12.901558 5.722495

N 0.340265 12.435307 4.423420

N 0.242946 14.588055 5.494372

N 0.899843 17.362770 6.097079

N 1.782584 19.259823 7.474572

N 0.871873 19.683440 5.398181

C 3.803799 14.909554 11.480582

C 5.021260 15.649308 10.918757

C 4.269876 14.050939 12.676134

C 2.752300 15.935728 11.949373

C 1.474922 10.010264 11.096986

C -0.265292 10.510237 9.359491

C -0.328020 11.724089 11.537291

C 1.865273 14.402285 -2.000742

C 3.277657 14.089706 0.053216

C 1.452410 15.832065 0.003822

C 2.477775 19.607894 0.398814

C 2.480906 19.156599 -1.073679

C 3.564095 18.817474 1.147913

C 2.775149 21.119415 0.460677

C 0.065964 9.614453 -1.732880

H 2.296977 16.442905 4.726515

H 1.171928 17.558596 3.932897

H 0.603443 15.195686 3.439839

H -0.778183 15.991085 4.311652

H 2.246397 16.014836 7.126243

H 0.949154 16.736700 8.162939

H 0.444481 14.326639 7.625575

H -0.832742 15.441287 7.051474

H 1.494350 11.050335 3.251882

H 2.391359 10.816048 6.152633

H 1.297942 8.701955 3.262944

H -0.662598 10.956322 6.388912

H -1.856310 8.905236 7.110560

H -1.478270 6.752423 5.918472

H 0.101946 6.657566 3.996812

H 6.610052 9.599309 4.577390

H 4.476244 9.830488 5.818986

H 3.181075 12.852389 3.000544

H 5.382275 12.724611 1.861211

H 7.080403 11.052122 2.613304

H -1.286140 13.620090 3.684853

H -1.551206 11.859166 3.723010

H -1.591417 10.822588 1.693996

H 1.001395 14.249892 2.128188

H 0.632276 12.274688 -1.693537

H -1.273049 8.766478 0.530325

H -2.217687 18.639278 2.957205

H 1.867644 20.027014 3.003495

H 0.091122 18.643010 -0.670948

H -0.911261 20.624715 4.710379

H -1.071120 18.872746 5.040266

H 2.815752 21.161033 7.562918

H 0.640908 21.808923 5.735070

H -0.876207 20.108139 7.702620

H -2.512367 21.237557 9.183166

H -1.804334 23.163694 10.590608

H 0.556116 23.954393 10.516925

H 2.203488 22.827984 9.047430

H 1.949452 23.382552 4.610431

H 3.950362 24.009081 3.292518

H 5.786401 22.366376 2.938612

H 3.639772 19.423122 5.212263

H 5.600006 20.053262 3.923734

H 3.910930 19.096390 7.725786

H 3.025053 17.578133 8.049067

H 5.111468 18.904589 9.702068

H 3.158245 19.686229 13.471646

H 0.816284 19.073339 9.904304

H 4.092540 14.921237 8.703826

H 2.019174 12.921309 11.921036

H 1.336718 11.702027 7.853676

H 3.129467 14.653976 6.424681

H 4.105904 13.172910 6.478095

H 3.714663 21.356131 -0.050135

H 1.982234 21.704337 -0.014204

H 2.878211 21.471301 1.494467

H 3.685035 19.164693 2.186939

H 3.331171 17.740268 1.186803

H 4.545921 18.913228 0.671172

H 2.294850 18.071736 -1.156005

H 1.730444 19.682593 -1.669997

H 3.455686 19.345863 -1.537380

H -1.492460 18.159067 -1.724101

H -3.251834 18.071270 -1.718199

H -2.444791 19.564312 -1.230174

H -3.863505 19.437515 0.958612

H -4.556809 17.930963 0.353553

H -3.811437 17.972606 1.948707

H -1.176550 16.164458 -0.045455

H -2.275234 16.058263 1.336307

H -2.915906 15.941066 -0.305287

H 1.976941 16.618517 -0.562399

H 0.371988 16.004570 -0.109652

H 1.716184 15.998537 1.063933

H 4.010045 14.857514 -0.234827

H 3.308519 14.063393 1.157692

H 3.634771 13.127311 -0.318315

H 2.566123 15.146045 -2.397588

H 2.179232 13.431120 -2.397095

H 0.876557 14.634264 -2.407375

H 0.783410 9.195720 -1.019035

H -0.295052 8.791117 -2.358148

H 0.617938 10.297009 -2.388225

H -2.764483 9.689846 0.278196

H -2.266731 8.476382 -0.895779

H -1.504214 11.719606 -2.655841

H -2.472578 10.247164 -2.714007

H -2.858692 11.512784 -1.544011

H 2.087338 10.390234 11.921935

H 0.858092 9.198113 11.494125

H 2.159845 9.575383 10.360582

H 0.227097 12.093918 12.406756

H -0.904116 12.562752 11.134408

H -1.040559 10.978815 11.905890

H 0.339758 10.003280 8.599057

H -0.950761 9.759824 9.769666

H -0.875072 11.270940 8.862980

H 1.873181 15.454390 12.383559

H 3.171482 16.614347 12.701997

H 2.419261 16.569487 11.118168

H 4.811924 14.659846 13.407258

H 3.432101 13.581637 13.200425

H 4.946574 13.254303 12.349077

H 5.814149 14.962619 10.583325

H 4.775568 16.279696 10.042694

H 5.470051 16.324519 11.665818

H 6.919790 18.413260 10.950352

H 6.771290 20.159173 10.661942

H 7.775733 19.552623 11.979542

H 6.635975 20.910029 13.684409

H 5.551902 21.631091 12.491336

H 4.892019 20.846036 13.929864

H 6.693989 18.270579 13.919615

H 4.940222 18.271224 14.132331

H 5.675875 17.300454 12.842387

H 1.346314 21.358624 13.616115

H 0.390879 21.667907 12.164301

H -0.410792 21.205733 13.671046

H -0.655257 18.082227 11.576380

H -1.581090 19.243108 12.528658

H -0.856676 19.748185 11.002964

H 1.526957 18.975278 14.553420

H -0.213696 18.705857 14.512736

H 0.871244 17.611406 13.645732

S 9.370297 17.519533 7.013508

C 10.038234 19.189458 6.465714

C 9.262453 17.670437 8.829417

H 9.293935 19.465389 5.714649

C 11.383853 19.053436 5.766117

C 10.003072 20.263761 7.545123

H 8.666271 18.545599 9.096073

H 10.259768 17.805170 9.269522

C 8.625521 16.465875 9.509268

C 11.623557 19.842028 4.631471

C 12.405170 18.208402 6.219694

C 8.804783 20.959441 7.777697

C 11.128777 20.585883 8.316411

O 8.225307 16.509133 10.654533

O 8.578545 15.386034 8.724517

C 12.853040 19.796464 3.972363

H 10.839118 20.498171 4.262172

C 13.637887 18.162875 5.564524

H 12.229868 17.567240 7.078354

C 8.737995 21.948692 8.760226

H 7.923948 20.706813 7.198531

C 11.059972 21.575862 9.299742

H 12.070405 20.073657 8.138615

C 7.956397 14.210291 9.280857

C 13.867077 18.956628 4.438660

H 13.015374 20.412160 3.090672

H 14.416987 17.498404 5.931462

C 9.864626 22.261342 9.525531

H 7.803249 22.478493 8.924188

H 11.946034 21.814482 9.884806

H 7.776639 13.548182 8.437101

H 8.630253 13.747238 10.010284

H 7.010857 14.480021 9.767539

H 14.824814 18.915039 3.924421

H 9.813081 23.035585 10.288198

**TSR-05**

Mo 4.815670 15.933621 3.115344

Mo 5.663963 17.189455 6.494667

O 5.970105 13.594413 6.385910

O 8.041751 13.727779 4.969200

O 7.011826 15.779098 5.841043

O 5.853950 14.463478 4.084174

O 5.498659 15.679176 1.584796

O 3.232620 14.764447 3.220971

O 2.888224 16.108646 2.769784

O 4.838966 17.866511 3.298755

O 5.980004 17.327496 4.007452

O 4.210268 16.087774 5.537658

O 3.785135 17.418554 5.927034

O 5.388322 16.638433 8.084758

O 5.684546 19.053513 6.577979

O 7.284826 18.315171 6.645257

S 6.760890 14.308732 5.364481

C 3.044411 13.552692 6.677703

C 2.715196 13.342211 8.133385

C 1.818525 12.359601 8.571813

C 1.556980 12.203798 9.940756

C 0.609147 11.121361 10.470686

C 2.214518 13.038325 10.853600

C 3.130784 14.015537 10.430820

C 3.369290 14.168368 9.059799

C 2.393296 11.486812 5.250053

C 3.694989 11.365930 4.499816

C 4.656593 10.442122 4.935993

C 5.862053 10.315998 4.240366

C 6.123611 11.127655 3.130389

C 5.167569 12.053137 2.698404

C 3.943606 12.151753 3.366119

C 1.164970 11.183493 4.322126

C 0.397614 9.965679 4.780083

C 0.607706 8.752477 4.106728

C -0.066128 7.600052 4.519090

C -0.953784 7.652926 5.598892

C -1.165575 8.862185 6.268141

C -0.490719 10.017307 5.862833

C -0.786534 12.643585 3.485047

C -0.351151 12.552717 2.039516

C -0.892724 11.528064 1.247220

C -0.535064 11.418440 -0.099336

C -1.097964 10.320565 -1.009379

C -1.893061 9.259399 -0.226071

C -2.033971 10.987659 -2.037013

C 0.356418 12.362205 -0.640686

C 0.896666 13.394050 0.133717

C 1.867622 14.424944 -0.459085

C 0.549010 13.470186 1.496290

C 0.957884 13.391827 5.218380

C 0.230457 15.130133 6.881880

C 1.161259 16.329771 7.147492

C 1.215376 16.794966 4.741552

C 0.272795 15.635487 4.425584

C 1.242018 18.691127 6.312821

C 2.959385 18.689906 8.193712

C 2.954266 18.989851 9.672012

C 4.207814 19.071542 10.298987

C 4.290872 19.333352 11.670194

C 5.633601 19.444099 12.402709

C 5.738760 18.261790 13.386418

C 5.677572 20.781960 13.170797

C 6.834552 19.394834 11.442799

C 3.100020 19.484599 12.401306

C 1.842516 19.374892 11.791875

C 0.582176 19.540816 12.648345

C 0.698550 18.660487 13.909821

C 0.471102 21.025425 13.049027

C -0.696332 19.128965 11.893515

C 1.777313 19.135196 10.411295

C 1.784529 20.769744 7.338713

C 0.769736 21.400455 8.263915

C 1.169372 22.480111 9.065561

C 0.242128 23.113143 9.898092

C -1.083229 22.668957 9.940384

C -1.480613 21.586878 9.148363

C -0.558739 20.952855 8.310999

C 1.426995 21.016180 5.834642

C 2.648624 21.369200 5.016032

C 2.746625 22.655942 4.462909

C 3.874780 23.009719 3.718997

C 4.908283 22.086052 3.519155

C 4.809691 20.804300 4.065202

C 3.682081 20.445422 4.811408

C -0.384692 19.641570 4.603676

C -0.187180 19.368786 3.127801

C -1.290084 18.848500 2.427390

C -1.196976 18.598245 1.055530

C -2.370685 17.981787 0.284929

C -2.178886 16.452914 0.320637

C -2.388776 18.471805 -1.175805

C -3.722466 18.351833 0.925397

C 0.019613 18.854223 0.397532

C 1.128458 19.354631 1.084124

C 1.007425 19.628097 2.460945

N 2.130446 12.901558 5.722495

N 0.340265 12.435307 4.423420

N 0.242946 14.588055 5.494372

N 0.899843 17.362770 6.097079

N 1.782584 19.259823 7.474572

N 0.871873 19.683440 5.398181

C 3.803799 14.909554 11.480582

C 5.021260 15.649308 10.918757

C 4.269876 14.050939 12.676134

C 2.752300 15.935728 11.949373

C 1.474922 10.010264 11.096986

C -0.265292 10.510237 9.359491

C -0.328020 11.724089 11.537291

C 1.865273 14.402285 -2.000742

C 3.277657 14.089706 0.053216

C 1.452410 15.832065 0.003822

C 2.477775 19.607894 0.398814

C 2.480906 19.156599 -1.073679

C 3.564095 18.817474 1.147913

C 2.775149 21.119415 0.460677

C 0.065964 9.614453 -1.732880

H 2.296977 16.442905 4.726515

H 1.171928 17.558596 3.932897

H 0.603443 15.195686 3.439839

H -0.778183 15.991085 4.311652

H 2.246397 16.014836 7.126243

H 0.949154 16.736700 8.162939

H 0.444481 14.326639 7.625575

H -0.832742 15.441287 7.051474

H 1.494350 11.050335 3.251882

H 2.391359 10.816048 6.152633

H 1.297942 8.701955 3.262944

H -0.662598 10.956322 6.388912

H -1.856310 8.905236 7.110560

H -1.478270 6.752423 5.918472

H 0.101946 6.657566 3.996812

H 6.610052 9.599309 4.577390

H 4.476244 9.830488 5.818986

H 3.181075 12.852389 3.000544

H 5.382275 12.724611 1.861211

H 7.080403 11.052122 2.613304

H -1.286140 13.620090 3.684853

H -1.551206 11.859166 3.723010

H -1.591417 10.822588 1.693996

H 1.001395 14.249892 2.128188

H 0.632276 12.274688 -1.693537

H -1.273049 8.766478 0.530325

H -2.217687 18.639278 2.957205

H 1.867644 20.027014 3.003495

H 0.091122 18.643010 -0.670948

H -0.911261 20.624715 4.710379

H -1.071120 18.872746 5.040266

H 2.815752 21.161033 7.562918

H 0.640908 21.808923 5.735070

H -0.876207 20.108139 7.702620

H -2.512367 21.237557 9.183166

H -1.804334 23.163694 10.590608

H 0.556116 23.954393 10.516925

H 2.203488 22.827984 9.047430

H 1.949452 23.382552 4.610431

H 3.950362 24.009081 3.292518

H 5.786401 22.366376 2.938612

H 3.639772 19.423122 5.212263

H 5.600006 20.053262 3.923734

H 3.910930 19.096390 7.725786

H 3.025053 17.578133 8.049067

H 5.111468 18.904589 9.702068

H 3.158245 19.686229 13.471646

H 0.816284 19.073339 9.904304

H 4.092540 14.921237 8.703826

H 2.019174 12.921309 11.921036

H 1.336718 11.702027 7.853676

H 3.129467 14.653976 6.424681

H 4.105904 13.172910 6.478095

H 3.714663 21.356131 -0.050135

H 1.982234 21.704337 -0.014204

H 2.878211 21.471301 1.494467

H 3.685035 19.164693 2.186939

H 3.331171 17.740268 1.186803

H 4.545921 18.913228 0.671172

H 2.294850 18.071736 -1.156005

H 1.730444 19.682593 -1.669997

H 3.455686 19.345863 -1.537380

H -1.492460 18.159067 -1.724101

H -3.251834 18.071270 -1.718199

H -2.444791 19.564312 -1.230174

H -3.863505 19.437515 0.958612

H -4.556809 17.930963 0.353553

H -3.811437 17.972606 1.948707

H -1.176550 16.164458 -0.045455

H -2.275234 16.058263 1.336307

H -2.915906 15.941066 -0.305287

H 1.976941 16.618517 -0.562399

H 0.371988 16.004570 -0.109652

H 1.716184 15.998537 1.063933

H 4.010045 14.857514 -0.234827

H 3.308519 14.063393 1.157692

H 3.634771 13.127311 -0.318315

H 2.566123 15.146045 -2.397588

H 2.179232 13.431120 -2.397095

H 0.876557 14.634264 -2.407375

H 0.783410 9.195720 -1.019035

H -0.295052 8.791117 -2.358148

H 0.617938 10.297009 -2.388225

H -2.764483 9.689846 0.278196

H -2.266731 8.476382 -0.895779

H -1.504214 11.719606 -2.655841

H -2.472578 10.247164 -2.714007

H -2.858692 11.512784 -1.544011

H 2.087338 10.390234 11.921935

H 0.858092 9.198113 11.494125

H 2.159845 9.575383 10.360582

H 0.227097 12.093918 12.406756

H -0.904116 12.562752 11.134408

H -1.040559 10.978815 11.905890

H 0.339758 10.003280 8.599057

H -0.950761 9.759824 9.769666

H -0.875072 11.270940 8.862980

H 1.873181 15.454390 12.383559

H 3.171482 16.614347 12.701997

H 2.419261 16.569487 11.118168

H 4.811924 14.659846 13.407258

H 3.432101 13.581637 13.200425

H 4.946574 13.254303 12.349077

H 5.814149 14.962619 10.583325

H 4.775568 16.279696 10.042694

H 5.470051 16.324519 11.665818

H 6.919790 18.413260 10.950352

H 6.771290 20.159173 10.661942

H 7.775733 19.552623 11.979542

H 6.635975 20.910029 13.684409

H 5.551902 21.631091 12.491336

H 4.892019 20.846036 13.929864

H 6.693989 18.270579 13.919615

H 4.940222 18.271224 14.132331

H 5.675875 17.300454 12.842387

H 1.346314 21.358624 13.616115

H 0.390879 21.667907 12.164301

H -0.410792 21.205733 13.671046

H -0.655257 18.082227 11.576380

H -1.581090 19.243108 12.528658

H -0.856676 19.748185 11.002964

H 1.526957 18.975278 14.553420

H -0.213696 18.705857 14.512736

H 0.871244 17.611406 13.645732

S 9.358549 17.480073 6.908890

C 10.071070 19.126633 6.445864

C 9.254597 17.699600 8.713972

H 9.328678 19.860858 6.774914

C 10.180303 19.224128 4.927745

C 11.385440 19.417756 7.158428

H 8.647946 18.582245 8.938342

H 10.254971 17.862076 9.137470

C 8.629414 16.518261 9.443797

C 9.993179 20.479723 4.332080

C 10.499523 18.133758 4.107677

C 11.435221 20.415998 8.140658

C 12.561793 18.714596 6.857119

O 8.236219 16.595693 10.589380

O 8.585023 15.414903 8.692205

C 10.129218 20.646283 2.954218

H 9.743489 21.334929 4.956155

C 10.631215 18.298257 2.726395

H 10.636709 17.146016 4.536776

C 12.628977 20.708621 8.807121

H 10.533379 20.973147 8.383212

C 13.753304 19.003246 7.521389

H 12.545375 17.942391 6.093657

C 7.972427 14.251007 9.282219

C 10.449565 19.553454 2.144820

H 9.980910 21.629212 2.512774

H 10.873945 17.438620 2.107027

C 13.792253 20.002241 8.499914

H 12.646458 21.488849 9.564681

H 14.655759 18.449330 7.272712

H 7.781539 13.571238 8.454721

H 8.657632 13.804081 10.011151

H 7.033393 14.529709 9.776176

H 10.553406 19.679328 1.069578

H 14.723230 20.227312 9.015698

**TSR-06**

Mo 4.843683 15.924365 3.122927

Mo 5.659257 17.240072 6.487666

O 5.927987 13.613179 6.404850

O 8.058474 13.736826 5.076647

O 7.017585 15.791760 5.920128

O 5.915962 14.492589 4.106132

O 5.523263 15.658680 1.593544

O 3.270281 14.747296 3.246462

O 2.914281 16.084218 2.782414

O 4.854846 17.859892 3.285336

O 5.996157 17.332039 4.004234

O 4.227981 16.100851 5.561753

O 3.775884 17.431420 5.921929

O 5.424145 16.748174 8.103331

O 5.663680 19.108139 6.531981

O 7.268879 18.389812 6.508231

S 6.766303 14.323069 5.422890

C 3.044411 13.552692 6.677703

C 2.715196 13.342211 8.133385

C 1.818525 12.359601 8.571813

C 1.556980 12.203798 9.940756

C 0.609147 11.121361 10.470686

C 2.214518 13.038325 10.853600

C 3.130784 14.015537 10.430820

C 3.369290 14.168368 9.059799

C 2.393296 11.486812 5.250053

C 3.694989 11.365930 4.499816

C 4.656593 10.442122 4.935993

C 5.862053 10.315998 4.240366

C 6.123611 11.127655 3.130389

C 5.167569 12.053137 2.698404

C 3.943606 12.151753 3.366119

C 1.164970 11.183493 4.322126

C 0.397614 9.965679 4.780083

C 0.607706 8.752477 4.106728

C -0.066128 7.600052 4.519090

C -0.953784 7.652926 5.598892

C -1.165575 8.862185 6.268141

C -0.490719 10.017307 5.862833

C -0.786534 12.643585 3.485047

C -0.351151 12.552717 2.039516

C -0.892724 11.528064 1.247220

C -0.535064 11.418440 -0.099336

C -1.097964 10.320565 -1.009379

C -1.893061 9.259399 -0.226071

C -2.033971 10.987659 -2.037013

C 0.356418 12.362205 -0.640686

C 0.896666 13.394050 0.133717

C 1.867622 14.424944 -0.459085

C 0.549010 13.470186 1.496290

C 0.957884 13.391827 5.218380

C 0.230457 15.130133 6.881880

C 1.161259 16.329771 7.147492

C 1.215376 16.794966 4.741552

C 0.272795 15.635487 4.425584

C 1.242018 18.691127 6.312821

C 2.959385 18.689906 8.193712

C 2.954266 18.989851 9.672012

C 4.207814 19.071542 10.298987

C 4.290872 19.333352 11.670194

C 5.633601 19.444099 12.402709

C 5.738760 18.261790 13.386418

C 5.677572 20.781960 13.170797

C 6.834552 19.394834 11.442799

C 3.100020 19.484599 12.401306

C 1.842516 19.374892 11.791875

C 0.582176 19.540816 12.648345

C 0.698550 18.660487 13.909821

C 0.471102 21.025425 13.049027

C -0.696332 19.128965 11.893515

C 1.777313 19.135196 10.411295

C 1.784529 20.769744 7.338713

C 0.769736 21.400455 8.263915

C 1.169372 22.480111 9.065561

C 0.242128 23.113143 9.898092

C -1.083229 22.668957 9.940384

C -1.480613 21.586878 9.148363

C -0.558739 20.952855 8.310999

C 1.426995 21.016180 5.834642

C 2.648624 21.369200 5.016032

C 2.746625 22.655942 4.462909

C 3.874780 23.009719 3.718997

C 4.908283 22.086052 3.519155

C 4.809691 20.804300 4.065202

C 3.682081 20.445422 4.811408

C -0.384692 19.641570 4.603676

C -0.187180 19.368786 3.127801

C -1.290084 18.848500 2.427390

C -1.196976 18.598245 1.055530

C -2.370685 17.981787 0.284929

C -2.178886 16.452914 0.320637

C -2.388776 18.471805 -1.175805

C -3.722466 18.351833 0.925397

C 0.019613 18.854223 0.397532

C 1.128458 19.354631 1.084124

C 1.007425 19.628097 2.460945

N 2.130446 12.901558 5.722495

N 0.340265 12.435307 4.423420

N 0.242946 14.588055 5.494372

N 0.899843 17.362770 6.097079

N 1.782584 19.259823 7.474572

N 0.871873 19.683440 5.398181

C 3.803799 14.909554 11.480582

C 5.021260 15.649308 10.918757

C 4.269876 14.050939 12.676134

C 2.752300 15.935728 11.949373

C 1.474922 10.010264 11.096986

C -0.265292 10.510237 9.359491

C -0.328020 11.724089 11.537291

C 1.865273 14.402285 -2.000742

C 3.277657 14.089706 0.053216

C 1.452410 15.832065 0.003822

C 2.477775 19.607894 0.398814

C 2.480906 19.156599 -1.073679

C 3.564095 18.817474 1.147913

C 2.775149 21.119415 0.460677

C 0.065964 9.614453 -1.732880

H 2.296977 16.442905 4.726515

H 1.171928 17.558596 3.932897

H 0.603443 15.195686 3.439839

H -0.778183 15.991085 4.311652

H 2.246397 16.014836 7.126243

H 0.949154 16.736700 8.162939

H 0.444481 14.326639 7.625575

H -0.832742 15.441287 7.051474

H 1.494350 11.050335 3.251882

H 2.391359 10.816048 6.152633

H 1.297942 8.701955 3.262944

H -0.662598 10.956322 6.388912

H -1.856310 8.905236 7.110560

H -1.478270 6.752423 5.918472

H 0.101946 6.657566 3.996812

H 6.610052 9.599309 4.577390

H 4.476244 9.830488 5.818986

H 3.181075 12.852389 3.000544

H 5.382275 12.724611 1.861211

H 7.080403 11.052122 2.613304

H -1.286140 13.620090 3.684853

H -1.551206 11.859166 3.723010

H -1.591417 10.822588 1.693996

H 1.001395 14.249892 2.128188

H 0.632276 12.274688 -1.693537

H -1.273049 8.766478 0.530325

H -2.217687 18.639278 2.957205

H 1.867644 20.027014 3.003495

H 0.091122 18.643010 -0.670948

H -0.911261 20.624715 4.710379

H -1.071120 18.872746 5.040266

H 2.815752 21.161033 7.562918

H 0.640908 21.808923 5.735070

H -0.876207 20.108139 7.702620

H -2.512367 21.237557 9.183166

H -1.804334 23.163694 10.590608

H 0.556116 23.954393 10.516925

H 2.203488 22.827984 9.047430

H 1.949452 23.382552 4.610431

H 3.950362 24.009081 3.292518

H 5.786401 22.366376 2.938612

H 3.639772 19.423122 5.212263

H 5.600006 20.053262 3.923734

H 3.910930 19.096390 7.725786

H 3.025053 17.578133 8.049067

H 5.111468 18.904589 9.702068

H 3.158245 19.686229 13.471646

H 0.816284 19.073339 9.904304

H 4.092540 14.921237 8.703826

H 2.019174 12.921309 11.921036

H 1.336718 11.702027 7.853676

H 3.129467 14.653976 6.424681

H 4.105904 13.172910 6.478095

H 3.714663 21.356131 -0.050135

H 1.982234 21.704337 -0.014204

H 2.878211 21.471301 1.494467

H 3.685035 19.164693 2.186939

H 3.331171 17.740268 1.186803

H 4.545921 18.913228 0.671172

H 2.294850 18.071736 -1.156005

H 1.730444 19.682593 -1.669997

H 3.455686 19.345863 -1.537380

H -1.492460 18.159067 -1.724101

H -3.251834 18.071270 -1.718199

H -2.444791 19.564312 -1.230174

H -3.863505 19.437515 0.958612

H -4.556809 17.930963 0.353553

H -3.811437 17.972606 1.948707

H -1.176550 16.164458 -0.045455

H -2.275234 16.058263 1.336307

H -2.915906 15.941066 -0.305287

H 1.976941 16.618517 -0.562399

H 0.371988 16.004570 -0.109652

H 1.716184 15.998537 1.063933

H 4.010045 14.857514 -0.234827

H 3.308519 14.063393 1.157692

H 3.634771 13.127311 -0.318315

H 2.566123 15.146045 -2.397588

H 2.179232 13.431120 -2.397095

H 0.876557 14.634264 -2.407375

H 0.783410 9.195720 -1.019035

H -0.295052 8.791117 -2.358148

H 0.617938 10.297009 -2.388225

H -2.764483 9.689846 0.278196

H -2.266731 8.476382 -0.895779

H -1.504214 11.719606 -2.655841

H -2.472578 10.247164 -2.714007

H -2.858692 11.512784 -1.544011

H 2.087338 10.390234 11.921935

H 0.858092 9.198113 11.494125

H 2.159845 9.575383 10.360582

H 0.227097 12.093918 12.406756

H -0.904116 12.562752 11.134408

H -1.040559 10.978815 11.905890

H 0.339758 10.003280 8.599057

H -0.950761 9.759824 9.769666

H -0.875072 11.270940 8.862980

H 1.873181 15.454390 12.383559

H 3.171482 16.614347 12.701997

H 2.419261 16.569487 11.118168

H 4.811924 14.659846 13.407258

H 3.432101 13.581637 13.200425

H 4.946574 13.254303 12.349077

H 5.814149 14.962619 10.583325

H 4.775568 16.279696 10.042694

H 5.470051 16.324519 11.665818

H 6.919790 18.413260 10.950352

H 6.771290 20.159173 10.661942

H 7.775733 19.552623 11.979542

H 6.635975 20.910029 13.684409

H 5.551902 21.631091 12.491336

H 4.892019 20.846036 13.929864

H 6.693989 18.270579 13.919615

H 4.940222 18.271224 14.132331

H 5.675875 17.300454 12.842387

H 1.346314 21.358624 13.616115

H 0.390879 21.667907 12.164301

H -0.410792 21.205733 13.671046

H -0.655257 18.082227 11.576380

H -1.581090 19.243108 12.528658

H -0.856676 19.748185 11.002964

H 1.526957 18.975278 14.553420

H -0.213696 18.705857 14.512736

H 0.871244 17.611406 13.645732

S 9.386580 17.576613 6.519628

C 10.005410 19.347195 6.468490

C 9.241821 17.128579 8.271662

H 9.152219 19.853691 6.009830

C 11.190337 19.475833 5.520203

C 10.217827 19.985114 7.831896

H 8.581087 16.254060 8.261137

H 8.716092 17.910340 8.824402

C 10.476377 16.743251 9.073245

C 11.232782 20.582798 4.660242

C 12.250126 18.558917 5.485594

C 9.128806 20.606042 8.464959

C 11.464468 19.995775 8.473808

O 10.434883 16.582136 10.275182

O 11.584720 16.564144 8.335078

C 12.311193 20.779176 3.796268

H 10.413507 21.297686 4.668765

C 13.330646 18.754512 4.622004

H 12.219262 17.684943 6.128002

C 9.286281 21.225467 9.706265

H 8.158721 20.597029 7.978530

C 11.619181 20.609889 9.718529

H 12.323226 19.540182 7.990078

C 12.753854 16.158815 9.070631

C 13.366396 19.864239 3.774871

H 12.322556 21.642486 3.134872

H 14.143269 18.031481 4.606915

C 10.531579 21.228814 10.338784

H 8.435269 21.712413 10.176312

H 12.595516 20.612790 10.198940

H 13.543601 16.053507 8.326080

H 13.021546 16.918492 9.810624

H 12.575907 15.207554 9.578418

H 14.205801 20.011111 3.098613

H 10.655480 21.714264 11.304385

**TSR-07**

Mo 4.838798 15.929556 3.118904

Mo 5.654394 17.228839 6.484308

O 5.933889 13.612855 6.392513

O 8.059211 13.731949 5.054673

O 7.025022 15.788761 5.903910

O 5.914408 14.491430 4.093165

O 5.519416 15.671375 1.588411

O 3.267279 14.749037 3.235920

O 2.909317 16.088052 2.779484

O 4.849391 17.863985 3.290422

O 5.991380 17.334395 4.005862

O 4.224857 16.090725 5.548295

O 3.772270 17.418842 5.917607

O 5.416399 16.710076 8.091849

O 5.646723 19.091521 6.557780

O 7.263354 18.380550 6.526871

S 6.768743 14.319729 5.405333

C 3.044411 13.552692 6.677703

C 2.715196 13.342211 8.133385

C 1.818525 12.359601 8.571813

C 1.556980 12.203798 9.940756

C 0.609147 11.121361 10.470686

C 2.214518 13.038325 10.853600

C 3.130784 14.015537 10.430820

C 3.369290 14.168368 9.059799

C 2.393296 11.486812 5.250053

C 3.694989 11.365930 4.499816

C 4.656593 10.442122 4.935993

C 5.862053 10.315998 4.240366

C 6.123611 11.127655 3.130389

C 5.167569 12.053137 2.698404

C 3.943606 12.151753 3.366119

C 1.164970 11.183493 4.322126

C 0.397614 9.965679 4.780083

C 0.607706 8.752477 4.106728

C -0.066128 7.600052 4.519090

C -0.953784 7.652926 5.598892

C -1.165575 8.862185 6.268141

C -0.490719 10.017307 5.862833

C -0.786534 12.643585 3.485047

C -0.351151 12.552717 2.039516

C -0.892724 11.528064 1.247220

C -0.535064 11.418440 -0.099336

C -1.097964 10.320565 -1.009379

C -1.893061 9.259399 -0.226071

C -2.033971 10.987659 -2.037013

C 0.356418 12.362205 -0.640686

C 0.896666 13.394050 0.133717

C 1.867622 14.424944 -0.459085

C 0.549010 13.470186 1.496290

C 0.957884 13.391827 5.218380

C 0.230457 15.130133 6.881880

C 1.161259 16.329771 7.147492

C 1.215376 16.794966 4.741552

C 0.272795 15.635487 4.425584

C 1.242018 18.691127 6.312821

C 2.959385 18.689906 8.193712

C 2.954266 18.989851 9.672012

C 4.207814 19.071542 10.298987

C 4.290872 19.333352 11.670194

C 5.633601 19.444099 12.402709

C 5.738760 18.261790 13.386418

C 5.677572 20.781960 13.170797

C 6.834552 19.394834 11.442799

C 3.100020 19.484599 12.401306

C 1.842516 19.374892 11.791875

C 0.582176 19.540816 12.648345

C 0.698550 18.660487 13.909821

C 0.471102 21.025425 13.049027

C -0.696332 19.128965 11.893515

C 1.777313 19.135196 10.411295

C 1.784529 20.769744 7.338713

C 0.769736 21.400455 8.263915

C 1.169372 22.480111 9.065561

C 0.242128 23.113143 9.898092

C -1.083229 22.668957 9.940384

C -1.480613 21.586878 9.148363

C -0.558739 20.952855 8.310999

C 1.426995 21.016180 5.834642

C 2.648624 21.369200 5.016032

C 2.746625 22.655942 4.462909

C 3.874780 23.009719 3.718997

C 4.908283 22.086052 3.519155

C 4.809691 20.804300 4.065202

C 3.682081 20.445422 4.811408

C -0.384692 19.641570 4.603676

C -0.187180 19.368786 3.127801

C -1.290084 18.848500 2.427390

C -1.196976 18.598245 1.055530

C -2.370685 17.981787 0.284929

C -2.178886 16.452914 0.320637

C -2.388776 18.471805 -1.175805

C -3.722466 18.351833 0.925397

C 0.019613 18.854223 0.397532

C 1.128458 19.354631 1.084124

C 1.007425 19.628097 2.460945

N 2.130446 12.901558 5.722495

N 0.340265 12.435307 4.423420

N 0.242946 14.588055 5.494372

N 0.899843 17.362770 6.097079

N 1.782584 19.259823 7.474572

N 0.871873 19.683440 5.398181

C 3.803799 14.909554 11.480582

C 5.021260 15.649308 10.918757

C 4.269876 14.050939 12.676134

C 2.752300 15.935728 11.949373

C 1.474922 10.010264 11.096986

C -0.265292 10.510237 9.359491

C -0.328020 11.724089 11.537291

C 1.865273 14.402285 -2.000742

C 3.277657 14.089706 0.053216

C 1.452410 15.832065 0.003822

C 2.477775 19.607894 0.398814

C 2.480906 19.156599 -1.073679

C 3.564095 18.817474 1.147913

C 2.775149 21.119415 0.460677

C 0.065964 9.614453 -1.732880

H 2.296977 16.442905 4.726515

H 1.171928 17.558596 3.932897

H 0.603443 15.195686 3.439839

H -0.778183 15.991085 4.311652

H 2.246397 16.014836 7.126243

H 0.949154 16.736700 8.162939

H 0.444481 14.326639 7.625575

H -0.832742 15.441287 7.051474

H 1.494350 11.050335 3.251882

H 2.391359 10.816048 6.152633

H 1.297942 8.701955 3.262944

H -0.662598 10.956322 6.388912

H -1.856310 8.905236 7.110560

H -1.478270 6.752423 5.918472

H 0.101946 6.657566 3.996812

H 6.610052 9.599309 4.577390

H 4.476244 9.830488 5.818986

H 3.181075 12.852389 3.000544

H 5.382275 12.724611 1.861211

H 7.080403 11.052122 2.613304

H -1.286140 13.620090 3.684853

H -1.551206 11.859166 3.723010

H -1.591417 10.822588 1.693996

H 1.001395 14.249892 2.128188

H 0.632276 12.274688 -1.693537

H -1.273049 8.766478 0.530325

H -2.217687 18.639278 2.957205

H 1.867644 20.027014 3.003495

H 0.091122 18.643010 -0.670948

H -0.911261 20.624715 4.710379

H -1.071120 18.872746 5.040266

H 2.815752 21.161033 7.562918

H 0.640908 21.808923 5.735070

H -0.876207 20.108139 7.702620

H -2.512367 21.237557 9.183166

H -1.804334 23.163694 10.590608

H 0.556116 23.954393 10.516925

H 2.203488 22.827984 9.047430

H 1.949452 23.382552 4.610431

H 3.950362 24.009081 3.292518

H 5.786401 22.366376 2.938612

H 3.639772 19.423122 5.212263

H 5.600006 20.053262 3.923734

H 3.910930 19.096390 7.725786

H 3.025053 17.578133 8.049067

H 5.111468 18.904589 9.702068

H 3.158245 19.686229 13.471646

H 0.816284 19.073339 9.904304

H 4.092540 14.921237 8.703826

H 2.019174 12.921309 11.921036

H 1.336718 11.702027 7.853676

H 3.129467 14.653976 6.424681

H 4.105904 13.172910 6.478095

H 3.714663 21.356131 -0.050135

H 1.982234 21.704337 -0.014204

H 2.878211 21.471301 1.494467

H 3.685035 19.164693 2.186939

H 3.331171 17.740268 1.186803

H 4.545921 18.913228 0.671172

H 2.294850 18.071736 -1.156005

H 1.730444 19.682593 -1.669997

H 3.455686 19.345863 -1.537380

H -1.492460 18.159067 -1.724101

H -3.251834 18.071270 -1.718199

H -2.444791 19.564312 -1.230174

H -3.863505 19.437515 0.958612

H -4.556809 17.930963 0.353553

H -3.811437 17.972606 1.948707

H -1.176550 16.164458 -0.045455

H -2.275234 16.058263 1.336307

H -2.915906 15.941066 -0.305287

H 1.976941 16.618517 -0.562399

H 0.371988 16.004570 -0.109652

H 1.716184 15.998537 1.063933

H 4.010045 14.857514 -0.234827

H 3.308519 14.063393 1.157692

H 3.634771 13.127311 -0.318315

H 2.566123 15.146045 -2.397588

H 2.179232 13.431120 -2.397095

H 0.876557 14.634264 -2.407375

H 0.783410 9.195720 -1.019035

H -0.295052 8.791117 -2.358148

H 0.617938 10.297009 -2.388225

H -2.764483 9.689846 0.278196

H -2.266731 8.476382 -0.895779

H -1.504214 11.719606 -2.655841

H -2.472578 10.247164 -2.714007

H -2.858692 11.512784 -1.544011

H 2.087338 10.390234 11.921935

H 0.858092 9.198113 11.494125

H 2.159845 9.575383 10.360582

H 0.227097 12.093918 12.406756

H -0.904116 12.562752 11.134408

H -1.040559 10.978815 11.905890

H 0.339758 10.003280 8.599057

H -0.950761 9.759824 9.769666

H -0.875072 11.270940 8.862980

H 1.873181 15.454390 12.383559

H 3.171482 16.614347 12.701997

H 2.419261 16.569487 11.118168

H 4.811924 14.659846 13.407258

H 3.432101 13.581637 13.200425

H 4.946574 13.254303 12.349077

H 5.814149 14.962619 10.583325

H 4.775568 16.279696 10.042694

H 5.470051 16.324519 11.665818

H 6.919790 18.413260 10.950352

H 6.771290 20.159173 10.661942

H 7.775733 19.552623 11.979542

H 6.635975 20.910029 13.684409

H 5.551902 21.631091 12.491336

H 4.892019 20.846036 13.929864

H 6.693989 18.270579 13.919615

H 4.940222 18.271224 14.132331

H 5.675875 17.300454 12.842387

H 1.346314 21.358624 13.616115

H 0.390879 21.667907 12.164301

H -0.410792 21.205733 13.671046

H -0.655257 18.082227 11.576380

H -1.581090 19.243108 12.528658

H -0.856676 19.748185 11.002964

H 1.526957 18.975278 14.553420

H -0.213696 18.705857 14.512736

H 0.871244 17.611406 13.645732

S 9.364135 17.568038 6.551805

C 9.936465 19.256003 7.057373

C 9.174811 16.748155 8.165752

H 9.121945 19.648093 7.673070

C 10.031539 20.129390 5.811015

C 11.209019 19.258883 7.894738

H 8.497372 15.913484 7.954482

H 8.673831 17.417362 8.868847

C 10.414547 16.185883 8.843123

C 9.520402 21.433213 5.866298

C 10.641718 19.698221 4.624366

C 11.165303 19.770202 9.198634

C 12.437240 18.794911 7.399240

O 10.594787 16.232151 10.042423

O 11.243084 15.575820 7.980853

C 9.625874 22.291077 4.771020

H 9.038777 21.779731 6.777064

C 10.743095 20.553921 3.525389

H 11.030043 18.687156 4.545164

C 12.316508 19.824531 9.989099

H 10.221932 20.132876 9.599177

C 13.588020 18.847837 8.185680

H 12.495441 18.390025 6.393920

C 12.415673 14.981493 8.563018

C 10.239250 21.853883 3.595017

H 9.224265 23.299629 4.836422

H 11.215685 20.199459 2.612537

C 13.532841 19.364233 9.484696

H 12.259012 20.224346 10.998705

H 14.531966 18.488225 7.781986

H 12.949995 14.520113 7.731876

H 13.033636 15.749121 9.037763

H 12.137814 14.228333 9.305267

H 10.320798 22.519405 2.738632

H 14.431494 19.405166 10.096287

**TSS-01**

Mo 4.518091 15.390405 2.926691

Mo 5.439123 17.265406 6.022419

O 6.029327 13.729719 6.315479

O 8.125745 13.858763 4.928533

O 6.904910 15.903727 5.490584

O 5.914589 14.267619 3.910261

O 5.069350 15.041877 1.364795

O 3.199474 14.013503 3.409364

O 2.560683 15.185461 2.821819

O 4.224376 17.308569 2.875918

O 5.507052 17.054487 3.497244

O 4.085940 15.867817 5.394379

O 3.515248 17.183223 5.622178

O 5.440895 16.948361 7.695492

O 5.214211 19.122484 5.920719

O 6.871410 18.578042 5.671231

S 6.783072 14.363148 5.221396

C 3.135985 13.499010 6.849627

C 2.964848 13.421014 8.343820

C 2.130572 12.485418 8.965726

C 2.030654 12.447634 10.364515

C 1.151555 11.417581 11.083416

C 2.788890 13.350274 11.119435

C 3.636834 14.291641 10.509181

C 3.712007 14.327269 9.112910

C 2.505547 11.191024 5.845341

C 3.882584 10.915534 5.300927

C 4.749837 10.100197 6.046857

C 6.020403 9.797383 5.552251

C 6.437830 10.315185 4.320737

C 5.579537 11.135621 3.582940

C 4.300661 11.427809 4.066705

C 1.369995 10.749770 4.857096

C 0.596083 9.565240 5.387090

C 0.740348 8.324072 4.750733

C 0.057749 7.206289 5.239517

C -0.772045 7.323052 6.358875

C -0.920221 8.562115 6.991147

C -0.238567 9.682007 6.508499

C -0.627163 12.000212 3.807052

C -0.201919 11.691884 2.387421

C -0.975940 10.769380 1.663128

C -0.650058 10.466182 0.339116

C -1.459498 9.459264 -0.486430

C -0.627908 8.165638 -0.594834

C -2.818139 9.132836 0.161998

C 0.478303 11.077469 -0.238775

C 1.261704 11.991431 0.471236

C 2.499386 12.660313 -0.143041

C 0.902212 12.305308 1.797964

C 1.039880 13.037532 5.473294

C 0.230537 14.874832 6.982807

C 1.060652 16.168287 7.092219

C 0.994533 16.396768 4.655150

C 0.133014 15.142394 4.498141

C 1.076739 18.429733 6.014267

C 2.830582 18.577939 7.903429

C 2.549959 18.925270 9.344768

C 3.647108 19.260227 10.149831

C 3.461920 19.556957 11.505803

C 4.638961 19.927121 12.416589

C 4.489987 19.212733 13.775231

C 4.615712 21.455324 12.616242

C 5.989819 19.512410 11.806900

C 2.159246 19.536322 12.027384

C 1.053226 19.196012 11.233085

C -0.338782 19.176950 11.874748

C -0.340011 18.131531 13.008614

C -0.639905 20.578677 12.442398

C -1.448017 18.811003 10.869804

C 1.259312 18.880995 9.883360

C 1.897920 20.581676 6.634323

C 1.198806 21.435276 7.664695

C 1.931371 22.422449 8.339115

C 1.297025 23.245807 9.274285

C -0.065937 23.086104 9.541265

C -0.796031 22.096262 8.874306

C -0.167442 21.270567 7.939154

C 1.230415 20.680970 5.221756

C 2.244314 20.921980 4.126346

C 2.140579 22.082303 3.342409

C 3.076495 22.326960 2.335087

C 4.117982 21.419503 2.102632

C 4.219227 20.263660 2.879493

C 3.281477 20.011576 3.887194

C -0.669890 19.181793 4.298204

C -0.469115 19.559144 2.848228

C -1.247862 20.582005 2.293945

C -1.095959 20.922701 0.942848

C -1.916890 22.043553 0.294773

C -2.430373 21.583879 -1.084448

C -0.991646 23.265788 0.132863

C -3.135561 22.440935 1.149225

C -0.136824 20.241905 0.177994

C 0.660269 19.224345 0.725943

C 0.470146 18.875543 2.069087

N 2.216832 12.659082 6.063588

N 0.500537 11.972815 4.765156

N 0.249903 14.201107 5.653441

N 0.677085 17.075576 5.963598

N 1.795545 19.110243 6.980436

N 0.595370 19.324451 5.064259

C 4.424452 15.261114 11.400331

C 5.433114 16.100527 10.606324

C 5.195390 14.454218 12.466937

C 3.405609 16.195783 12.081861

C 2.082736 10.335717 11.665087

C 0.134994 10.751326 10.135555

C 0.364887 12.102152 12.219787

C 2.757141 12.205961 -1.593566

C 3.728942 12.296837 0.708721

C 2.288743 14.184480 -0.136397

C 1.688102 18.509544 -0.160563

C 0.914034 17.595341 -1.131682

C 2.672582 17.665008 0.661277

C 2.498011 19.557989 -0.950166

C -1.726300 10.034935 -1.891701

H 2.095743 16.118063 4.609435

H 0.848593 17.076003 3.780037

H 0.444375 14.640609 3.542649

H -0.948623 15.404238 4.422103

H 2.166229 15.935695 7.067741

H 0.839940 16.656859 8.069521

H 0.538317 14.171567 7.791916

H -0.850238 15.111502 7.161691

H 1.787278 10.522375 3.835765

H 2.380258 10.679406 6.842898

H 1.385092 8.223625 3.875947

H -0.363166 10.645182 7.002697

H -1.566941 8.654668 7.863801

H -1.302066 6.449628 6.738795

H 0.174732 6.241308 4.745179

H 6.693397 9.168520 6.134078

H 4.442251 9.708539 7.016104

H 3.636359 12.071116 3.469786

H 5.909892 11.572554 2.637409

H 7.436964 10.093480 3.945493

H -1.150363 12.984085 3.843936

H -1.373790 11.245360 4.168365

H -1.827623 10.292107 2.144880

H 1.510017 13.032984 2.357502

H 0.736671 10.821361 -1.267507

H 0.336983 8.343711 -1.081723

H -1.965989 21.110517 2.917575

H 1.068309 18.080493 2.529864

H -0.001452 20.513572 -0.871177

H -1.451641 19.821963 4.777399

H -1.050921 18.133955 4.371092

H 2.991543 20.852845 6.564098

H 0.434408 21.470104 5.204890

H -0.738533 20.495719 7.430900

H -1.857308 21.968551 9.085833

H -0.558201 23.730431 10.269150

H 1.870009 24.011153 9.798173

H 2.997315 22.549431 8.142946

H 1.331887 22.790959 3.510413

H 2.993675 23.225090 1.725628

H 4.846417 21.613458 1.316176

H 3.387659 19.085588 4.472807

H 5.020250 19.529126 2.715943

H 3.837806 19.000330 7.589910

H 2.956861 17.467173 7.793801

H 4.646912 19.274519 9.708416

H 2.003081 19.788100 13.077209

H 0.422306 18.624503 9.238599

H 4.375544 15.047046 8.607407

H 2.729326 13.319105 12.208295

H 1.573163 11.768983 8.368557

H 3.083314 14.568819 6.480319

H 4.208621 13.201804 6.580981

H 3.285166 19.080599 -1.544298

H 1.873677 20.132893 -1.640077

H 2.988944 20.266744 -0.271972

H 3.232325 18.289014 1.380116

H 2.178616 16.861836 1.225006

H 3.424783 17.178810 0.025544

H 0.328958 16.844678 -0.590195

H 0.224671 18.161657 -1.764713

H 1.601575 17.056324 -1.793052

H -0.128805 23.038294 -0.502740

H -1.520468 24.108566 -0.323638

H -0.602321 23.603248 1.099169

H -2.839802 22.838267 2.125714

H -3.724563 23.220814 0.653305

H -3.803053 21.589442 1.318299

H -1.609686 21.378308 -1.780609

H -3.028216 20.670082 -1.002549

H -3.060309 22.349761 -1.549051

H 3.201806 14.707489 -0.454869

H 1.469812 14.491608 -0.788518

H 2.079333 14.558296 0.881154

H 4.621028 12.843240 0.366383

H 3.595250 12.597825 1.764080

H 3.948227 11.228243 0.680677

H 3.647604 12.703617 -1.996501

H 2.936457 11.128553 -1.658527

H 1.922512 12.457050 -2.254863

H -0.798433 10.200332 -2.450501

H -2.343947 9.355719 -2.488530

H -2.250593 10.994776 -1.835758

H -0.421169 7.739779 0.392588

H -1.152412 7.403285 -1.179894

H -3.431145 10.031787 0.285644

H -3.390350 8.433027 -0.457402

H -2.701189 8.665610 1.145549

H 2.801989 10.760103 12.374210

H 1.517144 9.562889 12.194716

H 2.661286 9.841006 10.877127

H 1.030833 12.517493 12.984577

H -0.251165 12.923168 11.840016

H -0.300307 11.394077 12.724787

H 0.630524 10.178949 9.343711

H -0.504738 10.048104 10.680869

H -0.520747 11.490338 9.665423

H 2.670659 15.646417 12.674658

H 3.908703 16.909334 12.755578

H 2.861529 16.795328 11.341379

H 5.827214 15.109980 13.074352

H 4.525163 13.922030 13.147957

H 5.850637 13.710675 11.999163

H 6.256287 15.494331 10.211392

H 4.980878 16.597067 9.727742

H 5.872116 16.900138 11.221924

H 6.021661 18.426579 11.601936

H 6.190263 20.029884 10.861948

H 6.819231 19.741434 12.483036

H 5.431581 21.782607 13.268639

H 4.726127 21.981764 11.661873

H 3.676845 21.790359 13.068466

H 5.367956 19.372164 14.408632

H 3.617902 19.565548 14.334199

H 4.375547 18.124130 13.631284

H 0.069782 20.864027 13.225790

H -0.583122 21.342119 11.657424

H -1.641807 20.625041 12.880206

H -1.303598 17.809232 10.453273

H -2.431686 18.820297 11.352111

H -1.488174 19.524242 10.038938

H 0.374940 18.385190 13.798404

H -1.326154 18.053038 13.477061

H -0.070392 17.138424 12.633716

S 8.901603 17.897979 4.996850

C 9.974628 16.888335 6.149083

C 9.615259 19.577820 5.131494

H 9.392146 15.960219 6.137010

C 10.060175 17.372432 7.597000

C 11.296089 16.615666 5.450793

H 10.614830 19.585611 4.679348

H 9.683799 19.889410 6.176266

C 8.724807 20.527838 4.346385

C 11.115165 16.928624 8.411597

C 9.059919 18.164850 8.180226

C 11.420640 15.462700 4.659728

C 12.385242 17.498581 5.530276

O 8.534080 20.456870 3.150819

O 8.177932 21.459815 5.144591

C 11.177505 17.277055 9.761958

H 11.894650 16.301039 7.989254

C 9.126907 18.517695 9.530632

H 8.221817 18.502581 7.582997

C 12.602411 15.201541 3.964685

H 10.581197 14.776268 4.586926

C 13.565879 17.238231 4.831683

H 12.318603 18.382339 6.159485

C 7.244507 22.346770 4.499532

C 10.184747 18.079692 10.327869

H 12.006240 16.918990 10.370129

H 8.341734 19.136852 9.956667

C 13.678811 16.088788 4.045403

H 12.681590 14.301722 3.359342

H 14.400149 17.932938 4.906297

H 6.940436 23.056737 5.269316

H 7.723110 22.868515 3.665347

H 6.383680 21.781378 4.134988

H 10.235434 18.356990 11.378907

H 14.600118 15.884856 3.503594

**TSS-02**

Mo 4.520336 15.396026 2.932229

Mo 5.453262 17.254487 6.028809

O 6.017557 13.749062 6.322277

O 8.116531 13.832371 4.938942

O 6.926856 15.904442 5.473496

O 5.916034 14.264116 3.910336

O 5.075229 15.051463 1.370166

O 3.199182 14.016910 3.402824

O 2.563352 15.193319 2.821336

O 4.231097 17.314904 2.883343

O 5.514950 17.060587 3.502177

O 4.098351 15.856351 5.382096

O 3.528477 17.168884 5.623543

O 5.439516 16.902804 7.698247

O 5.217376 19.099049 5.941767

O 6.912188 18.561166 5.745235

S 6.781672 14.360246 5.221918

C 3.135985 13.499010 6.849627

C 2.964848 13.421014 8.343820

C 2.130572 12.485418 8.965726

C 2.030654 12.447634 10.364515

C 1.151555 11.417581 11.083416

C 2.788890 13.350274 11.119435

C 3.636834 14.291641 10.509181

C 3.712007 14.327269 9.112910

C 2.505547 11.191024 5.845341

C 3.882584 10.915534 5.300927

C 4.749837 10.100197 6.046857

C 6.020403 9.797383 5.552251

C 6.437830 10.315185 4.320737

C 5.579537 11.135621 3.582940

C 4.300661 11.427809 4.066705

C 1.369995 10.749770 4.857096

C 0.596083 9.565240 5.387090

C 0.740348 8.324072 4.750733

C 0.057749 7.206289 5.239517

C -0.772045 7.323052 6.358875

C -0.920221 8.562115 6.991147

C -0.238567 9.682007 6.508499

C -0.627163 12.000212 3.807052

C -0.201919 11.691884 2.387421

C -0.975940 10.769380 1.663128

C -0.650058 10.466182 0.339116

C -1.459498 9.459264 -0.486430

C -0.627908 8.165638 -0.594834

C -2.818139 9.132836 0.161998

C 0.478303 11.077469 -0.238775

C 1.261704 11.991431 0.471236

C 2.499386 12.660313 -0.143041

C 0.902212 12.305308 1.797964

C 1.039880 13.037532 5.473294

C 0.230537 14.874832 6.982807

C 1.060652 16.168287 7.092219

C 0.994533 16.396768 4.655150

C 0.133014 15.142394 4.498141

C 1.076739 18.429733 6.014267

C 2.830582 18.577939 7.903429

C 2.549959 18.925270 9.344768

C 3.647108 19.260227 10.149831

C 3.461920 19.556957 11.505803

C 4.638961 19.927121 12.416589

C 4.489987 19.212733 13.775231

C 4.615712 21.455324 12.616242

C 5.989819 19.512410 11.806900

C 2.159246 19.536322 12.027384

C 1.053226 19.196012 11.233085

C -0.338782 19.176950 11.874748

C -0.340011 18.131531 13.008614

C -0.639905 20.578677 12.442398

C -1.448017 18.811003 10.869804

C 1.259312 18.880995 9.883360

C 1.897920 20.581676 6.634323

C 1.198806 21.435276 7.664695

C 1.931371 22.422449 8.339115

C 1.297025 23.245807 9.274285

C -0.065937 23.086104 9.541265

C -0.796031 22.096262 8.874306

C -0.167442 21.270567 7.939154

C 1.230415 20.680970 5.221756

C 2.244314 20.921980 4.126346

C 2.140579 22.082303 3.342409

C 3.076495 22.326960 2.335087

C 4.117982 21.419503 2.102632

C 4.219227 20.263660 2.879493

C 3.281477 20.011576 3.887194

C -0.669890 19.181793 4.298204

C -0.469115 19.559144 2.848228

C -1.247862 20.582005 2.293945

C -1.095959 20.922701 0.942848

C -1.916890 22.043553 0.294773

C -2.430373 21.583879 -1.084448

C -0.991646 23.265788 0.132863

C -3.135561 22.440935 1.149225

C -0.136824 20.241905 0.177994

C 0.660269 19.224345 0.725943

C 0.470146 18.875543 2.069087

N 2.216832 12.659082 6.063588

N 0.500537 11.972815 4.765156

N 0.249903 14.201107 5.653441

N 0.677085 17.075576 5.963598

N 1.795545 19.110243 6.980436

N 0.595370 19.324451 5.064259

C 4.424452 15.261114 11.400331

C 5.433114 16.100527 10.606324

C 5.195390 14.454218 12.466937

C 3.405609 16.195783 12.081861

C 2.082736 10.335717 11.665087

C 0.134994 10.751326 10.135555

C 0.364887 12.102152 12.219787

C 2.757141 12.205961 -1.593566

C 3.728942 12.296837 0.708721

C 2.288743 14.184480 -0.136397

C 1.688102 18.509544 -0.160563

C 0.914034 17.595341 -1.131682

C 2.672582 17.665008 0.661277

C 2.498011 19.557989 -0.950166

C -1.726300 10.034935 -1.891701

H 2.095743 16.118063 4.609435

H 0.848593 17.076003 3.780037

H 0.444375 14.640609 3.542649

H -0.948623 15.404238 4.422103

H 2.166229 15.935695 7.067741

H 0.839940 16.656859 8.069521

H 0.538317 14.171567 7.791916

H -0.850238 15.111502 7.161691

H 1.787278 10.522375 3.835765

H 2.380258 10.679406 6.842898

H 1.385092 8.223625 3.875947

H -0.363166 10.645182 7.002697

H -1.566941 8.654668 7.863801

H -1.302066 6.449628 6.738795

H 0.174732 6.241308 4.745179

H 6.693397 9.168520 6.134078

H 4.442251 9.708539 7.016104

H 3.636359 12.071116 3.469786

H 5.909892 11.572554 2.637409

H 7.436964 10.093480 3.945493

H -1.150363 12.984085 3.843936

H -1.373790 11.245360 4.168365

H -1.827623 10.292107 2.144880

H 1.510017 13.032984 2.357502

H 0.736671 10.821361 -1.267507

H 0.336983 8.343711 -1.081723

H -1.965989 21.110517 2.917575

H 1.068309 18.080493 2.529864

H -0.001452 20.513572 -0.871177

H -1.451641 19.821963 4.777399

H -1.050921 18.133955 4.371092

H 2.991543 20.852845 6.564098

H 0.434408 21.470104 5.204890

H -0.738533 20.495719 7.430900

H -1.857308 21.968551 9.085833

H -0.558201 23.730431 10.269150

H 1.870009 24.011153 9.798173

H 2.997315 22.549431 8.142946

H 1.331887 22.790959 3.510413

H 2.993675 23.225090 1.725628

H 4.846417 21.613458 1.316176

H 3.387659 19.085588 4.472807

H 5.020250 19.529126 2.715943

H 3.837806 19.000330 7.589910

H 2.956861 17.467173 7.793801

H 4.646912 19.274519 9.708416

H 2.003081 19.788100 13.077209

H 0.422306 18.624503 9.238599

H 4.375544 15.047046 8.607407

H 2.729326 13.319105 12.208295

H 1.573163 11.768983 8.368557

H 3.083314 14.568819 6.480319

H 4.208621 13.201804 6.580981

H 3.285166 19.080599 -1.544298

H 1.873677 20.132893 -1.640077

H 2.988944 20.266744 -0.271972

H 3.232325 18.289014 1.380116

H 2.178616 16.861836 1.225006

H 3.424783 17.178810 0.025544

H 0.328958 16.844678 -0.590195

H 0.224671 18.161657 -1.764713

H 1.601575 17.056324 -1.793052

H -0.128805 23.038294 -0.502740

H -1.520468 24.108566 -0.323638

H -0.602321 23.603248 1.099169

H -2.839802 22.838267 2.125714

H -3.724563 23.220814 0.653305

H -3.803053 21.589442 1.318299

H -1.609686 21.378308 -1.780609

H -3.028216 20.670082 -1.002549

H -3.060309 22.349761 -1.549051

H 3.201806 14.707489 -0.454869

H 1.469812 14.491608 -0.788518

H 2.079333 14.558296 0.881154

H 4.621028 12.843240 0.366383

H 3.595250 12.597825 1.764080

H 3.948227 11.228243 0.680677

H 3.647604 12.703617 -1.996501

H 2.936457 11.128553 -1.658527

H 1.922512 12.457050 -2.254863

H -0.798433 10.200332 -2.450501

H -2.343947 9.355719 -2.488530

H -2.250593 10.994776 -1.835758

H -0.421169 7.739779 0.392588

H -1.152412 7.403285 -1.179894

H -3.431145 10.031787 0.285644

H -3.390350 8.433027 -0.457402

H -2.701189 8.665610 1.145549

H 2.801989 10.760103 12.374210

H 1.517144 9.562889 12.194716

H 2.661286 9.841006 10.877127

H 1.030833 12.517493 12.984577

H -0.251165 12.923168 11.840016

H -0.300307 11.394077 12.724787

H 0.630524 10.178949 9.343711

H -0.504738 10.048104 10.680869

H -0.520747 11.490338 9.665423

H 2.670659 15.646417 12.674658

H 3.908703 16.909334 12.755578

H 2.861529 16.795328 11.341379

H 5.827214 15.109980 13.074352

H 4.525163 13.922030 13.147957

H 5.850637 13.710675 11.999163

H 6.256287 15.494331 10.211392

H 4.980878 16.597067 9.727742

H 5.872116 16.900138 11.221924

H 6.021661 18.426579 11.601936

H 6.190263 20.029884 10.861948

H 6.819231 19.741434 12.483036

H 5.431581 21.782607 13.268639

H 4.726127 21.981764 11.661873

H 3.676845 21.790359 13.068466

H 5.367956 19.372164 14.408632

H 3.617902 19.565548 14.334199

H 4.375547 18.124130 13.631284

H 0.069782 20.864027 13.225790

H -0.583122 21.342119 11.657424

H -1.641807 20.625041 12.880206

H -1.303598 17.809232 10.453273

H -2.431686 18.820297 11.352111

H -1.488174 19.524242 10.038938

H 0.374940 18.385190 13.798404

H -1.326154 18.053038 13.477061

H -0.070392 17.138424 12.633716

S 9.036849 18.128442 5.257945

C 9.904926 16.825021 6.323621

C 9.490070 19.753052 5.977533

H 9.151906 16.036895 6.250935

C 10.019616 17.229445 7.783964

C 11.172217 16.305425 5.665117

H 10.057022 19.607056 6.900374

H 8.542440 20.248446 6.199261

C 10.263653 20.604030 4.991129

C 11.225694 17.648301 8.364637

C 8.869441 17.177843 8.591232

C 11.286936 14.923068 5.455571

C 12.232669 17.134783 5.268461

O 9.764718 21.253991 4.097377

O 11.593926 20.552928 5.223305

C 11.281523 18.014345 9.713108

H 12.134028 17.670386 7.770141

C 8.927512 17.542430 9.935657

H 7.926327 16.849394 8.170387

C 12.443195 14.380634 4.889401

H 10.456303 14.274300 5.714512

C 13.388646 16.591766 4.705197

H 12.153767 18.211575 5.383959

C 12.409371 21.307715 4.306706

C 10.133028 17.964964 10.503401

H 12.229594 18.331631 10.143537

H 8.027162 17.489071 10.542022

C 13.501206 15.211439 4.517262

H 12.510310 13.306697 4.731802

H 14.201111 17.250846 4.405363

H 13.436158 21.164745 4.646134

H 12.285731 20.934472 3.287043

H 12.139818 22.366856 4.338565

H 10.177717 18.245855 11.553721

H 14.401405 14.790337 4.074064

**TSS-03**

Mo 4.524831 15.393409 2.931821

Mo 5.447417 17.257512 6.036023

O 6.028320 13.724531 6.316182

O 8.126939 13.844523 4.933079

O 6.913782 15.894762 5.490635

O 5.920722 14.261866 3.909460

O 5.079766 15.045063 1.370136

O 3.200353 14.017964 3.403788

O 2.567566 15.195232 2.820536

O 4.242385 17.313303 2.876788

O 5.523769 17.053813 3.500073

O 4.096838 15.859576 5.383044

O 3.524183 17.172563 5.617163

O 5.420872 16.919710 7.705373

O 5.217170 19.101050 5.929575

O 6.917712 18.559254 5.752262

S 6.785201 14.355097 5.222863

C 3.135985 13.499010 6.849627

C 2.964848 13.421014 8.343820

C 2.130572 12.485418 8.965726

C 2.030654 12.447634 10.364515

C 1.151555 11.417581 11.083416

C 2.788890 13.350274 11.119435

C 3.636834 14.291641 10.509181

C 3.712007 14.327269 9.112910

C 2.505547 11.191024 5.845341

C 3.882584 10.915534 5.300927

C 4.749837 10.100197 6.046857

C 6.020403 9.797383 5.552251

C 6.437830 10.315185 4.320737

C 5.579537 11.135621 3.582940

C 4.300661 11.427809 4.066705

C 1.369995 10.749770 4.857096

C 0.596083 9.565240 5.387090

C 0.740348 8.324072 4.750733

C 0.057749 7.206289 5.239517

C -0.772045 7.323052 6.358875

C -0.920221 8.562115 6.991147

C -0.238567 9.682007 6.508499

C -0.627163 12.000212 3.807052

C -0.201919 11.691884 2.387421

C -0.975940 10.769380 1.663128

C -0.650058 10.466182 0.339116

C -1.459498 9.459264 -0.486430

C -0.627908 8.165638 -0.594834

C -2.818139 9.132836 0.161998

C 0.478303 11.077469 -0.238775

C 1.261704 11.991431 0.471236

C 2.499386 12.660313 -0.143041

C 0.902212 12.305308 1.797964

C 1.039880 13.037532 5.473294

C 0.230537 14.874832 6.982807

C 1.060652 16.168287 7.092219

C 0.994533 16.396768 4.655150

C 0.133014 15.142394 4.498141

C 1.076739 18.429733 6.014267

C 2.830582 18.577939 7.903429

C 2.549959 18.925270 9.344768

C 3.647108 19.260227 10.149831

C 3.461920 19.556957 11.505803

C 4.638961 19.927121 12.416589

C 4.489987 19.212733 13.775231

C 4.615712 21.455324 12.616242

C 5.989819 19.512410 11.806900

C 2.159246 19.536322 12.027384

C 1.053226 19.196012 11.233085

C -0.338782 19.176950 11.874748

C -0.340011 18.131531 13.008614

C -0.639905 20.578677 12.442398

C -1.448017 18.811003 10.869804

C 1.259312 18.880995 9.883360

C 1.897920 20.581676 6.634323

C 1.198806 21.435276 7.664695

C 1.931371 22.422449 8.339115

C 1.297025 23.245807 9.274285

C -0.065937 23.086104 9.541265

C -0.796031 22.096262 8.874306

C -0.167442 21.270567 7.939154

C 1.230415 20.680970 5.221756

C 2.244314 20.921980 4.126346

C 2.140579 22.082303 3.342409

C 3.076495 22.326960 2.335087

C 4.117982 21.419503 2.102632

C 4.219227 20.263660 2.879493

C 3.281477 20.011576 3.887194

C -0.669890 19.181793 4.298204

C -0.469115 19.559144 2.848228

C -1.247862 20.582005 2.293945

C -1.095959 20.922701 0.942848

C -1.916890 22.043553 0.294773

C -2.430373 21.583879 -1.084448

C -0.991646 23.265788 0.132863

C -3.135561 22.440935 1.149225

C -0.136824 20.241905 0.177994

C 0.660269 19.224345 0.725943

C 0.470146 18.875543 2.069087

N 2.216832 12.659082 6.063588

N 0.500537 11.972815 4.765156

N 0.249903 14.201107 5.653441

N 0.677085 17.075576 5.963598

N 1.795545 19.110243 6.980436

N 0.595370 19.324451 5.064259

C 4.424452 15.261114 11.400331

C 5.433114 16.100527 10.606324

C 5.195390 14.454218 12.466937

C 3.405609 16.195783 12.081861

C 2.082736 10.335717 11.665087

C 0.134994 10.751326 10.135555

C 0.364887 12.102152 12.219787

C 2.757141 12.205961 -1.593566

C 3.728942 12.296837 0.708721

C 2.288743 14.184480 -0.136397

C 1.688102 18.509544 -0.160563

C 0.914034 17.595341 -1.131682

C 2.672582 17.665008 0.661277

C 2.498011 19.557989 -0.950166

C -1.726300 10.034935 -1.891701

H 2.095743 16.118063 4.609435

H 0.848593 17.076003 3.780037

H 0.444375 14.640609 3.542649

H -0.948623 15.404238 4.422103

H 2.166229 15.935695 7.067741

H 0.839940 16.656859 8.069521

H 0.538317 14.171567 7.791916

H -0.850238 15.111502 7.161691

H 1.787278 10.522375 3.835765

H 2.380258 10.679406 6.842898

H 1.385092 8.223625 3.875947

H -0.363166 10.645182 7.002697

H -1.566941 8.654668 7.863801

H -1.302066 6.449628 6.738795

H 0.174732 6.241308 4.745179

H 6.693397 9.168520 6.134078

H 4.442251 9.708539 7.016104

H 3.636359 12.071116 3.469786

H 5.909892 11.572554 2.637409

H 7.436964 10.093480 3.945493

H -1.150363 12.984085 3.843936

H -1.373790 11.245360 4.168365

H -1.827623 10.292107 2.144880

H 1.510017 13.032984 2.357502

H 0.736671 10.821361 -1.267507

H 0.336983 8.343711 -1.081723

H -1.965989 21.110517 2.917575

H 1.068309 18.080493 2.529864

H -0.001452 20.513572 -0.871177

H -1.451641 19.821963 4.777399

H -1.050921 18.133955 4.371092

H 2.991543 20.852845 6.564098

H 0.434408 21.470104 5.204890

H -0.738533 20.495719 7.430900

H -1.857308 21.968551 9.085833

H -0.558201 23.730431 10.269150

H 1.870009 24.011153 9.798173

H 2.997315 22.549431 8.142946

H 1.331887 22.790959 3.510413

H 2.993675 23.225090 1.725628

H 4.846417 21.613458 1.316176

H 3.387659 19.085588 4.472807

H 5.020250 19.529126 2.715943

H 3.837806 19.000330 7.589910

H 2.956861 17.467173 7.793801

H 4.646912 19.274519 9.708416

H 2.003081 19.788100 13.077209

H 0.422306 18.624503 9.238599

H 4.375544 15.047046 8.607407

H 2.729326 13.319105 12.208295

H 1.573163 11.768983 8.368557

H 3.083314 14.568819 6.480319

H 4.208621 13.201804 6.580981

H 3.285166 19.080599 -1.544298

H 1.873677 20.132893 -1.640077

H 2.988944 20.266744 -0.271972

H 3.232325 18.289014 1.380116

H 2.178616 16.861836 1.225006

H 3.424783 17.178810 0.025544

H 0.328958 16.844678 -0.590195

H 0.224671 18.161657 -1.764713

H 1.601575 17.056324 -1.793052

H -0.128805 23.038294 -0.502740

H -1.520468 24.108566 -0.323638

H -0.602321 23.603248 1.099169

H -2.839802 22.838267 2.125714

H -3.724563 23.220814 0.653305

H -3.803053 21.589442 1.318299

H -1.609686 21.378308 -1.780609

H -3.028216 20.670082 -1.002549

H -3.060309 22.349761 -1.549051

H 3.201806 14.707489 -0.454869

H 1.469812 14.491608 -0.788518

H 2.079333 14.558296 0.881154

H 4.621028 12.843240 0.366383

H 3.595250 12.597825 1.764080

H 3.948227 11.228243 0.680677

H 3.647604 12.703617 -1.996501

H 2.936457 11.128553 -1.658527

H 1.922512 12.457050 -2.254863

H -0.798433 10.200332 -2.450501

H -2.343947 9.355719 -2.488530

H -2.250593 10.994776 -1.835758

H -0.421169 7.739779 0.392588

H -1.152412 7.403285 -1.179894

H -3.431145 10.031787 0.285644

H -3.390350 8.433027 -0.457402

H -2.701189 8.665610 1.145549

H 2.801989 10.760103 12.374210

H 1.517144 9.562889 12.194716

H 2.661286 9.841006 10.877127

H 1.030833 12.517493 12.984577

H -0.251165 12.923168 11.840016

H -0.300307 11.394077 12.724787

H 0.630524 10.178949 9.343711

H -0.504738 10.048104 10.680869

H -0.520747 11.490338 9.665423

H 2.670659 15.646417 12.674658

H 3.908703 16.909334 12.755578

H 2.861529 16.795328 11.341379

H 5.827214 15.109980 13.074352

H 4.525163 13.922030 13.147957

H 5.850637 13.710675 11.999163

H 6.256287 15.494331 10.211392

H 4.980878 16.597067 9.727742

H 5.872116 16.900138 11.221924

H 6.021661 18.426579 11.601936

H 6.190263 20.029884 10.861948

H 6.819231 19.741434 12.483036

H 5.431581 21.782607 13.268639

H 4.726127 21.981764 11.661873

H 3.676845 21.790359 13.068466

H 5.367956 19.372164 14.408632

H 3.617902 19.565548 14.334199

H 4.375547 18.124130 13.631284

H 0.069782 20.864027 13.225790

H -0.583122 21.342119 11.657424

H -1.641807 20.625041 12.880206

H -1.303598 17.809232 10.453273

H -2.431686 18.820297 11.352111

H -1.488174 19.524242 10.038938

H 0.374940 18.385190 13.798404

H -1.326154 18.053038 13.477061

H -0.070392 17.138424 12.633716

S 8.945475 17.887068 5.197100

C 9.954757 16.724153 6.274162

C 9.809617 19.501899 5.232093

H 9.330202 15.833904 6.143755

C 9.999398 17.038434 7.769231

C 11.281828 16.454255 5.588584

H 9.245144 20.101866 4.512079

H 10.828190 19.365079 4.855164

C 9.837134 20.269336 6.545261

C 11.024701 16.498690 8.562262

C 8.970673 17.748311 8.405587

C 11.384192 15.349158 4.728097

C 12.402100 17.285381 5.756890

O 8.918675 20.914792 7.001547

O 11.048392 20.173767 7.132232

C 11.032421 16.677520 9.947082

H 11.823260 15.928905 8.096327

C 8.981737 17.930726 9.791026

H 8.160900 18.167366 7.822431

C 12.575450 15.083101 4.051275

H 10.522135 14.701526 4.592390

C 13.592217 17.017883 5.076472

H 12.345032 18.137293 6.428075

C 11.182611 20.858431 8.392073

C 10.011374 17.398719 10.569276

H 11.840060 16.249417 10.538201

H 8.175254 18.489820 10.258097

C 13.683787 15.916934 4.221118

H 12.637676 14.220328 3.392276

H 14.451222 17.670911 5.218587

H 12.225720 20.726726 8.683343

H 10.947111 21.919865 8.277443

H 10.517958 20.412442 9.135358

H 10.017613 17.540836 11.648169

H 14.612963 15.708838 3.694298

**TSS-04**

Mo 4.216513 15.305363 2.608884

Mo 5.400333 17.147454 5.653225

O 6.045066 13.652746 5.903725

O 7.944581 13.748591 4.251257

O 6.804396 15.812307 4.932913

O 5.621545 14.140809 3.525278

O 4.674750 14.934365 1.020011

O 2.884044 13.968802 3.167786

O 2.251174 15.156283 2.604657

O 3.976083 17.230207 2.562740

O 5.284478 16.940390 3.106336

O 3.985563 15.767551 5.079950

O 3.453766 17.084713 5.370578

O 5.473691 16.777303 7.314560

O 5.163405 18.988910 5.571850

O 6.860998 18.471842 5.330010

S 6.655595 14.260910 4.710763

C 3.157641 13.474896 6.684155

C 3.063451 13.406201 8.186142

C 2.273891 12.464866 8.856756

C 2.243291 12.438613 10.258882

C 1.414788 11.405473 11.031531

C 3.023631 13.359590 10.968305

C 3.826346 14.306819 10.309028

C 3.833844 14.329016 8.910367

C 2.463207 11.165246 5.733329

C 3.778466 10.877545 5.058012

C 4.703590 10.048976 5.714303

C 5.910032 9.721362 5.091767

C 6.205911 10.226433 3.820413

C 5.292439 11.063267 3.172807

C 4.076551 11.381092 3.785849

C 1.240991 10.704436 4.864636

C 0.531679 9.521329 5.480774

C 0.677119 8.261835 4.881104

C 0.057820 7.144099 5.447598

C -0.709912 7.278535 6.608649

C -0.859257 8.535283 7.204311

C -0.240590 9.655668 6.643723

C -0.844085 11.933080 3.977297

C -0.538801 11.578962 2.537998

C -1.324112 10.587516 1.926041

C -1.100263 10.239010 0.591862

C -1.920873 9.152269 -0.112531

C -1.028172 7.902235 -0.244401

C -3.193528 8.782270 0.672479

C -0.063499 10.879766 -0.112014

C 0.729159 11.863727 0.485196

C 1.872136 12.563096 -0.263575

C 0.475152 12.217489 1.825950

C 0.965007 13.003309 5.466810

C 0.278597 14.883824 6.987860

C 1.119224 16.174705 6.996661

C 0.862713 16.336182 4.566204

C -0.014515 15.084517 4.514530

C 1.098530 18.401823 5.850627

C 2.933408 18.580717 7.660712

C 2.678299 18.951237 9.100941

C 3.788397 19.294177 9.884278

C 3.624698 19.609436 11.239016

C 4.817857 19.984306 12.126608

C 4.682381 19.293702 13.499024

C 4.810096 21.515650 12.300847

C 6.156031 19.547029 11.505308

C 2.329880 19.603831 11.780028

C 1.210229 19.259789 11.006666

C -0.173942 19.268622 11.665152

C -0.171919 18.253719 12.826179

C -0.454652 20.688100 12.198286

C -1.298432 18.886813 10.683249

C 1.395636 18.921646 9.659705

C 1.997582 20.554436 6.346778

C 1.373077 21.456542 7.383683

C 2.164708 22.437909 7.996818

C 1.598898 23.304374 8.937124

C 0.245697 23.193762 9.269914

C -0.543684 22.210163 8.663835

C 0.016767 21.340767 7.725014

C 1.258486 20.621944 4.968622

C 2.217546 20.825045 3.817601

C 2.075347 21.961531 3.004841

C 2.973613 22.184606 1.959430

C 4.019590 21.283407 1.721706

C 4.159127 20.151062 2.526425

C 3.250137 19.913839 3.564162

C -0.729078 19.146403 4.225655

C -0.642826 19.484286 2.754403

C -1.519018 20.441245 2.227474

C -1.485383 20.742441 0.859398

C -2.413113 21.792998 0.238389

C -3.002850 21.253393 -1.080168

C -1.571694 23.054952 -0.037351

C -3.583908 22.159730 1.169976

C -0.547043 20.088215 0.047282

C 0.345575 19.136742 0.566064

C 0.278956 18.828639 1.931329

N 2.193526 12.637191 5.950784

N 0.361059 11.922527 4.837693

N 0.192971 14.173038 5.681270

N 0.661322 17.057758 5.875552

N 1.882436 19.098591 6.748600

N 0.600555 19.269319 4.878765

C 4.633728 15.304818 11.149426

C 5.624075 16.116945 10.304305

C 5.430218 14.538492 12.227082

C 3.626421 16.259195 11.820432

C 2.390020 10.354718 11.597797

C 0.376338 10.698859 10.138609

C 0.659130 12.098392 12.183901

C 1.994735 12.093185 -1.726678

C 3.191433 12.253771 0.466067

C 1.610471 14.079644 -0.257421

C 1.347099 18.456978 -0.376370

C 0.552640 17.562853 -1.350156

C 2.367939 17.595687 0.380425

C 2.115658 19.543717 -1.156065

C -2.347022 9.646576 -1.509452

H 1.954871 16.046984 4.440812

H 0.650729 16.989488 3.686761

H 0.216866 14.556003 3.550469

H -1.097623 15.350955 4.519786

H 2.219479 15.932561 6.897280

H 0.972029 16.691053 7.973653

H 0.644825 14.201374 7.790516

H -0.784348 15.131292 7.242874

H 1.561516 10.463719 3.811507

H 2.436343 10.670661 6.746865

H 1.273858 8.147431 3.974536

H -0.366090 10.632790 7.109613

H -1.457366 8.641599 8.109449

H -1.190507 6.405037 7.049190

H 0.175564 6.165235 4.981603

H 6.628485 9.081937 5.603443

H 4.491040 9.665668 6.711910

H 3.366693 12.035943 3.259205

H 5.531242 11.492457 2.196898

H 7.155409 9.982986 3.343729

H -1.356296 12.921837 4.029213

H -1.561615 11.195765 4.423354

H -2.103323 10.092093 2.502743

H 1.095623 12.993586 2.300315

H 0.116586 10.589524 -1.148309

H -0.123718 8.109396 -0.826430

H -2.220017 20.949992 2.885999

H 0.960589 18.093525 2.373236

H -0.504302 20.329155 -1.016794

H -1.454228 19.816377 4.750302

H -1.128658 18.110257 4.351184

H 3.092622 20.795493 6.209463

H 0.472513 21.421441 4.971990

H -0.599476 20.569317 7.266886

H -1.597365 22.120775 8.926264

H -0.193102 23.871290 10.001715

H 2.217982 24.065008 9.413182

H 3.223555 22.527529 7.747994

H 1.267361 22.668376 3.181694

H 2.860790 23.063261 1.327429

H 4.723943 21.466375 0.912042

H 3.376200 18.999856 4.165099

H 4.977199 19.430673 2.367632

H 3.934866 18.995930 7.319265

H 3.059562 17.468754 7.564553

H 4.781170 19.302374 9.428276

H 2.190704 19.872064 12.828166

H 0.547653 18.659924 9.031473

H 4.462060 15.055956 8.369028

H 3.015970 13.338928 12.059069

H 1.696843 11.736009 8.294059

H 3.087779 14.543804 6.312087

H 4.213486 13.172274 6.362002

H 2.884624 19.098262 -1.796322

H 1.459600 20.137368 -1.798462

H 2.623676 20.232006 -0.468579

H 2.968287 18.205320 1.079229

H 1.897647 16.785887 0.955785

H 3.083714 17.115947 -0.299799

H -0.012999 16.795512 -0.810983

H -0.156609 18.138562 -1.951542

H 1.225241 17.043827 -2.041899

H -0.742443 22.846632 -0.722220

H -2.177414 23.847317 -0.488397

H -1.137359 23.454737 0.885031

H -3.237007 22.607746 2.106948

H -4.249189 22.889635 0.695037

H -4.190133 21.282356 1.418415

H -2.226613 21.064056 -1.829786

H -3.540831 20.313096 -0.920250

H -3.707663 21.966043 -1.521123

H 2.444288 14.624606 -0.719442

H 0.692795 14.341461 -0.786216

H 1.541054 14.471172 0.773106

H 4.019243 12.855039 0.058874

H 3.135954 12.527713 1.536059

H 3.464595 11.199705 0.396038

H 2.823167 12.610477 -2.225394

H 2.200492 11.020748 -1.796089

H 1.088811 12.307863 -2.301273

H -1.484898 9.835198 -2.158769

H -2.976124 8.908090 -2.017287

H -2.918583 10.578449 -1.444591

H -0.707875 7.535017 0.736261

H -1.558149 7.085796 -0.745513

H -3.845291 9.650690 0.814777

H -3.776810 8.023291 0.139076

H -2.960468 8.369283 1.659675

H 3.126159 10.807235 12.271203

H 1.861237 9.580956 12.162942

H 2.948717 9.856548 10.797754

H 1.345546 12.541664 12.914046

H 0.013133 12.899840 11.812132

H 0.027547 11.388482 12.727964

H 0.852351 10.126307 9.334743

H -0.221106 9.988509 10.721412

H -0.318027 11.413379 9.686403

H 2.895212 15.725628 12.432263

H 4.138244 16.985011 12.474025

H 3.076765 16.844702 11.072811

H 6.075788 15.216505 12.794360

H 4.775961 14.033635 12.943841

H 6.073817 13.776630 11.773137

H 6.448760 15.499800 9.931214

H 5.156764 16.563279 9.406662

H 6.058297 16.950383 10.877298

H 6.174362 18.459294 11.310814

H 6.353880 20.051624 10.552875

H 6.996633 19.773864 12.168447

H 5.638873 21.847064 12.934617

H 4.909851 22.024976 11.335964

H 3.881099 21.866129 12.761624

H 5.566954 19.464009 14.120346

H 3.816199 19.656613 14.060699

H 4.566604 18.203041 13.374802

H 0.263146 20.984494 12.970105

H -0.392445 21.430530 11.393640

H -1.453347 20.758293 12.640136

H -1.164856 17.875359 10.286954

H -2.275496 18.912109 11.178339

H -1.346538 19.581719 9.837511

H 0.556094 18.519724 13.599806

H -1.152673 18.199182 13.309223

H 0.081253 17.248016 12.474270

S 8.936182 17.780935 4.917144

C 10.215093 19.063662 5.404435

C 8.940027 17.700426 3.088880

H 10.085265 19.001829 6.494056

C 9.963045 20.522636 5.012436

C 11.595732 18.510039 5.089797

H 8.208607 16.915432 2.887594

H 9.927631 17.368043 2.754282

C 8.518973 18.947003 2.323748

C 11.042642 21.417869 4.938696

C 8.664443 21.028821 4.851347

C 12.306860 17.829489 6.089484

C 12.178195 18.629222 3.816945

O 7.380052 19.324248 2.157854

O 9.596000 19.574845 1.803056

C 10.834464 22.776844 4.693494

H 12.056605 21.055036 5.077986

C 8.459060 22.388806 4.602362

H 7.811753 20.365307 4.919467

C 13.561953 17.277707 5.829570

H 11.871682 17.729130 7.081112

C 13.432802 18.074511 3.555879

H 11.651475 19.162959 3.032500

C 9.310702 20.771150 1.054333

C 9.539557 23.269460 4.520545

H 11.689003 23.448870 4.639615

H 7.444063 22.755824 4.475121

C 14.129684 17.396590 4.558929

H 14.095911 16.756774 6.620886

H 13.867691 18.175716 2.563936

H 10.277245 21.118700 0.685375

H 8.638721 20.549916 0.221215

H 8.855113 21.523778 1.702517

H 9.375387 24.327699 4.327655

H 15.107908 16.967625 4.352938

**TSS-05**

Mo 4.223635 15.314206 2.608848

Mo 5.417229 17.131781 5.659605

O 6.028245 13.630048 5.905576

O 7.929221 13.720546 4.253004

O 6.802083 15.785941 4.948259

O 5.607711 14.131874 3.529626

O 4.680598 14.946358 1.019856

O 2.880925 13.984298 3.159685

O 2.256596 15.177317 2.598399

O 3.996314 17.241086 2.576390

O 5.301068 16.939342 3.123258

O 3.975859 15.781937 5.088625

O 3.471178 17.112870 5.364462

O 5.459434 16.778471 7.325460

O 5.231674 18.988721 5.549056

O 6.899225 18.423910 5.372942

S 6.643902 14.238337 4.714890

C 3.157641 13.474896 6.684155

C 3.063451 13.406201 8.186142

C 2.273891 12.464866 8.856756

C 2.243291 12.438613 10.258882

C 1.414788 11.405473 11.031531

C 3.023631 13.359590 10.968305

C 3.826346 14.306819 10.309028

C 3.833844 14.329016 8.910367

C 2.463207 11.165246 5.733329

C 3.778466 10.877545 5.058012

C 4.703590 10.048976 5.714303

C 5.910032 9.721362 5.091767

C 6.205911 10.226433 3.820413

C 5.292439 11.063267 3.172807

C 4.076551 11.381092 3.785849

C 1.240991 10.704436 4.864636

C 0.531679 9.521329 5.480774

C 0.677119 8.261835 4.881104

C 0.057820 7.144099 5.447598

C -0.709912 7.278535 6.608649

C -0.859257 8.535283 7.204311

C -0.240590 9.655668 6.643723

C -0.844085 11.933080 3.977297

C -0.538801 11.578962 2.537998

C -1.324112 10.587516 1.926041

C -1.100263 10.239010 0.591862

C -1.920873 9.152269 -0.112531

C -1.028172 7.902235 -0.244401

C -3.193528 8.782270 0.672479

C -0.063499 10.879766 -0.112014

C 0.729159 11.863727 0.485196

C 1.872136 12.563096 -0.263575

C 0.475152 12.217489 1.825950

C 0.965007 13.003309 5.466810

C 0.278597 14.883824 6.987860

C 1.119224 16.174705 6.996661

C 0.862713 16.336182 4.566204

C -0.014515 15.084517 4.514530

C 1.098530 18.401823 5.850627

C 2.933408 18.580717 7.660712

C 2.678299 18.951237 9.100941

C 3.788397 19.294177 9.884278

C 3.624698 19.609436 11.239016

C 4.817857 19.984306 12.126608

C 4.682381 19.293702 13.499024

C 4.810096 21.515650 12.300847

C 6.156031 19.547029 11.505308

C 2.329880 19.603831 11.780028

C 1.210229 19.259789 11.006666

C -0.173942 19.268622 11.665152

C -0.171919 18.253719 12.826179

C -0.454652 20.688100 12.198286

C -1.298432 18.886813 10.683249

C 1.395636 18.921646 9.659705

C 1.997582 20.554436 6.346778

C 1.373077 21.456542 7.383683

C 2.164708 22.437909 7.996818

C 1.598898 23.304374 8.937124

C 0.245697 23.193762 9.269914

C -0.543684 22.210163 8.663835

C 0.016767 21.340767 7.725014

C 1.258486 20.621944 4.968622

C 2.217546 20.825045 3.817601

C 2.075347 21.961531 3.004841

C 2.973613 22.184606 1.959430

C 4.019590 21.283407 1.721706

C 4.159127 20.151062 2.526425

C 3.250137 19.913839 3.564162

C -0.729078 19.146403 4.225655

C -0.642826 19.484286 2.754403

C -1.519018 20.441245 2.227474

C -1.485383 20.742441 0.859398

C -2.413113 21.792998 0.238389

C -3.002850 21.253393 -1.080168

C -1.571694 23.054952 -0.037351

C -3.583908 22.159730 1.169976

C -0.547043 20.088215 0.047282

C 0.345575 19.136742 0.566064

C 0.278956 18.828639 1.931329

N 2.193526 12.637191 5.950784

N 0.361059 11.922527 4.837693

N 0.192971 14.173038 5.681270

N 0.661322 17.057758 5.875552

N 1.882436 19.098591 6.748600

N 0.600555 19.269319 4.878765

C 4.633728 15.304818 11.149426

C 5.624075 16.116945 10.304305

C 5.430218 14.538492 12.227082

C 3.626421 16.259195 11.820432

C 2.390020 10.354718 11.597797

C 0.376338 10.698859 10.138609

C 0.659130 12.098392 12.183901

C 1.994735 12.093185 -1.726678

C 3.191433 12.253771 0.466067

C 1.610471 14.079644 -0.257421

C 1.347099 18.456978 -0.376370

C 0.552640 17.562853 -1.350156

C 2.367939 17.595687 0.380425

C 2.115658 19.543717 -1.156065

C -2.347022 9.646576 -1.509452

H 1.954871 16.046984 4.440812

H 0.650729 16.989488 3.686761

H 0.216866 14.556003 3.550469

H -1.097623 15.350955 4.519786

H 2.219479 15.932561 6.897280

H 0.972029 16.691053 7.973653

H 0.644825 14.201374 7.790516

H -0.784348 15.131292 7.242874

H 1.561516 10.463719 3.811507

H 2.436343 10.670661 6.746865

H 1.273858 8.147431 3.974536

H -0.366090 10.632790 7.109613

H -1.457366 8.641599 8.109449

H -1.190507 6.405037 7.049190

H 0.175564 6.165235 4.981603

H 6.628485 9.081937 5.603443

H 4.491040 9.665668 6.711910

H 3.366693 12.035943 3.259205

H 5.531242 11.492457 2.196898

H 7.155409 9.982986 3.343729

H -1.356296 12.921837 4.029213

H -1.561615 11.195765 4.423354

H -2.103323 10.092093 2.502743

H 1.095623 12.993586 2.300315

H 0.116586 10.589524 -1.148309

H -0.123718 8.109396 -0.826430

H -2.220017 20.949992 2.885999

H 0.960589 18.093525 2.373236

H -0.504302 20.329155 -1.016794

H -1.454228 19.816377 4.750302

H -1.128658 18.110257 4.351184

H 3.092622 20.795493 6.209463

H 0.472513 21.421441 4.971990

H -0.599476 20.569317 7.266886

H -1.597365 22.120775 8.926264

H -0.193102 23.871290 10.001715

H 2.217982 24.065008 9.413182

H 3.223555 22.527529 7.747994

H 1.267361 22.668376 3.181694

H 2.860790 23.063261 1.327429

H 4.723943 21.466375 0.912042

H 3.376200 18.999856 4.165099

H 4.977199 19.430673 2.367632

H 3.934866 18.995930 7.319265

H 3.059562 17.468754 7.564553

H 4.781170 19.302374 9.428276

H 2.190704 19.872064 12.828166

H 0.547653 18.659924 9.031473

H 4.462060 15.055956 8.369028

H 3.015970 13.338928 12.059069

H 1.696843 11.736009 8.294059

H 3.087779 14.543804 6.312087

H 4.213486 13.172274 6.362002

H 2.884624 19.098262 -1.796322

H 1.459600 20.137368 -1.798462

H 2.623676 20.232006 -0.468579

H 2.968287 18.205320 1.079229

H 1.897647 16.785887 0.955785

H 3.083714 17.115947 -0.299799

H -0.012999 16.795512 -0.810983

H -0.156609 18.138562 -1.951542

H 1.225241 17.043827 -2.041899

H -0.742443 22.846632 -0.722220

H -2.177414 23.847317 -0.488397

H -1.137359 23.454737 0.885031

H -3.237007 22.607746 2.106948

H -4.249189 22.889635 0.695037

H -4.190133 21.282356 1.418415

H -2.226613 21.064056 -1.829786

H -3.540831 20.313096 -0.920250

H -3.707663 21.966043 -1.521123

H 2.444288 14.624606 -0.719442

H 0.692795 14.341461 -0.786216

H 1.541054 14.471172 0.773106

H 4.019243 12.855039 0.058874

H 3.135954 12.527713 1.536059

H 3.464595 11.199705 0.396038

H 2.823167 12.610477 -2.225394

H 2.200492 11.020748 -1.796089

H 1.088811 12.307863 -2.301273

H -1.484898 9.835198 -2.158769

H -2.976124 8.908090 -2.017287

H -2.918583 10.578449 -1.444591

H -0.707875 7.535017 0.736261

H -1.558149 7.085796 -0.745513

H -3.845291 9.650690 0.814777

H -3.776810 8.023291 0.139076

H -2.960468 8.369283 1.659675

H 3.126159 10.807235 12.271203

H 1.861237 9.580956 12.162942

H 2.948717 9.856548 10.797754

H 1.345546 12.541664 12.914046

H 0.013133 12.899840 11.812132

H 0.027547 11.388482 12.727964

H 0.852351 10.126307 9.334743

H -0.221106 9.988509 10.721412

H -0.318027 11.413379 9.686403

H 2.895212 15.725628 12.432263

H 4.138244 16.985011 12.474025

H 3.076765 16.844702 11.072811

H 6.075788 15.216505 12.794360

H 4.775961 14.033635 12.943841

H 6.073817 13.776630 11.773137

H 6.448760 15.499800 9.931214

H 5.156764 16.563279 9.406662

H 6.058297 16.950383 10.877298

H 6.174362 18.459294 11.310814

H 6.353880 20.051624 10.552875

H 6.996633 19.773864 12.168447

H 5.638873 21.847064 12.934617

H 4.909851 22.024976 11.335964

H 3.881099 21.866129 12.761624

H 5.566954 19.464009 14.120346

H 3.816199 19.656613 14.060699

H 4.566604 18.203041 13.374802

H 0.263146 20.984494 12.970105

H -0.392445 21.430530 11.393640

H -1.453347 20.758293 12.640136

H -1.164856 17.875359 10.286954

H -2.275496 18.912109 11.178339

H -1.346538 19.581719 9.837511

H 0.556094 18.519724 13.599806

H -1.152673 18.199182 13.309223

H 0.081253 17.248016 12.474270

S 9.022459 17.744551 5.009648

C 9.914616 19.174166 5.809198

C 8.918957 18.223397 3.250533

H 9.224030 20.018527 5.738224

C 11.196492 19.544799 5.086432

C 10.070072 18.806362 7.286809

H 8.551397 17.333037 2.736501

H 9.929428 18.442719 2.880236

C 7.997356 19.375865 2.881308

C 11.440996 20.888662 4.773460

C 12.160726 18.588784 4.728613

C 8.909811 18.624420 8.059460

C 11.317978 18.663633 7.906460

O 7.173455 19.319235 1.993825

O 8.251019 20.490290 3.591736

C 12.620638 21.274171 4.131889

H 10.699417 21.639093 5.034773

C 13.339230 18.970768 4.085334

H 11.982744 17.540360 4.950023

C 9.001914 18.298155 9.412130

H 7.938133 18.729107 7.590149

C 11.407798 18.344828 9.264544

H 12.228869 18.811225 7.334729

C 7.386630 21.606747 3.314069

C 13.574779 20.315581 3.785766

H 12.790138 22.323018 3.898474

H 14.073722 18.215339 3.815969

C 10.251244 18.157191 10.021750

H 8.093372 18.151183 9.989427

H 12.387762 18.242542 9.727069

H 7.760865 22.424696 3.932223

H 7.435195 21.877450 2.255179

H 6.357535 21.359829 3.584473

H 14.492430 20.611901 3.282231

H 10.322273 17.904412 11.077629

**TSS-06**

Mo 4.247438 15.309339 2.608794

Mo 5.411408 17.130704 5.669513

O 6.006485 13.637996 5.917462

O 7.944259 13.717548 4.313479

O 6.809111 15.791944 4.984839

O 5.643591 14.145053 3.530618

O 4.695655 14.938077 1.017690

O 2.908891 13.976161 3.164015

O 2.281122 15.168055 2.605828

O 4.004747 17.236008 2.562300

O 5.319117 16.950619 3.100648

O 3.998027 15.768141 5.071097

O 3.464202 17.084404 5.358909

O 5.430133 16.796847 7.339565

O 5.197299 18.971794 5.496951

O 6.900034 18.433013 5.419666

S 6.652901 14.245404 4.741292

C 3.157641 13.474896 6.684155

C 3.063451 13.406201 8.186142

C 2.273891 12.464866 8.856756

C 2.243291 12.438613 10.258882

C 1.414788 11.405473 11.031531

C 3.023631 13.359590 10.968305

C 3.826346 14.306819 10.309028

C 3.833844 14.329016 8.910367

C 2.463207 11.165246 5.733329

C 3.778466 10.877545 5.058012

C 4.703590 10.048976 5.714303

C 5.910032 9.721362 5.091767

C 6.205911 10.226433 3.820413

C 5.292439 11.063267 3.172807

C 4.076551 11.381092 3.785849

C 1.240991 10.704436 4.864636

C 0.531679 9.521329 5.480774

C 0.677119 8.261835 4.881104

C 0.057820 7.144099 5.447598

C -0.709912 7.278535 6.608649

C -0.859257 8.535283 7.204311

C -0.240590 9.655668 6.643723

C -0.844085 11.933080 3.977297

C -0.538801 11.578962 2.537998

C -1.324112 10.587516 1.926041

C -1.100263 10.239010 0.591862

C -1.920873 9.152269 -0.112531

C -1.028172 7.902235 -0.244401

C -3.193528 8.782270 0.672479

C -0.063499 10.879766 -0.112014

C 0.729159 11.863727 0.485196

C 1.872136 12.563096 -0.263575

C 0.475152 12.217489 1.825950

C 0.965007 13.003309 5.466810

C 0.278597 14.883824 6.987860

C 1.119224 16.174705 6.996661

C 0.862713 16.336182 4.566204

C -0.014515 15.084517 4.514530

C 1.098530 18.401823 5.850627

C 2.933408 18.580717 7.660712

C 2.678299 18.951237 9.100941

C 3.788397 19.294177 9.884278

C 3.624698 19.609436 11.239016

C 4.817857 19.984306 12.126608

C 4.682381 19.293702 13.499024

C 4.810096 21.515650 12.300847

C 6.156031 19.547029 11.505308

C 2.329880 19.603831 11.780028

C 1.210229 19.259789 11.006666

C -0.173942 19.268622 11.665152

C -0.171919 18.253719 12.826179

C -0.454652 20.688100 12.198286

C -1.298432 18.886813 10.683249

C 1.395636 18.921646 9.659705

C 1.997582 20.554436 6.346778

C 1.373077 21.456542 7.383683

C 2.164708 22.437909 7.996818

C 1.598898 23.304374 8.937124

C 0.245697 23.193762 9.269914

C -0.543684 22.210163 8.663835

C 0.016767 21.340767 7.725014

C 1.258486 20.621944 4.968622

C 2.217546 20.825045 3.817601

C 2.075347 21.961531 3.004841

C 2.973613 22.184606 1.959430

C 4.019590 21.283407 1.721706

C 4.159127 20.151062 2.526425

C 3.250137 19.913839 3.564162

C -0.729078 19.146403 4.225655

C -0.642826 19.484286 2.754403

C -1.519018 20.441245 2.227474

C -1.485383 20.742441 0.859398

C -2.413113 21.792998 0.238389

C -3.002850 21.253393 -1.080168

C -1.571694 23.054952 -0.037351

C -3.583908 22.159730 1.169976

C -0.547043 20.088215 0.047282

C 0.345575 19.136742 0.566064

C 0.278956 18.828639 1.931329

N 2.193526 12.637191 5.950784

N 0.361059 11.922527 4.837693

N 0.192971 14.173038 5.681270

N 0.661322 17.057758 5.875552

N 1.882436 19.098591 6.748600

N 0.600555 19.269319 4.878765

C 4.633728 15.304818 11.149426

C 5.624075 16.116945 10.304305

C 5.430218 14.538492 12.227082

C 3.626421 16.259195 11.820432

C 2.390020 10.354718 11.597797

C 0.376338 10.698859 10.138609

C 0.659130 12.098392 12.183901

C 1.994735 12.093185 -1.726678

C 3.191433 12.253771 0.466067

C 1.610471 14.079644 -0.257421

C 1.347099 18.456978 -0.376370

C 0.552640 17.562853 -1.350156

C 2.367939 17.595687 0.380425

C 2.115658 19.543717 -1.156065

C -2.347022 9.646576 -1.509452

H 1.954871 16.046984 4.440812

H 0.650729 16.989488 3.686761

H 0.216866 14.556003 3.550469

H -1.097623 15.350955 4.519786

H 2.219479 15.932561 6.897280

H 0.972029 16.691053 7.973653

H 0.644825 14.201374 7.790516

H -0.784348 15.131292 7.242874

H 1.561516 10.463719 3.811507

H 2.436343 10.670661 6.746865

H 1.273858 8.147431 3.974536

H -0.366090 10.632790 7.109613

H -1.457366 8.641599 8.109449

H -1.190507 6.405037 7.049190

H 0.175564 6.165235 4.981603

H 6.628485 9.081937 5.603443

H 4.491040 9.665668 6.711910

H 3.366693 12.035943 3.259205

H 5.531242 11.492457 2.196898

H 7.155409 9.982986 3.343729

H -1.356296 12.921837 4.029213

H -1.561615 11.195765 4.423354

H -2.103323 10.092093 2.502743

H 1.095623 12.993586 2.300315

H 0.116586 10.589524 -1.148309

H -0.123718 8.109396 -0.826430

H -2.220017 20.949992 2.885999

H 0.960589 18.093525 2.373236

H -0.504302 20.329155 -1.016794

H -1.454228 19.816377 4.750302

H -1.128658 18.110257 4.351184

H 3.092622 20.795493 6.209463

H 0.472513 21.421441 4.971990

H -0.599476 20.569317 7.266886

H -1.597365 22.120775 8.926264

H -0.193102 23.871290 10.001715

H 2.217982 24.065008 9.413182

H 3.223555 22.527529 7.747994

H 1.267361 22.668376 3.181694

H 2.860790 23.063261 1.327429

H 4.723943 21.466375 0.912042

H 3.376200 18.999856 4.165099

H 4.977199 19.430673 2.367632

H 3.934866 18.995930 7.319265

H 3.059562 17.468754 7.564553

H 4.781170 19.302374 9.428276

H 2.190704 19.872064 12.828166

H 0.547653 18.659924 9.031473

H 4.462060 15.055956 8.369028

H 3.015970 13.338928 12.059069

H 1.696843 11.736009 8.294059

H 3.087779 14.543804 6.312087

H 4.213486 13.172274 6.362002

H 2.884624 19.098262 -1.796322

H 1.459600 20.137368 -1.798462

H 2.623676 20.232006 -0.468579

H 2.968287 18.205320 1.079229

H 1.897647 16.785887 0.955785

H 3.083714 17.115947 -0.299799

H -0.012999 16.795512 -0.810983

H -0.156609 18.138562 -1.951542

H 1.225241 17.043827 -2.041899

H -0.742443 22.846632 -0.722220

H -2.177414 23.847317 -0.488397

H -1.137359 23.454737 0.885031

H -3.237007 22.607746 2.106948

H -4.249189 22.889635 0.695037

H -4.190133 21.282356 1.418415

H -2.226613 21.064056 -1.829786

H -3.540831 20.313096 -0.920250

H -3.707663 21.966043 -1.521123

H 2.444288 14.624606 -0.719442

H 0.692795 14.341461 -0.786216

H 1.541054 14.471172 0.773106

H 4.019243 12.855039 0.058874

H 3.135954 12.527713 1.536059

H 3.464595 11.199705 0.396038

H 2.823167 12.610477 -2.225394

H 2.200492 11.020748 -1.796089

H 1.088811 12.307863 -2.301273

H -1.484898 9.835198 -2.158769

H -2.976124 8.908090 -2.017287

H -2.918583 10.578449 -1.444591

H -0.707875 7.535017 0.736261

H -1.558149 7.085796 -0.745513

H -3.845291 9.650690 0.814777

H -3.776810 8.023291 0.139076

H -2.960468 8.369283 1.659675

H 3.126159 10.807235 12.271203

H 1.861237 9.580956 12.162942

H 2.948717 9.856548 10.797754

H 1.345546 12.541664 12.914046

H 0.013133 12.899840 11.812132

H 0.027547 11.388482 12.727964

H 0.852351 10.126307 9.334743

H -0.221106 9.988509 10.721412

H -0.318027 11.413379 9.686403

H 2.895212 15.725628 12.432263

H 4.138244 16.985011 12.474025

H 3.076765 16.844702 11.072811

H 6.075788 15.216505 12.794360

H 4.775961 14.033635 12.943841

H 6.073817 13.776630 11.773137

H 6.448760 15.499800 9.931214

H 5.156764 16.563279 9.406662

H 6.058297 16.950383 10.877298

H 6.174362 18.459294 11.310814

H 6.353880 20.051624 10.552875

H 6.996633 19.773864 12.168447

H 5.638873 21.847064 12.934617

H 4.909851 22.024976 11.335964

H 3.881099 21.866129 12.761624

H 5.566954 19.464009 14.120346

H 3.816199 19.656613 14.060699

H 4.566604 18.203041 13.374802

H 0.263146 20.984494 12.970105

H -0.392445 21.430530 11.393640

H -1.453347 20.758293 12.640136

H -1.164856 17.875359 10.286954

H -2.275496 18.912109 11.178339

H -1.346538 19.581719 9.837511

H 0.556094 18.519724 13.599806

H -1.152673 18.199182 13.309223

H 0.081253 17.248016 12.474270

S 9.040048 17.872923 5.184685

C 10.201202 18.562143 6.481612

C 9.143059 19.093391 3.837213

H 10.181525 17.722073 7.188628

C 9.780059 19.816040 7.251973

C 11.605879 18.627746 5.897227

H 10.198857 19.317897 3.636000

H 8.618918 20.014282 4.099135

C 8.549078 18.482107 2.574378

C 10.661220 20.293244 8.239092

C 8.559256 20.475446 7.072202

C 12.399324 17.470518 5.865098

C 12.128285 19.812988 5.357115

O 8.829209 17.384953 2.144153

O 7.699899 19.336636 1.980327

C 10.336980 21.404524 9.015444

H 11.613349 19.791086 8.397077

C 8.237978 21.595568 7.847993

H 7.841177 20.102064 6.354490

C 13.678103 17.495202 5.309351

H 12.008153 16.542753 6.274869

C 13.407349 19.837716 4.795036

H 11.541166 20.726222 5.396156

C 7.093469 18.848080 0.765425

C 9.121057 22.066383 8.818084

H 11.035759 21.754786 9.772955

H 7.286828 22.095486 7.686995

C 14.186903 18.679874 4.768963

H 14.278177 16.588484 5.299561

H 13.794982 20.766588 4.382379

H 6.526831 19.690829 0.366076

H 7.864407 18.538236 0.053133

H 6.439302 18.007231 0.996106

H 8.868071 22.938033 9.418280

H 15.184467 18.700328 4.335593

**TSS-07**

Mo 4.235172 15.315690 2.608366

Mo 5.406173 17.123508 5.667237

O 6.013916 13.642097 5.906738

O 7.936006 13.719598 4.282035

O 6.807482 15.795655 4.962774

O 5.627150 14.146429 3.524243

O 4.685200 14.961285 1.013851

O 2.899179 13.976887 3.154255

O 2.269205 15.171430 2.603599

O 3.990030 17.242692 2.574935

O 5.304120 16.956748 3.111940

O 3.988355 15.762421 5.071243

O 3.460254 17.079332 5.368841

O 5.450616 16.767424 7.334513

O 5.185244 18.971434 5.545946

O 6.862649 18.445241 5.401391

S 6.650497 14.248488 4.724765

C 3.157641 13.474896 6.684155

C 3.063451 13.406201 8.186142

C 2.273891 12.464866 8.856756

C 2.243291 12.438613 10.258882

C 1.414788 11.405473 11.031531

C 3.023631 13.359590 10.968305

C 3.826346 14.306819 10.309028

C 3.833844 14.329016 8.910367

C 2.463207 11.165246 5.733329

C 3.778466 10.877545 5.058012

C 4.703590 10.048976 5.714303

C 5.910032 9.721362 5.091767

C 6.205911 10.226433 3.820413

C 5.292439 11.063267 3.172807

C 4.076551 11.381092 3.785849

C 1.240991 10.704436 4.864636

C 0.531679 9.521329 5.480774

C 0.677119 8.261835 4.881104

C 0.057820 7.144099 5.447598

C -0.709912 7.278535 6.608649

C -0.859257 8.535283 7.204311

C -0.240590 9.655668 6.643723

C -0.844085 11.933080 3.977297

C -0.538801 11.578962 2.537998

C -1.324112 10.587516 1.926041

C -1.100263 10.239010 0.591862

C -1.920873 9.152269 -0.112531

C -1.028172 7.902235 -0.244401

C -3.193528 8.782270 0.672479

C -0.063499 10.879766 -0.112014

C 0.729159 11.863727 0.485196

C 1.872136 12.563096 -0.263575

C 0.475152 12.217489 1.825950

C 0.965007 13.003309 5.466810

C 0.278597 14.883824 6.987860

C 1.119224 16.174705 6.996661

C 0.862713 16.336182 4.566204

C -0.014515 15.084517 4.514530

C 1.098530 18.401823 5.850627

C 2.933408 18.580717 7.660712

C 2.678299 18.951237 9.100941

C 3.788397 19.294177 9.884278

C 3.624698 19.609436 11.239016

C 4.817857 19.984306 12.126608

C 4.682381 19.293702 13.499024

C 4.810096 21.515650 12.300847

C 6.156031 19.547029 11.505308

C 2.329880 19.603831 11.780028

C 1.210229 19.259789 11.006666

C -0.173942 19.268622 11.665152

C -0.171919 18.253719 12.826179

C -0.454652 20.688100 12.198286

C -1.298432 18.886813 10.683249

C 1.395636 18.921646 9.659705

C 1.997582 20.554436 6.346778

C 1.373077 21.456542 7.383683

C 2.164708 22.437909 7.996818

C 1.598898 23.304374 8.937124

C 0.245697 23.193762 9.269914

C -0.543684 22.210163 8.663835

C 0.016767 21.340767 7.725014

C 1.258486 20.621944 4.968622

C 2.217546 20.825045 3.817601

C 2.075347 21.961531 3.004841

C 2.973613 22.184606 1.959430

C 4.019590 21.283407 1.721706

C 4.159127 20.151062 2.526425

C 3.250137 19.913839 3.564162

C -0.729078 19.146403 4.225655

C -0.642826 19.484286 2.754403

C -1.519018 20.441245 2.227474

C -1.485383 20.742441 0.859398

C -2.413113 21.792998 0.238389

C -3.002850 21.253393 -1.080168

C -1.571694 23.054952 -0.037351

C -3.583908 22.159730 1.169976

C -0.547043 20.088215 0.047282

C 0.345575 19.136742 0.566064

C 0.278956 18.828639 1.931329

N 2.193526 12.637191 5.950784

N 0.361059 11.922527 4.837693

N 0.192971 14.173038 5.681270

N 0.661322 17.057758 5.875552

N 1.882436 19.098591 6.748600

N 0.600555 19.269319 4.878765

C 4.633728 15.304818 11.149426

C 5.624075 16.116945 10.304305

C 5.430218 14.538492 12.227082

C 3.626421 16.259195 11.820432

C 2.390020 10.354718 11.597797

C 0.376338 10.698859 10.138609

C 0.659130 12.098392 12.183901

C 1.994735 12.093185 -1.726678

C 3.191433 12.253771 0.466067

C 1.610471 14.079644 -0.257421

C 1.347099 18.456978 -0.376370

C 0.552640 17.562853 -1.350156

C 2.367939 17.595687 0.380425

C 2.115658 19.543717 -1.156065

C -2.347022 9.646576 -1.509452

H 1.954871 16.046984 4.440812

H 0.650729 16.989488 3.686761

H 0.216866 14.556003 3.550469

H -1.097623 15.350955 4.519786

H 2.219479 15.932561 6.897280

H 0.972029 16.691053 7.973653

H 0.644825 14.201374 7.790516

H -0.784348 15.131292 7.242874

H 1.561516 10.463719 3.811507

H 2.436343 10.670661 6.746865

H 1.273858 8.147431 3.974536

H -0.366090 10.632790 7.109613

H -1.457366 8.641599 8.109449

H -1.190507 6.405037 7.049190

H 0.175564 6.165235 4.981603

H 6.628485 9.081937 5.603443

H 4.491040 9.665668 6.711910

H 3.366693 12.035943 3.259205

H 5.531242 11.492457 2.196898

H 7.155409 9.982986 3.343729

H -1.356296 12.921837 4.029213

H -1.561615 11.195765 4.423354

H -2.103323 10.092093 2.502743

H 1.095623 12.993586 2.300315

H 0.116586 10.589524 -1.148309

H -0.123718 8.109396 -0.826430

H -2.220017 20.949992 2.885999

H 0.960589 18.093525 2.373236

H -0.504302 20.329155 -1.016794

H -1.454228 19.816377 4.750302

H -1.128658 18.110257 4.351184

H 3.092622 20.795493 6.209463

H 0.472513 21.421441 4.971990

H -0.599476 20.569317 7.266886

H -1.597365 22.120775 8.926264

H -0.193102 23.871290 10.001715

H 2.217982 24.065008 9.413182

H 3.223555 22.527529 7.747994

H 1.267361 22.668376 3.181694

H 2.860790 23.063261 1.327429

H 4.723943 21.466375 0.912042

H 3.376200 18.999856 4.165099

H 4.977199 19.430673 2.367632

H 3.934866 18.995930 7.319265

H 3.059562 17.468754 7.564553

H 4.781170 19.302374 9.428276

H 2.190704 19.872064 12.828166

H 0.547653 18.659924 9.031473

H 4.462060 15.055956 8.369028

H 3.015970 13.338928 12.059069

H 1.696843 11.736009 8.294059

H 3.087779 14.543804 6.312087

H 4.213486 13.172274 6.362002

H 2.884624 19.098262 -1.796322

H 1.459600 20.137368 -1.798462

H 2.623676 20.232006 -0.468579

H 2.968287 18.205320 1.079229

H 1.897647 16.785887 0.955785

H 3.083714 17.115947 -0.299799

H -0.012999 16.795512 -0.810983

H -0.156609 18.138562 -1.951542

H 1.225241 17.043827 -2.041899

H -0.742443 22.846632 -0.722220

H -2.177414 23.847317 -0.488397

H -1.137359 23.454737 0.885031

H -3.237007 22.607746 2.106948

H -4.249189 22.889635 0.695037

H -4.190133 21.282356 1.418415

H -2.226613 21.064056 -1.829786

H -3.540831 20.313096 -0.920250

H -3.707663 21.966043 -1.521123

H 2.444288 14.624606 -0.719442

H 0.692795 14.341461 -0.786216

H 1.541054 14.471172 0.773106

H 4.019243 12.855039 0.058874

H 3.135954 12.527713 1.536059

H 3.464595 11.199705 0.396038

H 2.823167 12.610477 -2.225394

H 2.200492 11.020748 -1.796089

H 1.088811 12.307863 -2.301273

H -1.484898 9.835198 -2.158769

H -2.976124 8.908090 -2.017287

H -2.918583 10.578449 -1.444591

H -0.707875 7.535017 0.736261

H -1.558149 7.085796 -0.745513

H -3.845291 9.650690 0.814777

H -3.776810 8.023291 0.139076

H -2.960468 8.369283 1.659675

H 3.126159 10.807235 12.271203

H 1.861237 9.580956 12.162942

H 2.948717 9.856548 10.797754

H 1.345546 12.541664 12.914046

H 0.013133 12.899840 11.812132

H 0.027547 11.388482 12.727964

H 0.852351 10.126307 9.334743

H -0.221106 9.988509 10.721412

H -0.318027 11.413379 9.686403

H 2.895212 15.725628 12.432263

H 4.138244 16.985011 12.474025

H 3.076765 16.844702 11.072811

H 6.075788 15.216505 12.794360

H 4.775961 14.033635 12.943841

H 6.073817 13.776630 11.773137

H 6.448760 15.499800 9.931214

H 5.156764 16.563279 9.406662

H 6.058297 16.950383 10.877298

H 6.174362 18.459294 11.310814

H 6.353880 20.051624 10.552875

H 6.996633 19.773864 12.168447

H 5.638873 21.847064 12.934617

H 4.909851 22.024976 11.335964

H 3.881099 21.866129 12.761624

H 5.566954 19.464009 14.120346

H 3.816199 19.656613 14.060699

H 4.566604 18.203041 13.374802

H 0.263146 20.984494 12.970105

H -0.392445 21.430530 11.393640

H -1.453347 20.758293 12.640136

H -1.164856 17.875359 10.286954

H -2.275496 18.912109 11.178339

H -1.346538 19.581719 9.837511

H 0.556094 18.519724 13.599806

H -1.152673 18.199182 13.309223

H 0.081253 17.248016 12.474270

S 9.029929 17.941877 5.071188

C 9.918906 18.961414 6.352963

C 8.976350 19.135084 3.696438

H 10.899349 19.171691 5.906495

C 10.157482 18.087388 7.579390

C 9.276968 20.304358 6.672813

H 9.995765 19.475410 3.463217

H 8.361688 19.994559 3.968413

C 8.422671 18.443049 2.458706

C 11.385715 18.199175 8.247077

C 9.194657 17.201013 8.083595

C 9.872408 21.476806 6.186728

C 8.126649 20.415318 7.466967

O 8.775736 17.351914 2.067715

O 7.518959 19.217051 1.836149

C 11.648578 17.450329 9.394391

H 12.142734 18.881850 7.864467

C 9.460990 16.446034 9.229046

H 8.239190 17.082918 7.586974

C 9.335033 22.732572 6.480544

H 10.774263 21.408473 5.579795

C 7.590240 21.667569 7.763861

H 7.653408 19.523465 7.855778

C 6.946528 18.648606 0.639638

C 10.684260 16.568332 9.889356

H 12.609224 17.550428 9.896093

H 8.706715 15.756391 9.597694

C 8.189999 22.830658 7.272103

H 9.816121 23.629950 6.096264

H 6.701189 21.735560 8.384374

H 6.324996 19.437873 0.213648

H 7.737070 18.367598 -0.063289

H 6.348744 17.775059 0.898538

H 10.889113 15.976834 10.779150

H 7.770439 23.806110 7.508848

**TSS-08**

Mo 4.366828 15.263087 2.615422

Mo 5.358830 17.107126 5.718207

O 5.850432 13.599277 6.011068

O 7.928370 13.628038 4.589075

O 6.788705 15.731207 5.136693

O 5.718634 14.098866 3.599866

O 4.897635 14.893005 1.050644

O 3.006201 13.929027 3.102736

O 2.403573 15.122405 2.519542

O 4.139811 17.193053 2.551798

O 5.412979 16.898099 3.175800

O 3.971478 15.745894 5.075390

O 3.431320 17.070320 5.316549

O 5.340328 16.795492 7.391022

O 5.170132 18.965853 5.562741

O 6.813389 18.406329 5.405328

S 6.614032 14.186531 4.898666

C 2.947968 13.439258 6.573267

C 2.775471 13.373982 8.067949

C 1.900939 12.479558 8.695493

C 1.800862 12.453994 10.094463

C 0.877972 11.468665 10.821193

C 2.597825 13.327578 10.844216

C 3.486888 14.225973 10.228357

C 3.562928 14.249472 8.831727

C 2.258952 11.138976 5.590759

C 3.627493 10.819944 5.048993

C 4.467676 9.982119 5.800955

C 5.727088 9.633503 5.308336

C 6.160410 10.127323 4.072503

C 5.330396 10.972283 3.329960

C 4.062526 11.311412 3.812070

C 1.112099 10.716287 4.607424

C 0.312082 9.553432 5.146391

C 0.433624 8.303139 4.523198

C -0.274033 7.204627 5.019906

C -1.106351 7.349723 6.134089

C -1.231739 8.597862 6.753246

C -0.524900 9.698567 6.262676

C -0.858455 12.004284 3.552478

C -0.446106 11.666808 2.135726

C -1.245758 10.755323 1.425367

C -0.930673 10.425518 0.105159

C -1.768258 9.430606 -0.706795

C -0.963466 8.120506 -0.819503

C -3.125649 9.132130 -0.042629

C 0.212248 10.999005 -0.483031

C 1.020479 11.901931 0.212981

C 2.273547 12.530582 -0.412236

C 0.671996 12.243552 1.535765

C 0.839324 13.016997 5.202765

C 0.074609 14.888075 6.693855

C 0.934598 16.162737 6.791881

C 0.878097 16.369217 4.352723

C -0.012106 15.133703 4.205435

C 0.993202 18.413644 5.693910

C 2.775530 18.539882 7.554858

C 2.545605 18.903633 9.001190

C 3.675780 19.202209 9.774437

C 3.538625 19.509955 11.133746

C 4.752647 19.843786 12.009390

C 4.606330 19.161800 13.385122

C 4.799815 21.374984 12.179628

C 6.068175 19.357988 11.376968

C 2.251245 19.533405 11.691646

C 1.113357 19.221635 10.931528

C -0.257328 19.237509 11.617395

C -0.225474 18.247762 12.800213

C -0.534816 20.665845 12.127031

C -1.398000 18.826791 10.666540

C 1.269902 18.902200 9.576150

C 1.845282 20.556313 6.303856

C 1.155276 21.419064 7.332849

C 1.894724 22.409984 7.994057

C 1.268809 23.241739 8.927484

C -0.092082 23.085620 9.206585

C -0.828669 22.091243 8.553520

C -0.209012 21.258239 7.619038

C 1.175956 20.658009 4.892674

C 2.186260 20.872969 3.788526

C 2.080773 22.018356 2.983259

C 3.000021 22.232482 1.953987

C 4.026086 21.308239 1.720444

C 4.132617 20.170271 2.522943

C 3.214202 19.950187 3.555652

C -0.742058 19.178242 3.969815

C -0.528121 19.539676 2.517532

C -1.269468 20.587466 1.957898

C -1.107086 20.914735 0.604913

C -1.886977 22.059709 -0.051610

C -2.448988 21.592562 -1.409267

C -0.909123 23.233045 -0.260031

C -3.067522 22.534918 0.816233

C -0.174838 20.194333 -0.157416

C 0.585101 19.151753 0.395443

C 0.384223 18.817864 1.741097

N 2.009384 12.616486 5.791884

N 0.271268 11.958542 4.507588

N 0.079110 14.201971 5.370702

N 0.572084 17.066500 5.653744

N 1.727724 19.087143 6.654858

N 0.518624 19.311113 4.744859

C 4.318182 15.166438 11.110917

C 5.373788 15.938918 10.309878

C 5.039690 14.338449 12.195588

C 3.345433 16.163683 11.770341

C 1.762535 10.363587 11.431376

C -0.152845 10.822223 9.875003

C 0.106209 12.202517 11.936872

C 2.514601 12.053554 -1.858343

C 3.495850 12.144733 0.440368

C 2.103194 14.059692 -0.421545

C 1.585794 18.394587 -0.486781

C 0.778694 17.484197 -1.434053

C 2.554599 17.539529 0.343020

C 2.416759 19.405733 -1.303052

C -2.038940 10.007050 -2.111057

H 1.972577 16.064023 4.313856

H 0.750141 17.043661 3.470787

H 0.290874 14.615127 3.255963

H -1.087106 15.419820 4.123230

H 2.034377 15.905082 6.769084

H 0.725846 16.666007 7.764287

H 0.365341 14.185405 7.509791

H -1.000474 15.151436 6.869721

H 1.522898 10.471991 3.587328

H 2.120422 10.642097 6.594035

H 1.080088 8.180631 3.652544

H -0.631771 10.668983 6.746635

H -1.880336 8.712512 7.621895

H -1.656010 6.491263 6.520186

H -0.174704 6.232481 4.535857

H 6.379370 8.987309 5.894887

H 4.147683 9.608216 6.773301

H 3.421258 11.974776 3.212464

H 5.674628 11.392134 2.381878

H 7.150912 9.868450 3.698467

H -1.355164 13.002087 3.578532

H -1.623693 11.274167 3.925588

H -2.108708 10.307534 1.915299

H 1.299333 12.963358 2.083858

H 0.461626 10.722102 -1.508520

H -0.001881 8.276799 -1.320092

H -1.966894 21.144923 2.579798

H 0.953369 18.003223 2.205082

H -0.032389 20.454151 -1.208623

H -1.517986 19.833511 4.438319

H -1.138249 18.136669 4.050480

H 2.940267 20.818647 6.232011

H 0.392240 21.459603 4.874557

H -0.785433 20.481392 7.119987

H -1.888109 21.965733 8.775333

H -0.577912 23.736634 9.932962

H 1.846897 24.010627 9.440602

H 2.959252 22.534141 7.788554

H 1.282079 22.738679 3.150845

H 2.915351 23.119293 1.328238

H 4.736954 21.475827 0.912710

H 3.327902 19.039702 4.163963

H 4.922803 19.426174 2.359750

H 3.783155 18.935190 7.209633

H 2.869559 17.424885 7.453816

H 4.664286 19.177569 9.308044

H 2.132772 19.794834 12.744076

H 0.406620 18.674214 8.955787

H 4.257159 14.937295 8.322481

H 2.536310 13.306320 11.933171

H 1.311663 11.785901 8.102324

H 2.920589 14.507453 6.196923

H 4.013343 13.115738 6.306625

H 3.186776 18.896325 -1.892743

H 1.801703 19.981512 -2.000872

H 2.928909 20.116317 -0.642967

H 3.131168 18.159741 1.051119

H 2.043025 16.756572 0.920133

H 3.290816 17.024767 -0.288814

H 0.179479 16.758677 -0.873843

H 0.097731 18.057529 -2.069986

H 1.444770 16.915614 -2.092515

H -0.073621 22.953180 -0.910856

H -1.408704 24.091137 -0.721090

H -0.481378 23.570280 0.690044

H -2.733600 22.936485 1.778565

H -3.626024 23.333572 0.314743

H -3.774098 21.722268 1.014982

H -1.653280 21.331790 -2.115681

H -3.087451 20.710386 -1.293662

H -3.051586 22.376327 -1.880357

H 3.025179 14.554711 -0.757488

H 1.283774 14.380653 -1.066327

H 1.917265 14.450374 0.594586

H 4.399541 12.667649 0.092597

H 3.370100 12.456022 1.493660

H 3.689624 11.071063 0.420322

H 3.416416 12.522767 -2.269675

H 2.664596 10.971017 -1.911866

H 1.684498 12.319359 -2.519670

H -1.114248 10.152484 -2.680404

H -2.676282 9.338189 -2.698859

H -2.543872 10.977085 -2.052607

H -0.752070 7.695578 0.167376

H -1.510606 7.365624 -1.393474

H -3.721387 10.042377 0.082673

H -3.716812 8.439644 -0.652458

H -3.006751 8.667583 0.941966

H 2.490113 10.773165 12.140728

H 1.163384 9.621660 11.968230

H 2.330141 9.834138 10.658184

H 0.779706 12.607187 12.700761

H -0.475327 13.038489 11.536042

H -0.589506 11.528908 12.447828

H 0.329638 10.219815 9.097478

H -0.821124 10.150647 10.425983

H -0.778867 11.575242 9.387039

H 2.581686 15.663035 12.369754

H 3.881601 16.863914 12.432600

H 2.835195 16.776035 11.016269

H 5.699726 14.971036 12.797744

H 4.338666 13.851782 12.879667

H 5.659858 13.555616 11.744733

H 6.162292 15.282456 9.925556

H 4.950440 16.450016 9.424530

H 5.854385 16.721276 10.916547

H 6.053698 18.266328 11.205868

H 6.265139 19.835374 10.410464

H 6.925449 19.574574 12.021954

H 5.643115 21.678021 12.808532

H 4.913219 21.878014 11.213116

H 3.886238 21.759053 12.644335

H 5.508022 19.290376 13.991781

H 3.767631 19.566100 13.959940

H 4.437096 18.077492 13.265059

H 0.208067 20.988433 12.863828

H -0.511598 21.389809 11.303967

H -1.517080 20.735114 12.604418

H -1.264046 17.807560 10.290730

H -2.365441 18.856672 11.179839

H -1.467595 19.502310 9.806631

H 0.478160 18.563789 13.577717

H -1.209047 18.157519 13.271670

H 0.081253 17.248016 12.474270

S 8.965807 17.703816 5.091616

C 9.653023 19.445048 5.191588

C 8.854472 17.321019 3.311350

H 9.010785 19.861306 5.974891

C 9.420684 20.280427 3.939171

C 11.087478 19.466600 5.705832

H 9.284000 18.154991 2.743647

H 7.804953 17.223061 3.036768

C 9.629022 16.066330 2.939731

C 10.451140 20.566839 3.032403

C 8.135260 20.786982 3.680520

C 11.461737 20.492168 6.586493

C 12.053449 18.528076 5.316889

O 10.738481 15.774797 3.338048

O 8.932242 15.337766 2.054075

C 10.206282 21.344427 1.896317

H 11.454004 20.193528 3.219813

C 7.893808 21.563689 2.548191

H 7.325170 20.550254 4.360529

C 12.769636 20.586009 7.064825

H 10.720739 21.225012 6.898703

C 13.364313 18.624645 5.791600

H 11.778013 17.707735 4.660344

C 9.538588 14.097130 1.647784

C 8.928214 21.847671 1.651208

H 11.020650 21.559202 1.207240

H 6.893499 21.950079 2.368479

C 13.728089 19.651164 6.665999

H 13.037827 21.385592 7.752036

H 14.100894 17.886858 5.481376

H 8.876092 13.686694 0.886522

H 10.537307 14.274015 1.236427

H 9.600112 13.426429 2.506656

H 8.739511 22.457236 0.770177

H 14.747873 19.718334 7.039269

**TSS-09**

Mo 4.369378 15.268169 2.615838

Mo 5.358566 17.101438 5.727294

O 5.844751 13.594391 6.012606

O 7.924605 13.625345 4.593367

O 6.785576 15.727181 5.145981

O 5.718752 14.101737 3.601670

O 4.902019 14.897762 1.051490

O 3.007444 13.933432 3.098940

O 2.406073 15.127906 2.516938

O 4.143474 17.197902 2.555210

O 5.416422 16.901101 3.179165

O 3.969003 15.746356 5.073579

O 3.431828 17.072400 5.311086

O 5.310926 16.796285 7.400719

O 5.184165 18.956715 5.539573

O 6.839846 18.377454 5.461153

S 6.609730 14.183625 4.902374

C 2.947968 13.439258 6.573267

C 2.775471 13.373982 8.067949

C 1.900939 12.479558 8.695493

C 1.800862 12.453994 10.094463

C 0.877972 11.468665 10.821193

C 2.597825 13.327578 10.844216

C 3.486888 14.225973 10.228357

C 3.562928 14.249472 8.831727

C 2.258952 11.138976 5.590759

C 3.627493 10.819944 5.048993

C 4.467676 9.982119 5.800955

C 5.727088 9.633503 5.308336

C 6.160410 10.127323 4.072503

C 5.330396 10.972283 3.329960

C 4.062526 11.311412 3.812070

C 1.112099 10.716287 4.607424

C 0.312082 9.553432 5.146391

C 0.433624 8.303139 4.523198

C -0.274033 7.204627 5.019906

C -1.106351 7.349723 6.134089

C -1.231739 8.597862 6.753246

C -0.524900 9.698567 6.262676

C -0.858455 12.004284 3.552478

C -0.446106 11.666808 2.135726

C -1.245758 10.755323 1.425367

C -0.930673 10.425518 0.105159

C -1.768258 9.430606 -0.706795

C -0.963466 8.120506 -0.819503

C -3.125649 9.132130 -0.042629

C 0.212248 10.999005 -0.483031

C 1.020479 11.901931 0.212981

C 2.273547 12.530582 -0.412236

C 0.671996 12.243552 1.535765

C 0.839324 13.016997 5.202765

C 0.074609 14.888075 6.693855

C 0.934598 16.162737 6.791881

C 0.878097 16.369217 4.352723

C -0.012106 15.133703 4.205435

C 0.993202 18.413644 5.693910

C 2.775530 18.539882 7.554858

C 2.545605 18.903633 9.001190

C 3.675780 19.202209 9.774437

C 3.538625 19.509955 11.133746

C 4.752647 19.843786 12.009390

C 4.606330 19.161800 13.385122

C 4.799815 21.374984 12.179628

C 6.068175 19.357988 11.376968

C 2.251245 19.533405 11.691646

C 1.113357 19.221635 10.931528

C -0.257328 19.237509 11.617395

C -0.225474 18.247762 12.800213

C -0.534816 20.665845 12.127031

C -1.398000 18.826791 10.666540

C 1.269902 18.902200 9.576150

C 1.845282 20.556313 6.303856

C 1.155276 21.419064 7.332849

C 1.894724 22.409984 7.994057

C 1.268809 23.241739 8.927484

C -0.092082 23.085620 9.206585

C -0.828669 22.091243 8.553520

C -0.209012 21.258239 7.619038

C 1.175956 20.658009 4.892674

C 2.186260 20.872969 3.788526

C 2.080773 22.018356 2.983259

C 3.000021 22.232482 1.953987

C 4.026086 21.308239 1.720444

C 4.132617 20.170271 2.522943

C 3.214202 19.950187 3.555652

C -0.742058 19.178242 3.969815

C -0.528121 19.539676 2.517532

C -1.269468 20.587466 1.957898

C -1.107086 20.914735 0.604913

C -1.886977 22.059709 -0.051610

C -2.448988 21.592562 -1.409267

C -0.909123 23.233045 -0.260031

C -3.067522 22.534918 0.816233

C -0.174838 20.194333 -0.157416

C 0.585101 19.151753 0.395443

C 0.384223 18.817864 1.741097

N 2.009384 12.616486 5.791884

N 0.271268 11.958542 4.507588

N 0.079110 14.201971 5.370702

N 0.572084 17.066500 5.653744

N 1.727724 19.087143 6.654858

N 0.518624 19.311113 4.744859

C 4.318182 15.166438 11.110917

C 5.373788 15.938918 10.309878

C 5.039690 14.338449 12.195588

C 3.345433 16.163683 11.770341

C 1.762535 10.363587 11.431376

C -0.152845 10.822223 9.875003

C 0.106209 12.202517 11.936872

C 2.514601 12.053554 -1.858343

C 3.495850 12.144733 0.440368

C 2.103194 14.059692 -0.421545

C 1.585794 18.394587 -0.486781

C 0.778694 17.484197 -1.434053

C 2.554599 17.539529 0.343020

C 2.416759 19.405733 -1.303052

C -2.038940 10.007050 -2.111057

H 1.972577 16.064023 4.313856

H 0.750141 17.043661 3.470787

H 0.290874 14.615127 3.255963

H -1.087106 15.419820 4.123230

H 2.034377 15.905082 6.769084

H 0.725846 16.666007 7.764287

H 0.365341 14.185405 7.509791

H -1.000474 15.151436 6.869721

H 1.522898 10.471991 3.587328

H 2.120422 10.642097 6.594035

H 1.080088 8.180631 3.652544

H -0.631771 10.668983 6.746635

H -1.880336 8.712512 7.621895

H -1.656010 6.491263 6.520186

H -0.174704 6.232481 4.535857

H 6.379370 8.987309 5.894887

H 4.147683 9.608216 6.773301

H 3.421258 11.974776 3.212464

H 5.674628 11.392134 2.381878

H 7.150912 9.868450 3.698467

H -1.355164 13.002087 3.578532

H -1.623693 11.274167 3.925588

H -2.108708 10.307534 1.915299

H 1.299333 12.963358 2.083858

H 0.461626 10.722102 -1.508520

H -0.001881 8.276799 -1.320092

H -1.966894 21.144923 2.579798

H 0.953369 18.003223 2.205082

H -0.032389 20.454151 -1.208623

H -1.517986 19.833511 4.438319

H -1.138249 18.136669 4.050480

H 2.940267 20.818647 6.232011

H 0.392240 21.459603 4.874557

H -0.785433 20.481392 7.119987

H -1.888109 21.965733 8.775333

H -0.577912 23.736634 9.932962

H 1.846897 24.010627 9.440602

H 2.959252 22.534141 7.788554

H 1.282079 22.738679 3.150845

H 2.915351 23.119293 1.328238

H 4.736954 21.475827 0.912710

H 3.327902 19.039702 4.163963

H 4.922803 19.426174 2.359750

H 3.783155 18.935190 7.209633

H 2.869559 17.424885 7.453816

H 4.664286 19.177569 9.308044

H 2.132772 19.794834 12.744076

H 0.406620 18.674214 8.955787

H 4.257159 14.937295 8.322481

H 2.536310 13.306320 11.933171

H 1.311663 11.785901 8.102324

H 2.920589 14.507453 6.196923

H 4.013343 13.115738 6.306625

H 3.186776 18.896325 -1.892743

H 1.801703 19.981512 -2.000872

H 2.928909 20.116317 -0.642967

H 3.131168 18.159741 1.051119

H 2.043025 16.756572 0.920133

H 3.290816 17.024767 -0.288814

H 0.179479 16.758677 -0.873843

H 0.097731 18.057529 -2.069986

H 1.444770 16.915614 -2.092515

H -0.073621 22.953180 -0.910856

H -1.408704 24.091137 -0.721090

H -0.481378 23.570280 0.690044

H -2.733600 22.936485 1.778565

H -3.626024 23.333572 0.314743

H -3.774098 21.722268 1.014982

H -1.653280 21.331790 -2.115681

H -3.087451 20.710386 -1.293662

H -3.051586 22.376327 -1.880357

H 3.025179 14.554711 -0.757488

H 1.283774 14.380653 -1.066327

H 1.917265 14.450374 0.594586

H 4.399541 12.667649 0.092597

H 3.370100 12.456022 1.493660

H 3.689624 11.071063 0.420322

H 3.416416 12.522767 -2.269675

H 2.664596 10.971017 -1.911866

H 1.684498 12.319359 -2.519670

H -1.114248 10.152484 -2.680404

H -2.676282 9.338189 -2.698859

H -2.543872 10.977085 -2.052607

H -0.752070 7.695578 0.167376

H -1.510606 7.365624 -1.393474

H -3.721387 10.042377 0.082673

H -3.716812 8.439644 -0.652458

H -3.006751 8.667583 0.941966

H 2.490113 10.773165 12.140728

H 1.163384 9.621660 11.968230

H 2.330141 9.834138 10.658184

H 0.779706 12.607187 12.700761

H -0.475327 13.038489 11.536042

H -0.589506 11.528908 12.447828

H 0.329638 10.219815 9.097478

H -0.821124 10.150647 10.425983

H -0.778867 11.575242 9.387039

H 2.581686 15.663035 12.369754

H 3.881601 16.863914 12.432600

H 2.835195 16.776035 11.016269

H 5.699726 14.971036 12.797744

H 4.338666 13.851782 12.879667

H 5.659858 13.555616 11.744733

H 6.162292 15.282456 9.925556

H 4.950440 16.450016 9.424530

H 5.854385 16.721276 10.916547

H 6.053698 18.266328 11.205868

H 6.265139 19.835374 10.410464

H 6.925449 19.574574 12.021954

H 5.643115 21.678021 12.808532

H 4.913219 21.878014 11.213116

H 3.886238 21.759053 12.644335

H 5.508022 19.290376 13.991781

H 3.767631 19.566100 13.959940

H 4.437096 18.077492 13.265059

H 0.208067 20.988433 12.863828

H -0.511598 21.389809 11.303967

H -1.517080 20.735114 12.604418

H -1.264046 17.807560 10.290730

H -2.365441 18.856672 11.179839

H -1.467595 19.502310 9.806631

H 0.478160 18.563789 13.577717

H -1.209047 18.157519 13.271670

H 0.081253 17.248016 12.474270

S 9.000546 17.699751 5.260891

C 9.523830 19.473628 5.396289

C 8.855266 17.464517 3.453244

H 8.674159 20.049484 5.015526

C 10.758424 19.851046 4.590465

C 9.640536 19.757820 6.893474

H 9.379996 18.290560 2.957050

H 7.805205 17.483503 3.165620

C 9.519472 16.170705 3.002032

C 11.817130 18.967390 4.335522

C 10.852544 21.166587 4.110319

C 10.866739 19.703309 7.569006

C 8.478624 20.058248 7.621615

O 10.595982 15.763519 3.385295

O 8.764434 15.557995 2.076140

C 12.945703 19.397244 3.631555

H 11.747111 17.933514 4.658744

C 11.979729 21.596701 3.409376

H 10.035975 21.861190 4.294854

C 10.933124 19.952206 8.941926

H 11.777189 19.478252 7.020511

C 8.546484 20.305892 8.992893

H 7.523671 20.075300 7.109684

C 9.260999 14.292542 1.598933

C 13.034287 20.711751 3.169709

H 13.754779 18.696192 3.439635

H 12.031892 22.620929 3.046728

C 9.773567 20.254979 9.658672

H 11.894778 19.910727 9.449792

H 7.633084 20.531097 9.543641

H 8.578003 13.996702 0.803324

H 10.279813 14.401259 1.213953

H 9.240432 13.569072 2.415508

H 13.913067 21.042258 2.620082

H 9.826810 20.450040 10.727887
