# Supplementary material for: Bisguanidinium dinuclear oxodiperoxomolybdosulfate ion pair-catalyzed enantioselective sulfoxidation
Source: Nat Commun. 2016 Nov 21;7:13455. doi: 10.1038/ncomms13455 (PMC5121337; doi:10.1038/ncomms13455)

**checkCIF/PLATON report**

Structure factors have been supplied for datablock(s) tch66s

THIS REPORT IS FOR GUIDANCE ONLY. IF USED AS PART OF A REVIEW PROCEDURE

FOR PUBLICATION, IT SHOULD NOT REPLACE THE EXPERTISE OF AN EXPERIENCED

CRYSTALLOGRAPHIC REFEREE.

No syntax errors found. CIF dictionary Interpreting this report

**Datablock: tch66s-(*R*,*R*)-**1b

| Bond precision: | | C-C = 0.0160 A | | Wavelength=0.71073 | | |
| --- | --- | --- | --- | --- | --- | --- |
| Cell: | a=9.9398(8)  alpha=90 | | b=30.471(3) beta=97.837(3) | | | c=17.5604(15)  gamma=90 |
| Temperature: | 103 K | | | |  | |
|  | Calculated | | | | Reported | |
| Volume | 5268.9(8) | | | | 5268.9(8) | |
| Space group | P 21 | | | | P 1 21 1 | |
| Hall group | P 2yb | | | | P 2yb | |
| Moiety formula | C94 H124 N6, Mo2 O14 S, C4 H10 O, 2(C3 H7 N O) | | | | ? | |
| Sum formula | C104 H148 Mo2 N8 O17 S | | | | C104 H148 Mo2 N8 O17 S | |
| Mr | 2006.25 | | | | 2006.24 | |
| Dx,g cm-3 | 1.265 | | | | 1.265 | |
| Z | 2 | | | | 2 | |
| Mu (mm-1) | 0.324 | | | | 0.324 | |
| F000 | 2128.0 | | | | 2128.0 | |
| F000’ | 2122.20 | | | |  | |
| h,k,lmax | 12,37,21 | | | | 12,37,21 | |
| Nref | 20295[ 10353] | | | | 20085 | |
| Tmin,Tmax | 0.954,0.987 | | | | 0.830,0.990 | |
| Tmin’ | 0.873 | | | |  | |
| Correction method= # Reported T Limits: Tmin=0.830 Tmax=0.990  AbsCorr = MULTI-SCAN | | | | | | |
| Data completeness= 1.94/0.99 | | | | | Theta(max)= 25.820 | |
| R(reflections)=0.0715(12673)  S = 1.008 | | | wR2(reflections)= 0.1585(20085)  Npar= 1426 | | | |

The following ALERTS were generated. Each ALERT has the format

**test-name_ALERT_alert-type_alert-level**.

Click on the hyperlinks for more details of the test.

| - **Alert level C** | | |
| --- | --- | --- |
| PLAT090_ALERT_3_C | Poor Data / Parameter Ratio (Zmax > 18) ........ | 7.20 Note |
| PLAT213_ALERT_2_C | Atom C9 has ADP max/min Ratio ..... | 3.3 oblate |
| PLAT213_ALERT_2_C | Atom C50 has ADP max/min Ratio ..... | 3.3 prolat |
| PLAT213_ALERT_2_C | Atom C70 has ADP max/min Ratio ..... | 3.4 prolat |
| PLAT213_ALERT_2_C | Atom C93A has ADP max/min Ratio ..... | 3.1 prolat |
| PLAT213_ALERT_2_C | Atom C6A has ADP max/min Ratio ..... | 3.5 oblate |
| PLAT220_ALERT_2_C | Large Non-Solvent C Ueq(max)/Ueq(min) Range | 4.9 Ratio |
| PLAT222_ALERT_3_C | Large Non-Solvent H Uiso(max)/Uiso(min) ... | 4.9 Ratio |
| PLAT234_ALERT_4_C | Large Hirshfeld Difference C20 -- C21 .. | 0.20 Ang. |
| PLAT234_ALERT_4_C | Large Hirshfeld Difference C74 -- C79 .. | 0.16 Ang. |
| PLAT234_ALERT_4_C | Large Hirshfeld Difference S1 -- O1 .. | 0.17 Ang. |
| PLAT234_ALERT_4_C | Large Hirshfeld Difference S1 -- O2 .. | 0.16 Ang. |
| PLAT241_ALERT_2_C | High ’MainMol’ Ueq as Compared to Neighbors of | O3 Check |
| PLAT241_ALERT_2_C | High ’MainMol’ Ueq as Compared to Neighbors of | O4 Check |
| PLAT242_ALERT_2_C | Low ’MainMol’ Ueq as Compared to Neighbors of | S1 Check |
| PLAT243_ALERT_4_C | High ’Solvent’ Ueq as Compared to Neighbors of | C96 Check |
| PLAT243_ALERT_4_C | High ’Solvent’ Ueq as Compared to Neighbors of | C97 Check |
| PLAT244_ALERT_4_C | Low ’Solvent’ Ueq as Compared to Neighbors of | O15 Check |
| PLAT244_ALERT_4_C | Low ’Solvent’ Ueq as Compared to Neighbors of | N7 Check |
| PLAT250_ALERT_2_C | Large U3/U1 Ratio for Average U(i,j) Tensor .... | 2.1 Note |
| PLAT342_ALERT_3_C | Low Bond Precision on C-C Bonds ............... | 0.01601 Ang. |
| PLAT360_ALERT_2_C | Short C(sp3)-C(sp3) Bond C95 - C96 .. | 1.43 Ang. |
| PLAT413_ALERT_2_C | Short Inter XH3 .. XHn H10C .. H93D .. | 2.07 Ang. |

| - **Alert level G** | |
| --- | --- |
| [PLAT002_ALERT_2_G Number of Distance or Angle Restraints on AtSite](http://journals.iucr.org/services/cif/checking/PLAT002.html) | 57 Note |
| [PLAT003_ALERT_2_G Number of Uiso or Uij Restrained non-H Atoms ...](http://journals.iucr.org/services/cif/checking/PLAT003.html) | 51 Report |
| [PLAT172_ALERT_4_G The CIF-Embedded .res File Contains DFIX Records](http://journals.iucr.org/services/cif/checking/PLAT172.html) | 1 Report |
| [PLAT174_ALERT_4_G The CIF-Embedded .res File Contains FLAT Records](http://journals.iucr.org/services/cif/checking/PLAT174.html) | 3 Report |
| [PLAT175_ALERT_4_G The CIF-Embedded .res File Contains SAME Records](http://journals.iucr.org/services/cif/checking/PLAT175.html) | 10 Report |
| [PLAT176_ALERT_4_G The CIF-Embedded .res File Contains SADI Records](http://journals.iucr.org/services/cif/checking/PLAT176.html) | 3 Report |
| [PLAT178_ALERT_4_G The CIF-Embedded .res File Contains SIMU Records](http://journals.iucr.org/services/cif/checking/PLAT178.html) | 2 Report |
| [PLAT301_ALERT_3_G Main Residue Disorder ............ Percentage =](http://journals.iucr.org/services/cif/checking/PLAT301.html) | 14 Note |
| [PLAT302_ALERT_4_G Anion/Solvent Disorder ............ Percentage =](http://journals.iucr.org/services/cif/checking/PLAT302.html) | 33 Note |
| [PLAT304_ALERT_4_G Non-Integer Number of Atoms ( 6.16) in Resd. #](http://journals.iucr.org/services/cif/checking/PLAT304.html) | 5 Check |
| [PLAT304_ALERT_4_G Non-Integer Number of Atoms ( 5.84) in Resd. #](http://journals.iucr.org/services/cif/checking/PLAT304.html) | 6 Check |
| [PLAT432_ALERT_2_G Short Inter X...Y Contact O1 .. C103 ..](http://journals.iucr.org/services/cif/checking/PLAT432.html) | 3.00 Ang. |
| [PLAT432_ALERT_2_G Short Inter X...Y Contact O16 .. C50 ..](http://journals.iucr.org/services/cif/checking/PLAT432.html) | 3.00 Ang. |
| [PLAT720_ALERT_4_G Number of Unusual/Non-Standard Labels ..........](http://journals.iucr.org/services/cif/checking/PLAT720.html) | 15 Note |
| [PLAT791_ALERT_4_G The Model has Chirality at C16 (Chiral SPGR)](http://journals.iucr.org/services/cif/checking/PLAT791.html) | R Verify |
| [PLAT791_ALERT_4_G The Model has Chirality at C23 (Chiral SPGR)](http://journals.iucr.org/services/cif/checking/PLAT791.html) | R Verify |
| [PLAT791_ALERT_4_G The Model has Chirality at C66 (Chiral SPGR)](http://journals.iucr.org/services/cif/checking/PLAT791.html) | R Verify |
| [PLAT791_ALERT_4_G The Model has Chirality at C73 (Chiral SPGR)](http://journals.iucr.org/services/cif/checking/PLAT791.html) | R Verify |
| [PLAT850_ALERT_4_G Check Flack Parameter Exact Value 0.00 and s.u.](http://journals.iucr.org/services/cif/checking/PLAT850.html) | 0.02 Check |
| [PLAT860_ALERT_3_G Number of Least-Squares Restraints .............](http://journals.iucr.org/services/cif/checking/PLAT860.html) | 1029 Note |
| [PLAT910_ALERT_3_G Missing # of FCF Reflection(s) Below Th(Min) ...](http://journals.iucr.org/services/cif/checking/PLAT910.html) | 3 Report |
| [PLAT912_ALERT_4_G Missing # of FCF Reflections Above STh/L= 0.600](http://journals.iucr.org/services/cif/checking/PLAT912.html) | 83 Note |
| 0 **ALERT level A** = Most likely a serious problem - resolve or explain  0 **ALERT level B** = A potentially serious problem, consider carefully  23 **ALERT level C** = Check. Ensure it is not caused by an omission or oversight  22 **ALERT level G** = General information/check it is not something unexpected  0 ALERT type 1 CIF construction/syntax error, inconsistent or missing data  16 ALERT type 2 Indicator that the structure model may be wrong or deficient  6 ALERT type 3 Indicator that the structure quality may be low  23 ALERT type 4 Improvement, methodology, query or suggestion  0 ALERT type 5 Informative message, check | |

It is advisable to attempt to resolve as many as possible of the alerts in all categories. Often the

minor alerts point to easily fixed oversights, errors and omissions in your CIF or refinement

strategy, so attention to these fine details can be worthwhile. In order to resolve some of the more

serious problems it may be necessary to carry out additional measurements or structure

refinements. However, the purpose of your study may justify the reported deviations and the more

serious of these should normally be commented upon in the discussion or experimental section of a

paper or in the "special_details" fields of the CIF. checkCIF was carefully designed to identify

outliers and unusual parameters, but every test has its limitations and alerts that are not important

in a particular case may appear. Conversely, the absence of alerts does not guarantee there are no

aspects of the results needing attention. It is up to the individual to critically assess their own

results and, if necessary, seek expert advice.

**Publication of your CIF in IUCr journals**

A basic structural check has been run on your CIF. These basic checks will be run on all CIFs

submitted for publication in IUCr journals (*Acta Crystallographica*, *Journal of Applied*

*Crystallography*, *Journal of Synchrotron Radiation*); however, if you intend to submit to *Acta*

*Crystallographica Section C* or *E* or *IUCrData*, you should make sure that full publication checks

are run on the final version of your CIF prior to submission.

**Publication of your CIF in other journals**

Please refer to the *Notes for Authors* of the relevant journal for any special instructions relating to

CIF submission.

**PLATON version of 19/11/2015; check.def file version of 17/11/2015**

**Datablock tch66s**-(*R*,*R*)-**1b** - ellipsoid plot


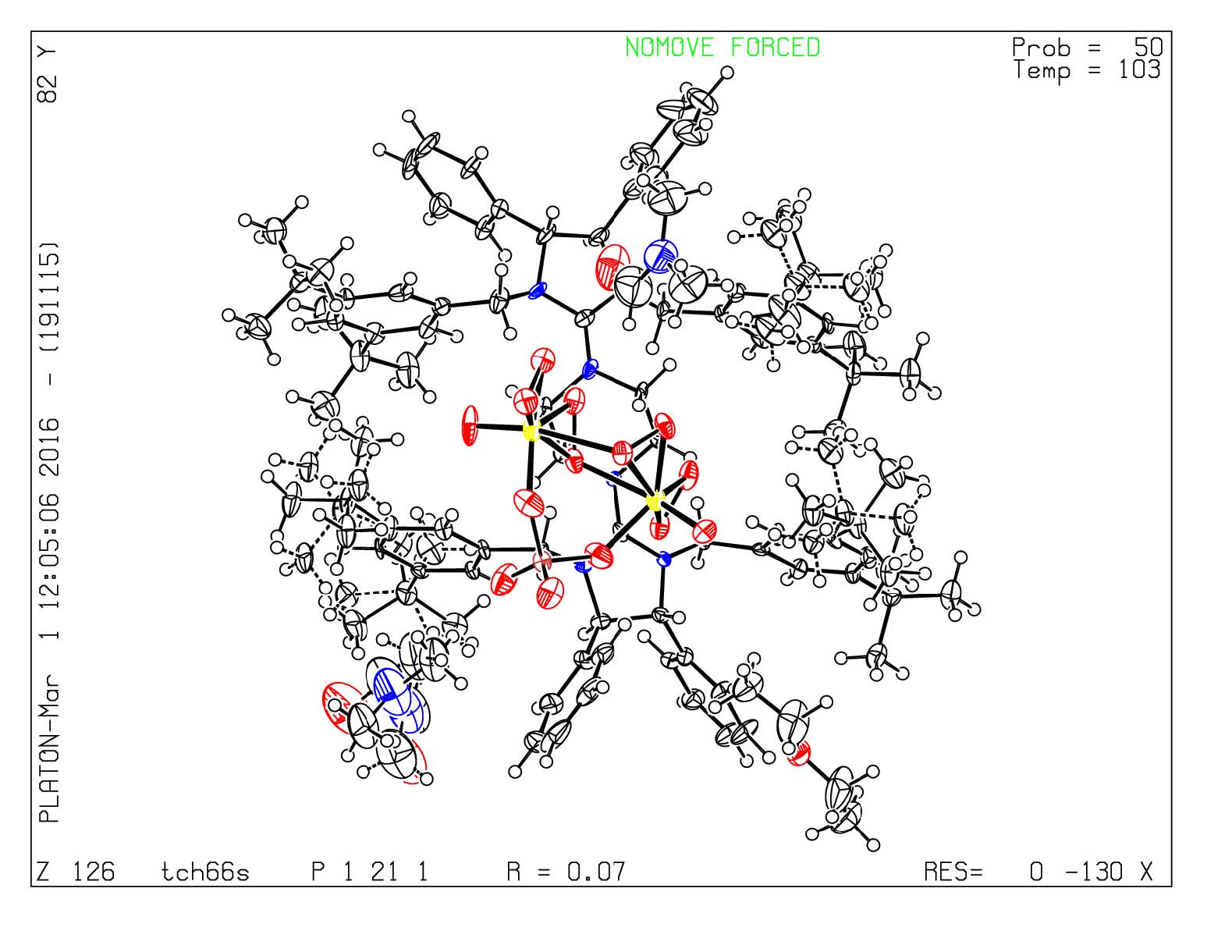

Supplement: Supplementary Data 2 — IUCR's CheckCIF report of compound 1b. [file ncomms13455-s3.doc]
