# Supplementary material for: Bisguanidinium dinuclear oxodiperoxomolybdosulfate ion pair-catalyzed enantioselective sulfoxidation
Source: Nat Commun. 2016 Nov 21;7:13455. doi: 10.1038/ncomms13455 (PMC5121337; doi:10.1038/ncomms13455)

**checkCIF/PLATON report**

Structure factors have been supplied for datablock(s) tch64s

THIS REPORT IS FOR GUIDANCE ONLY. IF USED AS PART OF A REVIEW PROCEDURE

FOR PUBLICATION, IT SHOULD NOT REPLACE THE EXPERTISE OF AN EXPERIENCED

CRYSTALLOGRAPHIC REFEREE.

No syntax errors found. CIF dictionary Interpreting this report

**Datablock: tch64s-3f**

| Bond precision: | | C-C = 0.0044 A | | Wavelength=0.71073 | | |
| --- | --- | --- | --- | --- | --- | --- |
| Cell: | a=5.2790(5)  alpha=90 | | b=11.5315(8)  beta=90 | | | c=23.4325(19)  gamma=90 |
| Temperature: | 103 K | | | |  | |
|  | Calculated | | | | Reported | |
| Volume | 1426.5(2) | | | | 1426.4(2) | |
| Space group | P 21 21 21 | | | | P 21 21 21 | |
| Hall group | P 2ac 2ab | | | | P 2ac 2ab | |
| Moiety formula | C13 H17 Cl O3 S | | | | ? | |
| Sum formula | C13 H17 Cl O3 S | | | | C13 H17 Cl O3 S | |
| Mr | 288.78 | | | | 288.77 | |
| Dx,g cm-3 | 1.345 | | | | 1.345 | |
| Z | 4 | | | | 4 | |
| Mu (mm-1) | 0.412 | | | | 0.412 | |
| F000 | 608.0 | | | | 608.0 | |
| F000’ | 609.34 | | | |  | |
| h,k,lmax | 6,14,30 | | | | 6,14,30 | |
| Nref | 3282[ 1927] | | | | 3281 | |
| Tmin,Tmax | 0.888,0.944 | | | | 0.800,0.940 | |
| Tmin’ | 0.841 | | | |  | |
| Correction method= # Reported T Limits: Tmin=0.800 Tmax=0.940  AbsCorr = MULTI-SCAN | | | | | | |
| Data completeness= 1.70/1.00 | | | | | Theta(max)= 27.510 | |
| R(reflections)= 0.0377(3001)  S = 1.080 | | | wR2(reflections)= 0.0808(3281)  Npar= 242 | | | |

The following ALERTS were generated. Each ALERT has the format

**test-name_ALERT_alert-type_alert-level**.

Click on the hyperlinks for more details of the test.

| - **Alert level C** | | |
| --- | --- | --- |
| PLAT340_ALERT_3_C | Low Bond Precision on C-C Bonds ............... | 0.00443 Ang. |

| - **Alert level G** | |
| --- | --- |
| [PLAT002_ALERT_2_G Number of Distance or Angle Restraints on AtSite](http://journals.iucr.org/services/cif/checking/PLAT002.html) | 17 Note |
| [PLAT003_ALERT_2_G Number of Uiso or Uij Restrained non-H Atoms ...](http://journals.iucr.org/services/cif/checking/PLAT003.html) | 16 Report |
| [PLAT175_ALERT_4_G The CIF-Embedded .res File Contains SAME Records](http://journals.iucr.org/services/cif/checking/PLAT175.html) | 1 Report |
| [PLAT176_ALERT_4_G The CIF-Embedded .res File Contains SADI Records](http://journals.iucr.org/services/cif/checking/PLAT176.html) | 1 Report |
| [PLAT178_ALERT_4_G The CIF-Embedded .res File Contains SIMU Records](http://journals.iucr.org/services/cif/checking/PLAT178.html) | 1 Report |
| [PLAT301_ALERT_3_G Main Residue Disorder ............ Percentage =](http://journals.iucr.org/services/cif/checking/PLAT301.html) | 44 Note |
| [PLAT720_ALERT_4_G Number of Unusual/Non-Standard Labels ..........](http://journals.iucr.org/services/cif/checking/PLAT720.html) | 2 Note |
| [PLAT811_ALERT_5_G No ADDSYM Analysis: Too Many Excluded Atoms ....](http://journals.iucr.org/services/cif/checking/PLAT811.html) | ! Info |
| [PLAT860_ALERT_3_G Number of Least-Squares Restraints .............](http://journals.iucr.org/services/cif/checking/PLAT860.html) | 300 Note |
| 0 **ALERT level A** = Most likely a serious problem - resolve or explain  0 **ALERT level B** = A potentially serious problem, consider carefully  1 **ALERT level C** = Check. Ensure it is not caused by an omission or oversight  9 **ALERT level G** = General information/check it is not something unexpected  0 ALERT type 1 CIF construction/syntax error, inconsistent or missing data  2 ALERT type 2 Indicator that the structure model may be wrong or deficient  3 ALERT type 3 Indicator that the structure quality may be low  4 ALERT type 4 Improvement, methodology, query or suggestion  1 ALERT type 5 Informative message, check | |

It is advisable to attempt to resolve as many as possible of the alerts in all categories. Often the

minor alerts point to easily fixed oversights, errors and omissions in your CIF or refinement

strategy, so attention to these fine details can be worthwhile. In order to resolve some of the more

serious problems it may be necessary to carry out additional measurements or structure

refinements. However, the purpose of your study may justify the reported deviations and the more

serious of these should normally be commented upon in the discussion or experimental section of a

paper or in the "special_details" fields of the CIF. checkCIF was carefully designed to identify

outliers and unusual parameters, but every test has its limitations and alerts that are not important

in a particular case may appear. Conversely, the absence of alerts does not guarantee there are no

aspects of the results needing attention. It is up to the individual to critically assess their own

results and, if necessary, seek expert advice.

**Publication of your CIF in IUCr journals**

A basic structural check has been run on your CIF. These basic checks will be run on all CIFs

submitted for publication in IUCr journals (*Acta Crystallographica*, *Journal of Applied*

*Crystallography*, *Journal of Synchrotron Radiation*); however, if you intend to submit to *Acta*

*Crystallographica Section C* or *E* or *IUCrData*, you should make sure that full publication checks

are run on the final version of your CIF prior to submission.

**Publication of your CIF in other journals**

Please refer to the *Notes for Authors* of the relevant journal for any special instructions relating to

CIF submission.

**PLATON version of 19/11/2015; check.def file version of 17/11/2015**

**Datablock tch64s-3f** - ellipsoid plot


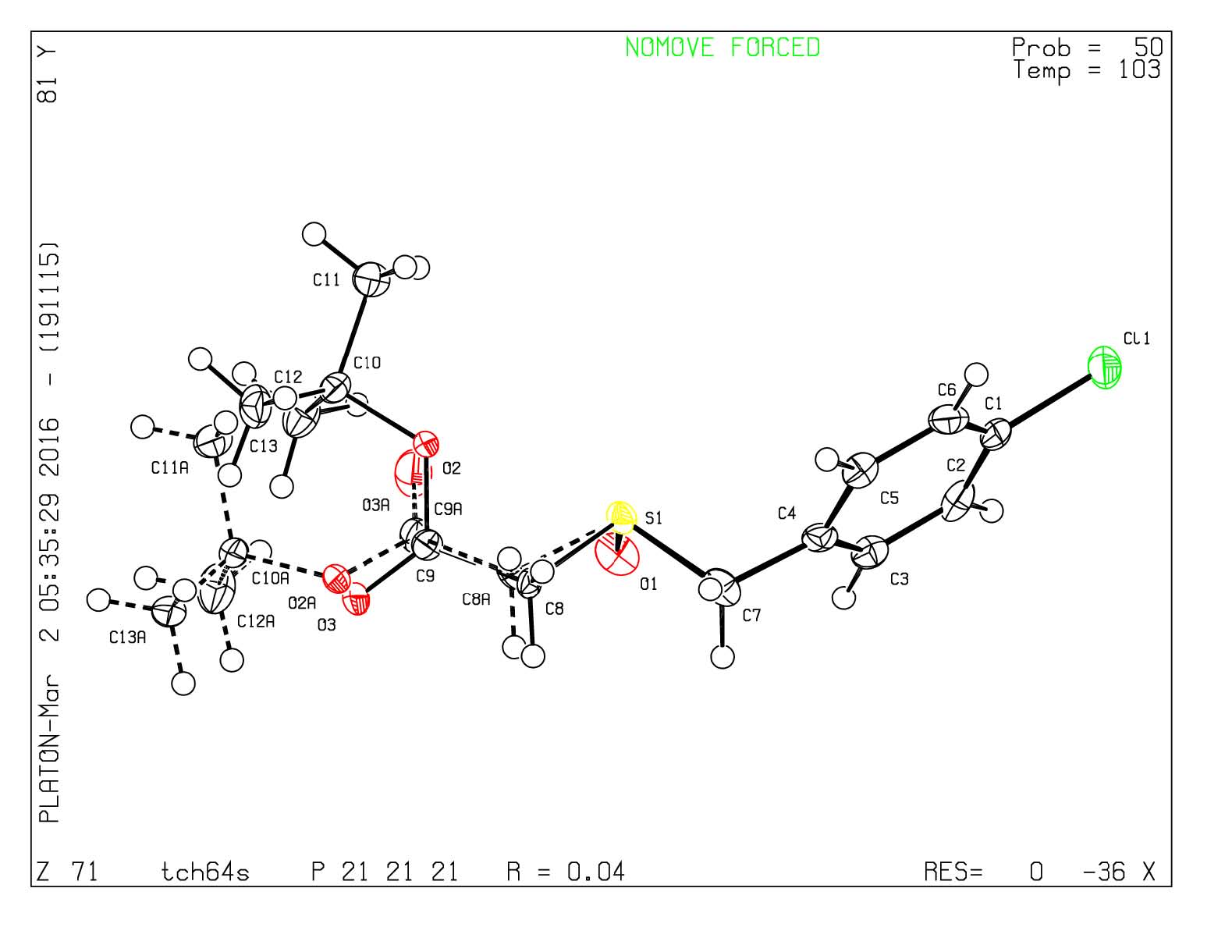

Supplement: Supplementary Data 4 — IUCR's CheckCIF report of compound 3f. [file ncomms13455-s5.doc]
