# Supplementary material for: Bisguanidinium dinuclear oxodiperoxomolybdosulfate ion pair-catalyzed enantioselective sulfoxidation
Source: Nat Commun. 2016 Nov 21;7:13455. doi: 10.1038/ncomms13455 (PMC5121337; doi:10.1038/ncomms13455)

**checkCIF/PLATON report**

Structure factors have been supplied for datablock(s) tch65s

THIS REPORT IS FOR GUIDANCE ONLY. IF USED AS PART OF A REVIEW PROCEDURE

FOR PUBLICATION, IT SHOULD NOT REPLACE THE EXPERTISE OF AN EXPERIENCED

CRYSTALLOGRAPHIC REFEREE.

No syntax errors found. CIF dictionary Interpreting this report

**Datablock: tch65s-3o**

| Bond precision: | | C-C = 0.0169 A | | | Wavelength=0.71073 | | |
| --- | --- | --- | --- | --- | --- | --- | --- |
| Cell: | a=26.8296(16) alpha=90 | | b=9.6925(5) beta=101.2326(19) | | | | c=10.5106(6))  gamma=90 |
| Temperature: | 103 K | | | | |  | |
|  | Calculated | | | | | Reported | |
| Volume | 2680.9(3) | | | | | 2680.9(3) | |
| Space group | P 21 | | | | | P 1 21 1 | |
| Hall group | P 2yb | | | | | P 2yb | |
| Moiety formula | C12 H15 Br O3 S | | | | | ? | |
| Sum formula | C12 H15 Br O3 S | | | | | C12 H15 Br O3 S | |
| Mr | 319.20 | | | | | 319.21 | |
| Dx,g cm-3 | 1.582 | | | | | 1.582 | |
| Z | 8 | | | | | 8 | |
| Mu (mm-1) | 3.216 | | | | | 3.216 | |
| F000 | 1296.0 | | | | | 1296.0 | |
| F000’ | 1295.26 | | | | |  | |
| h,k,lmax | 43,15,16 | | | | | 42,15,16 | |
| Nref | 23605[ 12367] | | | | | 22922 | |
| Tmin,Tmax | 0.282,0.680 | | | | | 0.370,0.700 | |
| Tmin’ | 0.238 | | | | |  | |
| Correction method= # Reported T Limits: Tmin=0.370 Tmax=0.700  AbsCorr = MULTI-SCAN | | | | | | | |
| Data completeness= 1.85/0.97 | | | | | | Theta(max)= 35.010 | |
| R(reflections)= 0.0620(10957)  S = 0.949 | | | | wR2(reflections)= 0.1213(22922)  Npar= 626 | | | |

The following ALERTS were generated. Each ALERT has the format

**test-name_ALERT_alert-type_alert-level**.

Click on the hyperlinks for more details of the test.

| - **Alert level B** | | |
| --- | --- | --- |
| PLAT341_ALERT_3_B | Low Bond Precision on C-C Bonds ............... | 0.01685 Ang. |

| - **Alert level C** | | |
| --- | --- | --- |
| RINTA01_ALERT_3_C | The value of Rint is greater than 0.12  Rint given 0.127 |  |
| PLAT026_ALERT_3_C | Ratio Observed / Unique Reflections (too) Low .. | 48 % |
| PLAT112_ALERT_2_C | ADDSYM Detects New (Pseudo) Symm. Elem. c/2 | 83 %Fit |

| - **Alert level G** | |
| --- | --- |
| [PLAT003_ALERT_2_G Number of Uiso or Uij Restrained non-H Atoms ... PLAT158_ALERT_4_G The Input Unitcell is NOT Standard/Reduced ..... PLAT860_ALERT_3_G Number of Least-Squares Restraints ............. PLAT870_ALERT_4_G ALERTS Related to Twinning Effects Suppressed .. PLAT910_ALERT_3_G Missing # of FCF Reflection(s) Below Th(Min) ... PLAT912_ALERT_4_G Missing # of FCF Reflections Above STh/L= 0.600 PLAT931_ALERT_5_G Found Twin Law ( 1 0 0)[ ] Estimated BASF](http://journals.iucr.org/services/cif/checking/PLAT003.html) | 68 Report Please Check 493 Note  ! Info  2 Report  177 Note  0.30 Check |
| 0 **ALERT level A** = Most likely a serious problem - resolve or explain  1 **ALERT level B** = A potentially serious problem, consider carefully  3 **ALERT level C** = Check. Ensure it is not caused by an omission or oversight  7 **ALERT level G** = General information/check it is not something unexpected  0 ALERT type 1 CIF construction/syntax error, inconsistent or missing data  2 ALERT type 2 Indicator that the structure model may be wrong or deficient  5 ALERT type 3 Indicator that the structure quality may be low  3 ALERT type 4 Improvement, methodology, query or suggestion  1 ALERT type 5 Informative message, check  **Validation response form**  Please find below a validation response form (VRF) that can be filled in and pasted into your CIF.  # start Validation Reply Form  _vrf_PLAT341_tch65s  ;  PROBLEM: Low Bond Precision on C-C Bonds ............... 0.01685 Ang.  RESPONSE: ...  ;  # end Validation Reply Form | |

It is advisable to attempt to resolve as many as possible of the alerts in all categories. Often the

minor alerts point to easily fixed oversights, errors and omissions in your CIF or refinement

strategy, so attention to these fine details can be worthwhile. In order to resolve some of the more

serious problems it may be necessary to carry out additional measurements or structure

refinements. However, the purpose of your study may justify the reported deviations and the more

serious of these should normally be commented upon in the discussion or experimental section of a

paper or in the "special_details" fields of the CIF. checkCIF was carefully designed to identify

outliers and unusual parameters, but every test has its limitations and alerts that are not important

in a particular case may appear. Conversely, the absence of alerts does not guarantee there are no

aspects of the results needing attention. It is up to the individual to critically assess their own

results and, if necessary, seek expert advice.

**Publication of your CIF in IUCr journals**

A basic structural check has been run on your CIF. These basic checks will be run on all CIFs

submitted for publication in IUCr journals (*Acta Crystallographica*, *Journal of Applied*

*Crystallography*, *Journal of Synchrotron Radiation*); however, if you intend to submit to *Acta*

*Crystallographica Section C* or *E* or *IUCrData*, you should make sure that full publication checks

are run on the final version of your CIF prior to submission.

**Publication of your CIF in other journals**

Please refer to the *Notes for Authors* of the relevant journal for any special instructions relating to

CIF submission.

**PLATON version of 19/11/2015; check.def file version of 17/11/2015**

**Datablock tch65s-3o** - ellipsoid plot


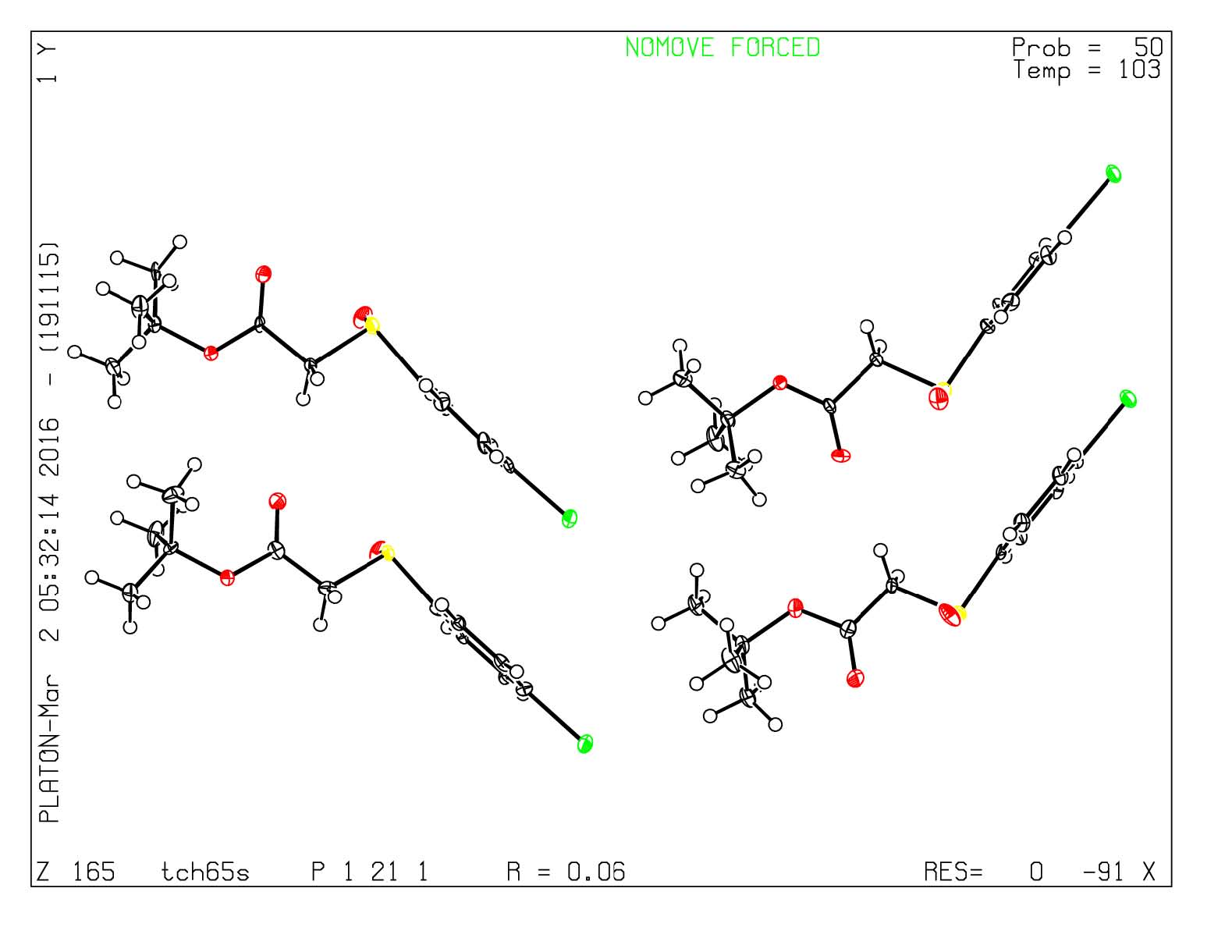

Supplement: Supplementary Data 6 — IUCR's CheckCIF report of compound 3o. [file ncomms13455-s7.doc]
